# Synthesis of New Thiazole-Privileged Chalcones as Tubulin Polymerization Inhibitors with Potential Anticancer Activities

**DOI:** 10.3390/ph17091154

**Published:** 2024-08-31

**Authors:** Hamada Hashem, Abdelfattah Hassan, Walid M. Abdelmagid, Ahmed G. K. Habib, Mohamed A. A. Abdel-Aal, Ali M. Elshamsy, Amr El Zawily, Ibrahim Taha Radwan, Stefan Bräse, Ahmed S. Abdel-Samea, Safwat M. Rabea

**Affiliations:** 1 Pharmaceutical Chemistry Department, Faculty of Pharmacy, Sohag University, Sohag 82524, Egypt; 2 Medicinal Chemistry Department, Faculty of Pharmacy, South Valley University, Qena 52242, Egypt; 3 Medicinal Chemistry Department, Clinical Pharmacy Program, South Valley National University, Qena 52242, Egypt; 4 Medicinal Chemistry and Drug Discovery Research Centre, Swenam College, 210-6125 Sussex Avenue, Burnaby, BC V5H 4G1, Canada; 5 Department of Biotechnology and Life Sciences, Faculty of Postgraduate Studies for Advanced Sciences, Beni-Suef University, Beni-Suef 62521, Egypt; 6 Pharmaceutical Chemistry Department, Faculty of Pharmacy, Al-Azhar University, Assiut Branch, Assiut 71524, Egypt; 7 Medicinal Chemistry Department, Faculty of Pharmacy, Deraya University, New Minia 61768, Egypt; 8 Department of Plant and Microbiology, Faculty of Science, Damanhour University, Damanhour 22511, Egypt; 9 Division of Pharmaceutics and Translation Therapeutics, College of Pharmacy, University of Iowa, Iowa City, IA 52242, USA; 10 Supplementary General Sciences Department, Faculty of Oral and Dental Medicine, Future University in Egypt, Cairo 11835, Egypt; 11 Institute of Biological and Chemical Systems—Functional Molecular Systems (IBCS-FMS), Karlsruhe Institute of Technology (KIT), Kaiserstrasse 12, 76131 Karlsruhe, Germany; 12 Pharmacology and Toxicology Department, Faculty of Pharmacy, Deraya University, New Minia 61768, Egypt; 13 Medicinal Chemistry Department, Faculty of Pharmacy, Minia University, Minia 61519, Egypt; 14 Apogee Pharmaceuticals Inc., 4475 Wayburne Dr., Suite 105, Burnaby, BC V6V2H8, Canada

**Keywords:** thiazole chalcones, anticancer, tubulin inhibitors, colchicine binding site

## Abstract

A series of novel thiazole-based chalcones were evaluated for their anticancer activity as potential tubulin polymerization inhibitors. In vitro anticancer screening for the thiazole derivatives **2a**–**2p** exhibited broad-spectrum antitumor activity against various cancer cell lines particularly Ovar-3 and MDA-MB-468 cells with a GI_50_ range from 1.55 to 2.95 μΜ, respectively. Compound **2e** demonstrated significant inhibition of tubulin polymerization, with an IC_50_ value of 7.78 μM compared to Combretastatin-A4 (CA-4), with an IC_50_ value of 4.93 μM. Molecular docking studies of compounds **2e**, **2g**, and **2h** into tubulin further supported these findings, revealing that they bind effectively to the colchicine binding site, mirroring key interactions exhibited by CA-4. Computational predictions suggested favorable oral bioavailability and drug-likeness for these compounds, highlighting their potential for further development as chemotherapeutic agents.

## 1. Introduction

Microtubules are dynamic cytoskeletal elements in human cells, involved in cellular activities throughout cell division [1]. The highly dynamic behavior of microtubules can be successfully targeted to combat rapidly replicating cancer cells [1,2]. Tumor cells possess unique traits such as unlimited growth, angiogenesis, adaptability, and effortless spread throughout the body [3,4]. These features strongly rely on the involvement of microtubules, making microtubules an essential target for treating cancer [5,6]. Antimitotic drugs are a variety of cyclic compounds that interfere with cell division [7,8] polymerization binding. Traditional antimitotic drugs directly bind to tubulin to stabilize formed microtubules or prevent tubulin from polymerizing to form microtubules. These agents create abnormalities in the mitotic spindle, leading to an extended pause in mitosis that initiates apoptosis [7,9]. The effectiveness of antimitotic therapies indicates that focusing on mitosis is a promising strategy for creating novel anticancer medications [10].

Antimitotic drugs that target tubulin bind at four distinct binding sites, taxanes, vinca alkaloids, colchicine, and laulimalide sites [11,12]. Tubulin inhibitors that target vinca alkaloids and taxane sites, like paclitaxel, vinblastine, and ixabepilone, have been commonly used in medical practice for years [13,14]. Nevertheless, due to their limited water solubility, narrow therapeutic range, and the development of drug resistance, there is a push to find safer and more potent antimitotic drugs [15,16,17].

Colchicine, a natural product, binds to a different location on tubulin and successfully prevents tubulin assembly [14]. Colchicine is not utilized clinically due to its significant toxicity [18]. Additionally, there are presently no FDA-approved tubulin inhibitors that target the colchicine site [19]. Hence, it is crucial to create new antimitotic drugs that target the colchicine binding site [20]. There is a growing interest in antimitotic agents that interact with the colchicine binding site because they are simple molecules with enhanced solubility in water and a wide therapeutic range [13,21]. Compared to other binding sites, targeting the colchicine binding site is reported to cause a rapid disruption of existing tumor vasculature and decrease multidrug resistance [21,22]. In addition, CA-4 is a potent antimitotic agent that binds to the colchicine binding site and suppresses tubulin assembly [23,24]. Nevertheless, the in vivo efficacy of CA-4 is limited due to its unfavorable pharmacokinetics, which is caused by its high hydrophobicity, low aqueous solubility, and isomerism to a less active *E*-isomer [11,17].

**Figure 1:**
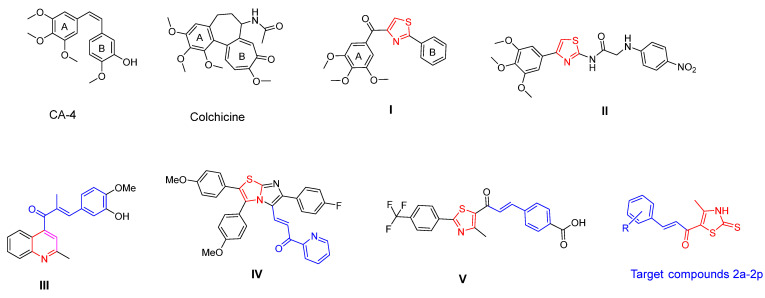
Some reported heterocyclic-chalcones and thiazole derivatives targeting colchicine binding site on tubulin.

Chalcones are an important group of flavonoid compounds with unique structures in medicinal chemistry [23,25]. Due to their uncomplicated structure and anticancer characteristics, chalcones can be easily hybridized with other anticancer pharmacophores, creating several bioactive derivatives [15,25]. Hundreds of chalcone derivatives were synthesized and evaluated as tubulin inhibitors [26]. Thiazole-linked chalcone V revealed remarkable anticancer activity against the colorectal cancer cells, HT-29, HCT-116, and Lovo, with IC_50_ values of 7.94, 3.12, and 2.21 μM, respectively [27]. On the other hand, thiazole was incorporated into many chemotherapeutic agents due to its favorable pharmacokinetic and pharmacodynamic characteristics [3,28]. Various clinically used anticancer drugs contain thiazole rings like bleomycin, ixabepilone, and dasatinib [29]. Bioactive thiazole enhances the binding to target, molecular conformation, water solubility, physicochemical properties, and pharmacokinetic properties [3,30]. Many CA-4 analogs such as tubulin polymerization inhibitors have been reported as replacing the double bond of CA-4 with a thiazole ring to maintain the cis-conformation, for example, compounds **I** and **II** [31,32]. Several research teams have designed heterocycle–chalcone hybrids to improve both the pharmacokinetics and pharmacodynamics of chalcones as antimitotic agents [33,34]. Li and coworkers incorporated a quinoline moiety in chalcone to improve physicochemical properties as compound **III** [21]. Conversely, Kamal and his team designed imidazothiazole-chalcone **IV** that showed enhanced binding interactions with the colchicine binding region in the tubulin dimer, compared to CA-4 (Figure 1) [27,32,35].

**Figure 2:**
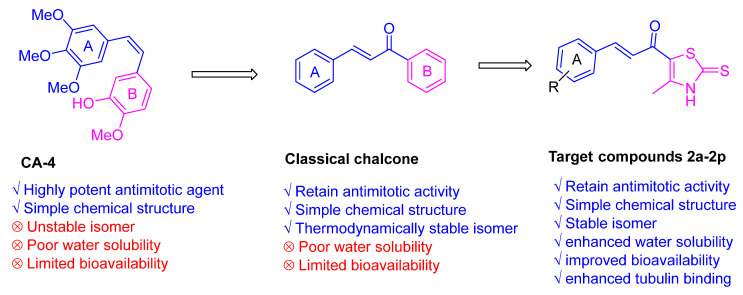
Design of novel thiazole-chalcones targeting colchicine binding site.

Motivated by these findings and continuing our endeavors to create novel derivatives with anticancer activity, we designed and synthesized a range of thiazole–chalcone derivatives as potential antimitotic agents (Figure 2). Subsequently, we assessed the effectiveness of these derivatives in inhibiting the growth of different types of human cancer cells. To investigate the mechanism by which these derivatives exhibit their anticancer activities, their impact on tubulin polymerization was evaluated.

## 2. Results and Discussion

### 2.1. Chemistry

**Scheme 1:**
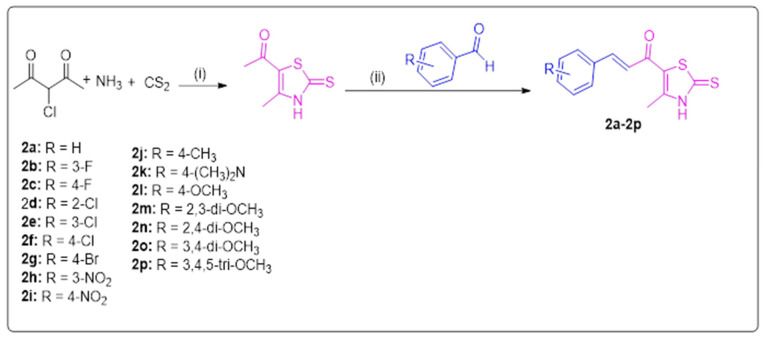
Synthesis of thiazole-based chalcones **2a**–**2p**. **Reagents and conditions**: (**i**) ethanol, 20 °C, 6 h.; (**ii**) appropriate aromatic aldehyde, 60% NaOH, ethanol, 0 °C, 18 h.

Both thiazole chalcones **2a**–**2p** and their intermediate **1** were prepared as illustrated in Scheme 1. 1-(4-methyl-2-thioxo-2,3-dihydrothiazol-5-yl)ethan-1-one was synthesized following the previously published procedure [36]. The thiazole derivatives **2a**–**2p** were synthesized in ethanol by condensation of intermediate **1** with the appropriate aromatic aldehyde in the presence of sodium hydroxide [2]. The structures of target compounds **2a**–**2p** were elucidated using ^1^H NMR, ^13^ C NMR, elemental analysis, and mass spectrometry. The ^1^H NMR spectra of chalcones derivatives **2a**–**2p** showed a singlet at δ = 2.50–2.60 ppm due to the methyl group of the thiazole ring. Also, two doublets appeared at δ = 7.10–7.80 ppm due to chalcone protons. One singlet for the NH appeared at δ 13.63–13.70 ppm. Moreover, ^13^ C NMR showed characteristic signals at δ = 188.91–189.44 ppm, 179.87–180.24 ppm, and 14.14–15.02 ppm, which corresponds to C=S, C=O, and the methyl group of the chalcone scaffold, respectively.

### 2.2. Biological Investigation

#### 2.2.1. In Vitro Screening of Anticancer Activity of Thiazole Derivatives **2a**–**2p** at 10 μM

According to the protocol of the drug evaluation, all thiazole chalcones **2a**–**2p** were selected by the National Cancer Institute (NCI) for in vitro screening of their anticancer activities at a single dose of 10 μM against nine tumor subpanels, including leukemia, CNS, melanoma, colon, lung, breast, ovarian, renal, and prostate cancer (PC) cell lines (Table 1). From the results in Table 1, it is clear that all tested thiazole derivatives displayed a broad range of antiproliferative and cytotoxic activity against most of the tested cell lines, with mean growth percentages ranging from −21.75 to 77.71. Chalcone derivatives **2c**, **2e**, **2f, 2g**, **2h**, **2i**, and **2p** showed remarkable anticancer activity against most of the tested cell lines with mean growth percentages equal 36.74, 22.13, 23.72, 34.25, −21.75, 25.23, and 14.89, respectively. Among the tested derivatives, compound **2h** was the most potent derivative, with broad cytotoxic effect (negative value) against LOX IMVI, RXF 393, UO-31, HCC-2998, SF-539, MDA-MB-468, OVCAR-3, KM12, U251, NCI-H23, SK-MEL-5, NCI-H522, ACHN, UACC-62, T-47D, CAKI-1, SK-MEL-28, MCF7, MALME-3M, NCI-H226, SW-620, OVCAR-5, MDA-MB-435, HCT-15, NCI-H460, SN12C, COLO 205, MDA-MB-231/ATCC, BT-549, SR, 786-0, and HCT-116 cancer cells with growth percentages of −98.99, −91.92, −89.63, −86.72, −85.88, −82.86, −79.48, −78.4, −75.99, −72.77, −68.8, −58.81, −58.35, −56.71, −56.63, −56.46, −47.02, −45.42, −42.94, −41.9, −37.8, −37.4, −36.95, −36.42, −30.68, −25.64, −24.88, −21.75, −17.58, −14.03, −10.99, −10.85, and −6.25, respectively. Also, it displayed remarkable cytostatic action (positive value) against EKVX, HOP-62, M14, SF-295, HL-60(TB), NCI/ADR-RES, PC-3, DU-145, RPMI-8226, UACC-257, IGROV1, SNB-19, OVCAR-4, MOLT-4, OVCAR-8, K-562, CCRF-CEM, SK-MEL-2, A549/ATCC, HT29, HS 578T, SF-268, NCI-H322M, SK-OV-3, and A498 cancer cells with growth inhibition percentages of 0.65, 0.68, 1.86, 4.14, 5.25, 8.19, 8.43, 8.55, 9.39, 9.4, 10.16, 10.38, 13.05, 13.31, 13.44, 13.77, 13.78, 16.88, 19.51, 20.35, 24.47, 27.46, 28.92, 44.61, and 8.87, respectively. Thiazole chalcones **2c**, **2e**, **2f**, **2g**, **2i**, and **2p** exhibited broad and cytotoxic effects against most of the tested cells with growth percentages of −13.63 to 119.09, −26.09 to 98.01, −48.15 to 105.85, −34.08 to 108.90, −86.21 to 107.77, and −64.57 to 96.50, respectively. Thiazole derivatives **2a**, **2b**, **2d**, **2j**, **2k**, **2l**, **2m**, **2n**, and **2o** showed moderate potency against most of the tested cancer cell lines with growth percentages of 14.13 to 128.73, −22.69 to 106.03, 14.10 to 116.44, 11.77 to 130.77, 35.38 to 129.94, 15.11 to 120.21, 5.15 to 108.77, 19.19 to 122.75, −0.42 to 125.58, respectively. The data presented in Table 1 showed that substituting the phenyl ring of thiazole chalcones greatly impacts the potency against various cancer cell lines. The presence of an electron-withdrawing group increases cytotoxic activity. In contrast, an electron-donating group decreases the anticancer activity of thiazole derivatives.

#### 2.2.2. Screening of the Anticancer Activity at Five Doses

The findings from the five-dose experiments (100 μM, 10 μM, 1 μM, 0.1 μM, and 0.01 μM) conducted on compounds **2c**, **2e**, **2f**, **2g**, **2h**, **2i**, and **2p** indicate that these compounds had significant and wide-ranging antitumor activity toward the cancer cell line panels that were evaluated (Table 2). Moreover, the IC_50_ of compounds **2e**, **2f**, **2h**, and **2p** were evaluated. Compound **2c** (R = 4-F) had significant efficacy against all cancer cell lines tested, with GI_50_ Value (growth inhibition 50%) ranging from 1.93 μM (against MDA-MB-468) to 16.7 μM (against MOLT-4). Compound **2e** (R = 3-Cl) exhibited significant efficacy against all selected cell lines, especially against HCT-116, LOX IMVI, and cell lines (IC_50_ 2.95, 2.88, 2.88 μM, respectively). Compared to **2e**, compound **2f** (R = 4-Cl) showed its highest inhibitory activity against different cell lines namely, CCRF-CEM, RPMI-8226, OVCAR-3, and MDA-MB-468 (IC_50_ 2.88, 2.40, 2.82, 2.51 μM, respectively). Compound **2g** (R = 4-Br) had significant efficacy against all cancer cell lines tested, with GI_50_ ranging from 1.79 μM (against MDA-MB-468) to 15.40 μM (against OVCAR-5). On the other hand, compound **2h** (R = 3-NO_2_) exhibited broad activity against the tested cell lines with remarkable inhibition against both U251 and LOX IMVI cell lines (IC_50_ 3.09 and 2.75 μM, respectively). Compound **2i** (R = 4-NO_2_) showed significant inhibition against all tested cancer cell lines, with GI_50_ ranging from 1.85 μM (against LOX IMVI) to 18.6 μM (against SK-OV-3). Finally, compound **2p** (R = 3,4,5-tri-OCH_3_) showed anticancer activity against CCRF-CEM, NCI-H460, SK-MEL-5, and OVCAR-8 (IC_50_ 3.55, 3.31, 3.31, and 3.55 μM, respectively).

From the data in Table 2, it can be concluded that an electron-withdrawing group on the phenyl ring is crucial for the anticancer activity. The *meta* position is optimal for anticancer activity (thiazole chalcones **2h** (R = 3-NO_2_) and **2e** (R = 3-Cl)). Shifting the substituent from *meta* to *para* slightly reduces the antitumor activity (thiazole derivatives **2f** (R = 4-Cl) and **2i** (R = 4-NO_2_)). On the other hand, the introduction of an electron donating group markedly decreases the anticancer activity (thiazole derivatives **2j** (R = 4-CH_3_), **2k** (R = 4-(CH_3_)_2_N), and **2l** (R = 4-OCH_3_)). Thiazole chalcone with trimethoxy phenyl moiety displayed remarkable anticancer activity.

#### 2.2.3. Effect of Compound **2e**, **2g**, **2h**, and **2p** on Tubulin Polymerization

**Figure 3:**
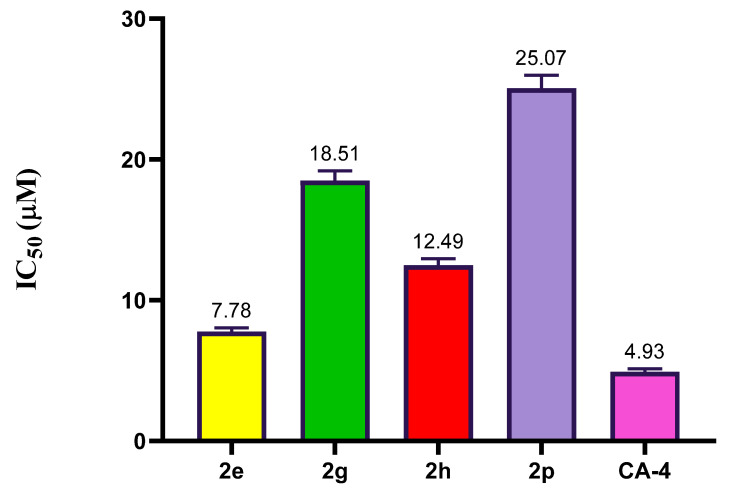
The inhibitory activity of thiazole derivatives **2e**, **2g**, **2h**, **2j**, **2q**, and **CA-4** on tubulin polymerization.

To investigate how thiazole derivatives exert their cytotoxic effects, particularly the most potent ones, the effects of compounds **2e**, **2g**, **2h**, and **2p** on tubulin polymerization were examined compared to the positive control **CA-4**. Both **CA-4** and **2e** demonstrated significant inhibition of tubulin polymerization, with IC_50_ values of 7.78 μM and 4.93 μM, respectively. Conversely, compounds **2g**, **2h**, and **2p** displayed moderate inhibition of tubulin polymerization compared to **CA-4**, with IC_50_ values of 18.51 μM, 12.49 μM, and 25.07 μM, respectively, as shown in Figure 3. These findings suggest that thiazole derivative **2e** may disrupt tubulin polymerization.

### 2.3. In Silico Studies

#### 2.3.1. Molecular Docking Studies

**Figure 4:**
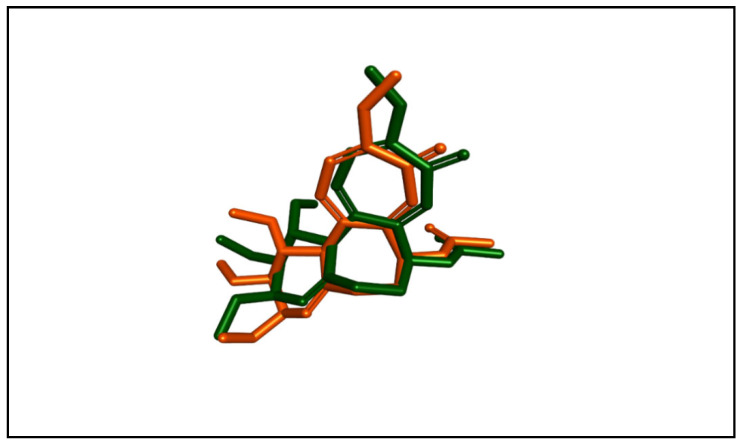
The superimposition of the redocked (orange) and co-crystallized (green) poses of colchicine (RMSD = 1.0090 Å).

Molecular docking studies were performed to assess the binding abilities and modes of the most potent compounds at the colchicine binding site of tubulin. These studies sought to compare the interactions of these compounds with the established CA-4 and to elucidate their binding mechanisms. Autodock vina was used for the docking simulations. A crystal structure of the tubulin–colchicine complex was used (PDB ID: 4O2B) [37]. To validate the molecular docking method, we performed a redocking procedure of colchicine into its binding site. Results indicated that the redocked colchicine exhibited an affinity of −8.7 kcal/mol. The redocked ligand had a root mean square deviation (RMSD) value of 1.0090 Å compared to the co-crystallized pose. It established most of the binding interactions exhibited by the native ligand. These results confirm the accuracy and reliability of our docking protocol for evaluating the binding interactions of the newly synthesized compounds. The superimposition of both the redocked and co-crystallized poses of colchicine is depicted in Figure 4.

**Figure 5:**
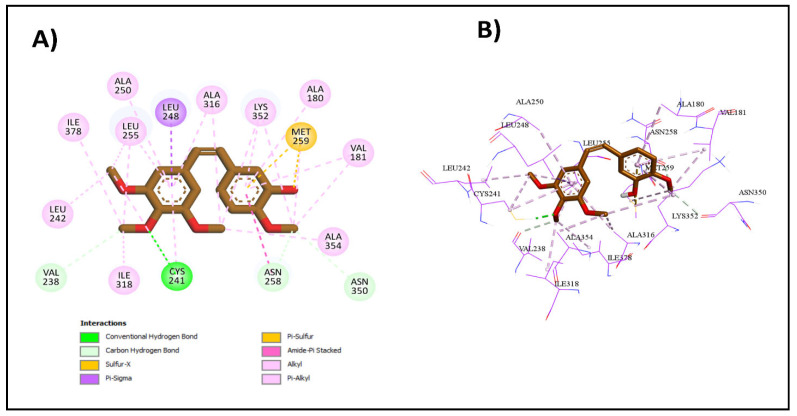
Interactions of CA-4 with colchicine binding site: (**A**) the 2D binding interactions; (**B**) the 3D binding interactions.

**Figure 6:**
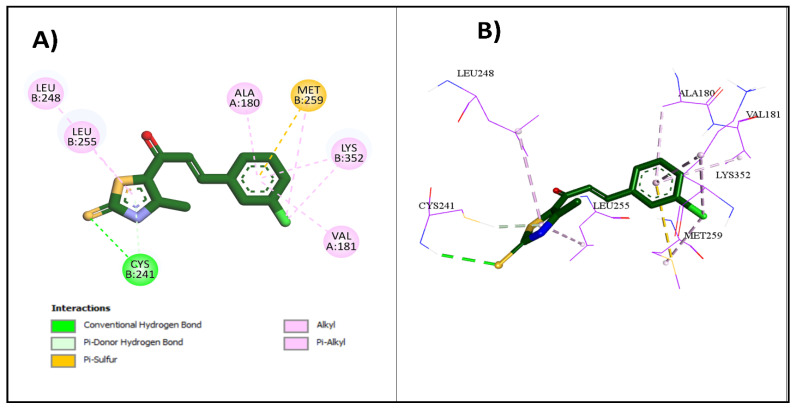
Interactions of **2e** with colchicine binding site: (**A**) the 2D binding interactions; (**B**) the 3D binding interactions.

**Figure 7:**
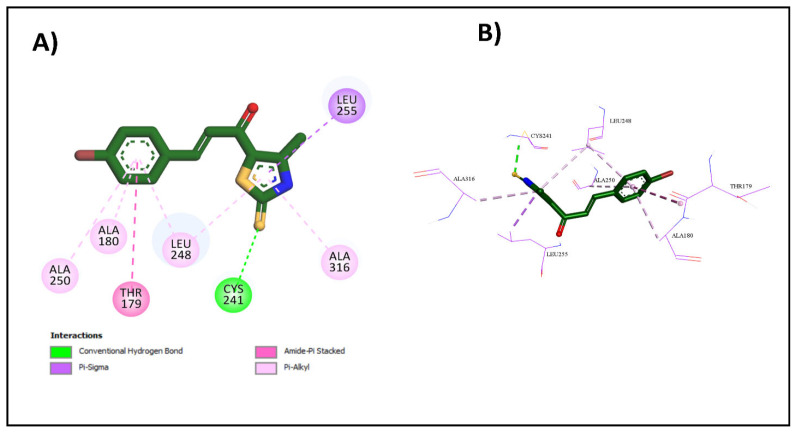
Interactions of **2g** with colchicine binding site: (**A**) the 2D binding interactions; (**B**) the 3D binding interactions.

**Figure 8:**
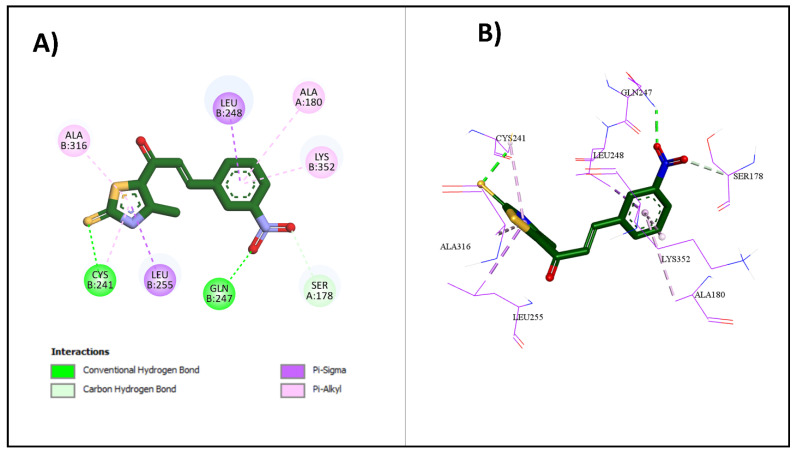
Interactions of **2h** with colchicine binding site: (**A**) the 2D binding interactions; (**B**) the 3D binding interactions.

Upon docking of the most potent compounds into the colchicine binding site, the binding affinities of the docked compounds **2e**, **2g**, and **2h** ranged from −7.3 to −8.9 kcal/mol, which was nearly comparable to the affinity of CA-4 at −9.2 kcal/mol (Table 3). Additionally, by examining the best docking poses for these compounds, they seemed to share a similar ligand–receptor interaction profile where they were able to establish key hydrogen bonding with Cys241 through its thiocarbonyl moiety, highlighted, which is pivotal in facilitating the tight binding of CA-4 and colchicine with β-tubulin as highlighted by Gracheva et al. (Figure 5) [38]. Additional non-classical hydrogen bonding between the thiazole ring and Cys241 in compounds **2e** and **2h** could explain their higher potency than compound **2g**. The thiazole ring also formed several hydrophobic interactions with amino acids Leu255, Ala316, and Leu248. The incorporation of the chalcone moiety also contributed to further anchoring these compounds into the pocket by forming more hydrophobic interactions through the phenyl ring with several amino acids, such as Ala180 and Lys352. Both the 2D and 3D diagrams of the best docking poses and their interactions are illustrated in Figure 6, Figure 7 and Figure 8. The docking results presented here align well with the in vitro findings, further substantiating the potential of these newly synthesized compounds as promising candidates for further investigation as tubulin polymerization inhibitors.

#### 2.3.2. Physicochemical and ADME Prediction

In the drug design journey, it is necessary to optimize the pharmacodynamics and pharmacokinetics of potential drug candidates in a parallel way. Therefore, we investigated the physicochemical and pharmacokinetic characteristics of the designed thiazole chalcones **2a**–**2p**; computational calculations were conducted using the SwissADME website to determine the physicochemical and ADME parameters. The Supplementary Materials section displays comprehensive results of the in silico studies (Tables S1–S5).

**Figure 9:**
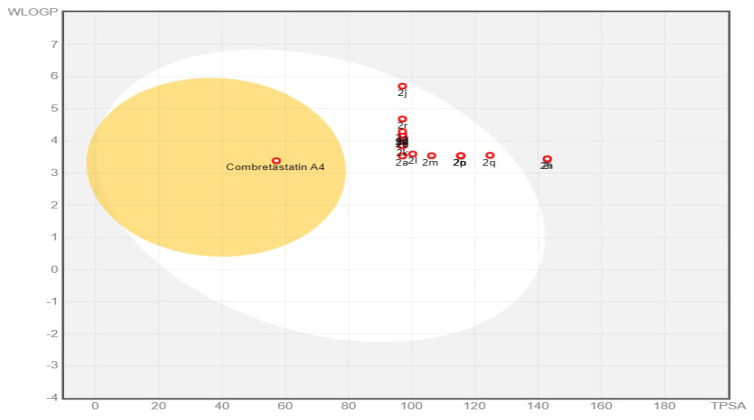
BOILED Egg plot of target compounds **2a**–**2p** and CA-4.

The BOILED Egg approach is a reliable model that precisely predicts the absorption of drug candidates in the gastrointestinal tract and their accessibility via BBB. It achieves this by estimating their lipophilicity (measured in WLOGP) against their polarity (measured in TPSA) (Figure 9). All designed compounds except **2h** and **2i** appeared in a white zone, indicating a significant gastrointestinal absorption level. This can be attributed to a balance between their lipophilicity (WLOGP 3.38–5.7) and their polarity (TPSA 97.00–142.82 Å^2^) (Supplementary Materials Tables S2 and S3). Compared to CA-4, all target compounds **2a**–**2p** are predicted not to cross the BBB, which confirms their favorable CNS safety profile. All designed compounds appeared as red points in the BOILED Egg plot, which means they are predicted not to be p-gp substrates, which enhances gastrointestinal absorption and overcomes one of the drug resistance mechanisms [39,40].

**Figure 10:**
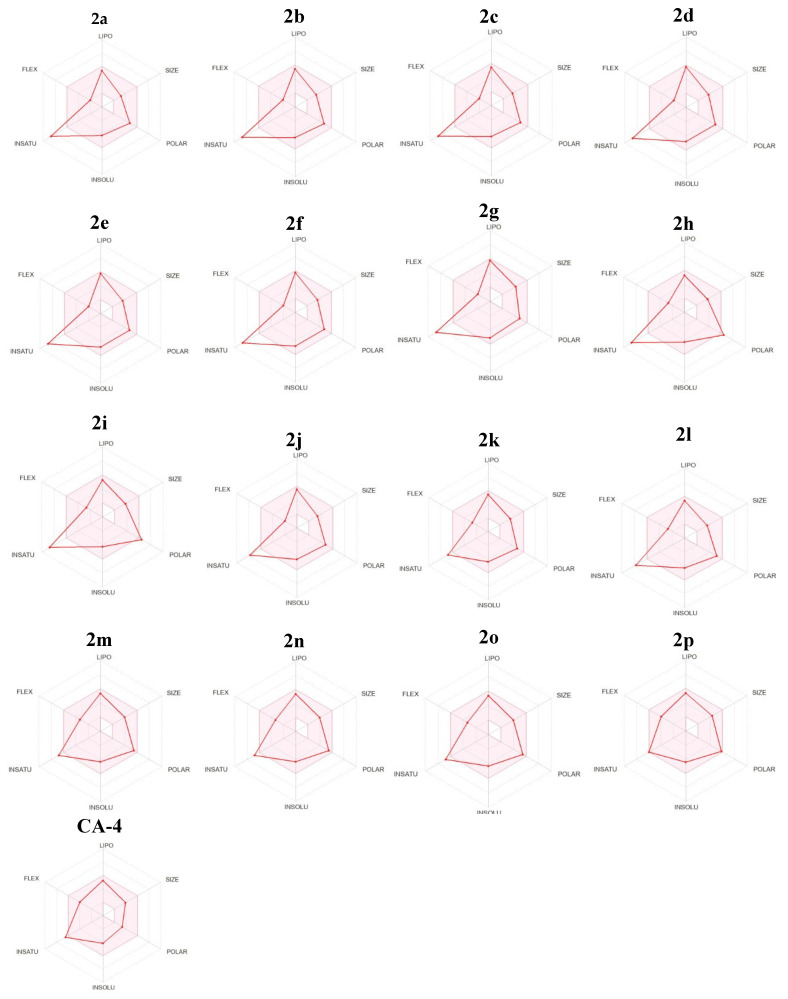
Rader model for target compounds **2a**–**2p** and Combretastatin A4.

Most target compounds demonstrate favorable anticipated physicochemical properties that make them suitable for oral bioavailability. The bioavailability radar can serve as a convenient means of representing this concept (Figure 10). In the bioavailability radar plot, the pink zone represents the best range for six physicochemical parameters: size, solubility, lipophilicity, polarity, saturation, and flexibility. These properties are considered optimal for achieving optimal oral bioavailability. The majority of the thiazole chalcones are concentrated in the pink region. However, there is a little deviation in the degree of saturation from the pink region due to the presence of less than 0.25 sp^3^ hybridized carbons [3].

It is worth noting that all target compounds **2a**–**2p** satisfy the drug-likeness criteria of Lipinski’s rule [41]. Additionally, all target compounds satisfy the Ghose filter [42]. Except for **2h** and **2i**, all target compounds satisfy the Veber rule [43] and the Egan filter [44]. Finally, all target compounds satisfy Muegge’s filter (Supplementary Materials Table S5) [45].

Based on the findings of the in silico ADME prediction studies, it can be inferred that the designed thiazole chalcones exhibit substantial cytotoxic and tubulin polymerization inhibitory properties, as well as favorable physicochemical, pharmacokinetic, and drug-likeness characteristics. These attributes make them suitable for further optimization as potential chemotherapeutic agents.

### 2.4. Structure Activity Relationship (SAR) Studies

**Figure 11:**
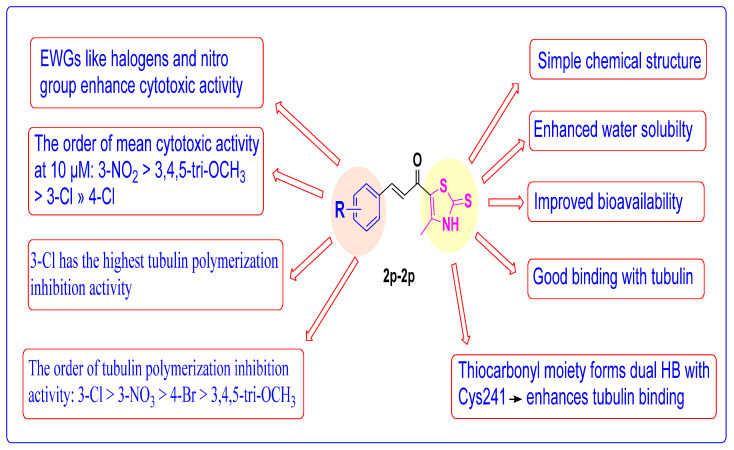
Structure–activity relationship of thiazole–chalcone derivatives.

SAR studies revealed that substituting the phenyl ring of thiazole chalcones **2a**–**2p** greatly impacts the potency against various cancer cell lines. SAR findings re-garding the anticancer activity, tubulin polymerization inhibitory activity, molecular docking, and ADME Studies are summarized in Figure 11.

## 3. Experimental Section

### 3.1. Chemistry

Thin-layer chromatography (TLC), using a Merck Grade-9385 precoated aluminum TLC plate with silica gel 60, measuring 5*20 cm, and having a thickness of 0.2 mm, was employed to monitor the progress of the chemical reaction. To detect the spots, the plates were exposed to ultraviolet (UV) light with a wavelength of 254 nm. The Stuart Electrothermal, Melting Point Apparatus was also utilized to determine the melting points without correcting the values. Furthermore, NMR spectra were obtained using a Bruker 400 MHz spectrometer operating at 100 MHz for ^13^C and 400 MHz for ^1^H. The solvent used was DMSO-*d*_6_, and tetramethylsilane served as the internal standard. In this study, the chemical shifts (δ) and coupling constants (J) were reported in parts per million (ppm) and hertz (Hz), respectively. The following abbreviations were employed to describe the diversity of NMR peaks: singlet (s), doublet (d), doublet of doublets (dd), triplet (t), quartet (q), multiplet (m), and broad signal (brs). Elemental analyses were performed using Shimadzu’s GC/MS-QP5050A instrument at the Regional Centre for Mycology and Biotechnology, Al-Azhar University, Cairo, Egypt. Low-resolution mass analyses were performed at Agilent Pharmaceuticals Inc. (Canada). The spectra were acquired using an Agilent 6400 LC/TQ spectrometer in the negative/positive mode of electrospray ionization (ESI). The intermediate **1** was prepared according to the reported method [36].

#### 3.1.1. General Procedures for the Synthesis of Derivatives **2a**–**2p**

An equimolar amount of thiazole derivative 1 (173 mg, 1 mmol) and the appropriate aromatic aldehyde (1 mmol) were dissolved in ethanol, and aqueous NaOH (140 mg, 3.5 mmol 60%) was added dropwise [24].The reaction mixture was stirred in an ice bath for 2 h, then at rt for 18–20 h. The reaction mixture was acidified by diluted acetic acid. The formed precipitate was filtered off and washed with distilled water, then recrystallized from ethanol.

##### (E)-1-(4-Methyl-2-Thioxo-2,3-Dihydrothiazol-5-yl)-3-Phenylprop-2-En-1-One **2a**

Yellow powder; 0.227 g, 87% yield; mp 245–247 °C; ^1^H NMR (400 MHz, DMSO-*d*_6_) δ 13.63 (1H, s, N*-H*), 7.79–7.80 (2H, m, Ar*-H*), 7.66 (1H, d, *J*_trans_ = 16 Hz, **=**C*H*), 7.45–7.47 (3H, m, Ar*-H*), 7.24 (1H, d, *J*_trans_ = 16 Hz, **=**C*H*), 2.57 (3H, s, C*H*_3_); ^13^C NMR (100 MHz, DMSO-*d*_6_) δ 189.2, 180.2, 147.4, 144.1, 134.7, 131.3, 129.5, 129.3, 124.3, 123.8, 14.9; ESI-MS (*m*/*z*): Calcd. 261.03, found 260.56 [M-H]^−^; Anal. Calcd. For C_13_H_11_NOS_2_: C, 59.74%; H, 4.24%; N, 5.36%. Found: C, 59.52%; H, 4.33%; N, 5.45%.

##### (E)-3-(3-Fluorophenyl)-1-(4-Methyl-2-Thioxo-2,3-Dihydrothiazol-5-yl)Prop-2-En-1-One **2b**

Yellow powder; 0.203 g, 73% yield; mp 234–236 °C; ^1^H NMR (400 MHz, DMSO-*d*_6_) δ 13.70 (1H, s, N*-H*), 7.73 (1H, d, *J* = 8 Hz, Ar*-H*), 7.66–7.62 (2H, m, Ar*-H* and **=**C*H*), 7.49 (1H, td, *J* = 8 Hz, Ar*-H*), 7.31–7.27 (2H, m, Ar*-H* and **=**C*H*), 2.57 (3H, s, C*H*_3_). ^13^C NMR (100 MHz, DMSO) δ 189.3, 180.1, 162.9 (C-3, d, ^1^*J*_CF*ipso*_ = 244.01 Hz), 147.8, 142.6, 137.2 (C-1, d, ^3^*J*_CF*meta*_ = 8.14 Hz), 131.4 (C-5, d, ^3^*J*_CF*meta*_ = 8.32 Hz), 125.8, 125.1, 124.2, 117.9 (C-2, d, ^2^*J*_CF*ortho*_ = 21.43 Hz), 115.3 (C-4, d, ^2^*J*_CF*ortho*_ = 21.86 Hz), 14.9; ESI-MS (*m*/*z*): Calcd. 279.02, found 278.58 [M-H]^−^; Anal. Calcd. For C_13_H_10_FNOS_2_: C, 55.90%; H, 3.61%; N, 5.01%. Found: C, 56.04%; H, 3.85%; N, 5.10%.

##### (E)-3-(4-Fluorophenyl)-1-(4-Methyl-2-Thioxo-2,3-Dihydrothiazol-5-yl)Prop-2-En-1-One **2c**

Yellow powder; 0.220 g, 79% yield; mp 262–264 °C; ^1^H NMR (400 MHz, DMSO-*d*_6_) 13.63 (1H, s, N*-H*), 7.89 (2H, dd, *J* = 4 Hz, Ar-*H*), 7.66 (1H, d, *J*_trans_ = 16 Hz, **=**C*H*), 7.29 (2H, t, *J_HF_* = 8 Hz, Ar-*H*), 7.19 (1H, d, *J*_trans_ = 16 Hz, **=**C*H*), δ 2.56 (3H, s, CH_3_); ^13^C NMR (100 MHz, DMSO-*d*_6_) δ 189.2, 180.2, 164.0 (C-4, d, ^1^*J*_CF*ipso*_ = 249.12 Hz), 147.4, 142.9, 131.7 (C-2 and C-6, d, ^3^*J*_CF*meta*_ = 8.81 Hz), 131.3, 124.2, 123.7, 116.5 (C-3 and C-5, d, ^2^*J*_CF*ortho*_ = 21.70 Hz), 14.9; ESI-MS (*m*/*z*): Calcd. 279.02, found 278.53 [M-H]^−^; Anal. Calcd. For C_13_H_10_FNOS_2_: C, 55.90%; H, 3.61%; N, 5.01%. Found: C, 56.03%; H, 3.51%; N, 5.12%.

##### (E)-3-(2-Chlorophenyl)-1-(4-Methyl-2-Thioxo-2,3-Dihydrothiazol-5-yl)Prop-2-En-1-One **2d**

Yellow powder; 0.192 g, 65% yield; mp 240–242 °C; ^1^H NMR (400 MHz, DMSO-*d*_6_) δ 13.67 (1H, s, N*-H*), 8.03 (1H, d, *J* = 8 Hz, Ar*-H*), 7.92 (1H, d, *J*_trans_ = 16 Hz, **=**C*H*), 7.56 (1H, d, *J* = 4 Hz, Ar*-H*), 7.50–7.42 (2H, m, Ar*-H*), 7.29 (1H, d, *J*_trans_ = 16 Hz, **=**C*H*), 2.58 (3H, s, C*H*_3_). ^13^C NMR (100 MHz, DMSO) δ 189.3, 179.8, 148.1, 139.3, 137.8, 134.8, 132.3, 131.3, 129.1, 127.5, 125.7, 123.9, 14.3; ESI-MS (*m*/*z*): Calcd. 294.99, found 294.51 [M-H]^−^; Anal. Calcd. For C_13_H_10_ClNOS_2_: C, 52.79%; H, 3.41%; N, 4.74%. Found: C, 52.91%; H, 3.37%; N, 4.58%.

##### (E)-3-(3-Chlorophenyl)-1-(4-Methyl-2-Thioxo-2,3-Dihydrothiazol-5-yl)Prop-2-En-1-One **2e**

Yellow powder; 0.251 g, 85% yield; mp 246–248 °C; ^1^H NMR (400 MHz, DMSO-*d*_6_) δ 13.69 (1H, s, N*-H*), 7.94 (1H, s, Ar*-H*), 7.76 (1H, d, *J* = 8 Hz, Ar*-H*), 7.62 (1H, d, *J*_trans_ = 16 Hz, **=**C*H*), 7.52–7.45 (2H, m, Ar*-H*), 7.30 (1H, d, *J*_trans_ = 12 Hz, **=**C*H*), 2.57 (3H, s, C*H*_3_); ^13^C NMR (100 MHz, DMSO-*d*_6_) δ 189.2, 180.1, 147.9, 142.4, 136.9, 134.3, 131.2, 130.9, 128.7, 128.1, 124.2, 124.2, 14.9; ESI-MS (*m*/*z*): Calcd. 294.99, found 294.50 [M-H]^−^; Anal. Calcd. For C_13_H_10_ClNOS_2_: C, 52.79%; H, 3.41%; N, 4.74%. Found: C, 53.01%; H, 3.48%; N, 4.62%.

##### (E)-3-(4-Chlorophenyl)-1-(4-Methyl-2-Thioxo-2,3-Dihydrothiazol-5-yl)Prop-2-En-1-One **2f**

Yellow powder; 0.260 g, 88% yield; mp 250–252 °C; ^1^H NMR (400 MHz, DMSO-*d*_6_) δ 13.67 (1H, s, N*-H*), 7.76 (2H, d, *J* = 8 Hz, Ar*-H*), 7.56 (1H, d, *J*_trans_ = 16 Hz, **=**C*H*), 7.43 (2H, d, *J* = 8 Hz, Ar*-H*), 7.17 (1H, d, *J*_trans_ = 16 Hz, **=**C*H*), 2.44 (3H, s, C*H*_3_). ^13^C NMR (100 MHz, DMSO) δ 189.2, 180.0, 147.8, 142.6, 135.8, 133.6, 131.0, 129.5, 124.4, 124.3, 39.9, 14.9; ESI-MS (*m*/*z*): Calcd. 294.99, found 294.61 [M-H]^−^; Anal. Calcd. For C_13_H_10_ClNOS_2_: C, 52.79%; H, 3.41%; N, 4.74%. Found: C, 52.85%; H, 3.64%; N, 4.81%.

##### (E)-3-(4-Bromophenyl)-1-(4-Methyl-2-Thioxo-2,3-Dihydrothiazol-5-yl)Prop-2-En-1-One **2g**

Yellow powder; 0.275 g, 80.82% yield; mp 264–266 °C; ^1^H NMR (400 MHz, DMSO-*d*_6_) δ 13.64 (1H, s, N*-H*), 7,76 (2H, d, *J* = 8 Hz, Ar*-H*), 7.65 (2H, d, *J* = 8 Hz, Ar*-H*), 7.62 (1H, d, *J*_trans_ = 16 Hz, =C*H*), 7.26 (1H, d, *J*_trans_ = 16 Hz, **=**C*H*), 2.57 (3H, s, CH_3_); ^13^C NMR (100 MHz, DMSO) 189.2, 180.0, 147.7, 143.4, 133.3, 131.5, 130.3, 125.4, 124.7, 123.8, 14.3; ESI-MS (*m*/*z*): Calcd. 338.94, found 340.50 [M+2-H]^−^; Anal. Calcd. For C_13_H_10_BrNOS_2_: C, 45.89%; H, 2.96%; N, 4.12%. Found: C, 45.97%; H, 2.78%; N, 4.25%.

##### (E)-1-(4-Methyl-2-Thioxo-2,3-Dihydrothiazol-5-yl)-3-(3-Nitrophenyl)Prop-2-En-1-One **2h**

Yellow powder; 0.226 g, 74% yield; mp 260–262 °C; ^1^H NMR (400 MHz, DMSO-*d*_6_) δ 13.63 (1H, s, N*-H*), 8.57 (1H, s, Ar*-H*), 8.19 (2H, d, *J* = 8 Hz, Ar*-H*), 7.71–7.64 (2H, m, Ar*-H* and **=**C*H*), 7.35 (1H, d, *J*_trans_ = 16 Hz, **=**C*H*), 2.43 (3H, s, C*H*_3_); ^13^C NMR (100 MHz, DMSO-*d*_6_) δ 189.3, 180.0, 148.8, 148.2, 141.5, 136.5, 135.1, 130.8, 126.5, 125.4, 123.9, 123.8, 14.9; ESI-MS (*m*/*z*): Calcd. 306.01, found 305.51 [M-H]^−^; Anal. calcd. for C_13_H_10_N_2_O_3_S_2_: C, 50.97%; H, 3.29%; N, 9.14%. Found: C, 50.93%; H, 3.14%; N, 8.97%.

##### (E)-1-(4-Methyl-2-Thioxo-2,3-Dihydrothiazol-5-yl)-3-(4-Nitrophenyl)Prop-2-En-1-One **2i**

Yellow powder; 0.196 g, 64% yield; mp 255–257 °C; ^1^H NMR (400 MHz, DMSO-*d*_6_) δ 13.59 (1H, s, N*-H*), 8.27 (2H, d, *J* = 8 Hz, Ar-*H*), 8.07 (2H, d, *J* = 8 Hz, Ar*-H*), 7.72 (1H, d, *J*_trans_ = 16 Hz, =C*H*), 7.41 (1H, d, *J_trans_* = 16 Hz, **=**C*H*), 2.58 (3H, s, C*H*_3_); ^13^C NMR (100 MHz, DMSO) δ 189.4, 179.9, 148.7, 148.4, 141.1, 130.3, 128.0, 127.8, 124.4, 124.01, 15.1; ESI-MS (*m*/*z*): Calcd. 306.01, found 305.52 [M-H]^−^; Anal. calcd. for C_13_H_10_N_2_O_3_S_2_: C, 50.97%; H, 3.29%; N, 9.14%. Found: C, 51.15%; H, 3.13%; N, 9.38%

##### (E)-1-(4-Methyl-2-Thioxo-2,3-Dihydrothiazol-5-yl)-3-(p-Tolyl)Prop-2-En-1-One **2j**

Yellow powder; 0.247 g, 90% yield; mp 241–243 °C; ^1^H-NMR (400 MHz, DMSO-*d*_6_) δ 13.60 (1H, s, N*-H*), 7.68 (2H, d, *J* = 8 Hz, Ar*-H*), 7.63 (1H, d, *J*_trans_ = 16 Hz, =C*H*), 7.27 (2H, d, *J* = 8 Hz, Ar*-H*), 7.16 (1H, d, *J*_trans_ = 16 Hz, **=**C*H*), 2.56 (3H, s, C*H*_3_), 2.35 (3H, s, Ar-C*H*_3_); ^13^C NMR (100 MHz, DMSO) δ 189.1, 180.2, 147.1, 144.9, 141.5, 131.9, 130.9, 128.5, 124.3, 121.9, 20.9, 14.9; ESI-MS (*m*/*z*): Calcd. 275.04, found 274.59 [M-H]^−^; Anal. Calcd. For C_14_H_13_NOS_2_: C, 61.06%; H, 4.76%; N, 5.09%. Found: C, 61.14%; H, 4.67%; N, 5.24%.

##### (E)-3-(4-(Dimethylamino)Phenyl)-1-(4-Methyl-2-Thioxo-2,3-Dihydrothiazol-5-yl)Prop-2-En-1-One **2k**

Pale Red powder; 0.222 g, 73% yield; mp 249–251 °C; ^1^H NMR (400 MHz, DMSO-*d*_6_) δ 13.61 (1H, s, N*-H*), 7.62–7.57 (3H, m, Ar-*H* and =C*H*), 6.91 (1H, d, *J*_trans_ = 12 Hz, =C*H*), 6.74 (2H, d, *J* = 8 Hz, Ar-*H*), 3.01(6H, s, N(C*H*_3_)*_2_*), 2.54 (3H, s, C*H*_3_); ^13^C NMR (100 MHz, DMSO) δ 189.4, 179.9, 152.8, 148.4, 145.5, 131.3, 129.4, 121.9, 117.7, 112.3, 40.7, 14.9; ESI-MS (*m*/*z*): Calcd. 304.07, found 303.7 [M-H]^−^; Anal. Calcd. For C_15_H_16_N_2_OS_2_: C, 59.18%; H, 5.30%; N, 9.20%. Found: C, 59.02%; H, 5.48%; N, 9.36%.

##### (E)-3-(4-Methoxyphenyl)-1-(4-Methyl-2-Thioxo-2,3-Dihydrothiazol-5-yl)Prop-2-En-1-One **2l**

Yellow powder; 0.227 g, 78% yield; mp 246–248 °C; ^1^H NMR (400 MHz, DMSO-*d*_6_) δ 13.59 (1H, s, N*-H*), 7.76 (2H, d, *J* = 8 Hz, Ar-*H*), 7.63 (1H, d, *J_t_*_rans_ = 12 Hz, =C*H*), 7.08 (1H, d, *J_t_*_rans_ = 16 Hz, **=**C*H*), 7.01 (2H, d, *J* = 8 Hz, Ar-*H*), 3.83 (3H, s, OC*H_3_*), 2.56 (3H, s, C*H*_3_); ^13^C NMR (100 MHz, DMSO) δ ^13^C NMR (100 MHz, DMSO) δ 189.0, 180.1, 162.1, 146.9, 143.4, 130.4, 127.2, 124.4, 120.4, 114.2, 55.2, 14.1; ESI-MS (*m*/*z*): Calcd. 291.04, found 290.57 [M+H]^+^; Anal. Calcd. For C_14_H_13_NO_2_S_2_: C, 57.71%; H, 4.50%; N, 4.81%. Found: C, 57.83%; H, 4.65%; N, 4.67%.

##### (E)-3-(2,3-Dimethoxyphenyl)-1-(4-Methyl-2-Thioxo-2,3-Dihydrothiazol-5-yl)Prop-2-En-1-One **2m**

Yellow powder; 0.215 g, 67% yield; mp 239–240 °C; ^1^H NMR (400 MHz, DMSO-*d*_6_) δ 13.67 (1H, s, N*-H*), 7.84 (1H, d, *J*_trans_ = 16 Hz, =C*H*), 7.43 (1H, s, Ar*-H*), 7.27 (1H, d, *J_trans_* = 16 Hz, **=**C*H*), 7.17–7.15 (2H, m, Ar*-H*), 3.84 (3H, s, OC*H*_3_), 3.79 (3H, s, OC*H*_3_), 2.57 (3H, s, C*H*_3_); ^13^C NMR (100 MHz, DMSO) δ 189.2, 180.2, 153.4, 147.6, 139.3, 137.6, 128.1, 124.3, 124.0, 121.1, 119.1, 115.1, 57.1, 55.2, 14.2; ESI-MS (*m*/*z*): Calcd. 321.05, found 320.60 [M-H]^−^; Anal. Calcd. For C_15_H_15_NO_3_S_2_: C, 56.05%; H, 4.70%; N, 4.36%. Found: C, 56.14%; H, 4.57%; N, 4.53%.

##### (E)-3-(2,4-Dimethoxyphenyl)-1-(4-Methyl-2-Thioxo-2,3-Dihydrothiazol-5-yl)Prop-2-En-1-One **2n**

Yellow powder; 0.221 g, 69% yield; mp 255–257 °C; ^1^H NMR (400 MHz, DMSO-*d*_6_) δ 13.56 (1H, s, N*-H*), 7.76 (1H, d, *J*_trans_ = 16 Hz, =C*H*), 7.67 (1H, d, *J* = 12 Hz, Ar*-H*), 7.13 (1H, d, *J*_trans_ = 16 Hz, **=**C*H*), 7.60–7.56 (2H, m, Ar*-H*), 3.86 (3H, s, OC*H*_3_), 3.80 (3H, s, OC*H*_3_), 2.46 (3H, s, C*H*_3_); 13C NMR (100 MHz, DMSO) δ ^13^C NMR (100 MHz, DMSO) δ 188.9, 180.2, 163.8, 160.8, 146.7, 139.6, 131.9, 124.8, 121.0, 115.9, 106.9, 98.8, 56.4, 56.1, 40.4, 14.9; ESI-MS (*m*/*z*): Calcd. 321.05, found 320.63 [M-H]^−^; Anal. Calcd. For C_15_H_15_NO_3_S_2_: C, 56.05%; H, 4.70%; N, 4.36%. Found: C, 55.90%; H, 4.69%; N, 4.54%.

##### (E)-3-(3,4-Dimethoxyphenyl)-1-(4-Methyl-2-Thioxo-2,3-Dihydrothiazol-5-yl)Prop-2-En-1-One **2o**

Yellow powder; 0.228 g, 71% yield; mp 245–247 °C; ^1^H NMR (400 MHz, DMSO-*d*_6_) δ 13.70 (1H, s, N*-H*), 7.61 (1H, d, *J*_trans_ = 16 Hz, =C*H*), 7.40–7.34 (2H, m, Ar*-H*), 7.10 (1H, d, *J*_trans_ = 16 Hz, **=**C*H*), 7.01 (1H, d, *J* = 12 Hz, Ar*-H*), 3.83 (3H, s, OC*H*_3_), 3.81 (3H, s, OC*H*_3_), 2.55 (3H, s, C*H*_3_) ^13^C NMR (100 MHz, DMSO) δ 188.9, 180.2, 152.2, 149.6, 147.0, 143.8, 127.5, 124.2, 120.7, 117.3, 113.2, 111.5, 56.9, 55.4, 14.2. ESI-MS (*m*/*z*): Calcd. 321.05, found 320.61 [M+H]^+^; Anal. Calcd. For C_15_H_15_NO_3_S_2_: C, 56.05%; H, 4.70%; N, 4.36%. Found: C, 55.91%; H, 4.81%; N, 4.56%.

##### (E)-1-(4-Methyl-2-Thioxo-2,3-Dihydrothiazol-5-yl)-3-(3,4,5-Trimethoxyphenyl)Prop-2-En-1-One **2p**

Yellow powder: 0.312 g, 89% yield; mp 248–250 °C; ^1^H NMR (400 MHz, DMSO-*d*_6_) δ 13.63 (1H, s, N-*H*), 7.60 (1H, d, *J*_trans_ = 16 Hz, **=**C*H*), 7.13–7.18 (3H, m, 2Ar*-H* and **=**C*H*), 3.85(6H, s, 2OC*H*_3_), 3.72(3H, s, OC*H*_3_), 2.56 (3H, s, C*H*_3_). ^13^C NMR (100 MHz, DMSO) δ 189.1, 180.4, 153.6, 144.6, 140.7, 130.2, 124.0, 123.3, 107.5, 107.2, 60.6, 56.7, 14.9; ESI-MS (*m*/*z*): Calcd. 351.06, found 350.80 [M-H]^−^; Anal. calcd. for C_16_H_17_NO_4_S_2_: C, 54.68%; H, 4.88%; N, 3.99%. Found: C, 54.61%; H, 4.77%; N, 3.90%.

### 3.2. Biological Evaluation and In Silico Studies Methodology

#### 3.2.1. Screening of the Anticancer Activity against a Panel of 60 Cell Lines

The methodology of the NCI anticancer screening has been described in detail elsewhere (http://www.dtp.nci.nih.gov) [2]. For detailed information, see Appendix A in the Supplementary Materials.

#### 3.2.2. In Vitro Tubulin Polymerization Inhibition Assay

In vitro determination of the interaction of thiazole derivatives **2e**, **2g**, **2h**, **2p**, and the reference drug CA-4 with the microtubule system was carried out according to the reported protocol [32]. See Appendix A in the Supplementary Materials.

#### 3.2.3. In Silico Studies

##### Molecular Docking

Autodock vina v1.2.0 was used for molecular docking and the best docking poses were visualized using Discovery Studio Visualizer v24.1.0.23298 [46]. Detailed information is provided in Appendix A in the Supplementary Materials.

##### In Silico Physicochemical and Pharmacokinetic Properties

The physicochemical and pharmacokinetic parameters for **2a**–**2p** were predicted using the SwissADME tool (http://www.swissadme.ch/index.php) [40]. See Appendix A in the Supplementary Materials.

## 4. Conclusions

In summary, our research has led to the synthesis and evaluation of a series of novel thiazole-privileged chalcones as tubulin polymerization inhibitors with potential anticancer activities. Thiazole derivatives **2c**, **2e**, **2f**, **2g**, **2h**, **2i**, and **2p** revealed broad in vitro cytotoxic activity against various cancer cells, specifically leukemia, colon, renal, and breast cancer cells. Thiazole derivative **2e** displayed remarkable antitumor activity against UO-31, SNB-75, LOX IMVI, HCT-116, SW-620, U251, RXF 393, and KM12. Also, compound 2e has demonstrated the ability to inhibit tubulin polymerization in vitro. The use of the thiazole ring, instead of the phenyl ring of classical chalcone, has significantly enhanced both pharmacodynamics and pharmacokinetics. Incorporation of the thiazole moiety in these compounds improves their aqueous solubility, bioavailability, and potentiates binding interaction with the colchicine binding site by forming a dual hydrogen bond with Cys241. These findings underscore the potential of thiazole-privileged chalcones **2a**–**2p** as a promising new class of tubulin-inhibiting molecules for further investigation as a potential anti-cancer therapeutics.

## Figures and Tables

**Table 1:** Illustration of the in vitro screening results of the anticancer activity of thiazole derivatives **2a**–**2p** at a dose of 10 μM.

Cell Line Panel/Cell Line Name		Growth Percentage of Thiazole Chalcones 2a–2p															
		2a	2b	2c	2d	2e	2f	2g	2h	2i	2j	2k	2l	2m	2n	2o	2p
**Leukemia**	**CCRF-CEM**	37.22	15.42	−0.21	46.79	15.19	3.18	−11.02	13.78	−7.25	24.6	68.52	58.98	12.41	53.72	15.53	4.92
	**HL-60(TB)**	49.59	12.04	12.98	53.03	25.32	−0.98	13.16	5.25	−5.39	61	87.05	58.48	12.74	55.84	35.6	3.95
	**K-562**	50.96	22.81	20.43	47.41	16.76	7.94	13.64	13.77	12.28	34.16	47.03	56.62	27.51	48.87	32.43	8.21
	**MOLT-4**	74.19	43.64	58.71	84.22	19.78	10.18	30.17	13.31	14.2	68.76	62.1	81.55	36.78	72.27	73.82	9.11
	**RPMI-8226**	14.13	15.24	2.32	28.65	10.37	13.82	1.4	9.39	−7.9	18.86	41.48	19.63	9.09	35.64	18.58	5.31
	**SR**	32.39	6.82	18.33	14.1	3.42	ND	8.46	−10.99	−4.81	45.55	57.41	43.69	5.15	ND	32.51	ND
**NSCLC**	**A549/ATCC**	82.31	76.38	55.96	87.57	26.49	61.14	64.07	19.51	75.71	74.27	68.06	82.86	43.09	90	64.57	26.57
	**EKVX**	73.11	68.61	51.04	35.62	34.72	40.96	62.9	0.65	74.66	55.52	67.01	84.75	54.52	56.24	54.16	23.4
	**HOP-62**	ND	34.77	ND	91.65	10.45	−2.73	11.94	0.68	21.63	54.1	86.25	ND	91.75	88.82	34.07	4.61
	**HOP-92**	89.61	ND	45.42	ND	ND	34.78	67.12	ND	57.63	98.01	118.71	100.67	61.54	103.21	83.73	16.45
	**NCI-H226**	96.48	85.65	67.1	89.61	32.73	20.49	54.51	−41.9	91.38	93.76	88.28	93.62	90.78	79.73	80.5	16.43
	**NCI-H23**	77.52	71.09	62.66	91.77	18	36.43	65.01	−72.77	49.2	68.54	71.13	81.76	86.47	72.87	65.13	7.27
	**NCI-H322M**	66.51	84.46	63.98	106.22	50.15	ND	44.13	28.92	50.72	62.15	81.14	92.4	62.76	ND	63.43	ND
	**NCI-H460**	56.7	43.22	39.31	82.17	12.05	82.56	39.06	−30.68	11.33	53.09	85.67	85.62	40.11	89.77	40.01	−1.34
	**NCI-H522**	71.74	63.86	44.19	90.13	21.99	13.54	21.1	−58.81	33	60.24	58.91	61.35	32.87	73.46	56.8	16.3
**Colon Cancer**	**COLO 205**	ND	94.51	ND	117.87	70.71	78.15	89.96	−24.88	91.53	121.66	115.46	ND	102.52	122.75	115.96	81.66
	**HCC-2998**	91.33	58.22	27.06	132.42	13.88	−21.28	3.23	−86.72	−11.87	77.15	95.3	100.59	79.76	80.73	41.71	19.01
	**HCT-116**	49.38	21.2	23.71	44.58	3.28	6.98	16.57	−6.25	−47.76	46.74	72.22	75.95	46.54	62.3	34.55	6.81
	**HCT-15**	35	19.06	14.71	57.4	−22.01	−19.78	5.48	−36.42	−62.1	38.58	64.44	50.75	32.68	45.78	31.33	−3.26
	**HT29**	99.67	47.06	73.33	122.14	29.92	31.17	76.26	20.35	62.83	98.71	94.03	96.79	ND	114.99	97.72	40.09
	**KM12**	29.04	11.91	6.85	63.81	−30.72	6.77	1.17	−78.4	−72.47	29.81	62.65	42.17	25.26	73.4	42.25	9.52
	**SW-620**	69.11	20.94	13.76	82.04	8.6	8.02	5.7	−37.8	−44.79	35.87	89.86	75.28	47.89	84.93	34.92	6.92
**CNS Cancer**	**SF-268**	69.81	82.33	58.62	80.38	51.84	67.94	46.1	27.46	47.36	86.89	99.28	81.05	54.04	89.73	67.22	18.5
	**SF-295**	88.86	38.42	60.61	82.55	41.84	42.62	90.37	4.14	86.08	83.38	79.76	83.49	26.31	72.5	66.5	42.28
	**SF-539**	41.72	6.17	−4.61	93.59	−10.65	−30.44	−25.34	−85.88	−25.22	42.96	88.7	69.82	22.73	87.29	29.82	−13.9
	**SNB-19**	66.75	44.03	33.66	85.22	ND	15.84	32.68	10.38	41.18	65.54	71.2	75.59	37.21	77.37	61.91	19.56
	**SNB-75**	92.71	ND	36.49	ND	8.74	ND	2.52	ND	35.98	78.06	96	98.01	50.41	ND	58.12	ND
	**U251**	56.91	17.63	14.09	63.31	12.07	11.35	15.53	−75.99	13.65	60.37	67.11	62.29	20.33	68.6	33.72	14.98
**Melanoma**	**LOX IMVI**	31.05	13.83	8.91	57.46	−26.09	−48.15	−5.17	−98.99	−86.21	31.22	60.24	44.25	21.68	57.81	26.92	17.13
	**MALME-3M**	87.78	101.24	85.79	104.51	86.93	ND	72.94	−42.94	75.16	84.65	79.42	90.04	97.13	ND	89.57	ND
	**M14**	77.06	78.45	49.58	82.29	40.25	34.39	74.48	1.86	69.52	83.02	83.18	87.51	53.99	75.84	82.23	15.43
	**MDA-MB-435**	50.1	35.94	4.67	80.75	17.26	22.51	8.26	−36.95	3.76	39.01	58.35	63.89	51.86	84.73	38.89	10.98
	**SK-MEL-2**	68.19	81.22	56.59	84.85	32.1	58.38	62.33	16.88	49.55	89.83	88.89	80.29	94.22	64.72	68.6	34.94
	**SK-MEL-28**	85.25	93.67	50.57	101.08	44.69	45.71	64.25	−47.02	23.87	92.85	92.66	101.91	70	92.8	83.19	24.24
	**SK-MEL-5**	68.82	62.16	42.56	68.46	16.05	27.99	48.84	−68.8	53.78	56.13	51.26	55.87	23.46	37.84	63.12	−64.57
	**UACC-257**	76.15	83.72	64.09	87.25	52.71	66.8	69.1	9.4	63.26	80.08	64.13	67.97	35.39	84.44	71.4	42.08
	**UACC-62**	65.26	58.32	47.27	72.88	29.97	33.88	46.84	−56.71	47.53	59.43	58.71	71.85	45.46	65.16	63.85	−3.11
**Ovarian Cancer**	**IGROV1**	81.82	32.3	51.24	91.91	33.98	ND	37.11	10.16	16.06	83.42	82.27	89.07	73.86	ND	69.47	ND
	**OVCAR-3**	42.69	−1.17	−10.94	77.36	8.26	−24.25	−12.3	−79.48	−6	14.99	82.2	66.86	24.27	91.16	3.22	−25.23
	**OVCAR-4**	76.39	79.55	48.43	90.32	31.68	76.31	49.51	13.05	49.16	59.22	51.23	72.33	62.2	52.05	47.63	15.6
	**OVCAR-5**	128.73	88.15	119.09	116.44	36.37	50.81	115.99	−37.4	109.56	138.42	142.09	144.69	81.68	120.39	131.45	6.56
	**OVCAR-8**	65.76	60.46	−13.63	85.43	10.39	4.96	−34.08	13.44	7.06	17.26	83.37	76.93	44.61	81.51	34.3	4.32
	**NCI/ADR-RES**	64.78	10.81	−10.78	88.8	−5.11	−8.39	−13.89	8.19	−4.45	31	56.92	76.38	30.95	48.05	26.85	0.58
	**SK-OV-3**	ND	110.63	ND	118.47	59.91	60.83	96.66	44.61	107.77	102.36	92.59	ND	85.62	100.52	76.93	31.19
**Renal Cancer**	**786-0**	68.1	46.91	37.27	93.1	13.35	16.59	25.03	−10.85	25.64	70.71	91.67	78.11	ND	85.94	60	21.09
	**A498**	105.99	106.3	115	116	98.01	105.85	108.9	98.87	104.64	130.77	129.94	120.21	108.77	100.71	125.58	96.5
	**ACHN**	71.77	55.96	47.17	93.42	22.9	34.61	55.01	−58.35	3.88	71.22	75.67	85.64	52.53	76.96	64.88	19.58
	**CAKI-1**	52.53	37.69	31.6	68.49	23.85	53.21	32.76	−56.46	31.07	43.41	68.64	59.04	41.93	82.85	42.04	28.19
	**RXF 393**	46.35	23.17	23.24	93.84	10.66	9.78	15.96	−91.92	−4.35	55.42	90.79	77.53	52.45	68.86	48.08	30.28
	**SN12C**	66.07	32.09	24.05	80.04	12	4.62	19.22	−25.64	18.63	62.33	75.73	74.87	44.99	80.95	45.37	7.3
	**TK-10**	80.33	ND	40.37	ND	ND	40.37	37.82	ND	12.9	79.78	86.81	80.89	64.47	94.1	75.43	52.99
	**UO-31**	39.33	44.34	28.13	73.69	34.33	30.44	22.38	−89.63	−41.32	46.79	48.26	49	53.74	ND	49.17	ND
**PC**	**PC-3**	77.6	54.71	43.41	71.78	31.83	ND	48.79	8.43	51.28	74.84	71.73	82.15	41.63	75.69	68.88	35.71
	**DU-145**	27.95	12.86	20.32	75.95	16.1	7.29	23.9	8.55	21.39	54.64	92.79	58.53	41.98	86.87	48.1	12.86
**Prostate Cancer**	**MCF7**	19.79	7.97	9.48	28.93	7.9	4.86	10.52	−45.42	5.51	28.33	59.99	30.84	16.65	45.98	28.86	9.35
	**MDA-MB-231/ATCC**	70.05	67.38	29.72	101.38	−0.43	39.29	9.49	−17.58	10.75	71.69	90.28	89.32	68.26	98.69	58.36	2.51
	**HS 578T**	90.12	79.81	95.29	92.76	47.21	47.98	74.6	24.47	68.11	101.32	118	96.5	49.25	89.49	84.71	3.94
	**BT-549**	68.85	65.65	23.21	76.82	2.28	−14.41	19.5	−14.03	20.09	56.13	82.22	75.04	70.95	59.75	18.94	−11.78
	**T-47D**	ND	0.69	ND	47.34	4.54	1.38	29.03	−56.63	44.32	38.31	32.35	ND	34.48	30.94	44.15	−1.59
	**MDA-MB-468**	20.79	−22.69	−4.51	30.83	−7.12	−21.19	−4.75	−82.86	−18.95	11.77	35.38	15.11	41.07	19.19	−0.42	3.79
**Mean growth percentage**		64.97	47.5	36.74	79.42	22.13	23.72	34.25	−21.75	25.23	63.31	77.71	74.58	49.88	75.09	55.11	14.89

ND = not determined.

**Table 2:** Five dose results of compounds **2c**, **2e**, **2f**, **2g**, **2h**, **2i**, **2p**, and **CA-4** in μM.

Cell Line Panel/Cell Line NAME		2c			2e				2f				2g			2h				2i			2p				CA-4		
		GI50	LC50	TGI	GI50	IC50	LC50	TGI	GI50	IC50	LC50	TGI	GI50	LC50	TGI	GI50	IC50	LC50	TGI	GI50	LC50	TGI	GI50	IC50	LC50	TGI	GI50	LC50	TGI
**Leukemia**	**CCRF-CEM**	3.68	>100	>100	2.45	3.72	>100	7.94	2.88	3.98	>100	11.22	3.06	>100	61.5	2.14	3.39	>100	6.61	4.56	>100	>100	2.45	3.55	>100	8.71	0.10	89.95	5.43
	**HL-60(TB)**	2.83	>100	32.2	2.24	4.27	>100	7.08	3.55	5.89	>100	38.02	1.94	>100	9.92	1.95	3.31	>100	5.75	3.05	>100	16.9	2.57	3.89	>100	11.22	0.03	59.02	0.06
	**K-562**	3.25	>100	48.8	3.39	4.27	>100	>100	3.89	4.68	>100	>100	2.73	>100	>100	3.09	3.72	>100	>100	3.5	>100	>100	3.02	3.72	>100	>100	0.03	>100	2.09
	**MOLT-4**	16.7	>100	64.3	2.82	4.27	>100	>100	14.45	24.55	>100	79.43	3.57	>100	36.6	3.47	5.01	>100	30.2	3.79	>100	>100	2.88	4.17	>100	22.91	0.10	78.34	3.72
	**RPMI-8226**	2.85	>100	>100	2.04	3.72	>100	5.5	2.4	4.68	>100	8.32	2.29	>100	>100	2.04	3.72	>100	6.31	3.55	>100	>100	2.19	4.47	>100	9.12	0.15	96.61	6.50
	**SR**	3.19	>100	>100	2.63	3.89	>100	>100	3.55	5.37	>100	56.23	2.83	>100	23.2	2.75	3.89	>100	>100	2.86	>100	>100	2.34	3.8	>100	16.22	0.11	>100	75.34
**NSCLC**	**A549/ATCC**	12.4	57.7	26.8	4.79	7.41	>100	47.86	10.72	16.22	52.48	23.99	11.7	55.3	25.4	3.24	4.47	54.95	12.02	17	69	34.2	3.55	5.01	44.67	13.8	0.07	96.38	76.56
	**EKVX**	12.8	64.6	28.7	10.47	23.99	95.5	31.62	12.88	23.99	52.48	26.3	10.6	51	23.3	5.75	15.14	46.77	19.5	16.4	58.1	30.9	4.07	10.96	>100	22.91	0.36	>100	82.04
	**HOP-62**	3.83	48.7	16.2	2.57	4.68	74.13	6.31	5.37	12.02	72.44	22.91	3.94	61.2	14	3.98	6.76	40.74	12.3	6.43	67.7	23.2	3.16	5.37	>100	16.22	0.18	89.13	2.53
	**HOP-92**	3.35	56	15.8	3.55	79.43	>100	30.9	11.48	30.2	63.1	26.92	6.44	58.8	22.5	3.89	22.39	44.67	16.6	12.2	64.4	28	2.95	44.67	>100	19.95	0.22	>100	36.31
	**NCI-H226**	5.27	>100	27.6	4.37	29.51	>100	38.9	13.8	36.31	>100	38.02	4.13	>100	25.4	11.22	33.11	>100	38.02	15.6	>100	42.1	3.63	32.36	>100	36.31	0.67	96.16	48.98
	**NCI-H23**	10.6	49.9	23	2.09	3.63	12.88	4.68	14.79	26.92	58.88	29.51	8.23	48	21.3	5.37	10	45.71	18.2	12.2	52.6	25.3	2.95	5.25	44.67	10.47	0.02	96.16	0.40
	**NCI-H322M**	4.1	44.8	17.9	6.03	16.22	85.11	26.3	10.23	19.95	51.29	22.91	3.76	41.3	15.2	8.71	17.38	50.12	21.88	11	54.6	24.5	4.57	14.13	51.29	18.62	0.07	>100	74.82
	**NCI-H460**	15.2	83.3	35.6	2.75	3.55	45.71	7.59	12.02	19.05	69.18	28.84	14.3	80.9	34.1	3.63	4.27	54.95	13.8	15.9	72.5	34	2.14	3.31	44.67	5.62	0.03	>100	66.83
	**NCI-H522**	8.63	46.8	21.2	4.57	11.48	45.71	17.38	11.75	22.39	52.48	25.12	3.37	41.2	14.3	5.01	11.75	46.77	17.78	9.37	51.5	22.5	3.16	7.59	40.74	12.3	0.03	88.51	3.46
**Colon Cancer**	**COLO 205**	15.4	80.4	35.2	3.55	5.25	51.29	14.45	19.05	33.88	74.13	38.02	13.9	80	33.3	5.75	8.71	51.29	19.05	15.9	>100	41.1	16.22	28.84	70.79	33.88	2.49	>100	43.25
	**HCC-2998**	3.46	36.5	13.2	2.57	4.37	28.18	7.76	ND	ND	ND	ND	2.72	31.6	9.62	2	3.63	11.75	4.57	9.37	47	21.5	5.13	11.75	44.67	17.38	0.05	26.85	1.22
	**HCT-116**	3.99	42.5	15.2	1.82	2.95	7.41	3.63	6.61	7.94	74.13	24.55	3.82	40.9	13.1	2.88	3.31	>100	>100	2.78	44.3	7.89	3.16	3.63	69.18	14.45	0.03	>100	0.22
	**HCT-15**	3.27	43.7	14.2	2.09	3.55	46.77	5.89	4.07	5.37	48.98	16.6	2.66	36.4	10	2.14	3.47	31.62	7.76	2.46	33.7	7.86	3.24	4.17	74.13	15.49	0.03	>100	9.51
	**HT29**	10.6	52.4	23.6	2.24	3.55	12.59	4.9	6.76	9.55	53.7	20.89	5.09	57.8	19.3	5.5	7.24	60.26	19.95	10.7	68.7	27.1	3.47	4.57	>100	13.49	0.66	38.73	5.51
	**KM12**	4.28	41	15.4	1.95	3.39	7.76	3.89	4.17	8.13	45.71	16.6	3.27	35.9	11	3.63	5.37	42.66	13.18	3.8	40	13.6	3.24	6.46	42.66	13.8	0.05	91.41	0.76
	**SW-620**	3.37	49.3	15	1.91	3.16	7.59	3.8	3.09	4.37	38.9	11.22	2.82	41.6	11.7	3.39	4.07	45.71	10.96	3.59	44.2	13.9	2.57	3.72	46.77	9.77	0.03	>100	86.30
**CNS Cancer**	**SF-268**	13	63.1	28.7	3.72	7.59	97.72	16.98	12.02	24.55	64.57	28.18	13.5	64.2	29.5	10.47	19.05	57.54	24.55	15.6	67.3	32.4	2.63	6.46	54.95	11.22	0.13	91.62	49.66
	**SF-295**	12.5	52.9	25.7	4.37	11.48	43.65	16.98	15.85	28.84	54.95	29.51	12.7	52.7	25.8	3.63	8.71	39.81	14.79	16.1	56.5	30.2	5.5	14.45	66.07	21.38	0.07	>100	1.28
	**SF-539**	2.94	35.5	10.8	2	3.63	8.32	4.07	6.92	16.22	47.86	20.42	2	12.8	4.67	2.19	3.98	12.88	4.9	2.31	27.2	7.02	2.19	4.47	20.89	5.5	0.04	86.90	0.20
	**SNB-19**	4.87	43.1	17.8	7.76	16.98	48.98	21.38	12.02	22.39	50.12	24.55	3.47	37.7	13.4	4.9	11.75	41.69	16.98	5.42	43.2	18.3	5.01	13.18	43.65	17.78	0.04	>100	15.85
	**SNB-75**	ND	ND	ND	1.66	3.8	7.76	3.63	2.19	8.51	28.84	6.76	ND	ND	ND	2.29	8.51	27.54	7.41	ND	ND	ND	2.19	7.59	28.84	6.17	0.83	82.41	24.72
	**U251**	3.63	41.9	15.1	1.91	3.31	7.24	3.72	3.39	4.79	40.74	13.18	2.87	39.7	11.7	1.74	3.09	5.89	3.24	4.1	53	17	3.02	4.17	43.65	12.02	0.04	98.86	13.21
**Melanoma**	**LOX IMVI**	3.26	36.6	12.7	1.66	2.88	5.75	3.09	3.39	4.47	38.02	12.02	2.8	33.7	10.2	1.58	2.75	6.03	3.09	1.85	8.91	4.06	3.24	4.47	38.9	12.02	0.01	>100	12.74
	**MALME-3M**	12.4	60.9	27.5	2.24	5.13	50.12	5.75	14.45	30.9	75.86	33.11	12.5	67.2	29	12.3	24.55	54.95	26.3	13.7	70.8	31.1	5.75	17.78	54.95	20.42	0.44	>100	70.31
	**M14**	6.97	56.7	22.2	2.88	4.17	>100	9.55	14.13	24.55	72.44	32.36	9.02	54.4	22.9	7.08	11.75	83.18	25.7	12.2	66.3	28.4	3.24	5.13	72.44	13.18	0.14	88.31	0.24
	**MDA-MB-435**	4.24	48.7	17.5	2.09	3.55	16.98	5.01	5.5	10.96	48.98	19.05	4.12	45.1	16.7	4.57	7.41	44.67	16.98	7.39	50.9	21.1	2.57	4.47	>100	10.47	0.03	95.06	0.08
	**SK-MEL-2**	11.6	50.1	24.1	10.47	29.51	70.79	27.54	13.8	27.54	56.23	28.18	5.87	47.9	19.5	12.59	26.92	53.7	26.3	13.6	62	29	3.09	10.96	42.66	11.48	8.20	>100	60.53
	**SK-MEL-28**	11.8	51.4	24.7	2.14	4.07	13.49	4.9	14.45	26.3	53.7	28.18	11.9	50.2	24.5	5.5	13.18	44.67	18.2	13.7	52.5	26.8	4.07	9.12	57.54	17.38	3.16	90.78	82.41
	**SK-MEL-5**	6.77	44.2	19.5	4.79	9.12	41.69	16.98	11.22	20.42	48.98	23.44	5.82	43	18.4	13.18	22.91	51.29	26.3	12	49.7	24.4	1.58	3.31	10	3.98	0.01	94.19	53.70
	**UACC-257**	12.6	56.6	26.7	3.63	9.77	63.1	14.79	13.8	26.92	56.23	27.54	11.3	51.9	24.2	3.39	10	38.02	13.49	15.1	61.4	30.4	5.13	18.62	75.86	21.88	4.95	1.97	0.01
	**UACC-62**	5.3	45.7	19.3	2.34	4.17	34.67	8.71	9.77	16.98	48.98	22.39	5.17	44.2	18.6	3.55	7.94	41.69	15.85	10.5	48.1	22.4	2.82	5.37	42.66	12.3	0.01	>100	80.54
**Ovarian Cancer**	**IGROV1**	4.93	62.3	19.7	2.45	4.17	>100	6.46	13.18	25.7	93.33	34.67	3.29	53.4	11.9	3.02	4.9	47.86	12.3	4.43	50.8	14.9	3.31	5.75	>100	13.8	0.06	>100	63.53
	**OVCAR-3**	2.42	24.5	6.81	2.29	4.17	22.91	6.03	2.82	6.46	33.11	10.72	1.88	9.7	4.27	2.4	4.37	23.99	6.46	2.22	16.6	5.28	2.45	5.25	25.7	6.92	0.04	93.54	61.66
	**OVCAR-4**	6.03	44.7	19.6	4.47	12.88	>100	41.69	13.8	25.12	54.95	27.54	10.7	48.5	22.8	10.96	20.42	51.29	23.99	14.9	53.8	28.3	2.82	6.61	51.29	12.59	0.26	20.23	0.16
	**OVCAR-5**	15.6	57.7	30	5.25	15.49	57.54	19.95	17.78	32.36	60.26	33.11	15.4	57.3	29.7	3.63	9.12	38.9	13.8	17.8	58.2	32.2	3.8	12.3	43.65	15.14	0.18	>100	83.18
	**OVCAR-8**	3.99	50.9	16.2	2.69	3.89	>100	7.59	8.51	14.45	54.95	22.91	2.86	37.1	8.77	3.31	4.57	38.02	11.75	7.73	65.2	23.9	2.14	3.55	45.71	5.5	0.03	90.78	78.52
	**NCI/ADR-RES**	5.92	85	24.9	2.88	5.01	>100	>100	3.72	7.08	77.62	17.38	6.04	73.4	23.4	2.51	4.27	>100	5.75	14.9	82.8	35.2	2.4	4.37	>100	7.41	0.06	90.36	39.17
	**SK-OV-3**	11	61.8	26.1	17.38	51.29	>100	>100	15.85	30.2	66.07	32.36	13.8	74.9	32.2	5.89	15.14	45.71	18.62	18.6	76.9	37.8	7.41	46.77	>100	>100	28.44	89.54	0.36
**Renal Cancer**	**786-0**	4.59	47.1	17.6	2.51	4.07	37.15	6.31	4.68	7.76	58.88	20.42	3.33	41.8	13.3	3.98	6.31	50.12	15.85	3.99	54	17.6	4.57	6.61	97.72	20.89	0.02	>100	>100
	**A498**	13.6	52.6	26.8	19.5	47.86	91.2	42.66	15.85	30.9	56.23	29.51	13.5	51.8	26.5	14.79	29.51	54.95	28.18	15.5	55.3	29.3	11.48	43.65	>100	34.67	0.05	>100	25.47
	**ACHN**	7.02	44.3	19.6	2.29	3.72	21.38	6.03	13.8	23.99	51.29	26.92	7.67	45.4	20.3	4.07	6.17	38.9	14.79	11.3	48.4	23.3	3.89	6.46	54.95	16.6	0.01	>100	0.72
	**CAKI-1**	6.89	45.4	20.2	4.68	15.49	69.18	20.89	4.47	8.71	43.65	17.38	10	47.2	21.8	10.47	20.42	51.29	22.91	13.9	52.7	27.1	2.57	4.68	45.71	13.18	0.04	97.05	8.22
	**RXF 393**	3.02	37.2	13	1.91	5.13	10	4.37	5.89	23.99	48.98	19.95	2.58	33	9.43	10.23	24.55	47.86	22.39	4.88	44.8	18.6	2.51	11.22	30.9	7.08	0.89	>100	13.87
	**SN12C**	3.95	46.7	17	2	3.55	8.91	4.27	4.9	10.72	43.65	18.2	3.15	36.8	13.1	2.69	4.37	33.11	10.23	6.06	44.7	19.3	2.29	3.89	46.77	9.55	0.01	91.62	82.04
	**TK-10**	12.5	50.7	25.2	19.5	45.71	>100	47.86	15.14	28.18	54.95	28.84	3.04	35.2	11.8	11.22	22.91	50.12	23.44	12.4	51.3	25.3	5.01	16.22	47.86	17.78	0.57	>100	11.51
	**UO-31**	2.88	41.2	15.7	1.55	2.88	6.03	3.09	4.07	11.22	47.86	18.2	2.46	39.7	13.5	3.89	10.72	43.65	16.98	4.86	46.3	18.8	3.02	7.76	46.77	15.49	0.19	>100	4.16
**PC**	**PC-3**	5.82	>100	29.7	2.88	4.9	>100	13.8	5.37	9.77	81.28	23.44	4.68	>100	26	4.07	8.13	56.23	17.78	10.3	>100	37.3	5.01	8.91	>100	41.69	0.03	>100	51.40
	**DU-145**	4.32	39.9	15.7	3.24	4.79	42.66	11.48	4.9	8.91	42.66	17.38	3.52	35.3	12	2.4	3.89	23.44	6.17	3.41	34.5	11	3.24	5.75	69.18	15.49	0.01	>100	78.89
**Breast Cancer**	**MCF7**	2.65	37	11.4	3.31	8.13	37.15	12.3	3.16	4.68	43.65	13.18	2.57	38.7	10.6	2.75	6.31	38.02	9.77	2.67	51.3	11.7	3.02	4.27	45.71	10.72	0.04	>100	>100
	**MDA-MB-231/ATCC**	15.7	66.1	32.2	2.45	6.17	39.81	6.92	16.22	30.9	61.66	31.62	10.9	50.9	23.5	5.25	16.22	44.67	17.78	12	51.5	24.8	3.24	10	>100	13.18	0.01	91.62	73.62
	**HS 578T**	13.2	>100	40.2	3.09	>100	>100	85.11	21.38	>100	>100	77.62	10.4	>100	33	17.38	67.61	>100	52.48	13	80.9	32.4	2.88	>100	>100	39.81	0.15	>100	0.22
	**BT-549**	2.34	33.1	8.27	2.24	5.89	28.18	6.17	5.75	20.89	57.54	20.42	2.6	30.6	7.28	3.8	13.49	43.65	14.79	5.04	48.5	19.3	2.95	11.75	50.12	10.72	0.01	99.54	8.09
	**T-47D**	3.94	>100	28.8	3.39	7.94	>100	20.89	3.47	14.13	81.28	19.05	2.77	61.7	11.8	2	4.9	66.07	7.08	4.96	>100	25.7	2.19	7.24	>100	13.18	47.97	>100	2.04
	**MDA-MB-468**	1.93	25.3	6.46	3.8	12.59	46.77	15.85	2.51	12.02	30.2	8.32	1.79	27.2	6.77	2.95	7.76	40.74	10.72	2.2	31.4	8	1.55	5.75	17.78	4.47	ND	ND	ND

ND: not determined.

**Table 3:** The binding affinities and interactions of **2e**, **2g**, **2h**, and CA-4.

Compound	Binding Affinity (kcal/mol)	Classical Hydrogen Bonding	Non-Classical Hydrogen Bonding	Hydrophobic Interactions
CA4	−9.2	Cys241	Asn258, Asn350, Val238	Met259, Ala316, Ala354, Val181, Lys352, Cys241, Leu242, Leu255, Ile318, Ile378, Ala180, Leu248, Ala250, Asn258
**2e**	−8.6	Cys241	Cys241	Met259, Lys352, Leu248, Leu255, Ala180, Val181, Lys352
**2g**	−7.3	Cys241	N/A	Thr179, Ala180, Leu248, Leu255, Ala316, Ala250
**2h**	−8.9	Cys241, Gln247	Ser178	Cys241, Leu255, Ala316, Ala180, Leu248, Lys352

## Data Availability

The original contributions presented in this study are included in the article; further inquiries can be directed to the corresponding author.

## Supplementary Materials

The following supporting information can be downloaded at: https://www.mdpi.com/article/10.3390/ph17091154/s1, Figures S1–S48: NMR and Mass data; Table S1: Physicochemical properties of target compounds 2a–p and combretastatin CA4; Table S2: Lipophilicity parameters of target compounds 2a–p and combretastatin CA4; Table S3: Water solubility parameters of target compounds 2a–p and combretastatin CA4; Table S4: Pharmacokinetics of target compounds 2a–p and combretastatin CA4; Table S5: Drug likeness parameters of target compounds 2a–r and combretastatin CA4.
