# Supplementary material for: Synthesis of New Thiazole-Privileged Chalcones as Tubulin Polymerization Inhibitors with Potential Anticancer Activities
Source: Pharmaceuticals (Basel). 2024 Aug 31;17(9):1154. doi: 10.3390/ph17091154 (PMC11435058; doi:10.3390/ph17091154)
Supplement: Supplementary file 1 [file pharmaceuticals-17-01154-s001.zip › pharmaceuticals-3153734-supplementary.pdf]

Synthesis of new thiazole-privileged chalcones as tubulin polymerization inhibitors with potential anticancer activities

Hamada Hashem [1*], Abdelfattah Hassan [2,3], Walid M. Abdelmagid [4], Ahmed G. K. Habib [5], Mohamed A. A. Abdel-Aal [6], Ali M. Elshamsy [7], Amr El Zawily [8,9], Ibrahim Taha Radwan [10], Stefan Bräse [11*], Ahmed S. Abdel- Samea [12] and Safwat M. Rabea [13,14]

[1]Pharmaceutical Chemistry Department, Faculty of Pharmacy, Sohag University, Sohag, 82524, Egypt
[2]Medicinal Chemistry Department, Faculty of Pharmacy, South Valley University, Qena, Egypt
[3]Medicinal Chemistry Department, Clinical Pharmacy Program, South Valley National University, Qena, Egypt
[4]Medicinal Chemistry and Drug Discovery Research Centre, Swenam College, 210-6125 Sussex Avenue, Burnaby, BC, V5H 4G1, Canada
[5]Department of Biotechnology and Life Sciences, Faculty of Postgraduate Studies for Advanced Sciences, Beni-Suef University, Beni-Suef, Egypt
[6]Pharmaceutical Chemistry Department, Faculty of Pharmacy, Al-Azhar University, Assiut Branch, Assiut 71524, Egypt
[7]Medicinal Chemistry Department, Faculty of Pharmacy, Deraya University, Minia, Egypt
[8]Department of Plant and microbiology, Faculty of science, Damanhour University, Damanhour, 22511, Egypt
[9]Division of Pharmaceutics and translation therapeutics, college of pharmacy, University of Iowa, Iowa city, IA, 52242, USA
[10]Supplementary General Sciences Department, Faculty of Oral and Dental Medicine, Future University in Egypt, Cairo 11835, Egypt.
[11]Institute of Biological and Chemical Systems – Functional Molecular Systems (IBCS-FMS), Karlsruhe Institute of Technology (KIT), Kaiserstrasse 12, 76131 Karlsruhe
[12]Pharmacology and Toxicology Department, Faculty of Pharmacy, Deraya University, Minia, Egypt
[13]Medicinal Chemistry Department, Faculty of Pharmacy, Minia University, Minia 61519, Egypt.
[14]Apogee Pharmaceuticals Inc., 4475 Wayburne Dr, Suite 105, Burnaby, BC V6V2H8, Canada.
*** Authors to whom correspondence should be addressed.**

List of contents

Appendix A 3

4.2. Biology 3

4.2.1. Screening of the anticancer activity against a panel of 60 cell Lines. 3

4.2.2. In Vitro Tubulin Polymerization Inhibition Assay. 3

4.2.3. In silico studies 4

4.2.3.1 Molecular Docking: 4

4.2.3.2. In silico Physicochemical and Pharmacokinetic properties 4

Table *S*1: Physicochemical properties of target compounds **2a-*p*** and combretastatin *CA4* 5

Table *S*2: Lipophilicity parameters of target compounds **2a-*p*** and combretastatin *CA4*. ...6

Table S3: Water solubility parameters of target compounds **2a-*p*** and combretastatin *CA4*. 7

Table *S*4: Pharmacokinetics of target compounds **2a-*p*** and combretastatin *CA4*. 8

Table *S*5: Drug likeness parameters of target compounds **2a-*r*** and combretastatin *CA4*...9

References: 9

NMR and Mass data 11

List of Figures 11

Screening the anticancer activity 37

Screening results at 10 µM 37

Anticancer activity at five doses 54

Appendix A

4.2. Biology

4.2.1. Screening of the anticancer activity against a panel of 60 cell Lines.

The methodology of the NCI anticancer screening has been described in detail elsewhere (http://www.dtp.nci.nih.gov.) Briefly, the primary anticancer assay was performed at approximately 60 human tumor cell lines panel derived from nine neoplastic diseases, in accordance with the protocol of the Drug Evaluation Branch, National Cancer Institute, Bethesda. Tested compounds were added to the culture at a single concentration (10µM) and the cultures were incubated for 48 h. End-point determinations were made with a protein binding dye, SRB. Results for each tested compound was reported as the percent of growth of the treated cells when compared to the untreated control cells. The percentage growth was evaluated spectrophotometrically versus controls not treated with test agents [1].

4.2.2. In Vitro Tubulin Polymerization Inhibition Assay.

The study investigated the interaction of several proposed compounds (2e, 2g, 2h, 2p, and the reference drug CD-4) with the microtubule system. Specifically, the in vitro inhibition of tubulin polymerization was evaluated for these compounds. The standard tubulin polymerization reaction was carried out, containing 100 µL of 4 mg/mL tubulin in a buffer solution of 80 mM PIPES (pH 6.9), 0.5 mM EGTA, 2 mM MgCl2, and 1 mM GTP. Polymerization was initiated by incubating the reaction at 37°C, and the progress was monitored by measuring the absorbance at 340 nm. Under these conditions, polymerization typically reaches a maximum absorbance of 0.15-0.25 within 30 minutes, exhibiting the three characteristic phases: nucleation, growth, and steady state. In this experimental setup, an absorbance of 0.1 at 340 nm corresponds to approximately 1 mg/mL of polymerized tubulin, meaning that around

40% of the tubulin is polymerized, allowing for the detection of both enhancers and inhibitors of polymerization. The inhibitory effect of each compound on tubulin polymerization was quantified by determining the IC50 values, which were reported as the mean ± standard deviation from three independent experiments [2].

4.2.3. In silico studies

4.2.3.1 Molecular Docking:

The crystal structure of tubulin-colchicine complex (PDB code: 4O2B) was downloaded from the Protein Data Bank [3]. Structures of compounds **2e**, **2h** and **2g** were drawn and optimized using MarvinSketch and Avogadro molecular editors [4]. The protein was prepared using autodock tools where the co-crystallized water molecules and colchicine were removed then kollman charges and polar hydrogens were added. The grid coordinates for tubulin were set to 15.951x66.804x43.33 for x, y and z axes, respectively with grid dimensions of 80x80x80. Autodock vina was used for molecular docking [5] and the best docking poses were visualized using Discovery Studio Visualizer.

4.2.3.2. In silico Physicochemical and Pharmacokinetic properties

The physicochemical and pharmacokinetic parameters of 5a-h and 7a-ah were predicted using the SwissADME tool (http://www.swissadme.ch/index.php). Lipophilicity was estimated through five independent models: WLOGP, XLOGP3, MLOGP, iLOGP, and SILICOS-IT. The arithmetic mean of these models was calculated to provide a consensus log Po/w value. The BOILED Egg model maps polarity (expressed as TPSA) against lipophilicity (using WLOGP, SwissADME's own lipophilicity model). In this plot, the white region indicates a favorable likelihood for gastrointestinal (GI) absorption, while the yolk region suggests a good chance for blood-brain barrier (BBB) permeability. The bioavailability radar illustrates six different physicochemical properties: size, polarity, lipophilicity, solubility, flexibility, and saturation [6].

Table *S*1: Physicochemical properties of target compounds **2a-*p*** and combretastatin *CA4*

Molecule	MW	#Heavy atoms	#Aromatic heavy atoms	Fraction Csp3	#Rotatable bonds	#H-bond acceptors	#H-bond donors	MR	TPSA
2a	261.36	17	11	0.08	3	2	0	74.14	97.00
2b	279.35	18	11	0.08	3	3	0	74.1	97.00
2c	279.35	18	11	0.08	3	3	0	74.1	97.00
2d	295.81	18	11	0.08	3	2	0	79.15	97.00
2e	295.81	18	11	0.08	3	2	0	79.15	97.00
2f	295.81	18	11	0.08	3	2	0	79.15	97.00
2g	340.26	18	11	0.08	3	2	0	81.84	97.00
2h	306.36	20	11	0.08	4	4	0	82.96	142.82
2i	306.36	20	11	0.08	4	4	0	82.96	142.82
2j	329.36	21	11	0.14	4	5	0	79.14	97.00
2k	275.39	18	11	0.14	3	2	0	79.1	97.00
2l	304.43	20	11	0.2	4	2	0	88.35	100.24
2m	291.39	19	11	0.14	4	3	0	80.63	106.23
2n	321.41	21	11	0.2	5	4	0	87.12	115.46
2o	321.41	21	11	0.2	5	4	0	87.12	115.46
2p	321.41	21	11	0.2	5	4	0	87.12	115.46
2q	351.44	23	11	0.25	6	5	0	93.61	124.69
2r	311.42	21	15	0.06	3	2	0	91.64	97.00
CA4	316.35	23	12	0.22	6	5	1	89.8	57.15

Table *S2*: Lipophilicity parameters of target compounds **2a-*p*** and combretastatin *CA4*.

Molecule	iLOGP	XLOGP3	WLOGP	MLOGP	Silicos-IT Log P	Consensus Log P	ESOL Log S	ESOL Solubility (mg/ml)	ESOL Solubility (mol/l)	ESOL Class
2a	2.81	3.94	3.53	1.91	4.78	3.39	-4.22	1.56E-02	5.98E-05	Moderately soluble
2b	2.92	4.04	4.09	2.31	5.2	3.71	-4.37	1.19E-02	4.25E-05	Moderately soluble
2c	2.89	4.04	4.09	2.31	5.2	3.71	-4.37	1.19E-02	4.25E-05	Moderately soluble
2d	2.89	4.57	4.18	2.44	5.42	3.9	-4.81	4.61E-03	1.56E-05	Moderately soluble
2e	3.08	4.57	4.18	2.44	5.42	3.94	-4.81	4.61E-03	1.56E-05	Moderately soluble
2f	3.08	4.57	4.18	2.44	5.42	3.94	-4.81	4.61E-03	1.56E-05	Moderately soluble
2g	3.19	4.63	4.29	2.57	5.46	4.03	-5.12	2.58E-03	7.57E-06	Moderately soluble
2h	2.47	3.77	3.44	0.82	2.63	2.63	-4.26	1.69E-02	5.53E-05	Moderately soluble
2i	2.46	3.77	3.44	0.82	2.63	2.62	-4.26	1.69E-02	5.53E-05	Moderately soluble
2j	2.86	4.82	5.7	2.82	5.85	4.41	-5.04	2.99E-03	9.07E-06	Moderately soluble
2k	3.08	4.3	3.84	2.17	5.28	3.73	-4.51	8.50E-03	3.09E-05	Moderately soluble
2l	3.15	4.06	3.59	1.81	4.44	3.41	-4.43	1.14E-02	3.73E-05	Moderately soluble
2m	3.11	3.91	3.54	1.56	4.82	3.39	-4.27	1.55E-02	5.32E-05	Moderately soluble
2n	3.23	3.88	3.54	1.23	4.88	3.35	-4.33	1.49E-02	4.63E-05	Moderately soluble
2o	3.42	3.88	3.54	1.23	4.88	3.39	-4.33	1.49E-02	4.63E-05	Moderately soluble
2p	3.07	3.88	3.54	1.23	4.88	3.32	-4.33	1.49E-02	4.63E-05	Moderately soluble
2q	3.49	3.85	3.55	0.92	4.96	3.35	-4.4	1.39E-02	3.96E-05	Moderately soluble
2r	2.96	5.19	4.68	2.73	5.85	4.28	-5.37	1.33E-03	4.26E-06	Moderately soluble
CA4	3.42	3.72	3.38	2.1	3.73	3.27	-4.14	2.32E-02	7.33E-05	Moderately soluble

Table S3: Water solubility parameters of target compounds **2a-*p*** and combretastatin *CA4*.

Molecule	Ali Log S	Ali Solubility (mg/ml)	Ali Solubility (mol/l)	Ali Class	Silicos-IT LogSw	Silicos-IT Solubility (mg/ml)	Silicos-IT Solubility (mol/l)	Silicos-IT class
2a	-5.68	5.50E-04	2.11E-06	Moderately soluble	-4.35	1.17E-02	4.47E-05	Moderately soluble
2b	-5.78	4.63E-04	1.66E-06	Moderately soluble	-4.62	6.64E-03	2.38E-05	Moderately soluble
2c	-5.78	4.63E-04	1.66E-06	Moderately soluble	-4.62	6.64E-03	2.38E-05	Moderately soluble
2d	-6.33	1.38E-04	4.67E-07	Poorly soluble	-4.96	3.28E-03	1.11E-05	Moderately soluble
2e	-6.33	1.38E-04	4.67E-07	Poorly soluble	-4.96	3.28E-03	1.11E-05	Moderately soluble
2f	-6.33	1.38E-04	4.67E-07	Poorly soluble	-4.96	3.28E-03	1.11E-05	Moderately soluble
2g	-6.39	1.38E-04	4.05E-07	Poorly soluble	-5.17	2.32E-03	6.81E-06	Moderately soluble
2h	-6.46	1.06E-04	3.45E-07	Poorly soluble	-3.71	5.92E-02	1.93E-04	Soluble
2i	-6.46	1.06E-04	3.45E-07	Poorly soluble	-3.71	5.92E-02	1.93E-04	Soluble
2j	-6.59	8.47E-05	2.57E-07	Poorly soluble	-5.21	2.04E-03	6.19E-06	Moderately soluble
2k	-6.05	2.45E-04	8.91E-07	Poorly soluble	-4.73	5.08E-03	1.85E-05	Moderately soluble
2l	-5.87	4.11E-04	1.35E-06	Moderately soluble	-4.45	1.09E-02	3.58E-05	Moderately soluble
2m	-5.84	4.22E-04	1.45E-06	Moderately soluble	-4.47	9.92E-03	3.40E-05	Moderately soluble
2n	-6	3.20E-04	9.95E-07	Poorly soluble	-4.58	8.42E-03	2.62E-05	Moderately soluble
2o	-6	3.20E-04	9.95E-07	Poorly soluble	-4.58	8.42E-03	2.62E-05	Moderately soluble
2p	-6	3.20E-04	9.95E-07	Poorly soluble	-4.58	8.42E-03	2.62E-05	Moderately soluble
2q	-6.16	2.40E-04	6.84E-07	Poorly soluble	-4.69	7.16E-03	2.04E-05	Moderately soluble
2r	-6.97	3.31E-05	1.06E-07	Poorly soluble	-6.01	3.06E-04	9.84E-07	Poorly soluble
CA4	-4.61	7.74E-03	2.45E-05	Moderately soluble	-4.92	3.79E-03	1.20E-05	Moderately soluble

Table *S4*: Pharmacokinetics of target compounds **2a-*p*** and combretastatin *CA4*.

Molecule	GI absorption	BBB permeant	Pgp substrate	CYP1A2 inhibitor	CYP2C19 inhibitor	CYP2C9 inhibitor	CYP2D6 inhibitor	CYP3A4 inhibitor
2a	High	No	No	Yes	Yes	Yes	No	No
2b	High	No	No	Yes	Yes	Yes	No	No
2c	High	No	No	Yes	Yes	Yes	No	No
2d	High	No	No	Yes	Yes	Yes	No	No
2e	High	No	No	Yes	Yes	Yes	No	No
2f	High	No	No	Yes	Yes	Yes	No	No
2g	High	No	No	Yes	Yes	Yes	No	No
2h	Low	No	No	Yes	Yes	Yes	No	Yes
2i	Low	No	No	Yes	Yes	Yes	No	Yes
2j	High	No	No	Yes	Yes	Yes	No	No
2k	High	No	No	Yes	Yes	Yes	No	No
2l	High	No	No	Yes	Yes	Yes	No	Yes
2m	High	No	No	Yes	Yes	Yes	No	No
2n	High	No	No	Yes	Yes	Yes	No	No
2o	High	No	No	Yes	Yes	Yes	No	No
2p	High	No	No	Yes	Yes	Yes	No	No
2q	High	No	No	Yes	Yes	Yes	No	No
2r	High	No	No	Yes	Yes	Yes	No	Yes
CA4	High	Yes	No	Yes	Yes	Yes	Yes	Yes

Table *S*5: Drug likeness parameters of target compounds **2a-*r*** and combretastatin *CA4*.

Molecule	Lipinski #violations	Ghose #violations	Veber #violations	Egan #violations	Muegge #violations	Bioavailability Score
2a	0	0	0	0	0	0.55
2b	0	0	0	0	0	0.55
2c	0	0	0	0	0	0.55
2d	0	0	0	0	0	0.55
2e	0	0	0	0	0	0.55
2f	0	0	0	0	0	0.55
2g	0	0	0	0	0	0.55
2h	0	0	1	1	0	0.55
2i	0	0	1	1	0	0.55
2j	0	1	0	0	0	0.55
2k	0	0	0	0	0	0.55
2l	0	0	0	0	0	0.55
2m	0	0	0	0	0	0.55
2n	0	0	0	0	0	0.55
2o	0	0	0	0	0	0.55
2p	0	0	0	0	0	0.55
2q	0	0	0	0	0	0.55
2r	0	0	0	0	1	0.55
CA4	0	0	0	0	0	0.55

References:

[1] H.H.H. Mohammed, A.A. Abd El-Hafeez, S.H. Abbas, E.-S.M.N. Abdelhafez, G.E.-D.A. Abuo-Rahma, New antiproliferative 7-(4-({N}-substituted carbamoylmethyl)piperazin-1-yl) derivatives of ciprofloxacin induce cell cycle arrest at {G2}/{M} phase, Bioorganic & Medicinal Chemistry 24 (2016) 4636–4646. https://doi.org/10.1016/j.bmc.2016.07.070.

[2] A.O. El-Abd, S.M. Bayomi, A.K. El-Damasy, B. Mansour, N.I. Abdel-Aziz, M.A. El-Sherbeny, Synthesis and Molecular Docking Study of New Thiazole Derivatives as Potential Tubulin Polymerization Inhibitors, ACS Omega 7 (2022) 33599–33613. https://doi.org/10.1021/ACSOMEGA.2C05077/ASSET/IMAGES/LARGE/AO2C05077_0005.JPEG.

[3] A.E. Prota, F. Danel, F. Bachmann, K. Bargsten, R.M. Buey, J. Pohlmann, S. Reinelt, H. Lane, M.O. Steinmetz, The novel microtubule-destabilizing drug BAL27862 binds to the colchicine site of tubulin with distinct effects on microtubule organization, Journal of Molecular Biology 426 (2014) 1848–1860. https://doi.org/10.1016/J.JMB.2014.02.005.

[4] M.D. Hanwell, D.E. Curtis, D.C. Lonie, T. Vandermeerschd, E. Zurek, G.R. Hutchison, Avogadro: An advanced semantic chemical editor, visualization, and analysis platform,

Journal of Cheminformatics 4 (2012) 1–17. https://doi.org/10.1186/1758-2946-4-17/FIGURES/14.
[5] O. Trott, A.J. Olson, AutoDock Vina: Improving the speed and accuracy of docking with a new scoring function, efficient optimization, and multithreading, Journal of Computational Chemistry 31 (2010) 455–461. https://doi.org/10.1002/JCC.21334.
[6] A. Hassan, M. Badr, D. Abdelhamid, H.A. Hassan, M.A.S. Abourehab, G.E.A. Abuo-Rahma, Design, synthesis, *in vitro* antiproliferative evaluation and *in silico* studies of new VEGFR-2 inhibitors based on 4-piperazinylquinolin-2(1*H*)-one scaffold, Bioorganic Chemistry 120 (2022) 105631. https://doi.org/10.1016/j.bioorg.2022.105631.

NMR and Mass data

List of Figures

Figure S1: ^{1}H NMR spectrum of compound 2a. 12
Figure S2: ^{13}C NMR spectrum of compound **2a**. 13
Figure S3: Mass spectrum of compound **2a**. 13
Figure S4: ^{1}H NMR spectrum of compound **2b**. 14
Figure S5: ^{13}C NMR spectrum of compound **2b**. 14
Figure S6: Mass spectrum of compound **2b**. 15
Figure S7: ^{1}H NMR spectrum of compound **2c**. 15
Figure S8: ^{13}C NMR spectrum of compound **2c**. 16
Figure S9: Mass spectrum of compound **2c**. 16
Figure S10: ^{1}H NMR spectrum of compound **2d**. 17
Figure S11: ^{13}C NMR spectrum of compound **2d**. 17
Figure S12: Mass spectrum of compound **2d**. 18
Figure S13: ^{1}H NMR spectrum of compound **2e**. 18
Figure S14: ^{13}C NMR spectrum of compound **2e**. 19
Figure S15: Mass spectrum of compound **2e**. 19
Figure S16: ^{1}H NMR spectrum of compound **2f**. 20
Figure S17: ^{13}C NMR spectrum of compound **2f.** 20
Figure S18: Mass spectrum of compound **2f**. 21
Figure S19: ^{1}H NMR spectrum of compound **2g**. 21
Figure S20: ^{13}C NMR spectrum of compound **2g**. 22
Figure S21: Mass spectrum of compound **2g**. 22
Figure S22: ^{1}H NMR spectrum of compound **2h**. 23
Figure S23: ^{13}C NMR spectrum of compound **2h**. 23
Figure S24: Mass spectrum of compound **2h**. 24
Figure S25: ^{1}H NMR spectrum of compound **2i**. 24
Figure S26: ^{13}C NMR spectrum of compound **2i**. 25
Figure S27: Mass spectrum of compound **2i**. 25
Figure S28: ^{1}H NMR spectrum of compound **2j**. 26
Figure S29: ^{1}H NMR spectrum of compound **2j**. 26
Figure S30: Mass spectrum of compound **j**. 27
Figure S31: ^{1}H NMR spectrum of compound **2k**. 27
Figure S32: ^{13}C NMR spectrum of compound **2k**. 28
Figure S33: Mass spectrum of compound **2k**. 28
Figure S34: ^{1}H NMR spectrum of compound **2l**. 29
Figure S35: ^{13}C NMR spectrum of compound **2l**. 29
Figure S36: Mass spectrum of compound **2l**. 30
Figure S37: ^{1}H NMR spectrum of compound **2m**. 30
Figure S38: ^{13}C NMR spectrum of compound **2m**. 31
Figure S39: Mass spectrum of compound **2m**. 31

Figure S40: ^{1}H NMR spectrum of compound **2n**..32
Figure S41: ^{13}C NMR spectrum of compound **2n**..32
Figure S42: Mass spectrum of compound **2n**..33
Figure S43: ^{1}H NMR spectrum of compound **2o**..33
Figure S44: ^{13}C NMR spectrum of compound **2o**..34
Figure S45: Mass spectrum of compound **2o**..34
Figure S46: ^{1}H NMR spectrum of compound **2p**..35
Figure S47: ^{13}C NMR spectrum of compound **2p**..35
Figure S48: Mass spectrum of compound **2p**..36

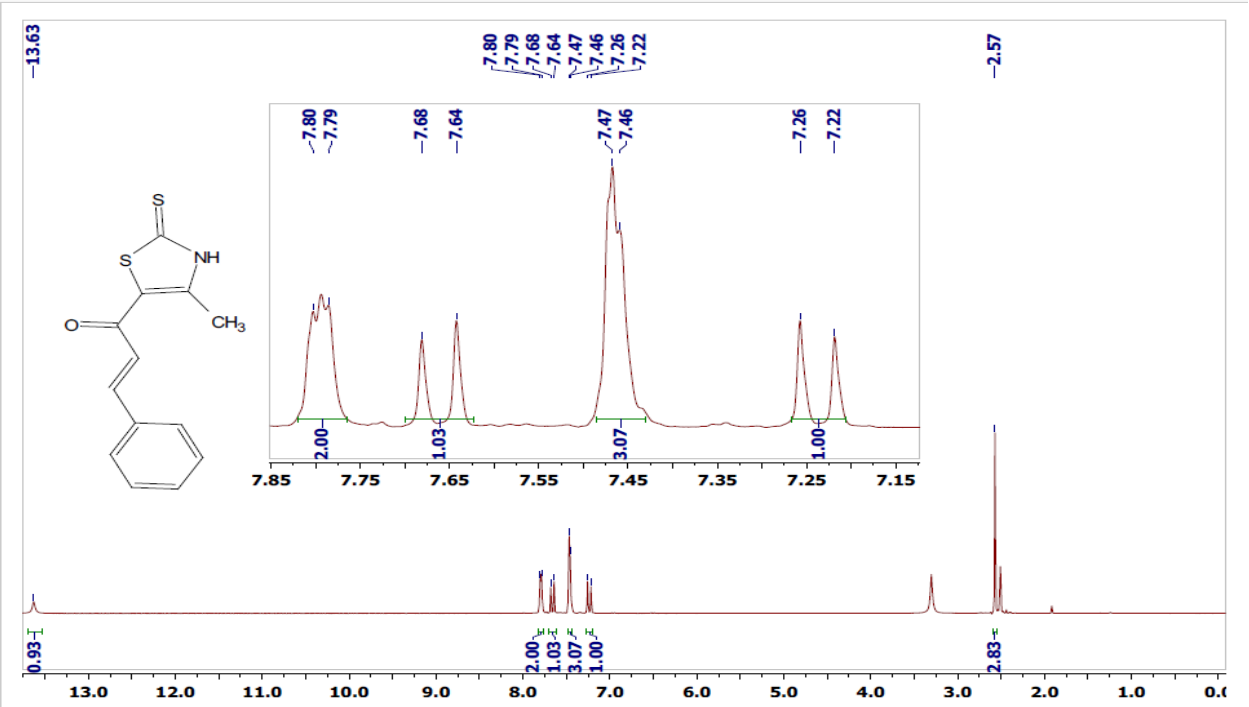

Figure S1: ^{1}H NMR spectrum of compound 2a.

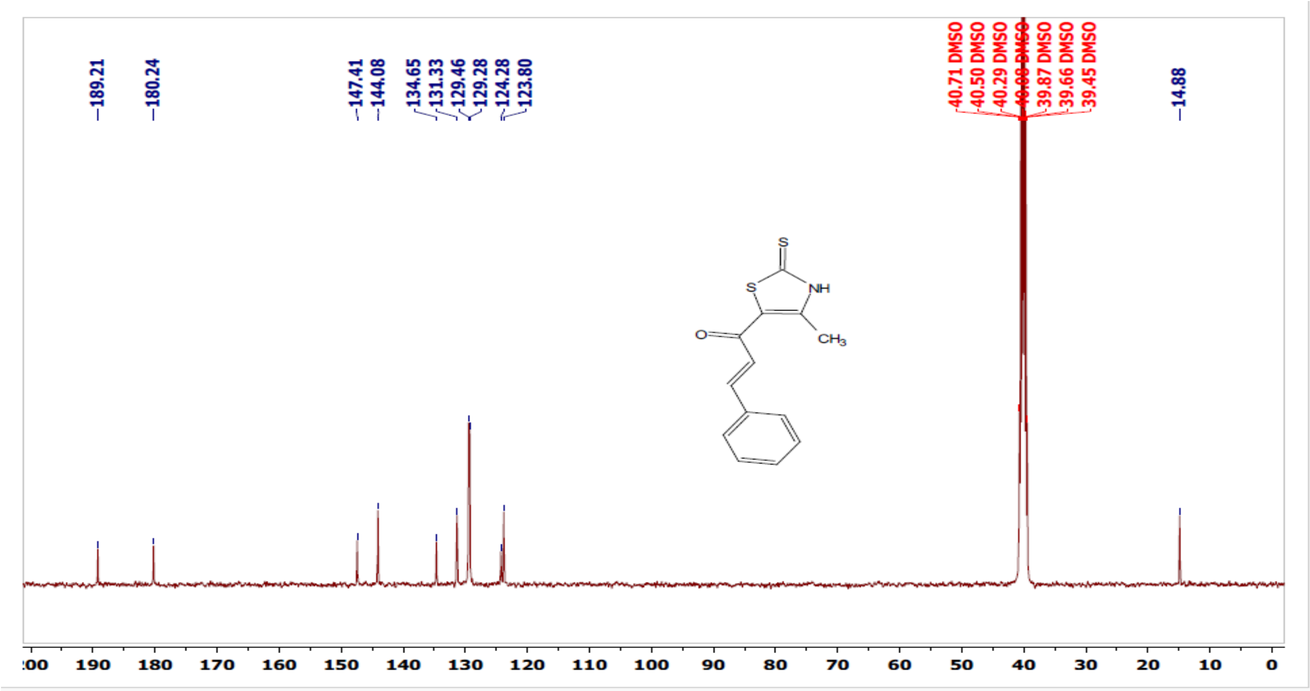

Figure S2: [13] C NMR spectrum of compound **2a**.

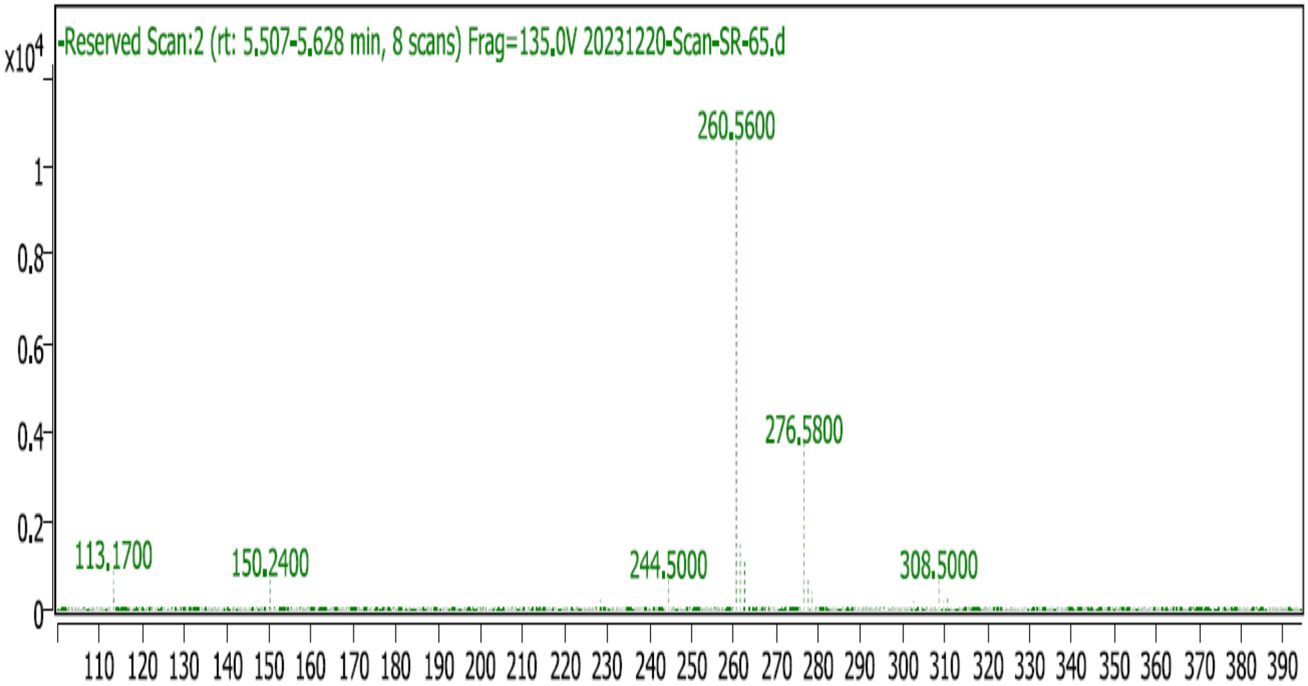

Figure S3: Mass spectrum of compound **2a**.

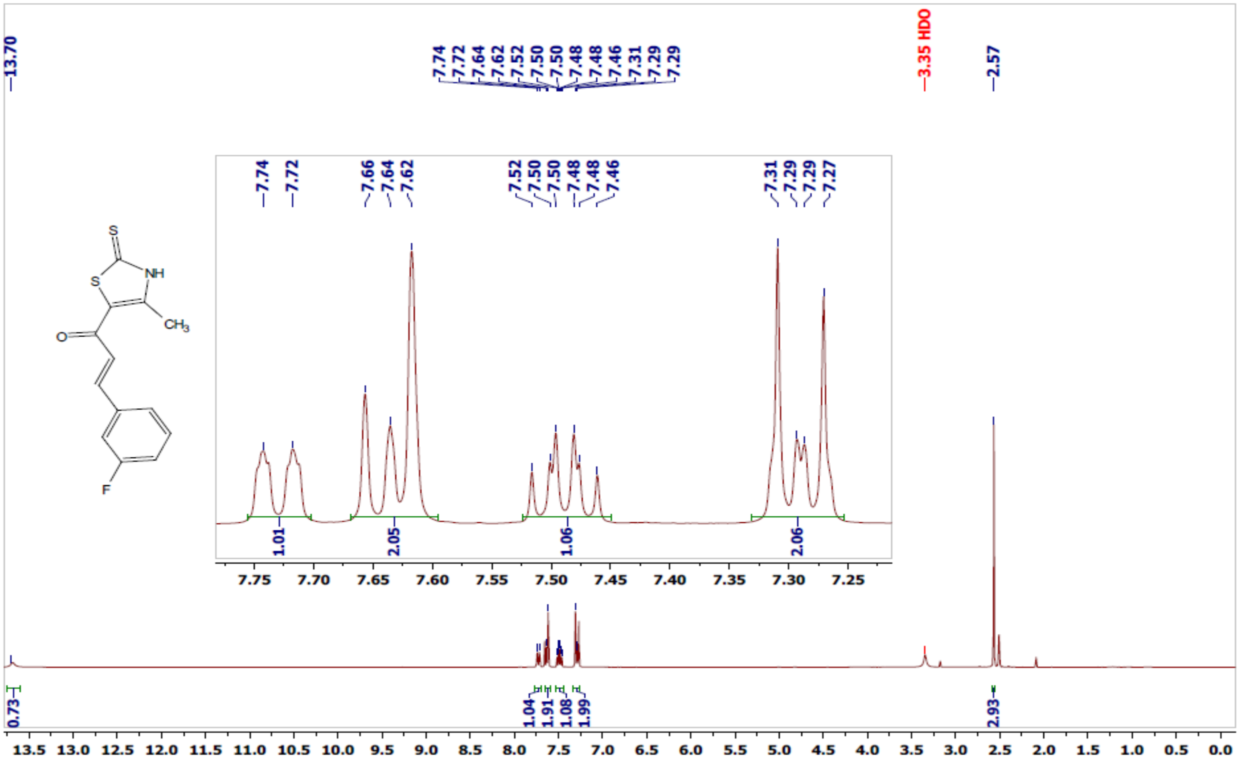

Figure S4: ^{1}H NMR spectrum of compound **2b**.

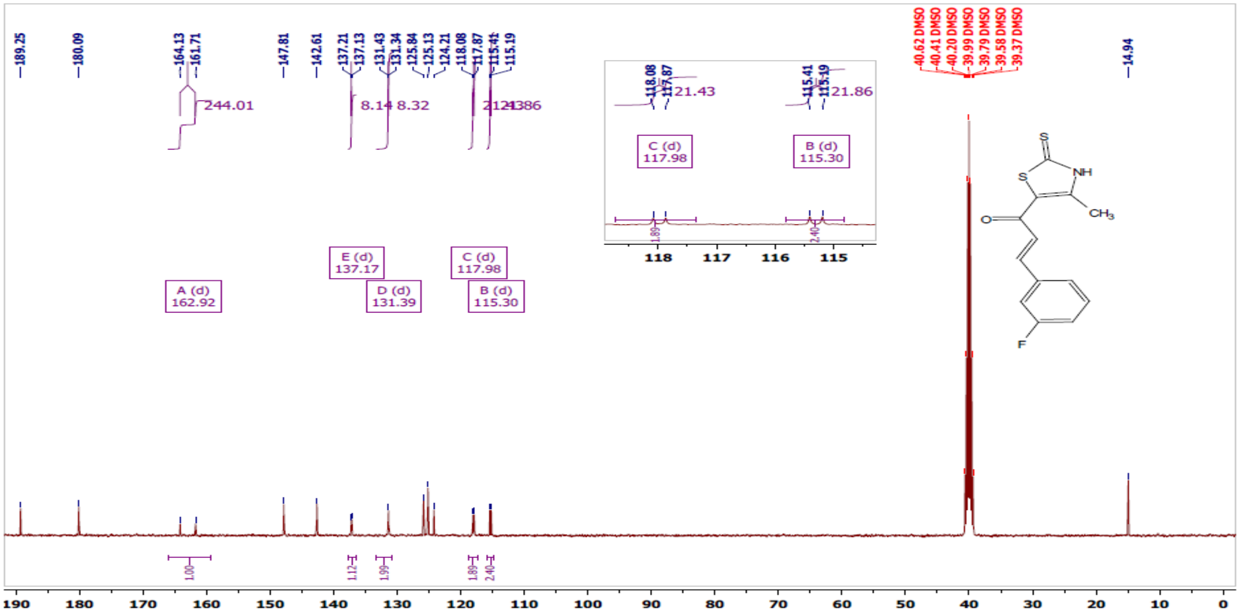

Figure S5: ^{13}C NMR spectrum of compound **2b**.

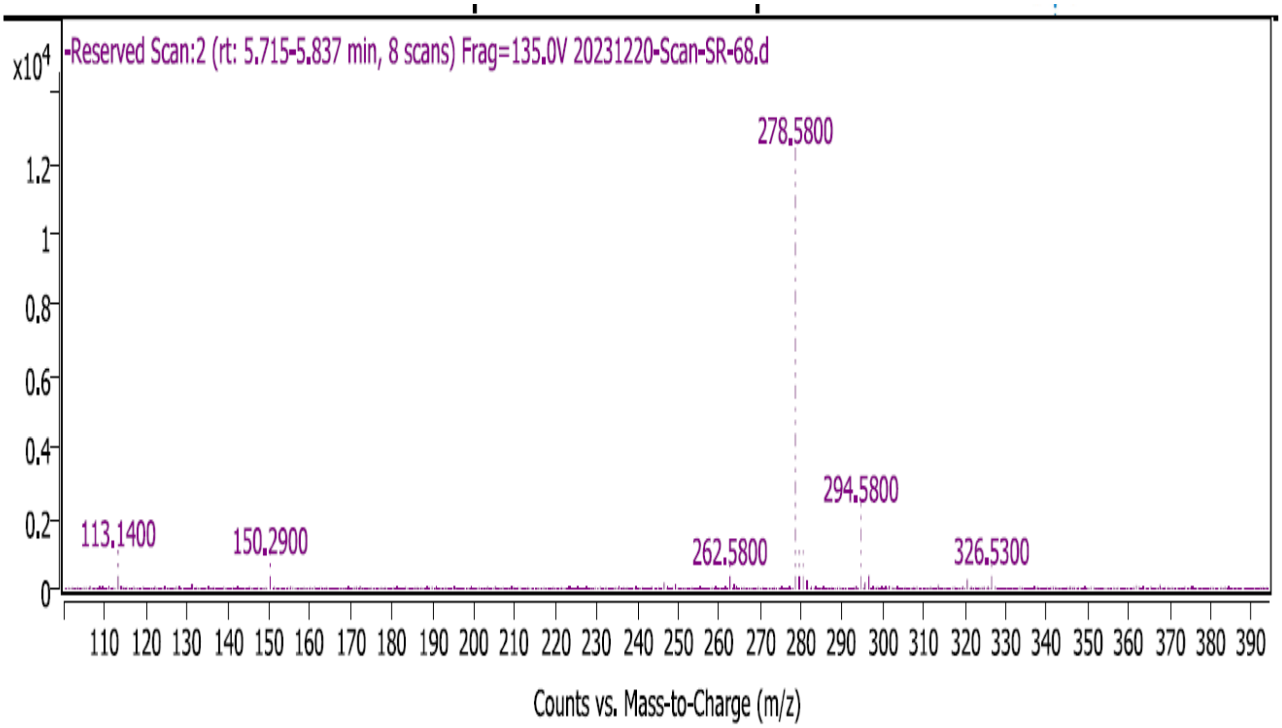

Figure S6: Mass spectrum of compound **2b**.

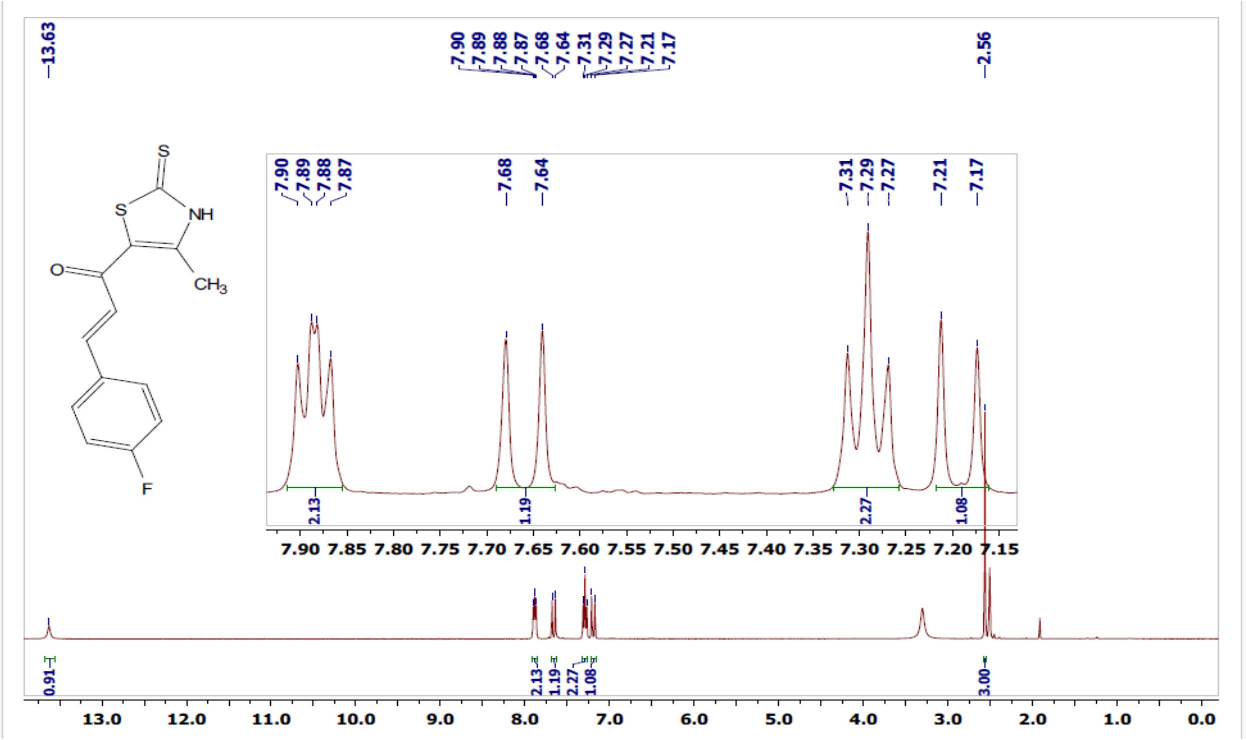

Figure S7: ^{1}H NMR spectrum of compound **2c**.

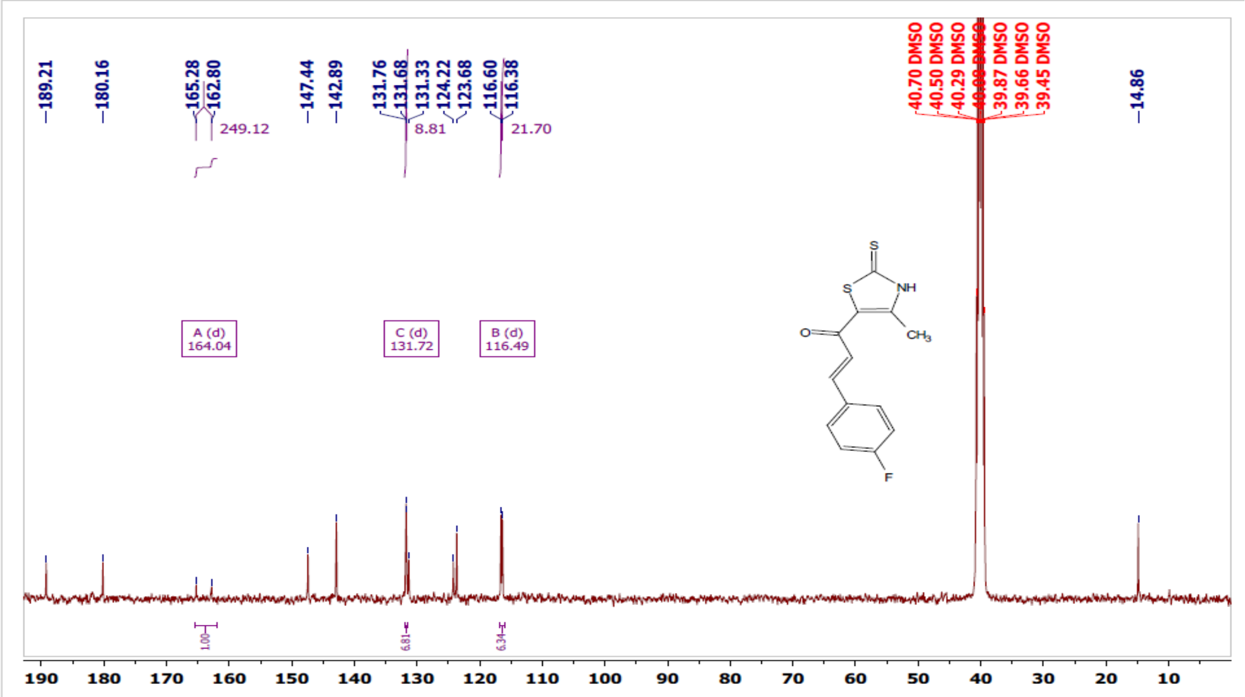

Figure S8: ^{13}C NMR spectrum of compound **2c**.

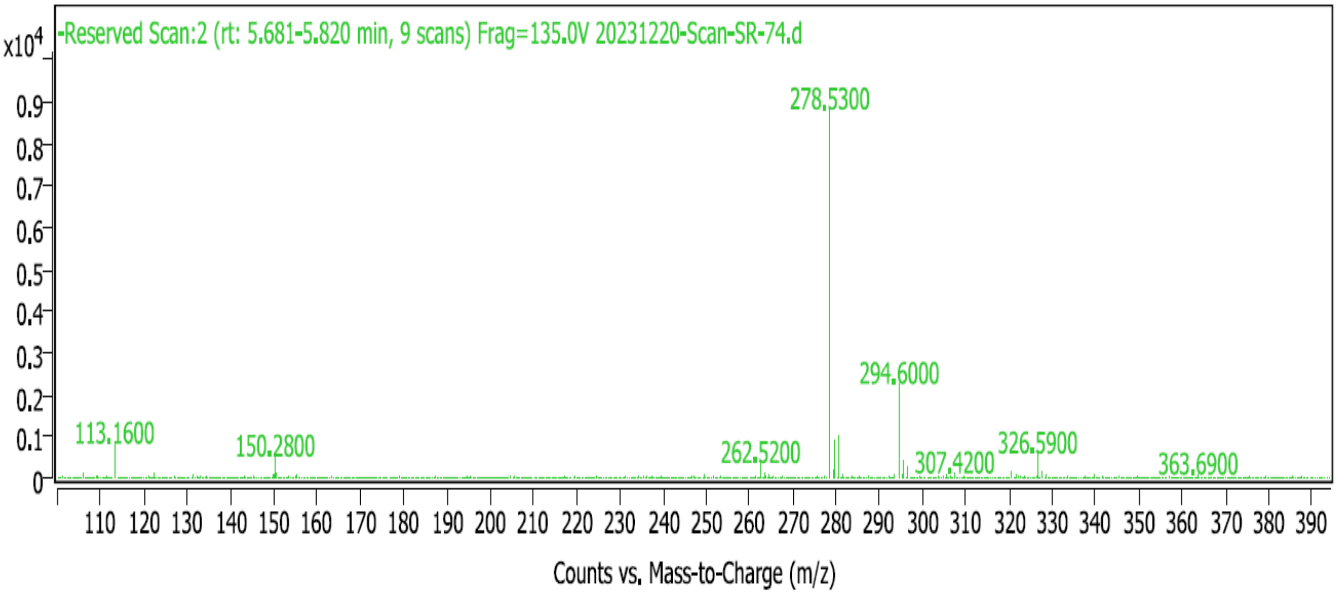

Figure S9: Mass spectrum of compound **2c**.

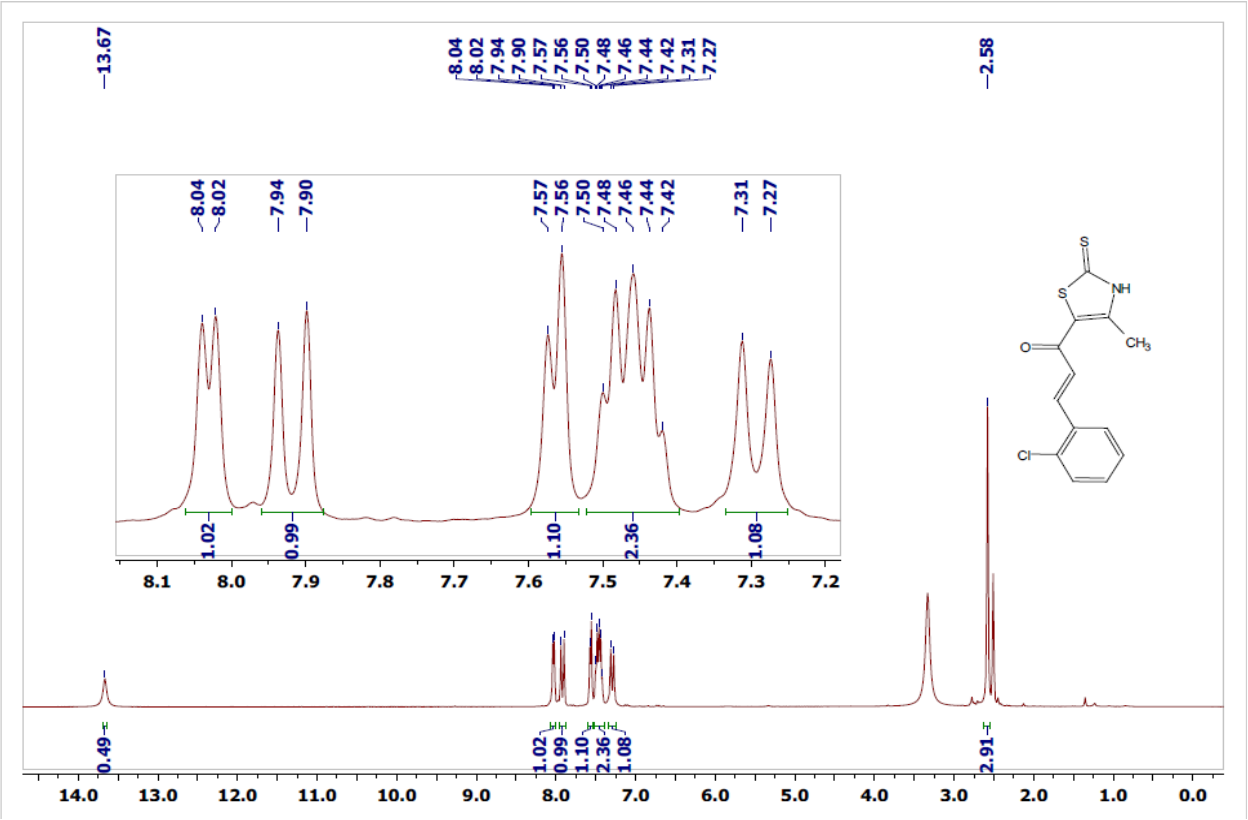

Figure S10: ^{1}H NMR spectrum of compound **2d**.

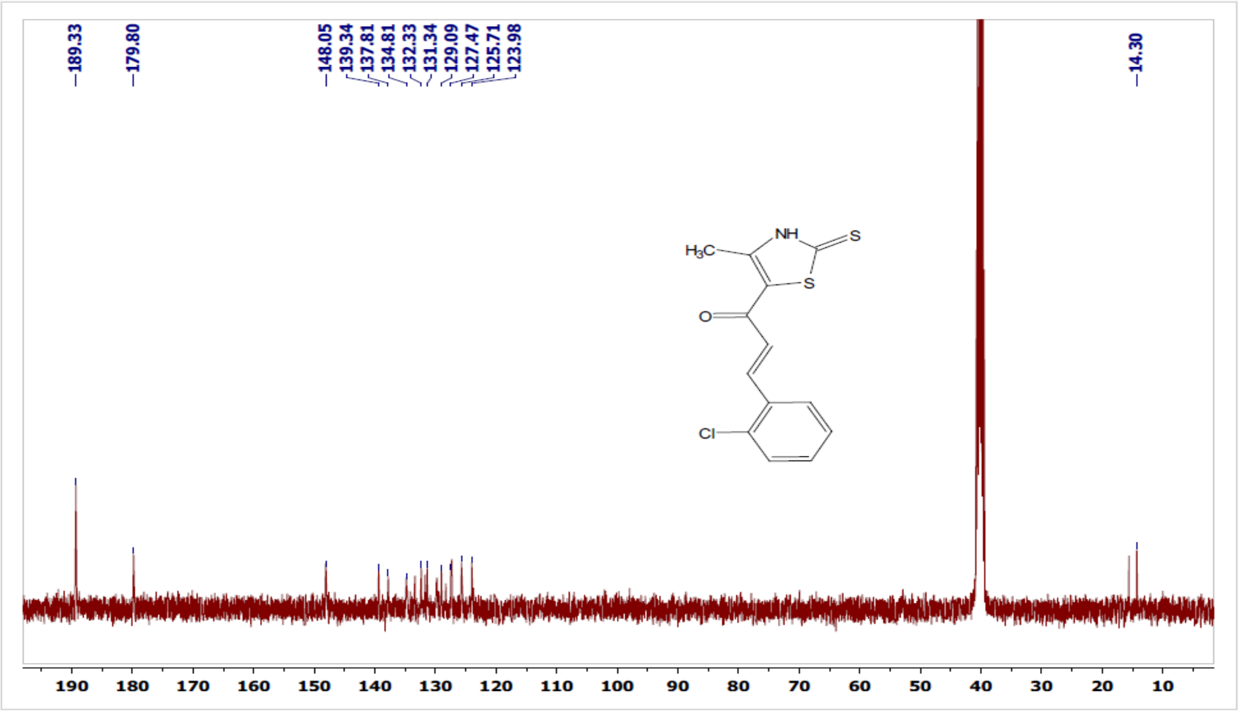

Figure S11: ^{13}C NMR spectrum of compound **2d**.

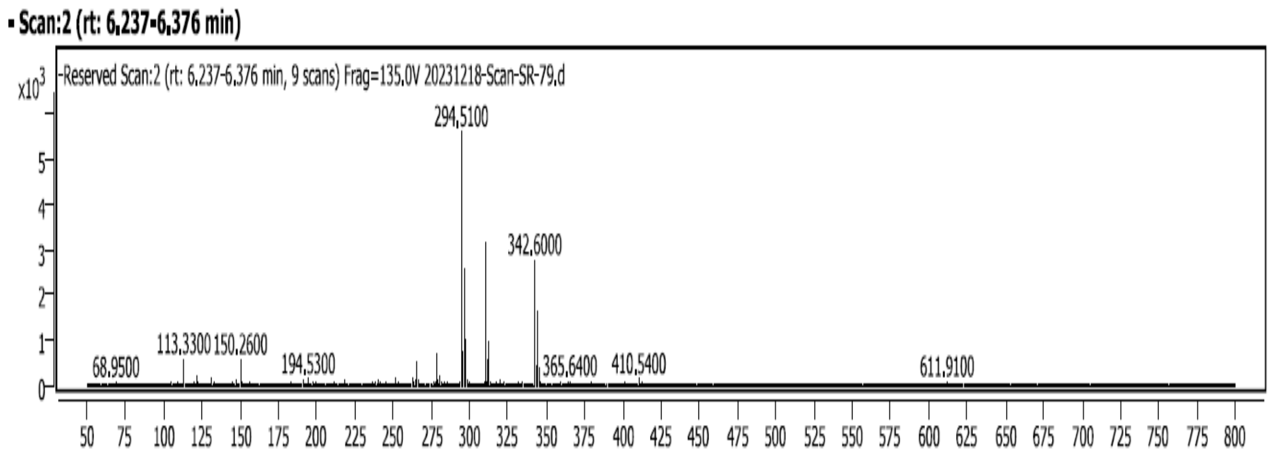

Figure S12: Mass spectrum of compound **2d**.

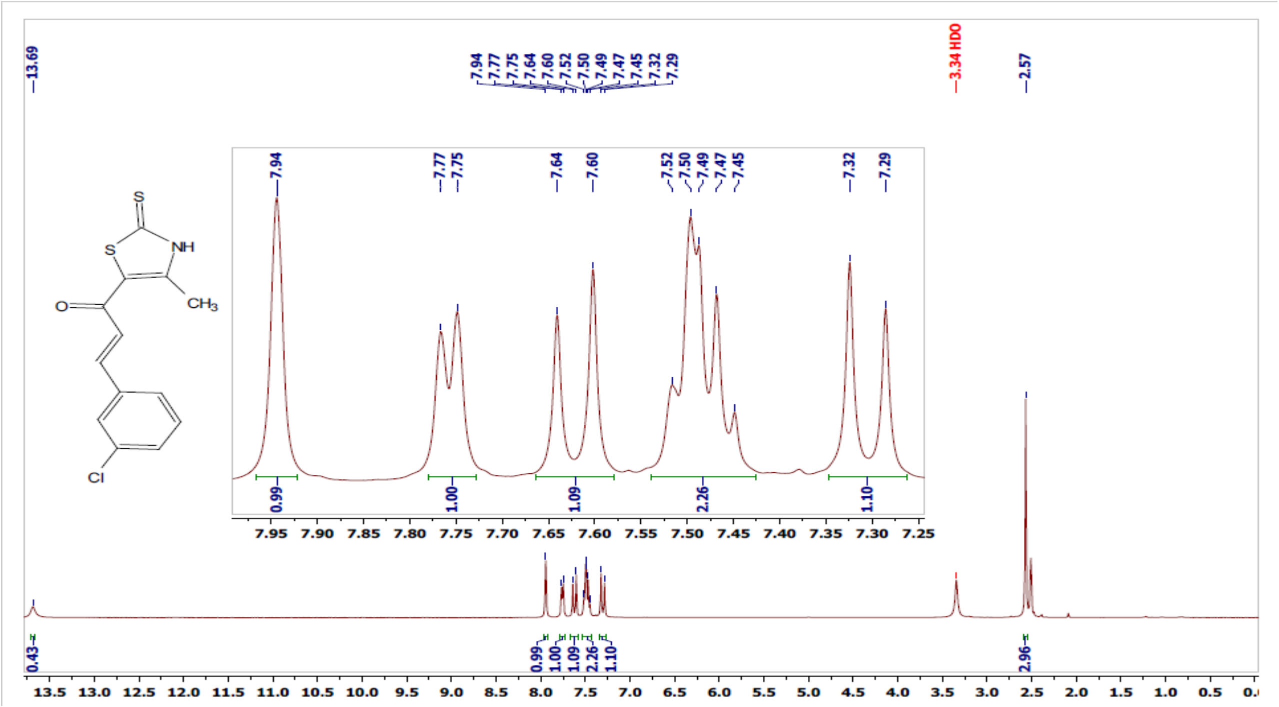

Figure S13: ^{1}H NMR spectrum of compound **2e**.

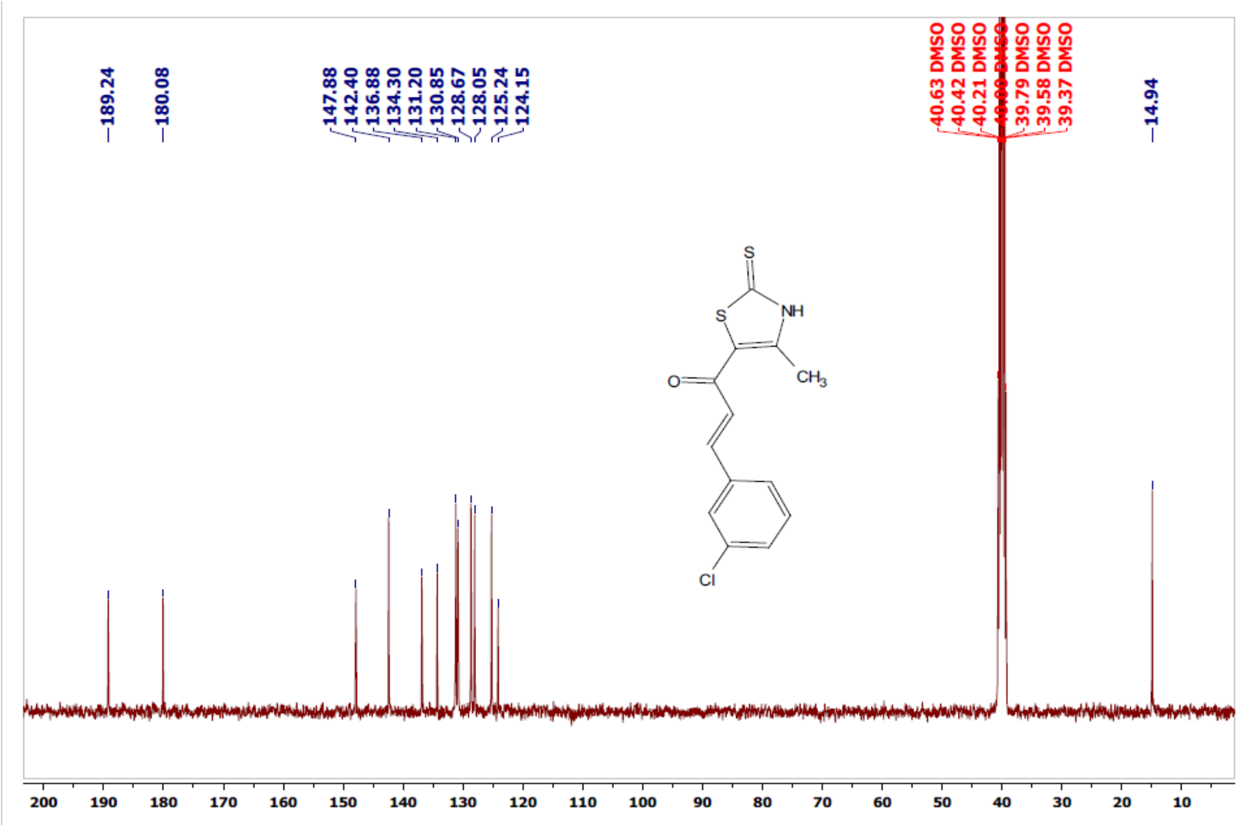

Figure S14: ^{13}C NMR spectrum of compound **2e**.

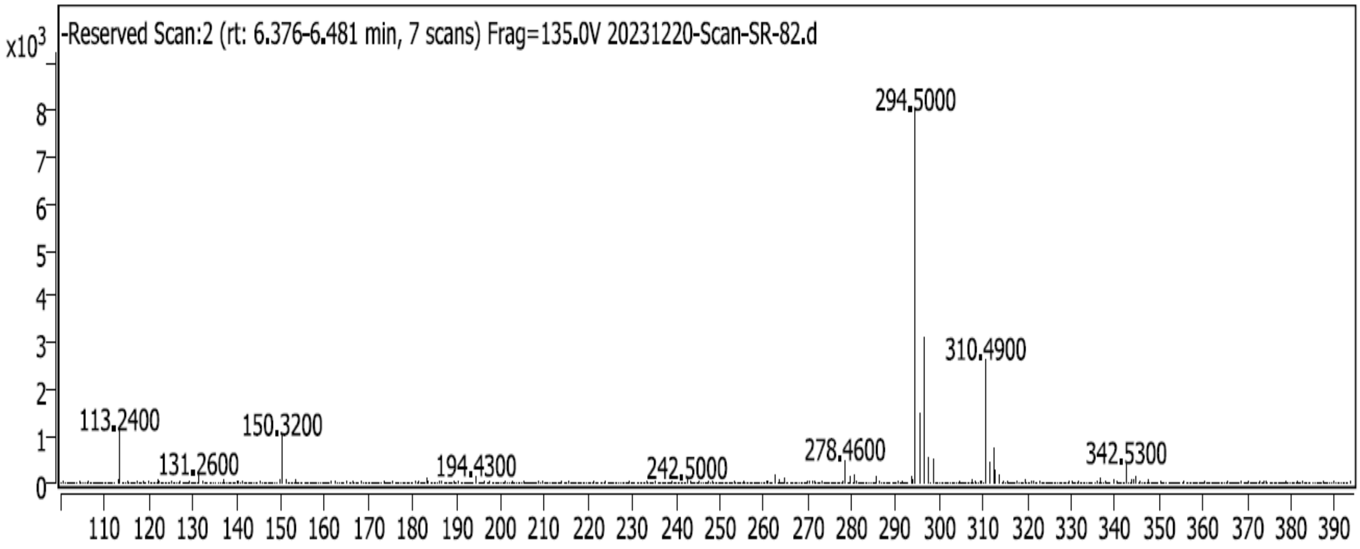

Figure S15: Mass spectrum of compound **2e**.

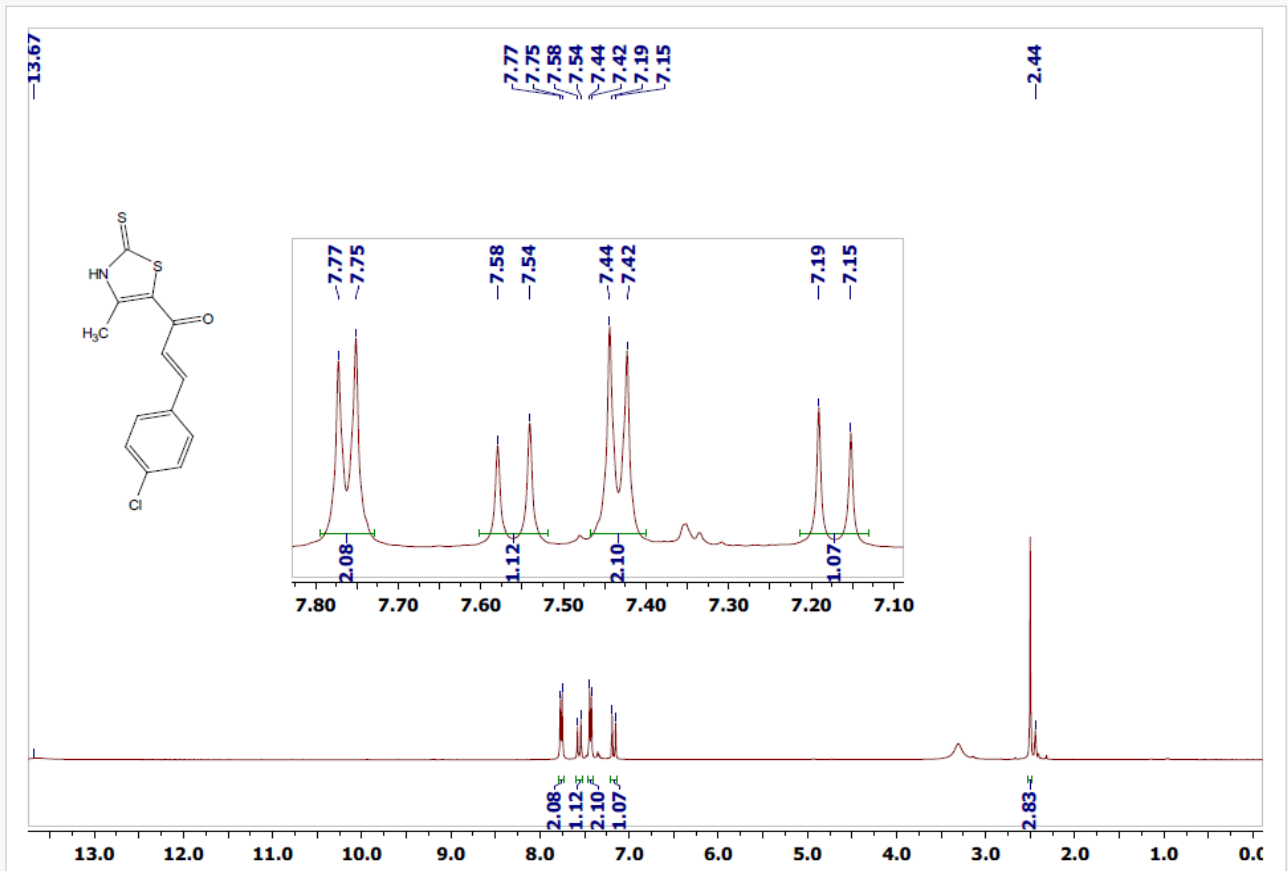

Figure S16: ^{1}H NMR spectrum of compound **2f**.

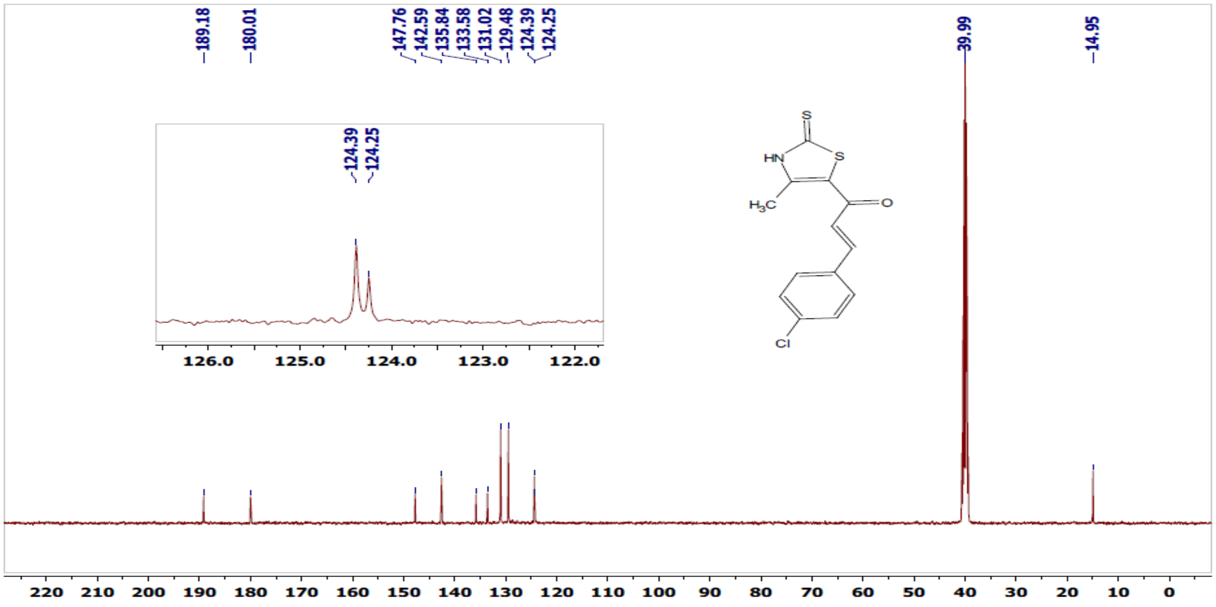

Figure S17: ^{13}C NMR spectrum of compound **2f.**

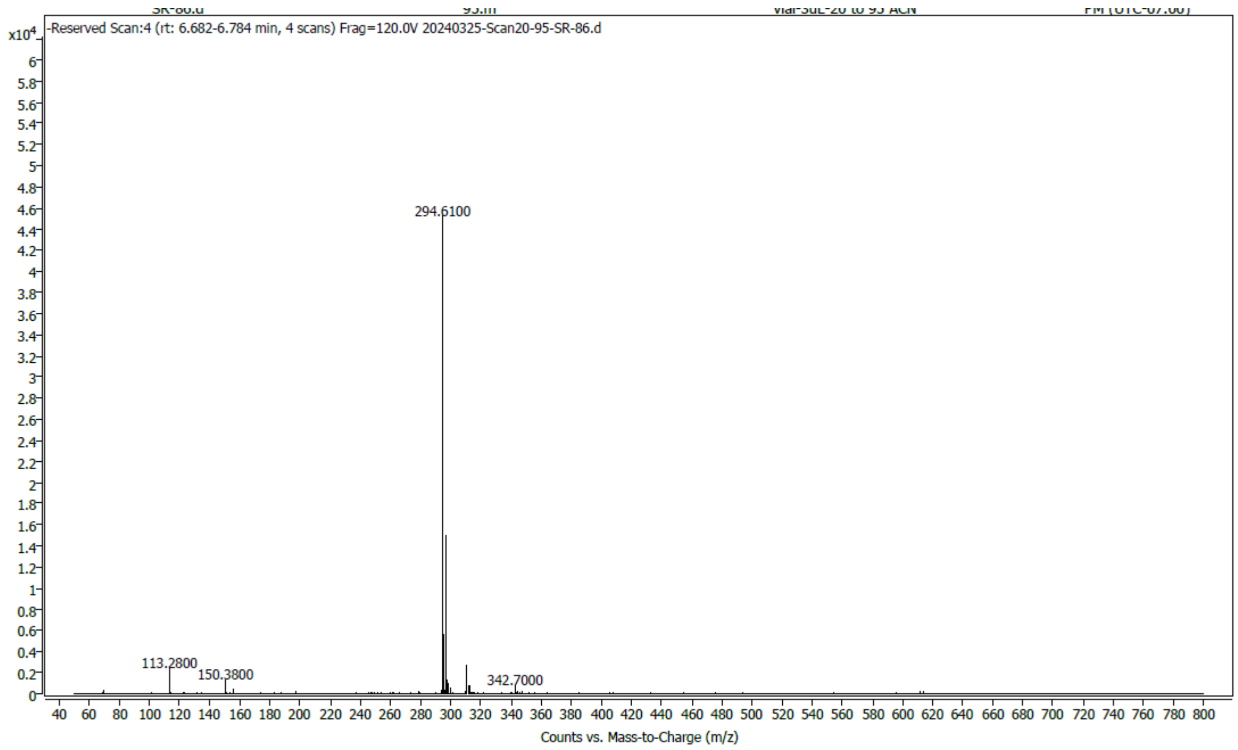

Figure S18: Mass spectrum of compound **2f**.

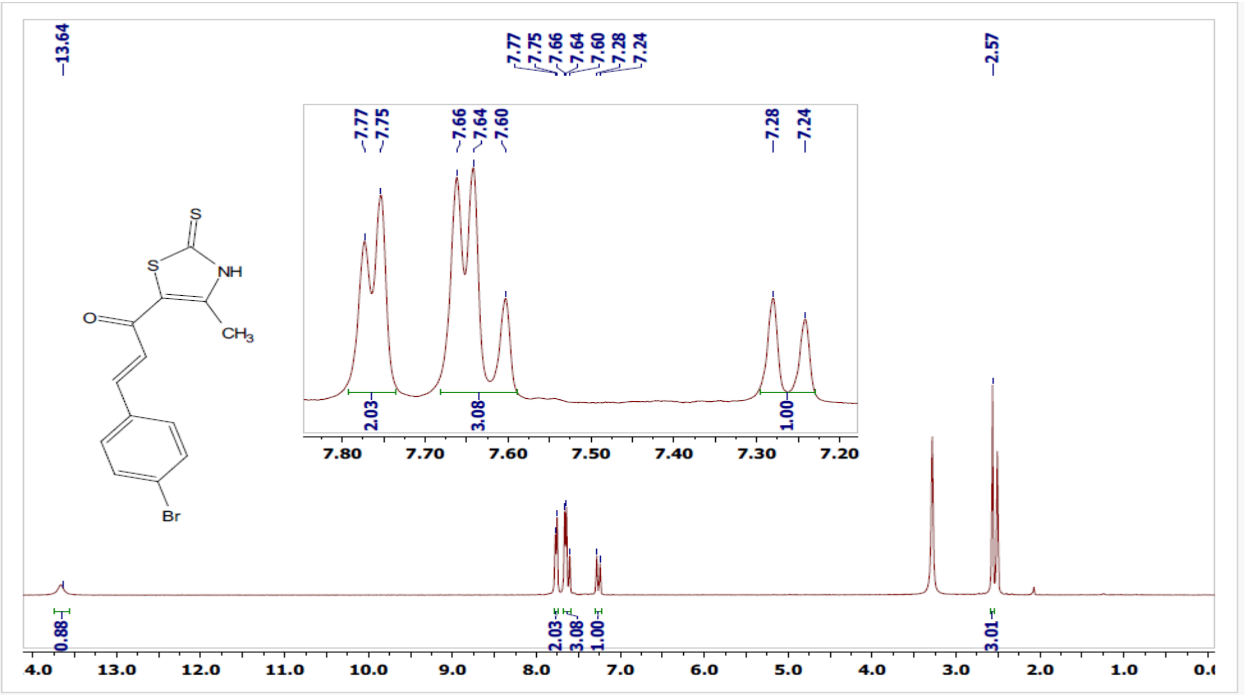

Figure S19: ^{1}H NMR spectrum of compound **2g**.

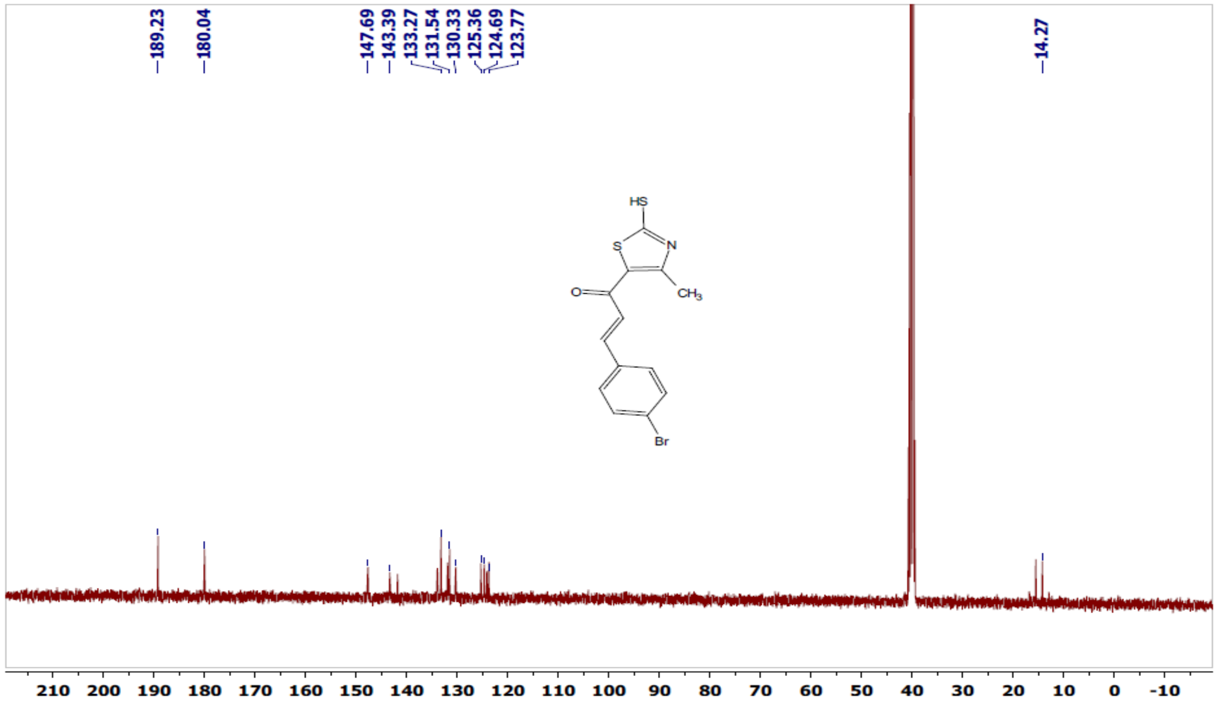

Figure S20: [13] C NMR spectrum of compound **2g**.

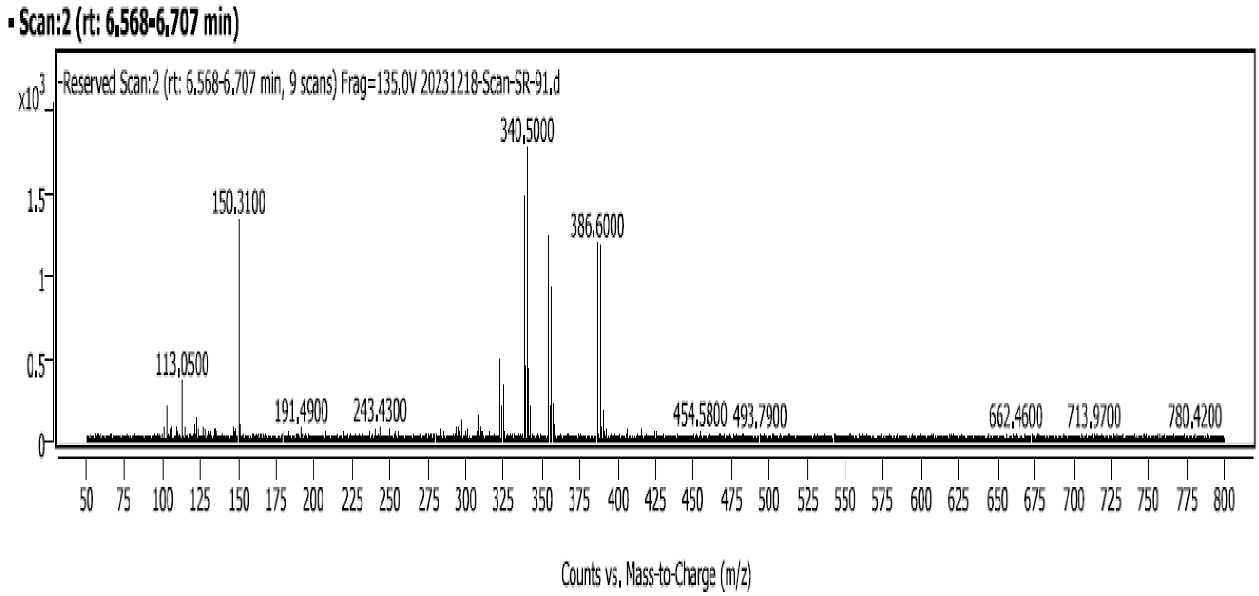

Figure S21: Mass spectrum of compound **2g**.

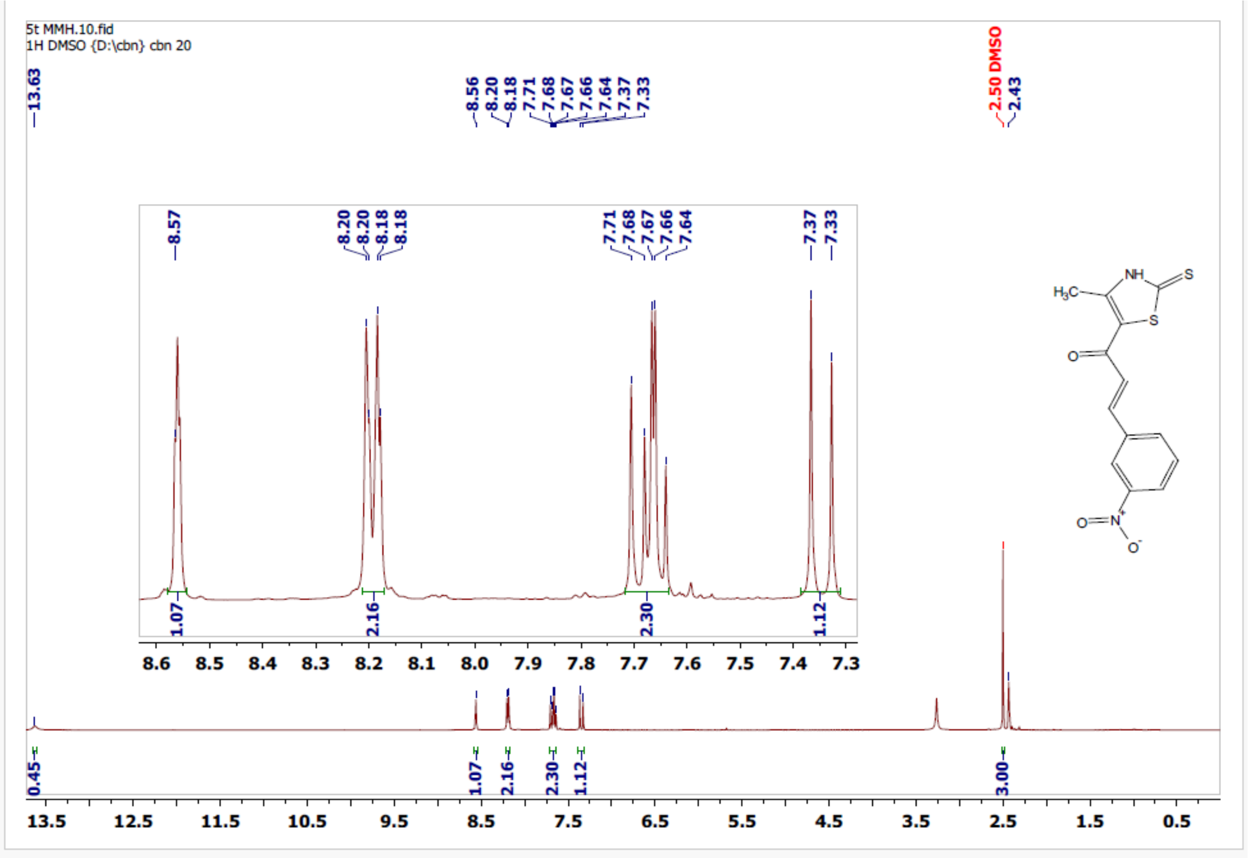

Figure S22: ^{1}H NMR spectrum of compound **2h**.

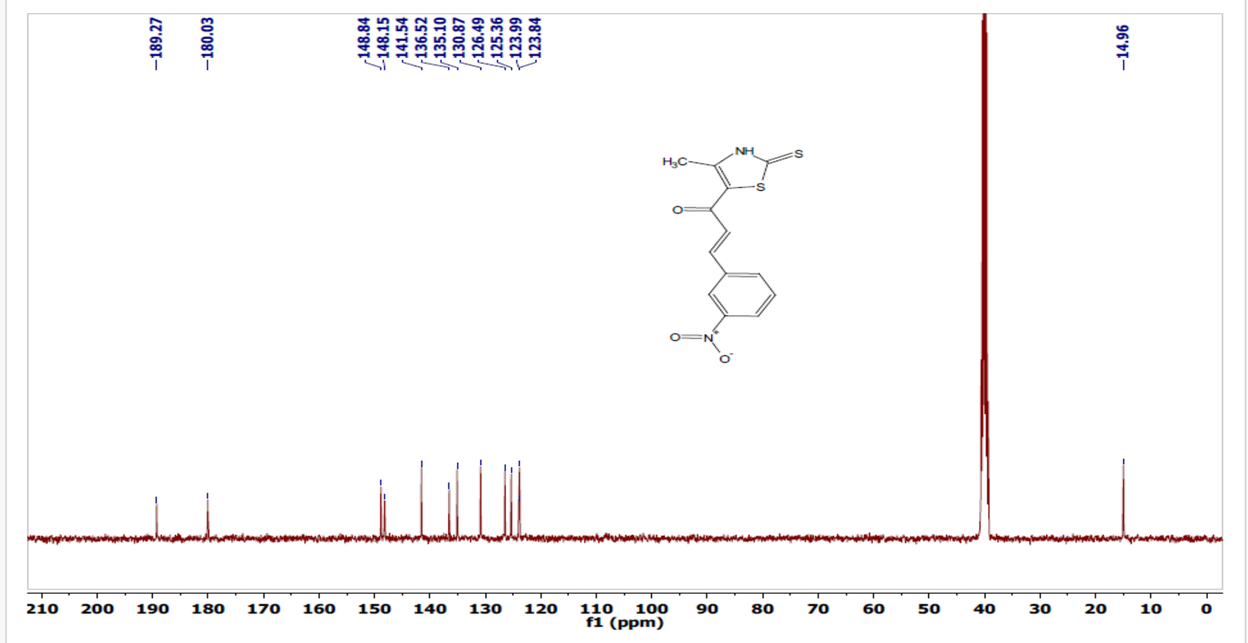

Figure S23: [13] C NMR spectrum of compound **2h**.

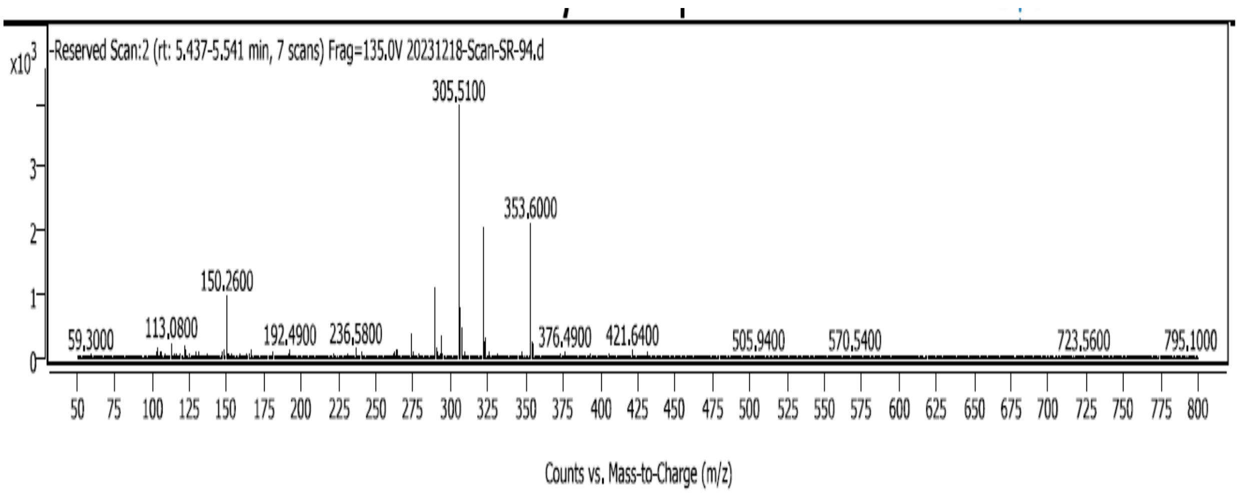

Figure S24: Mass spectrum of compound **2h**.

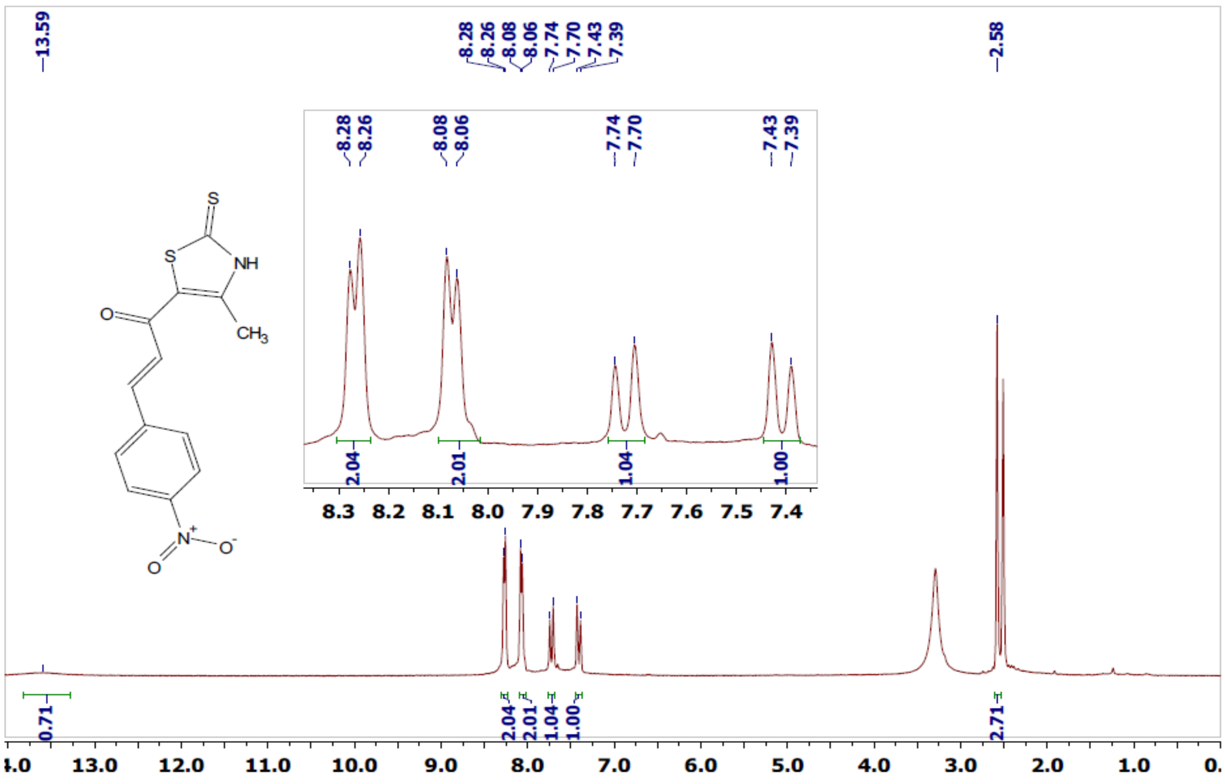

Figure S25: ^{1}H NMR spectrum of compound **2i**.

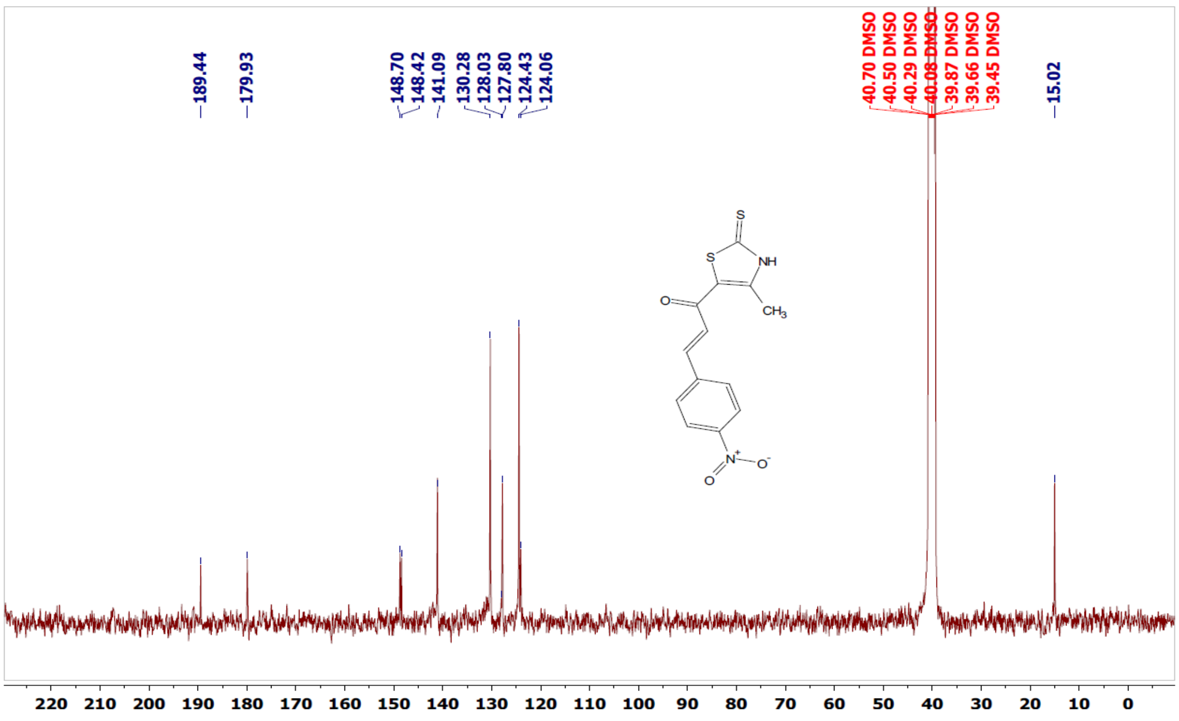

Figure S26: [13] C NMR spectrum of compound **2i**.

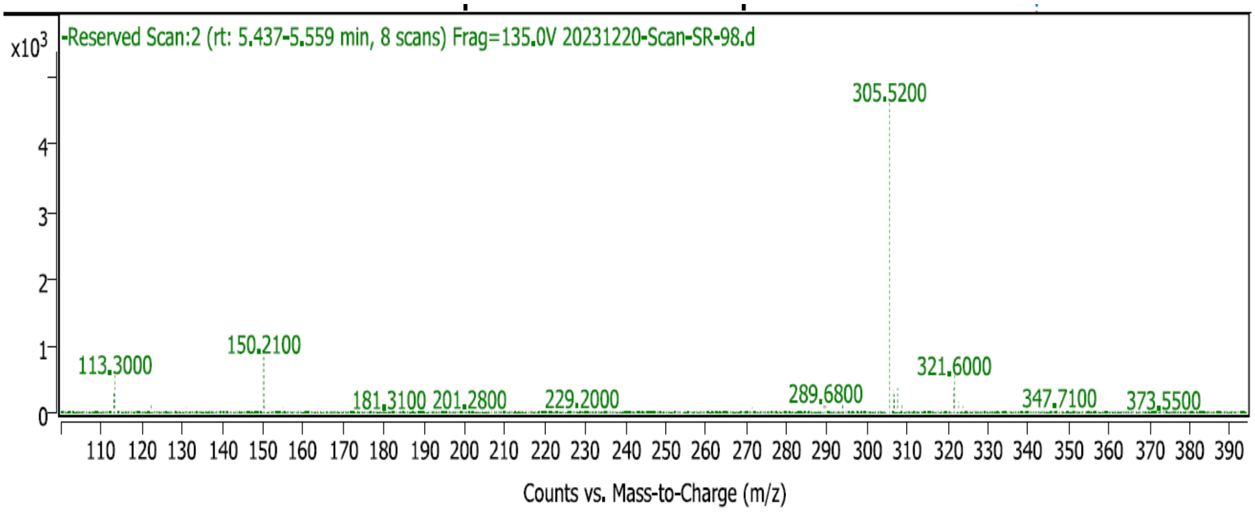

Figure S27: Mass spectrum of compound **2i**.

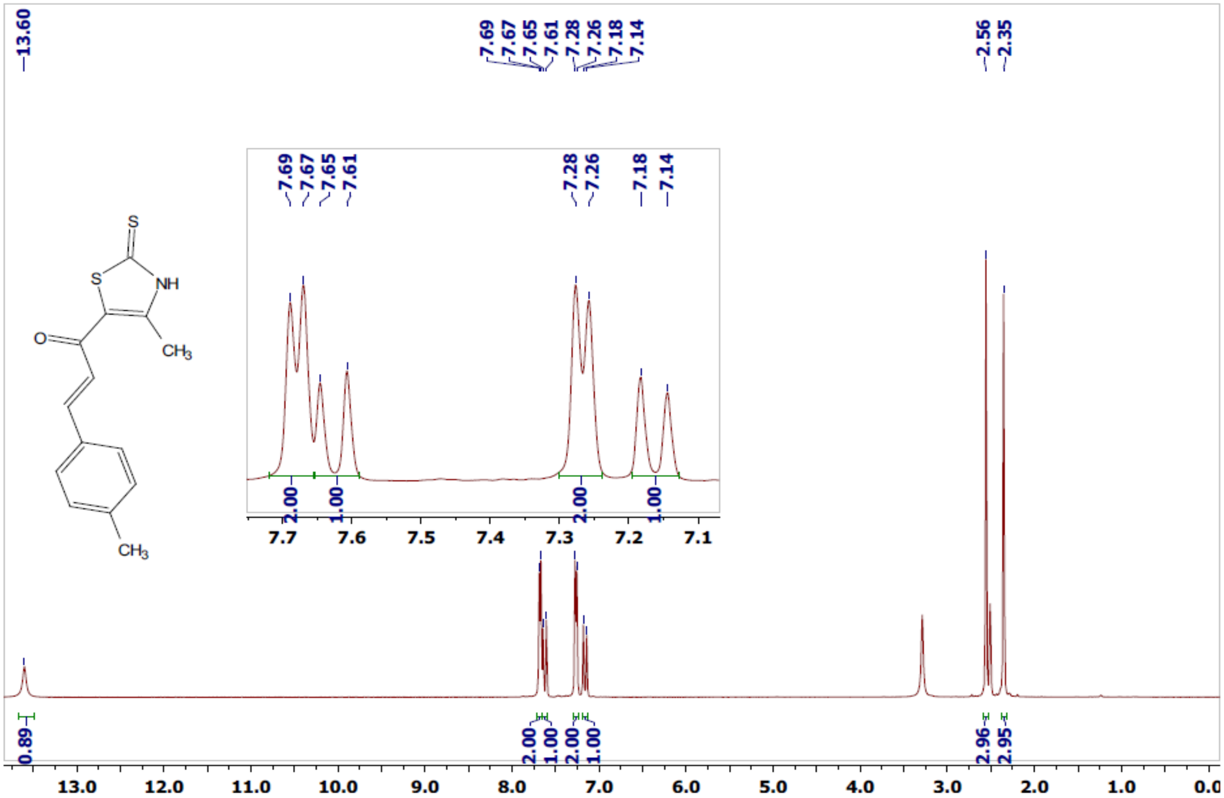

Figure S28: ^{1}H NMR spectrum of compound **2j**.

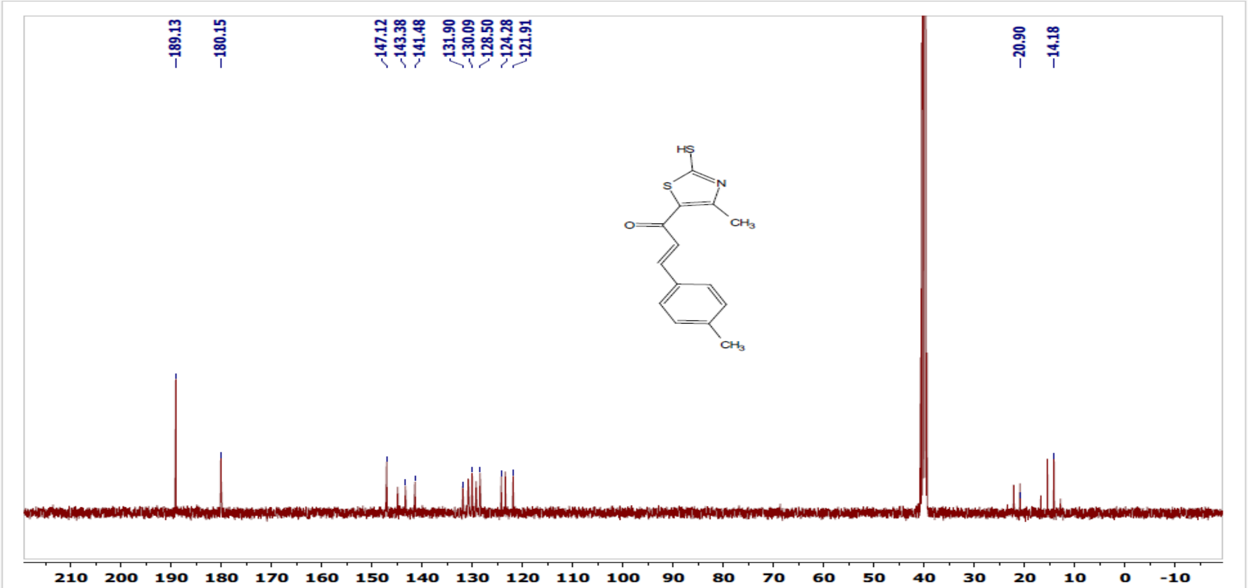

Figure S29: ^{1}H NMR spectrum of compound **2j**.

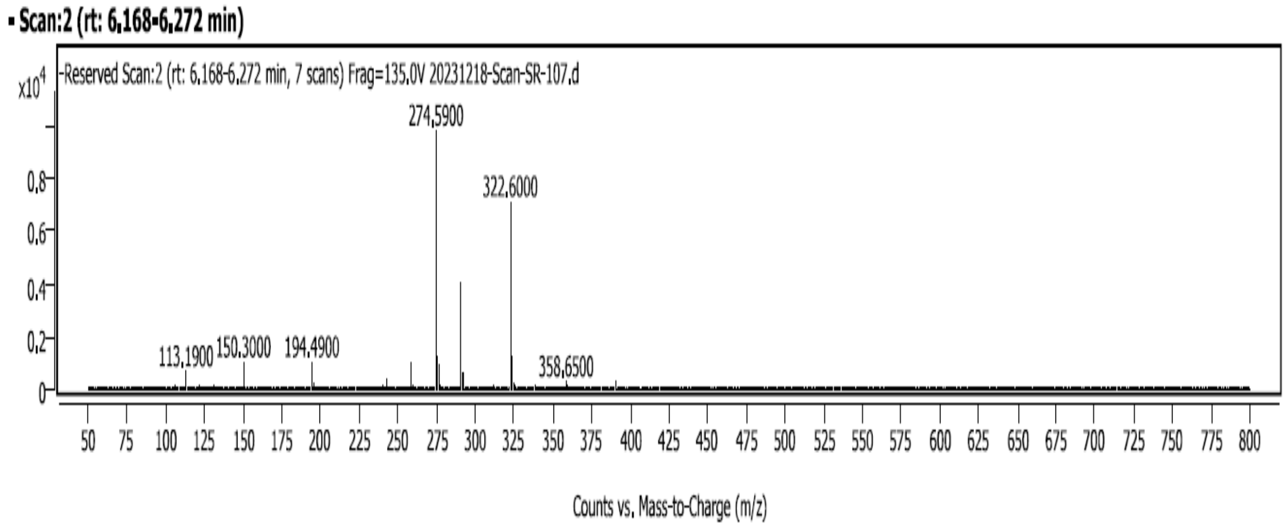

Figure S30: Mass spectrum of compound **j**.

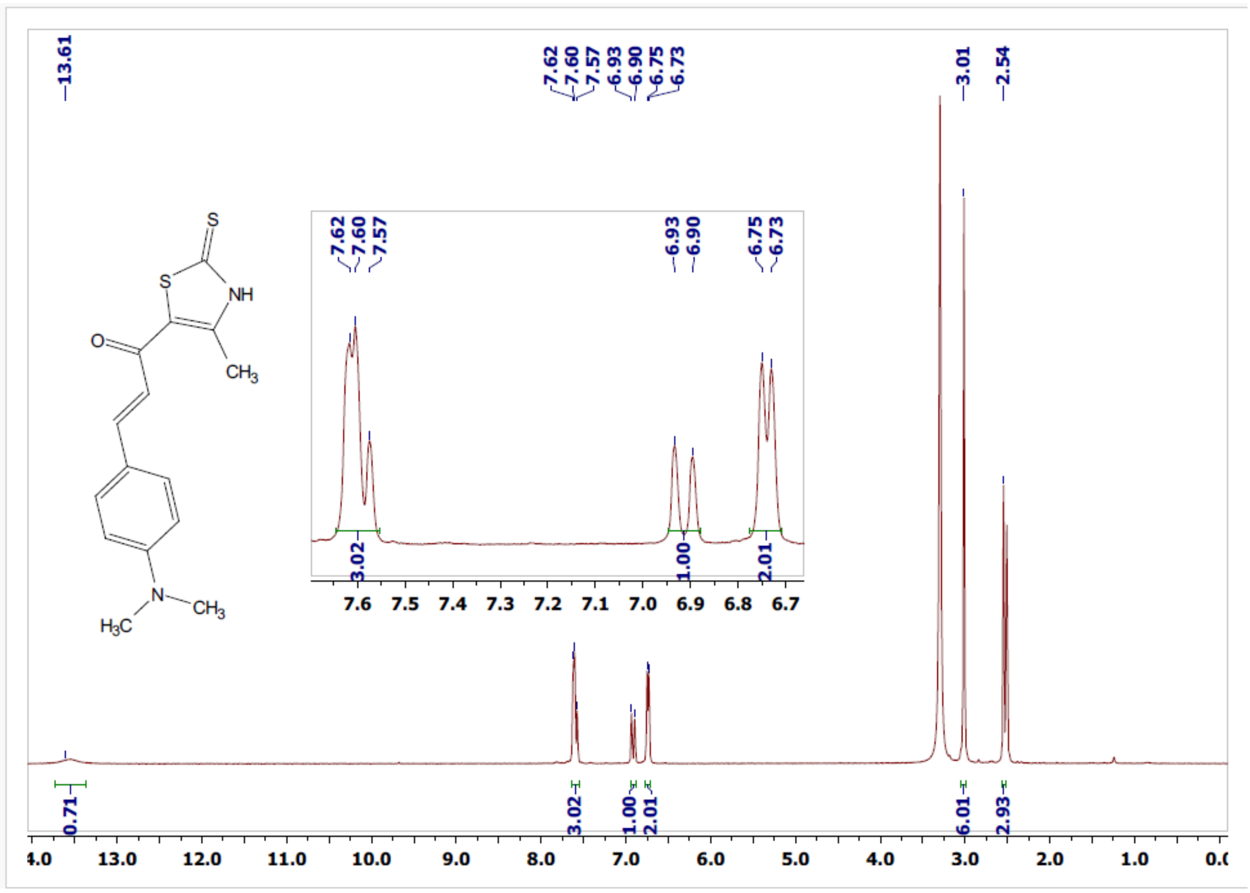

Figure S31: ^{1}H NMR spectrum of compound **2k**.

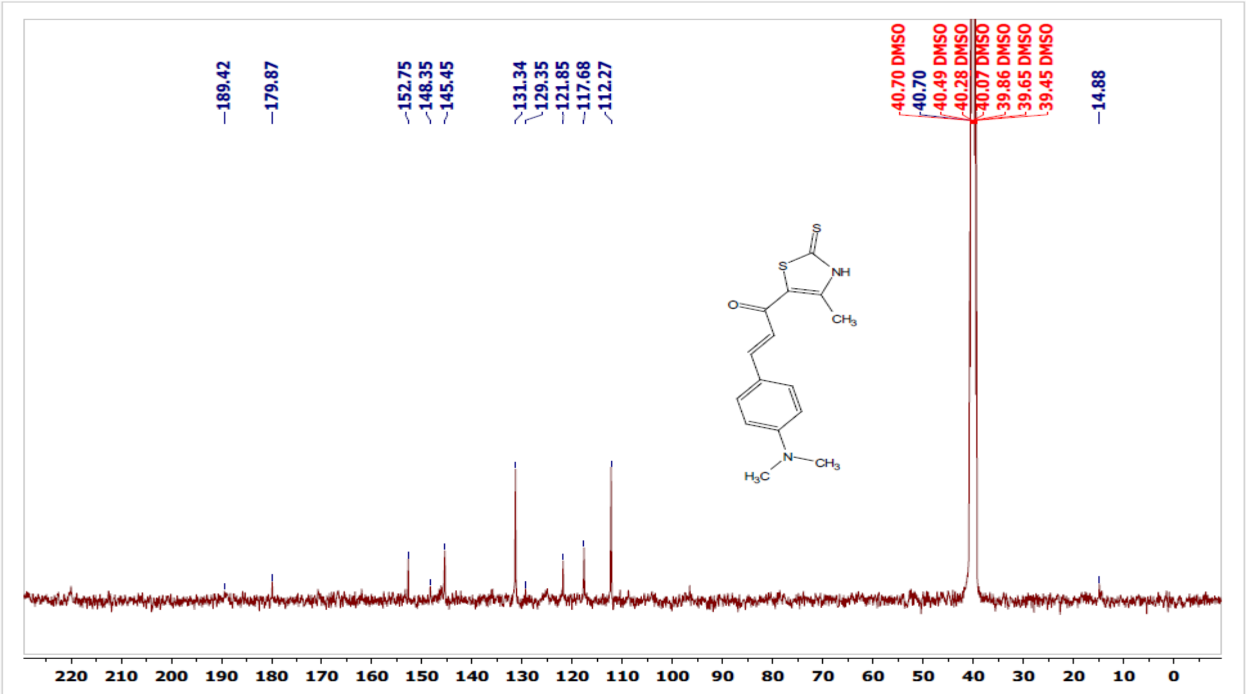

Figure S32: ^{13}C NMR spectrum of compound **2k**.

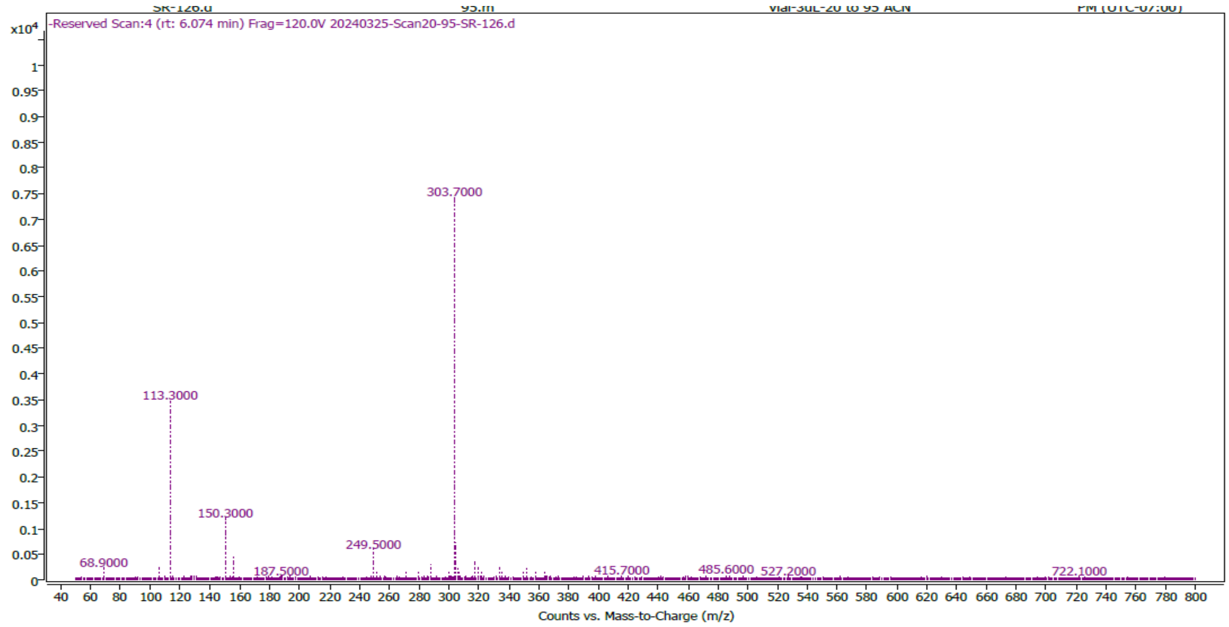

Figure S33: Mass spectrum of compound **2k**.

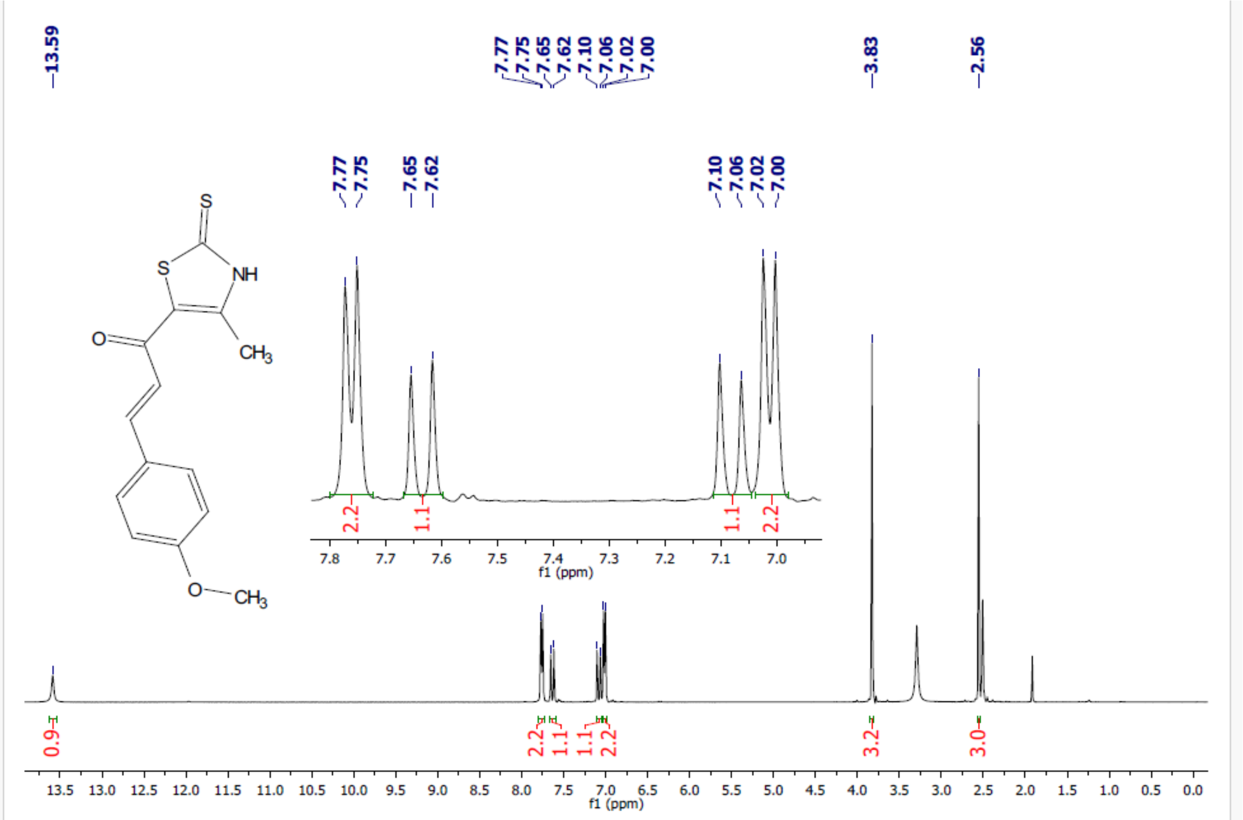

Figure S34: ^{1}H NMR spectrum of compound **2l**.

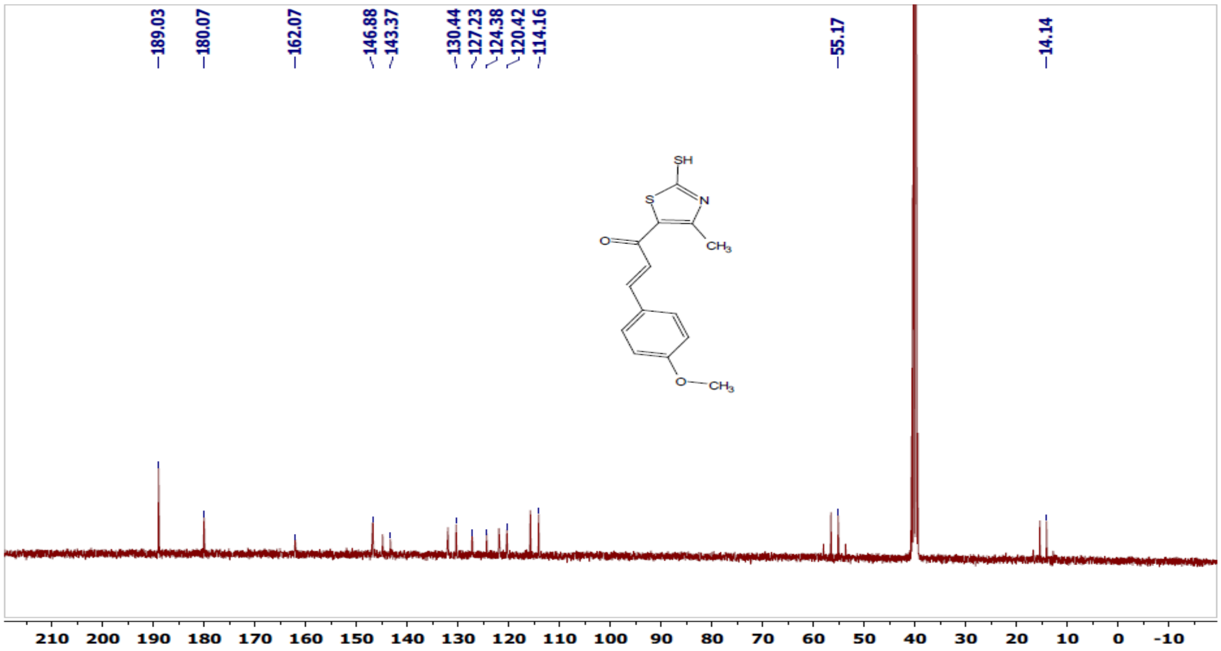

Figure S35: ^{13}C NMR spectrum of compound **2l**.

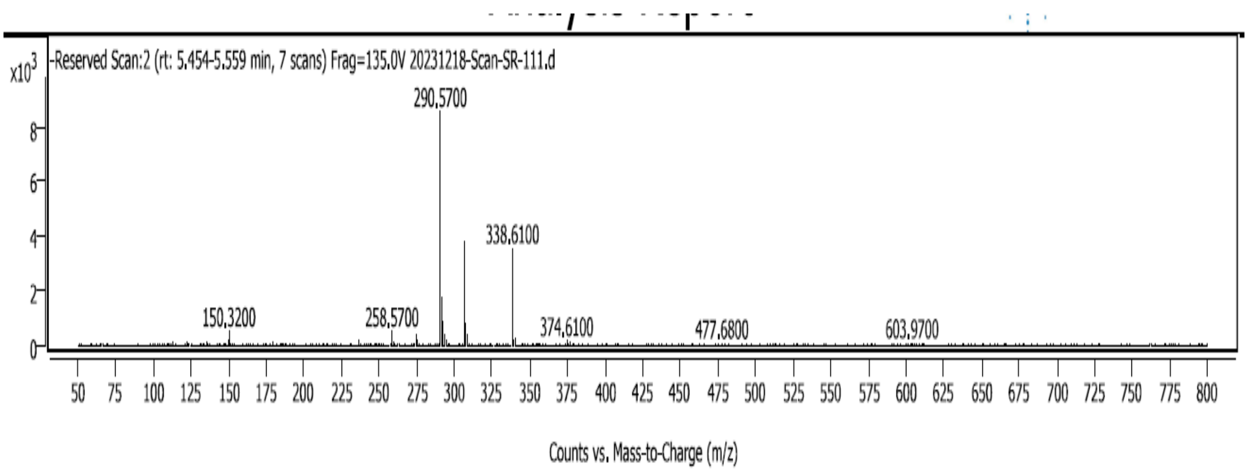

Figure S36: Mass spectrum of compound **2l**.

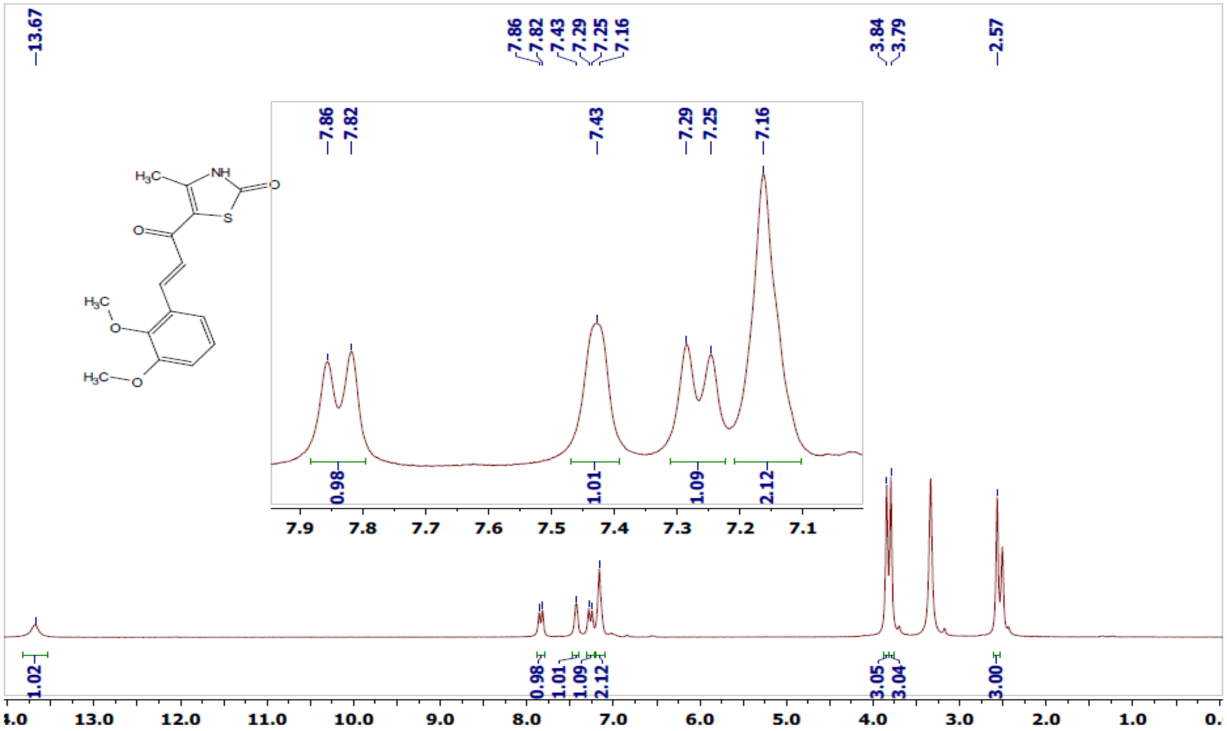

Figure S37: ^{1}H NMR spectrum of compound **2m**.

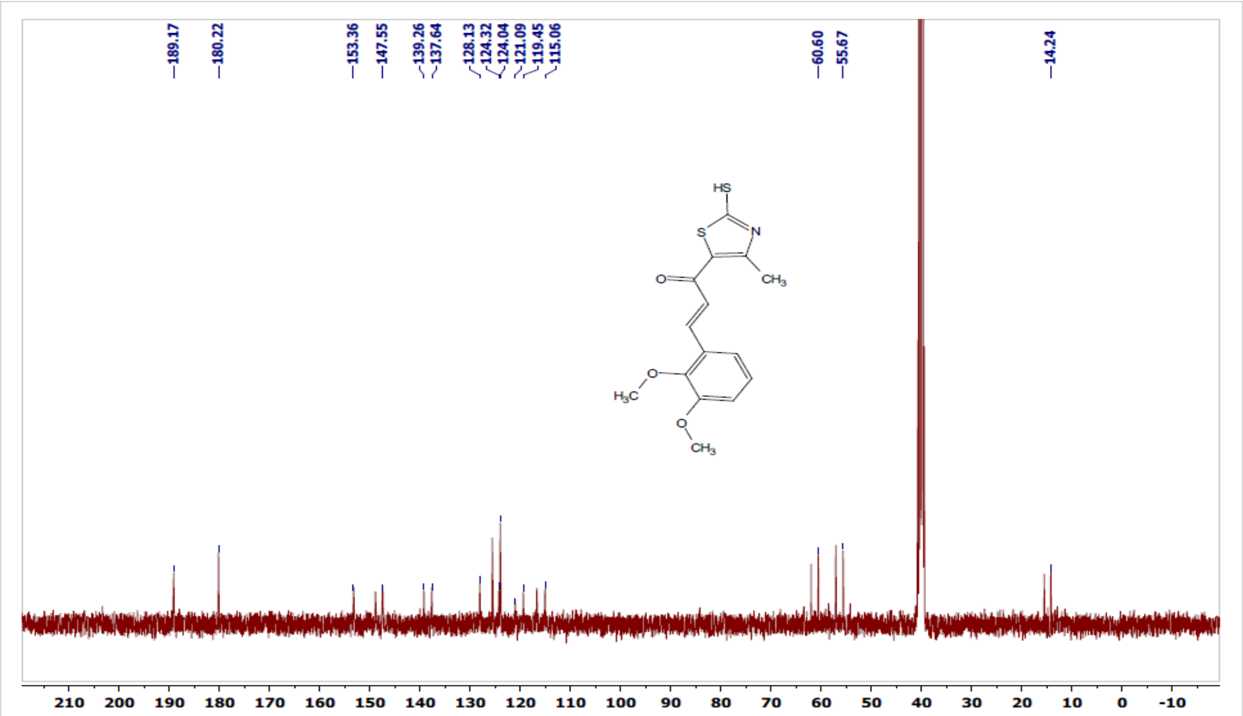

Figure S38: ^{13}C NMR spectrum of compound **2m**.

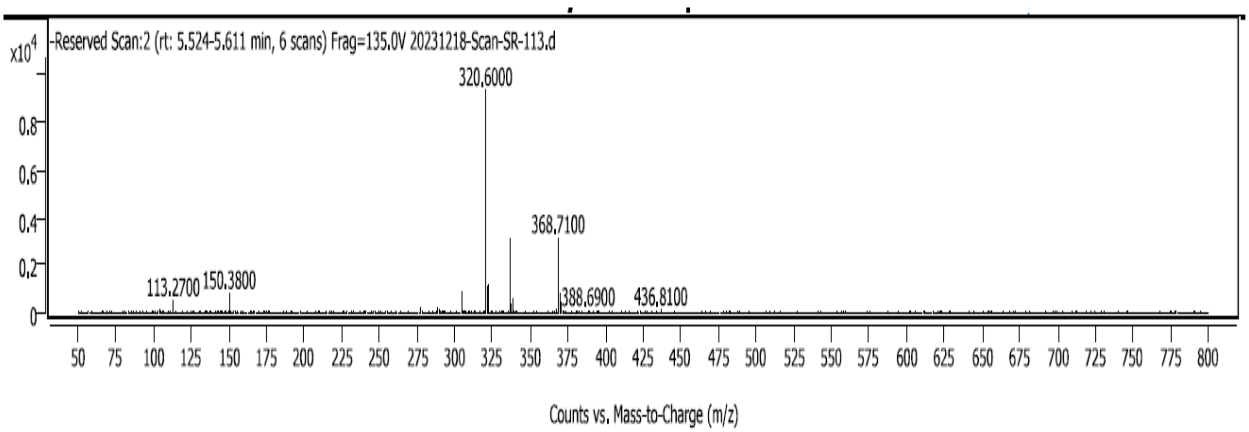

Figure S39: Mass spectrum of compound **2m**.

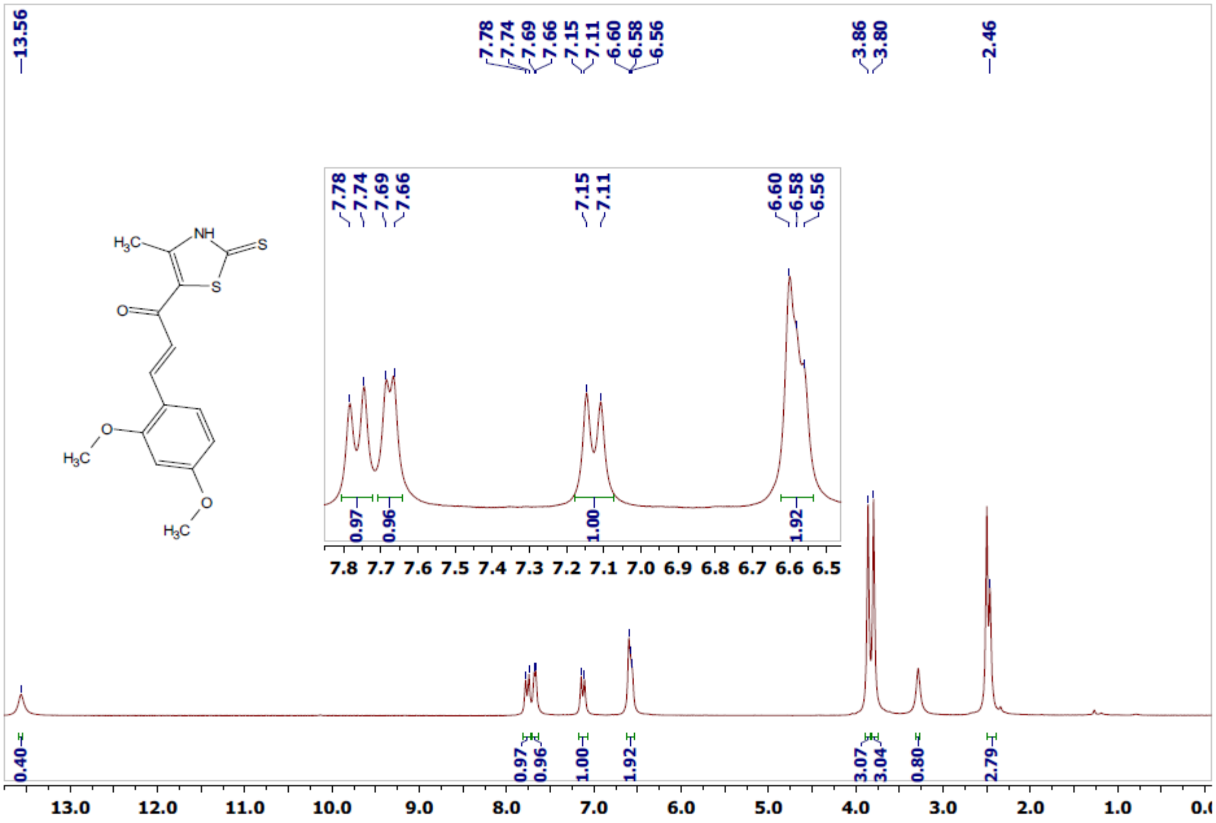

Figure S40: ^{1}H NMR spectrum of compound **2n**.

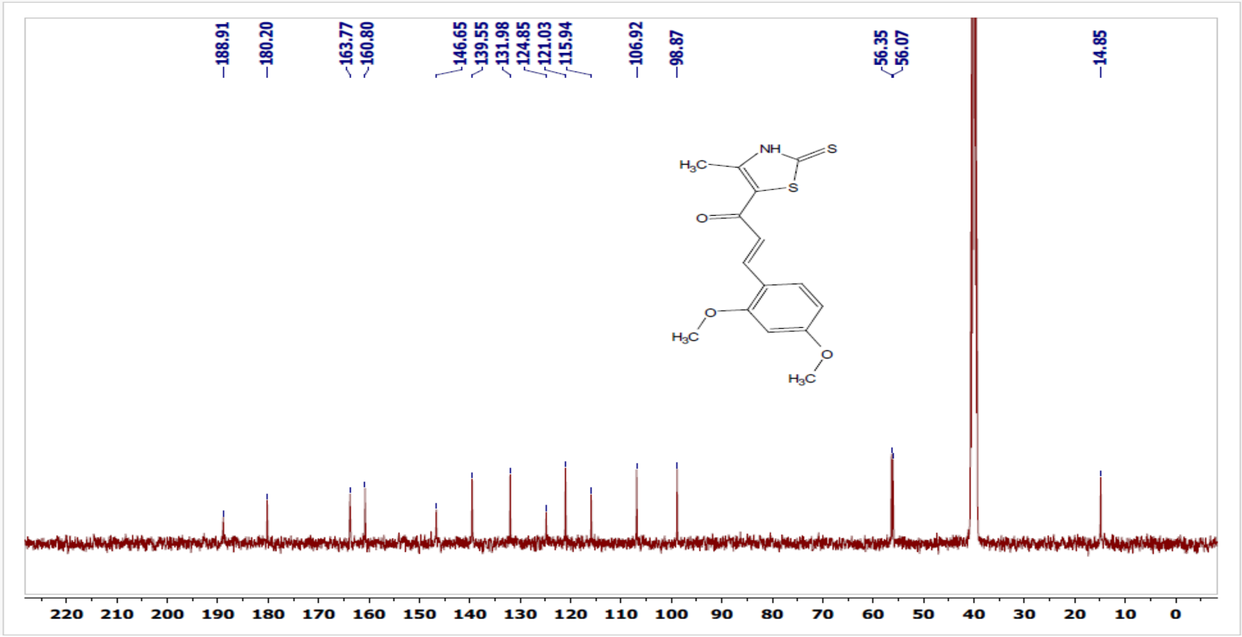

Figure S41: ^{13}C NMR spectrum of compound **2n**.

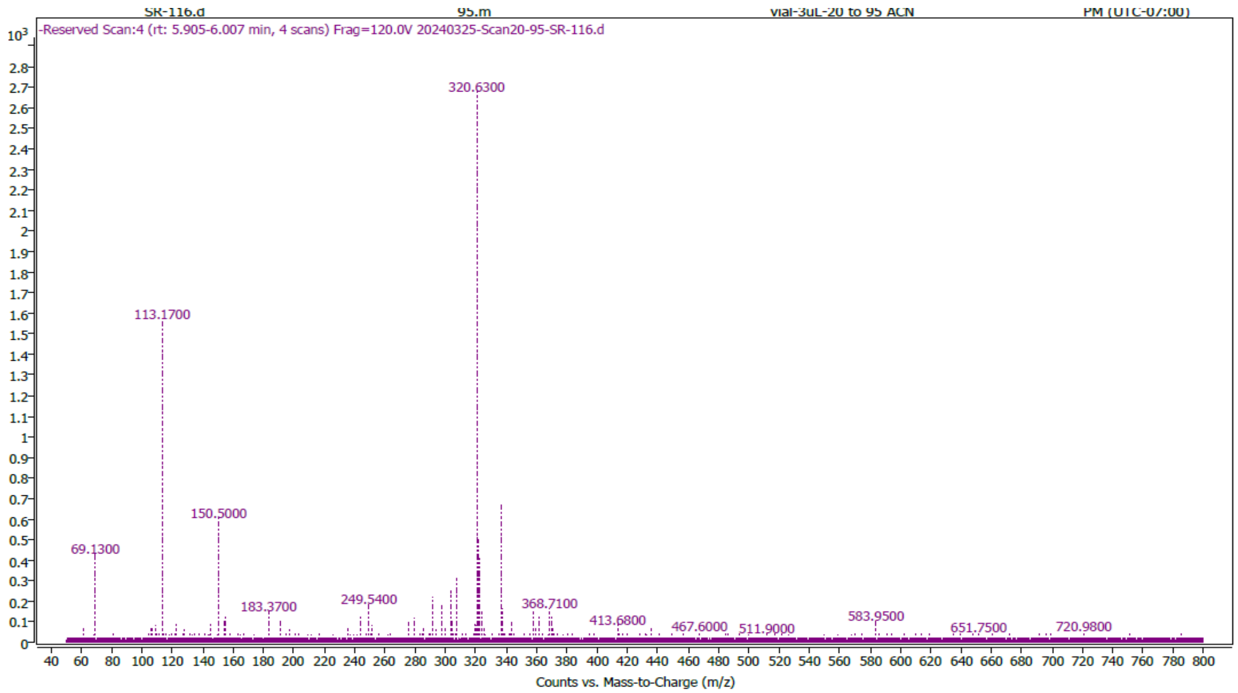

Figure S42: Mass spectrum of compound **2n**.

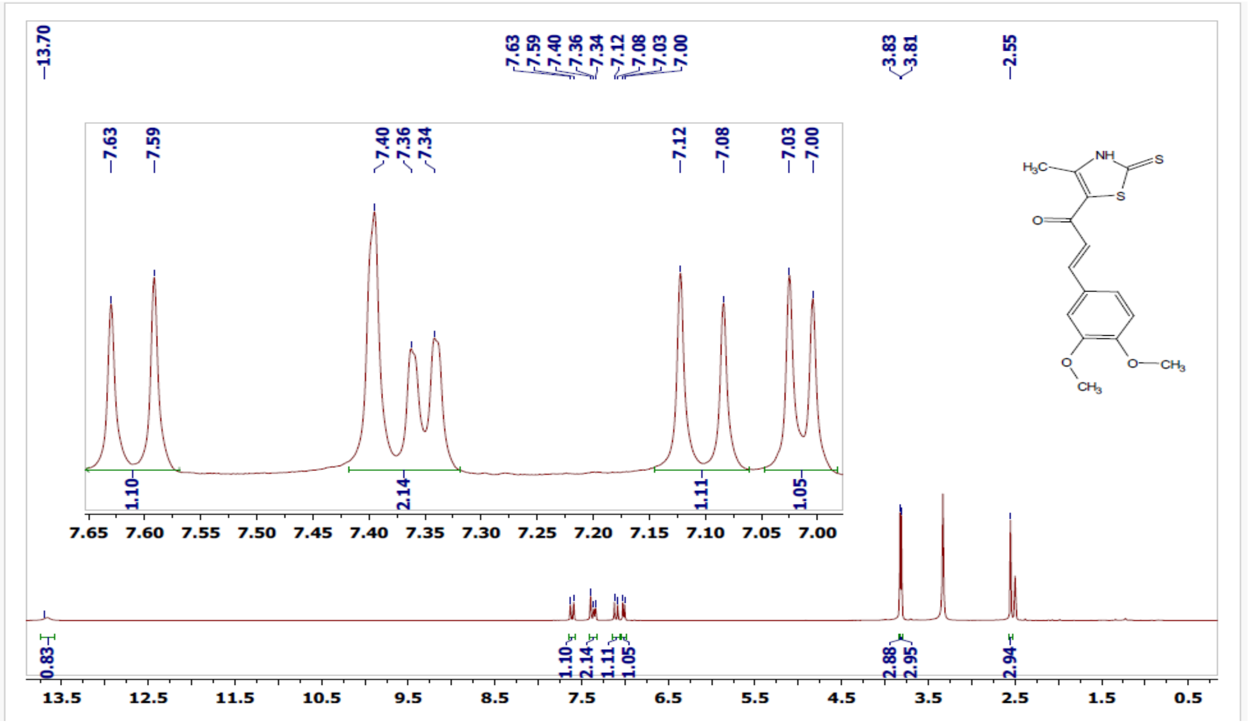

Figure S43: ^{1}H NMR spectrum of compound **2o**.

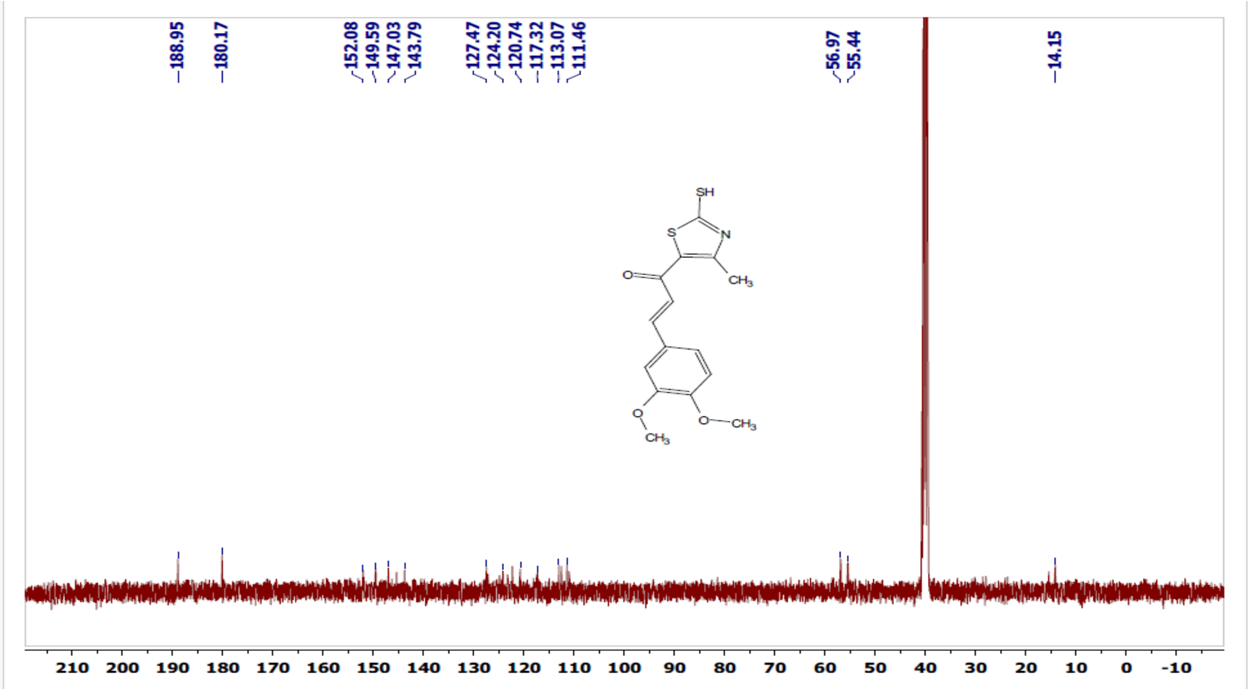

Figure S44: ^{13}C NMR spectrum of compound **2o**.

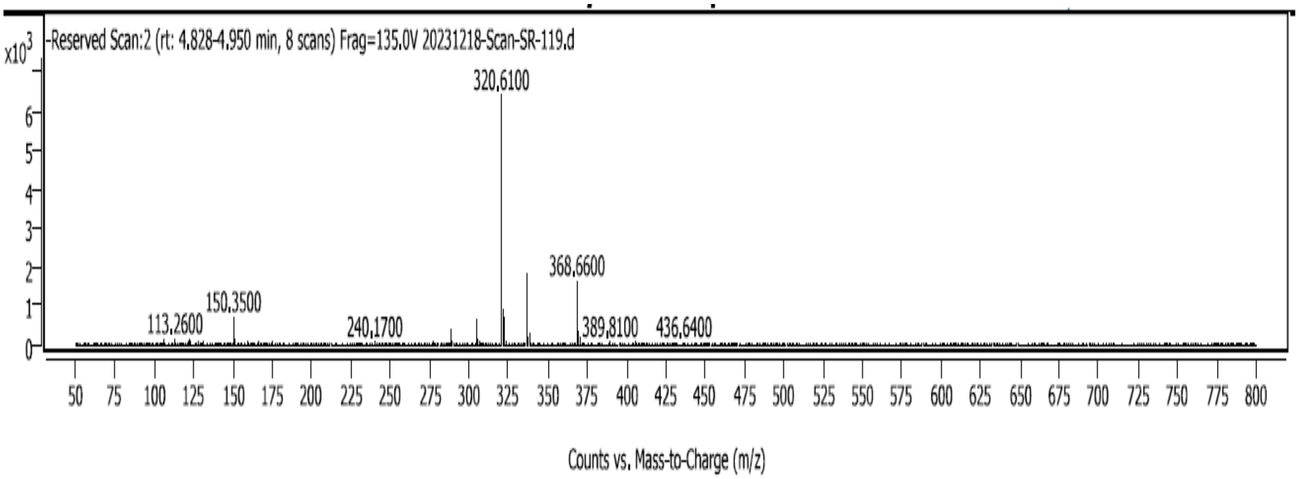

Figure S45: Mass spectrum of compound **2o**.

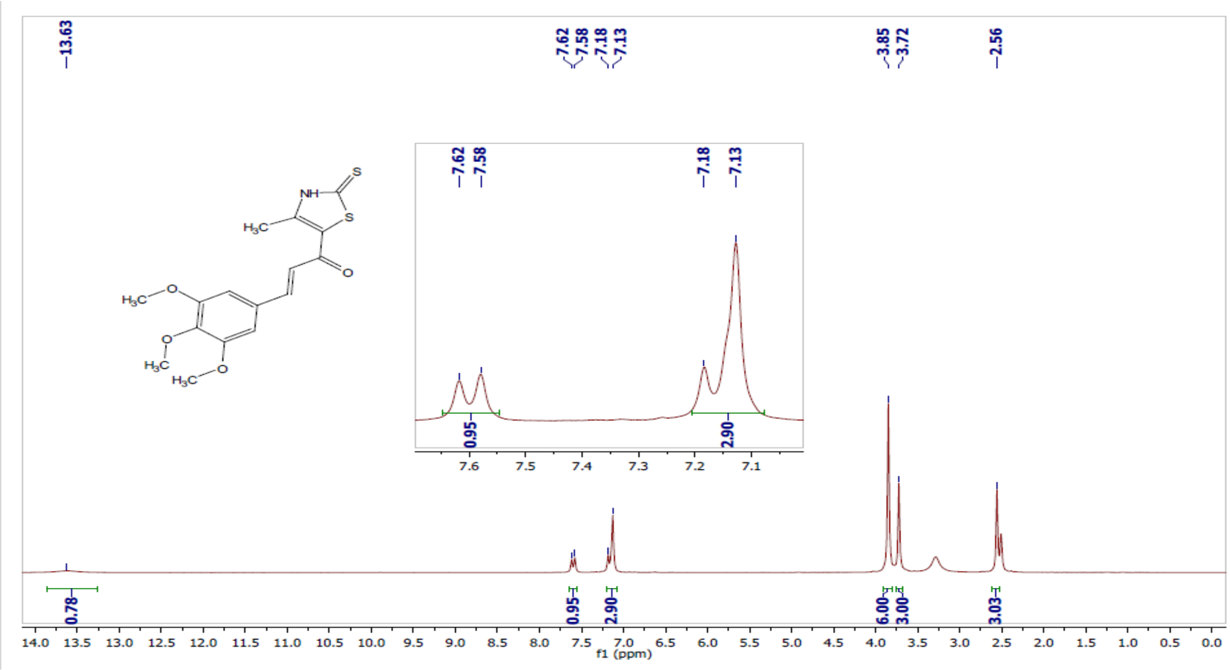

Figure S46: ^{1}H NMR spectrum of compound **2p**.

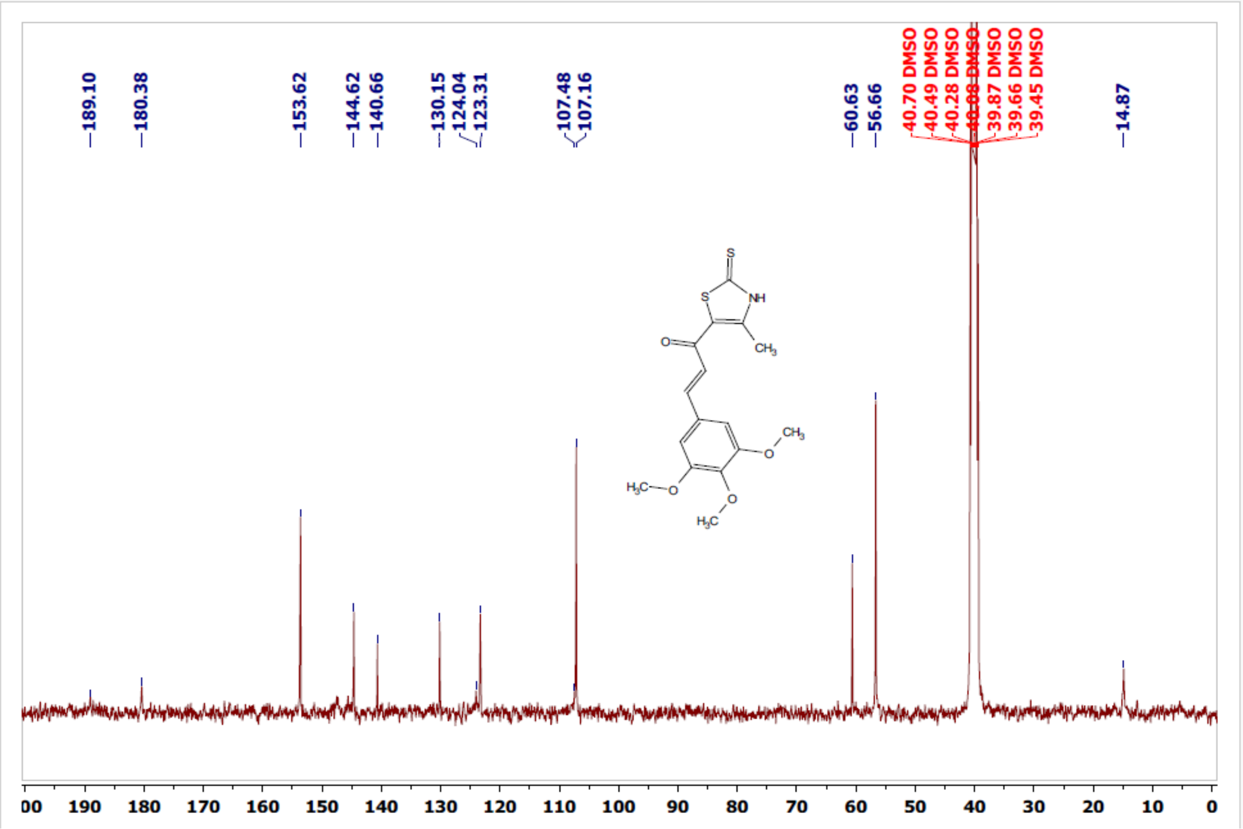

Figure S47: ^{13}C NMR spectrum of compound **2p**.

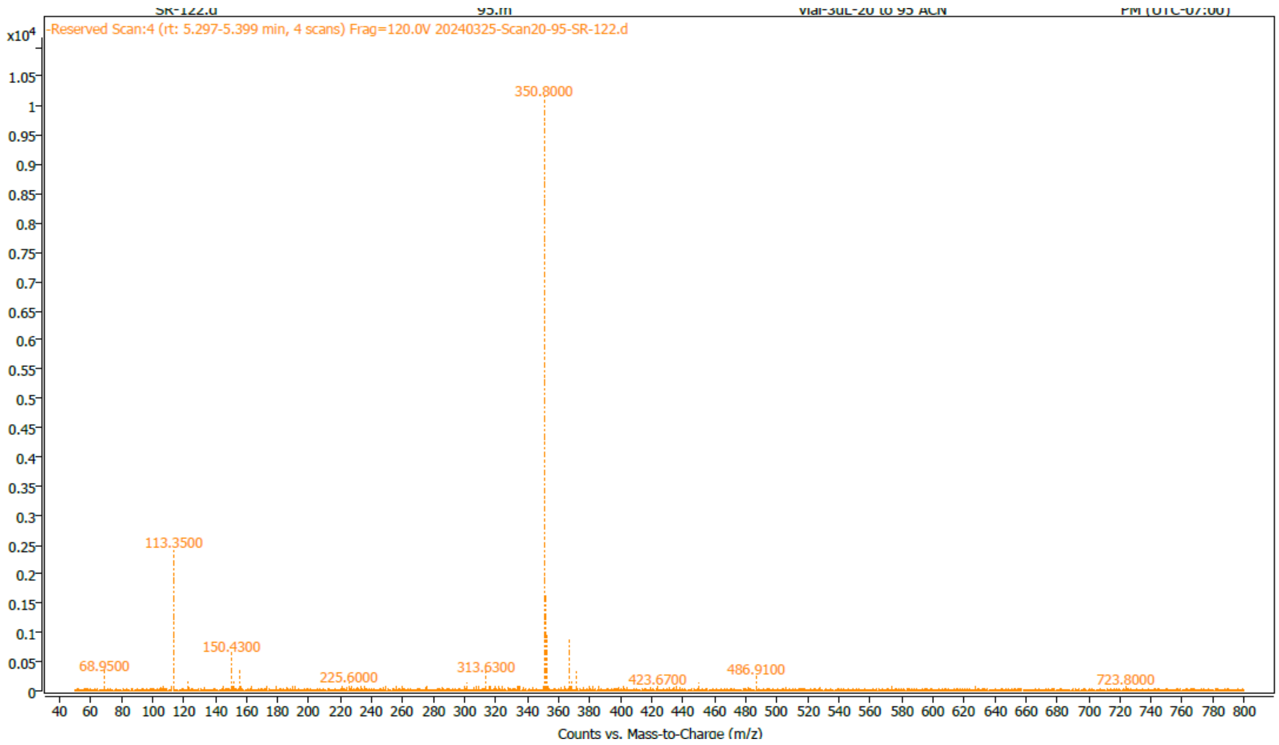

Figure S48: Mass spectrum of compound **2p**.

Screening the anticancer activity

Screening results at 10 µM

Compound 2a = NSC: D-841673

Compound 2b = NSC: D-846304

Compound 2c = NSC: D- 841672

Compound 2d = NSC: D-846307

Compound 2e = NSC: D-846303

Compound 2f = NSC: D- 845698

Compound 2g = NSC: D- 841678

Compound 2h = NSC: D- 846306

Compound 2i = NSC: D- 841675

Compound 2j = NSC: D-841677

Compound 2k = NSC: D-841676

Compound 2l = NSC: D-841671

Compound 2m = NSC: D-846771

Compound 2n = NSC: D-845704

Compound 2o = NSC: D-841679

Compound 2p = NSC: D-845697

Developmental Therapeutics Program	NSC: D-841673 / 1	Conc: 1.00E-5 Molar	Test Date: Jan 23, 2023
One Dose Mean Graph	Experiment ID: 2301OS90		Report Date: Mar 01, 2023

Panel/Cell Line	Growth Percent
**Leukemia**	
CCRF-CEM	37.22
HL-60(TB)	49.59
K-562	50.96
MOLT-4	74.19
RPMI-8226	14.13
SR	32.39
**Non-Small Cell Lung Cancer**	
A549/ATCC	82.31
EKVX	73.11
HOP-92	89.61
NCI-H226	96.48
NCI-H23	77.52
NCI-H322M	66.51
NCI-H460	56.70
NCI-H522	71.74
**Colon Cancer**	
HCC-2998	91.33
HCT-116	49.38
HCT-15	35.00
HT29	99.67
KM12	29.04
SW-620	69.11
**CNS Cancer**	
SF-268	69.81
SF-295	88.86
SF-539	41.72
SNB-19	66.75
SNB-75	92.71
U251	56.91
**Melanoma**	
LOX IMVI	31.05
MALME-3M	87.78
M14	77.06
MDA-MB-435	50.10
SK-MEL-2	68.19
SK-MEL-28	85.25
SK-MEL-5	68.82
UACC-257	76.15
UACC-62	65.26
**Ovarian Cancer**	
IGROV1	81.82
OVCAR-3	42.69
OVCAR-4	76.39
OVCAR-5	128.73
OVCAR-8	65.76
NCI/ADR-RES	64.78
**Renal Cancer**	
786-0	68.10
A498	105.99
ACHN	71.77
CAKI-1	52.53
RXF 393	46.35
SN12C	66.07
TK-10	80.33
UO-31	39.33
**Prostate Cancer**	
PC-3	77.60
DU-145	27.95
**Breast Cancer**	
MCF7	19.79
MDA-MB-231/ATCC	70.05
HS 578T	90.12
BT-549	68.85
MDA-MB-468	20.79
Mean	64.97
Delta	50.84
Range	114.60

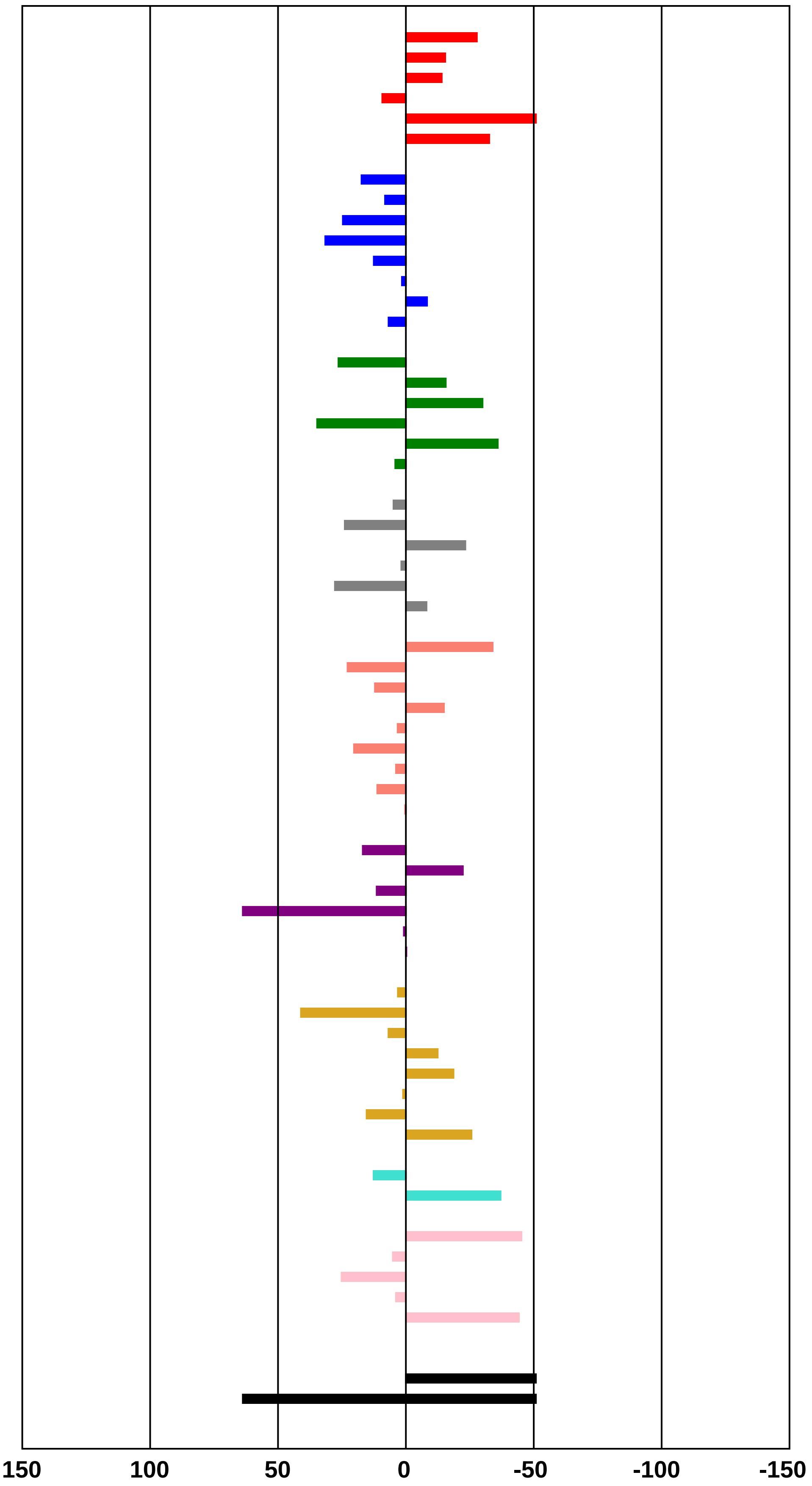

Developmental Therapeutics Program

One Dose Mean Graph

NSC: D-846304 / 1	Conc: 1.00E-5 Molar	Test Date: Jul 10, 2023
Experiment ID: 2307OS44		Report Date: Aug 11, 2023

Panel/Cell Line	Growth Percent
**Leukemia**	
CCRF-CEM	15.42
HL-60(TB)	12.04
K-562	22.81
MOLT-4	43.64
RPMI-8226	15.24
SR	6.82
**Non-Small Cell Lung Cancer**	
A549/ATCC	76.38
EKVX	68.61
HOP-62	34.77
NCI-H226	85.65
NCI-H23	71.09
NCI-H322M	84.46
NCI-H460	43.22
NCI-H522	63.86
**Colon Cancer**	
COLO 205	94.51
HCC-2998	58.22
HCT-116	21.20
HCT-15	19.06
HT29	47.06
KM12	11.91
SW-620	20.94
**CNS Cancer**	
SF-268	82.33
SF-295	38.42
SF-539	6.17
SNB-75	44.03
U251	17.63
**Melanoma**	
LOX IMVI	13.83
MALME-3M	101.24
M14	78.45
MDA-MB-435	35.94
SK-MEL-2	81.22
SK-MEL-28	93.67
SK-MEL-5	62.16
UACC-257	83.72
UACC-62	58.32
**Ovarian Cancer**	
IGROV1	32.30
OVCAR-3	-1.17
OVCAR-4	79.55
OVCAR-5	88.15
OVCAR-8	60.46
NCI/ADR-RES	10.81
SK-OV-3	110.63
**Renal Cancer**	
786-0	46.91
A498	106.30
ACHN	55.96
CAKI-1	37.69
RXF 393	23.17
SN12C	32.09
UO-31	44.34
**Prostate Cancer**	
PC-3	54.71
DU-145	12.86
**Breast Cancer**	
MCF7	7.97
MDA-MB-231/ATCC	67.38
HS 578T	79.81
BT-549	65.65
T-47D	0.69
MDA-MB-468	-22.69
Mean	47.50
Delta	70.19
Range	133.32

Mean Growth Percent - Growth Percent

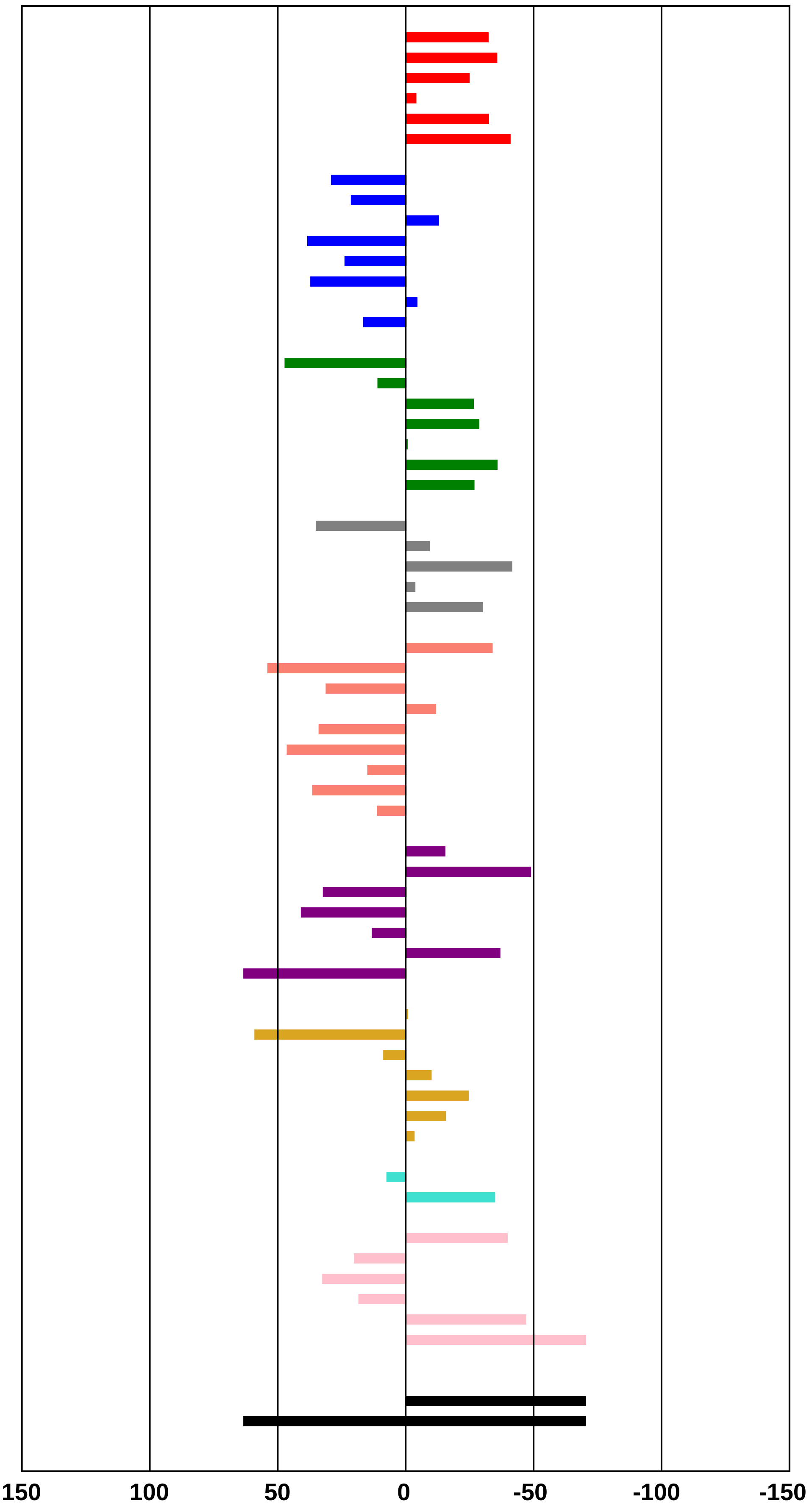

Developmental Therapeutics Program One Dose Mean Graph	NSC: D-841672 / 1	Conc: 1.00E-5 Molar	Test Date: Jan 23, 2023
	Experiment ID: 2301OS90		Report Date: Mar 01, 2023

Panel/Cell Line	Growth Percent
**Leukemia**	
CCRF-CEM	-0.21
HL-60(TB)	12.98
K-562	20.43
MOLT-4	58.71
RPMI-8226	2.32
SR	18.33
**Non-Small Cell Lung Cancer**	
A549/ATCC	55.96
EKVX	51.04
HOP-92	45.42
NCI-H226	67.10
NCI-H23	62.66
NCI-H322M	63.98
NCI-H460	39.31
NCI-H522	44.19
**Colon Cancer**	
HCC-2998	27.06
HCT-116	23.71
HCT-15	14.71
HT29	73.33
KM12	6.85
SW-620	13.76
**CNS Cancer**	
SF-268	58.62
SF-295	60.61
SF-539	-4.61
SNB-19	33.66
SNB-75	36.49
U251	14.09
**Melanoma**	
LOX IMVI	8.91
MALME-3M	85.79
M14	49.58
MDA-MB-435	4.67
SK-MEL-2	56.59
SK-MEL-28	50.57
SK-MEL-5	42.56
UACC-257	64.09
UACC-62	47.27
**Ovarian Cancer**	
IGROV1	51.24
OVCAR-3	-10.94
OVCAR-4	48.43
OVCAR-5	119.09
OVCAR-8	-13.63
NCI/ADR-RES	-10.78
**Renal Cancer**	
786-0	37.27
A498	115.00
ACHN	47.17
CAKI-1	31.60
RXF 393	23.24
SN12C	24.05
TK-10	40.37
UO-31	28.13
**Prostate Cancer**	
PC-3	43.41
DU-145	20.32
**Breast Cancer**	
MCF7	9.48
MDA-MB-231/ATCC	29.72
HS 578T	95.29
BT-549	23.21
MDA-MB-468	-4.51
Mean	36.74
Delta	50.37
Range	132.72

Mean Growth Percent - Growth Percent

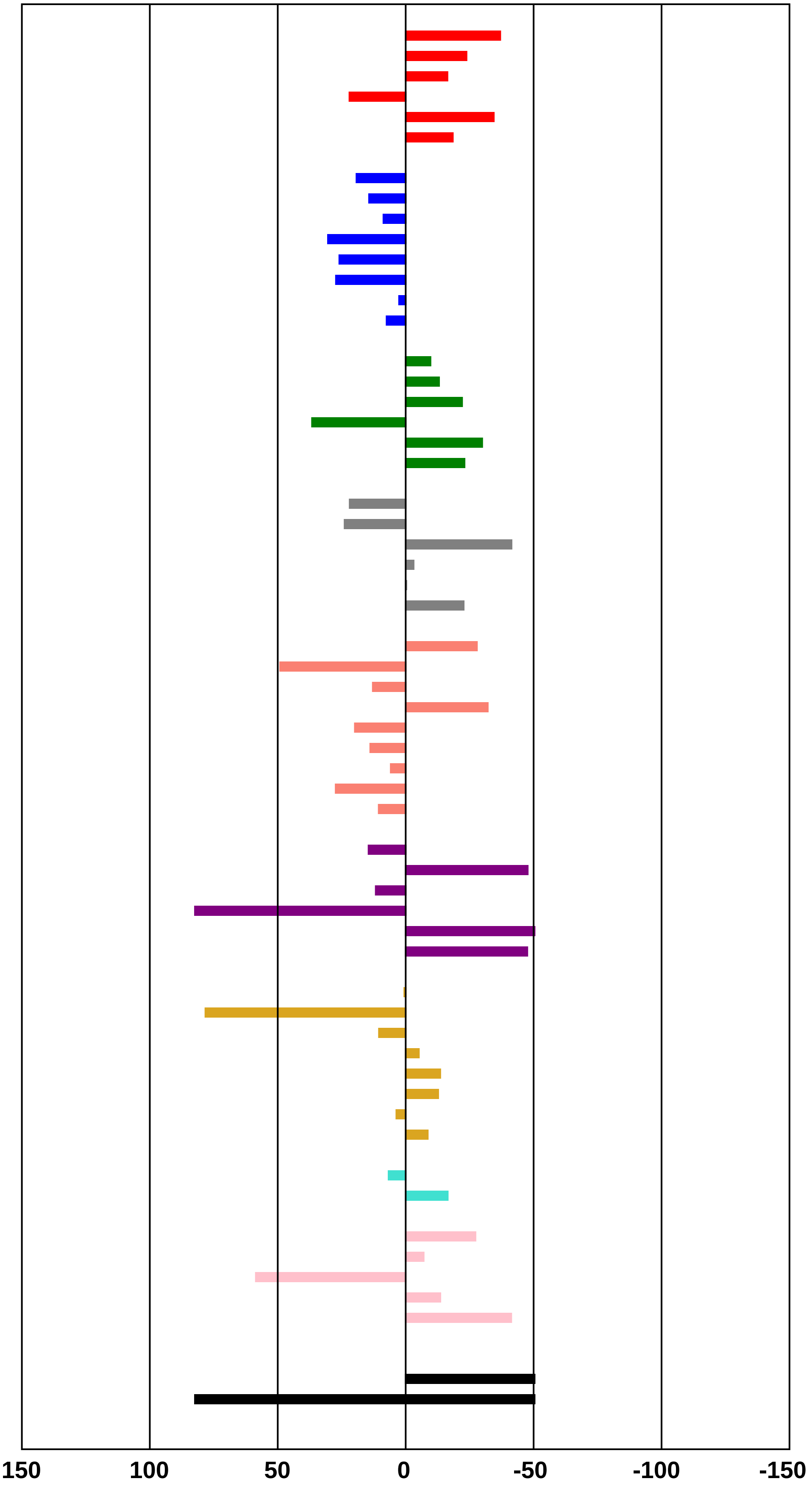

Developmental Therapeutics Program

One Dose Mean Graph

NSC: D-846307 / 1	Conc: 1.00E-5 Molar	Test Date: Jul 10, 2023
Experiment ID: 2307OS44		Report Date: Aug 11, 2023

Panel/Cell Line	Growth Percent
**Leukemia**	
CCRF-CEM	46.79
HL-60(TB)	53.03
K-562	47.41
MOLT-4	84.22
RPMI-8226	28.65
SR	14.10
**Non-Small Cell Lung Cancer**	
A549/ATCC	87.57
EKVX	35.62
HOP-62	91.65
NCI-H226	89.61
NCI-H23	91.77
NCI-H322M	106.22
NCI-H460	82.17
NCI-H522	90.13
**Colon Cancer**	
COLO 205	117.87
HCC-2998	132.42
HCT-116	44.58
HCT-15	57.40
HT29	122.14
KM12	63.81
SW-620	82.04
**CNS Cancer**	
SF-268	80.38
SF-295	82.55
SF-539	93.59
SNB-75	85.22
U251	63.31
**Melanoma**	
LOX IMVI	57.46
MALME-3M	104.51
M14	82.29
MDA-MB-435	80.75
SK-MEL-2	84.85
SK-MEL-28	101.08
SK-MEL-5	68.46
UACC-257	87.25
UACC-62	72.88
**Ovarian Cancer**	
IGROV1	91.91
OVCAR-3	77.36
OVCAR-4	90.32
OVCAR-5	116.44
OVCAR-8	85.43
NCI/ADR-RES	88.80
SK-OV-3	118.47
**Renal Cancer**	
786-0	93.10
A498	116.00
ACHN	93.42
CAKI-1	68.49
RXF 393	93.84
SN12C	80.04
UO-31	73.69
**Prostate Cancer**	
PC-3	71.78
DU-145	75.95
**Breast Cancer**	
MCF7	28.93
MDA-MB-231/ATCC	101.38
HS 578T	92.76
BT-549	76.82
T-47D	47.34
MDA-MB-468	30.83
Mean	79.42
Delta	65.32
Range	118.32

Mean Growth Percent - Growth Percent

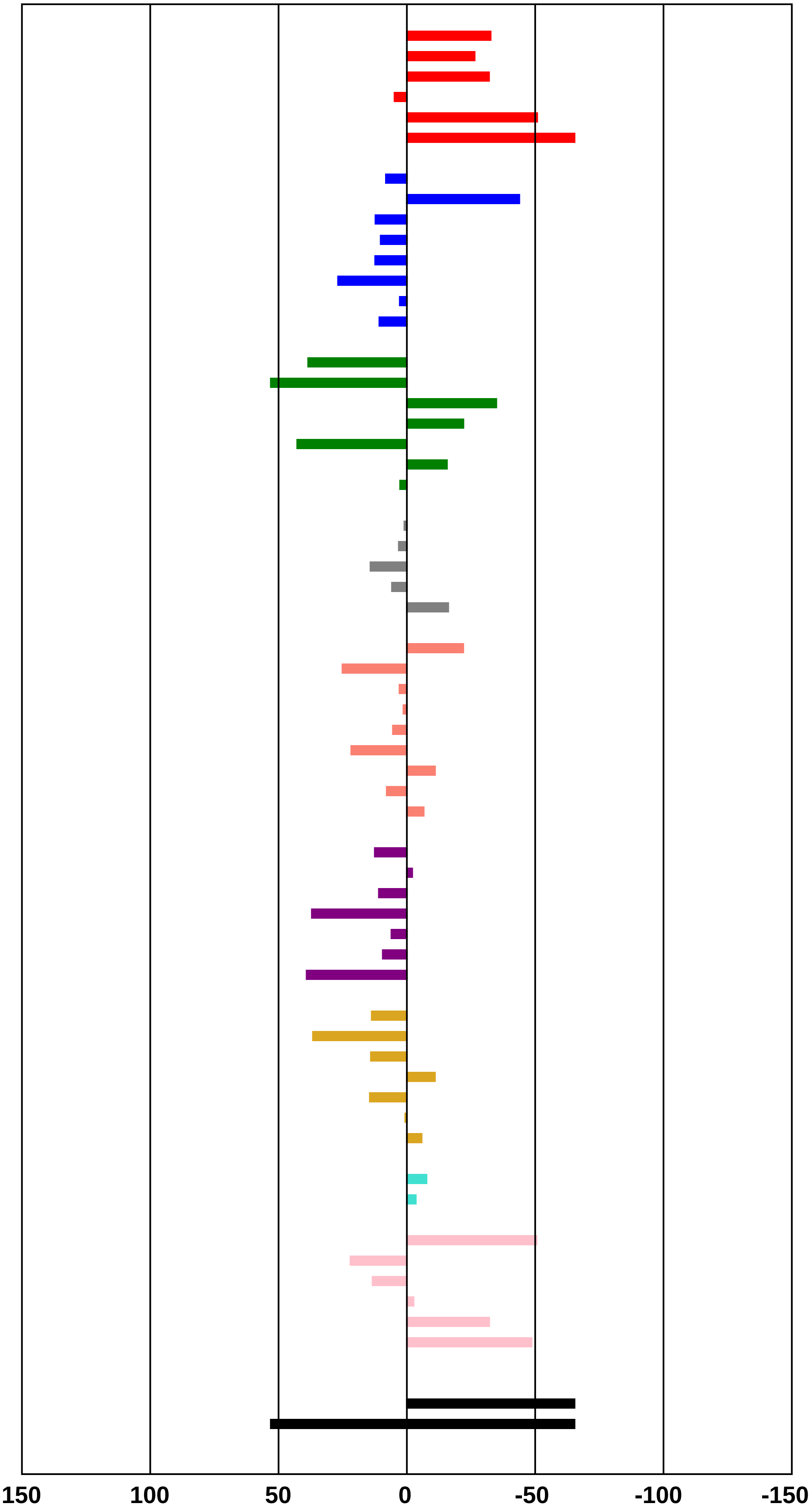

Developmental Therapeutics Program
One Dose Mean Graph

NSC: D-846303 / 1 | **Conc:** 1.00E-5 Molar | **Test Date:** Jul 10, 2023
Experiment ID: 2307OS44 | **Report Date:** Aug 11, 2023

Panel/Cell Line	Growth Percent
Leukemia	
CCRF-CEM	15.19
HL-60(TB)	25.32
K-562	16.76
MOLT-4	19.78
RPMI-8226	10.37
SR	3.42
Non-Small Cell Lung Cancer	
A549/ATCC	26.49
EKVX	34.72
HOP-62	10.45
NCI-H226	32.73
NCI-H23	18.00
NCI-H322M	50.15
NCI-H460	12.05
NCI-H522	21.99
Colon Cancer	
COLO 205	70.71
HCC-2998	13.88
HCT-116	3.28
HCT-15	-22.01
HT29	29.92
KM12	-30.72
SW-620	8.60
CNS Cancer	
SF-268	51.84
SF-295	41.84
SF-539	-10.65
SNB-75	8.74
U251	12.07
Melanoma	
LOX IMVI	-26.09
MALME-3M	86.93
M14	40.25
MDA-MB-435	17.26
SK-MEL-2	32.10
SK-MEL-28	44.69
SK-MEL-5	16.05
UACC-257	52.71
UACC-62	29.97
Ovarian Cancer	
IGROV1	33.98
OVCAR-3	8.26
OVCAR-4	31.68
OVCAR-5	36.37
OVCAR-8	10.39
NCI/ADR-RES	-5.11
SK-OV-3	59.91
Renal Cancer	
786-0	13.35
A498	98.01
ACHN	22.90
CAKI-1	23.85
RXF 393	10.66
SN12C	12.00
UO-31	34.33
Prostate Cancer	
PC-3	31.83
DU-145	16.10
Breast Cancer	
MCF7	7.90
MDA-MB-231/ATCC	-0.43
HS 578T	47.21
BT-549	2.28
T-47D	4.54
MDA-MB-468	-7.12
Mean	22.13
Delta	52.85
Range	128.73

Mean Growth Percent - Growth Percent

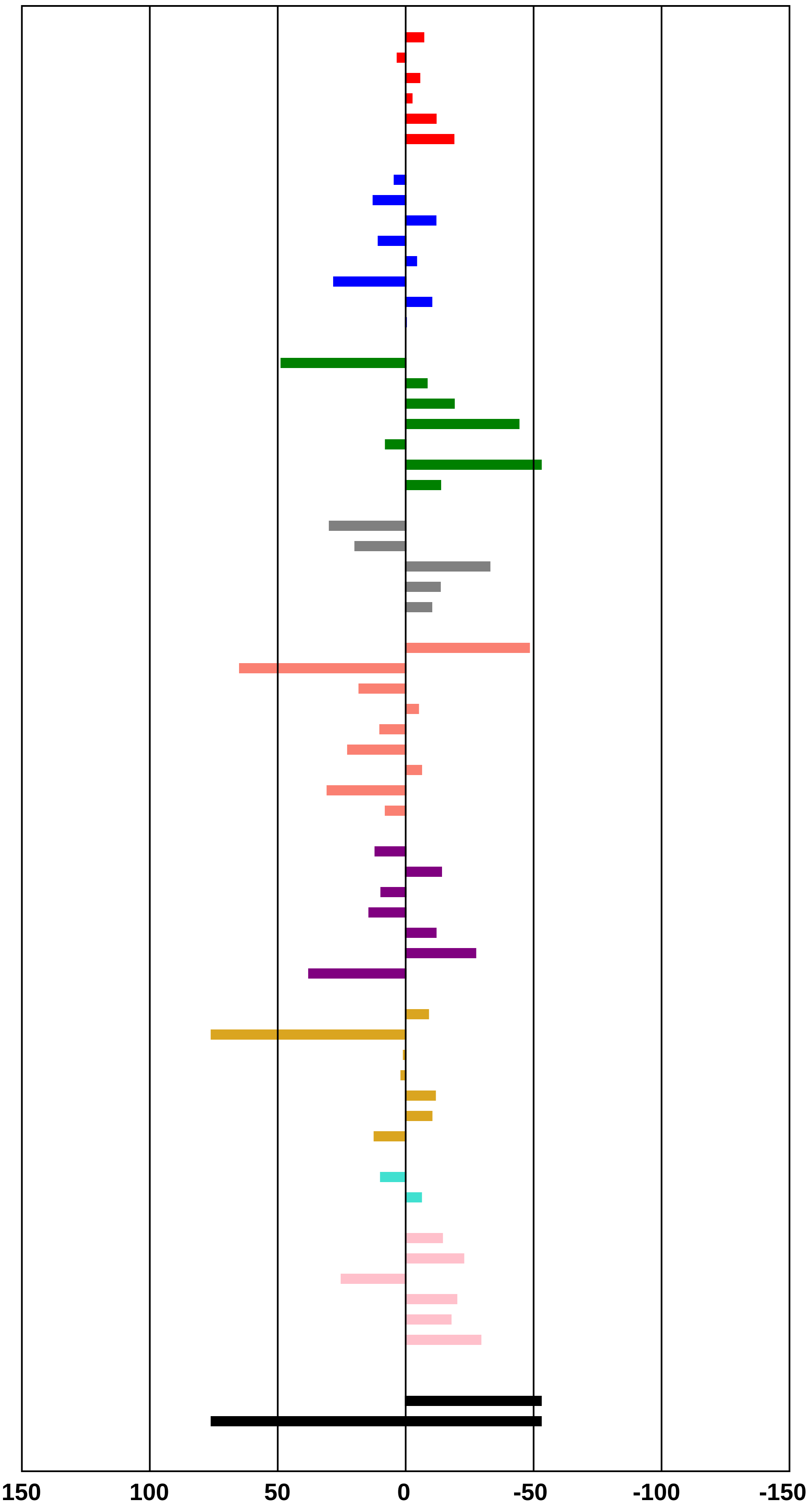

Developmental Therapeutics Program One Dose Mean Graph	NSC: D-845698 / 1	Conc: 1.00E-5 Molar	Test Date: Jun 12, 2023
	Experiment ID: 2306OS36		Report Date: Jul 23, 2023

Panel/Cell Line	Growth Percent
**Leukemia**	
CCRF-CEM	3.18
HL-60(TB)	-0.98
K-562	7.94
MOLT-4	10.18
RPMI-8226	13.82
**Non-Small Cell Lung Cancer**	
A549/ATCC	61.14
EKVX	40.96
HOP-62	-2.73
HOP-92	34.78
NCI-H226	20.49
NCI-H23	36.43
NCI-H460	82.56
NCI-H522	13.54
**Colon Cancer**	
COLO 205	78.15
HCC-2998	-21.28
HCT-116	6.98
HCT-15	-19.78
HT29	31.17
KM12	6.77
SW-620	8.02
**CNS Cancer**	
SF-268	67.94
SF-295	42.62
SF-539	-30.44
SNB-19	15.84
U251	11.35
**Melanoma**	
LOX IMVI	-48.15
M14	34.39
MDA-MB-435	22.51
SK-MEL-2	58.38
SK-MEL-28	45.71
SK-MEL-5	27.99
UACC-257	66.80
UACC-62	33.88
**Ovarian Cancer**	
OVCAR-3	-24.25
OVCAR-4	76.31
OVCAR-5	50.81
OVCAR-8	4.96
NCI/ADR-RES	-8.39
SK-OV-3	60.83
**Renal Cancer**	
786-0	16.59
A498	105.85
ACHN	34.61
CAKI-1	53.21
RXF 393	9.78
SN12C	4.62
TK-10	40.37
**Prostate Cancer**	
PC-3	30.44
DU-145	7.29
**Breast Cancer**	
MCF7	4.86
MDA-MB-231/ATCC	39.29
HS 578T	47.98
BT-549	-14.41
T-47D	1.38
MDA-MB-468	-21.19
Mean	23.72
Delta	71.87
Range	154.00

Mean Growth Percent - Growth Percent

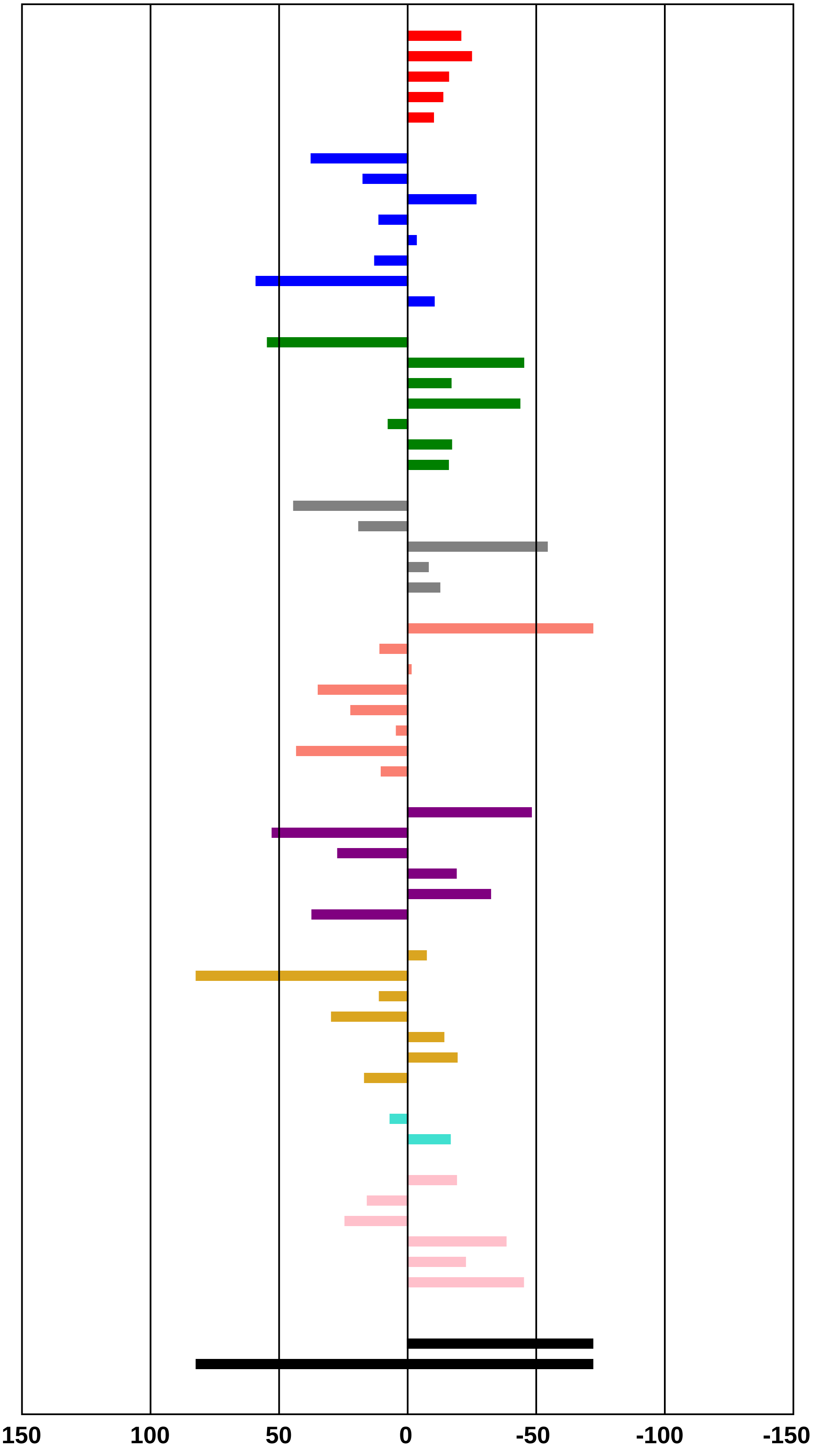

Developmental Therapeutics Program

One Dose Mean Graph

NSC: D-841678 / 1 | **Conc:** 1.00E-5 Molar | **Test Date:** Jan 23, 2023

Experiment ID: 2301OS90 | **Report Date:** Mar 01, 2023

Panel/Cell Line	Growth Percent
Leukemia	
CCRF-CEM	-11.02
HL-60(TB)	13.16
K-562	13.64
MOLT-4	30.17
RPMI-8226	1.40
SR	8.46
Non-Small Cell Lung Cancer	
A549/ATCC	64.07
EKVX	62.90
HOP-62	11.94
HOP-92	67.12
NCI-H226	54.51
NCI-H23	65.01
NCI-H322M	44.13
NCI-H460	39.06
NCI-H522	21.10
Colon Cancer	
COLO 205	89.96
HCC-2998	3.23
HCT-116	16.57
HCT-15	5.48
HT29	76.26
KM12	1.17
SW-620	5.70
CNS Cancer	
SF-268	46.10
SF-295	90.37
SF-539	-25.34
SNB-19	32.68
SNB-75	2.52
U251	15.53
Melanoma	
LOX IMVI	-5.17
MALME-3M	72.94
M14	74.48
MDA-MB-435	8.26
SK-MEL-2	62.33
SK-MEL-28	64.25
SK-MEL-5	48.84
UACC-257	69.10
UACC-62	46.84
Ovarian Cancer	
IGROV1	37.11
OVCAR-3	-12.30
OVCAR-4	49.51
OVCAR-5	115.99
OVCAR-8	-34.08
NCI/ADR-RES	-13.89
SK-OV-3	96.66
Renal Cancer	
786-0	25.03
A498	108.90
ACHN	55.01
CAKI-1	32.76
RXF 393	15.96
SN12C	19.22
TK-10	37.82
UO-31	22.38
Prostate Cancer	
PC-3	48.79
DU-145	23.90
Breast Cancer	
MCF7	10.52
MDA-MB-231/ATCC	9.49
HS 578T	74.60
BT-549	19.50
T-47D	29.03
MDA-MB-468	-4.75
Mean	34.25
Delta	68.33
Range	150.07

Mean Growth Percent - Growth Percent

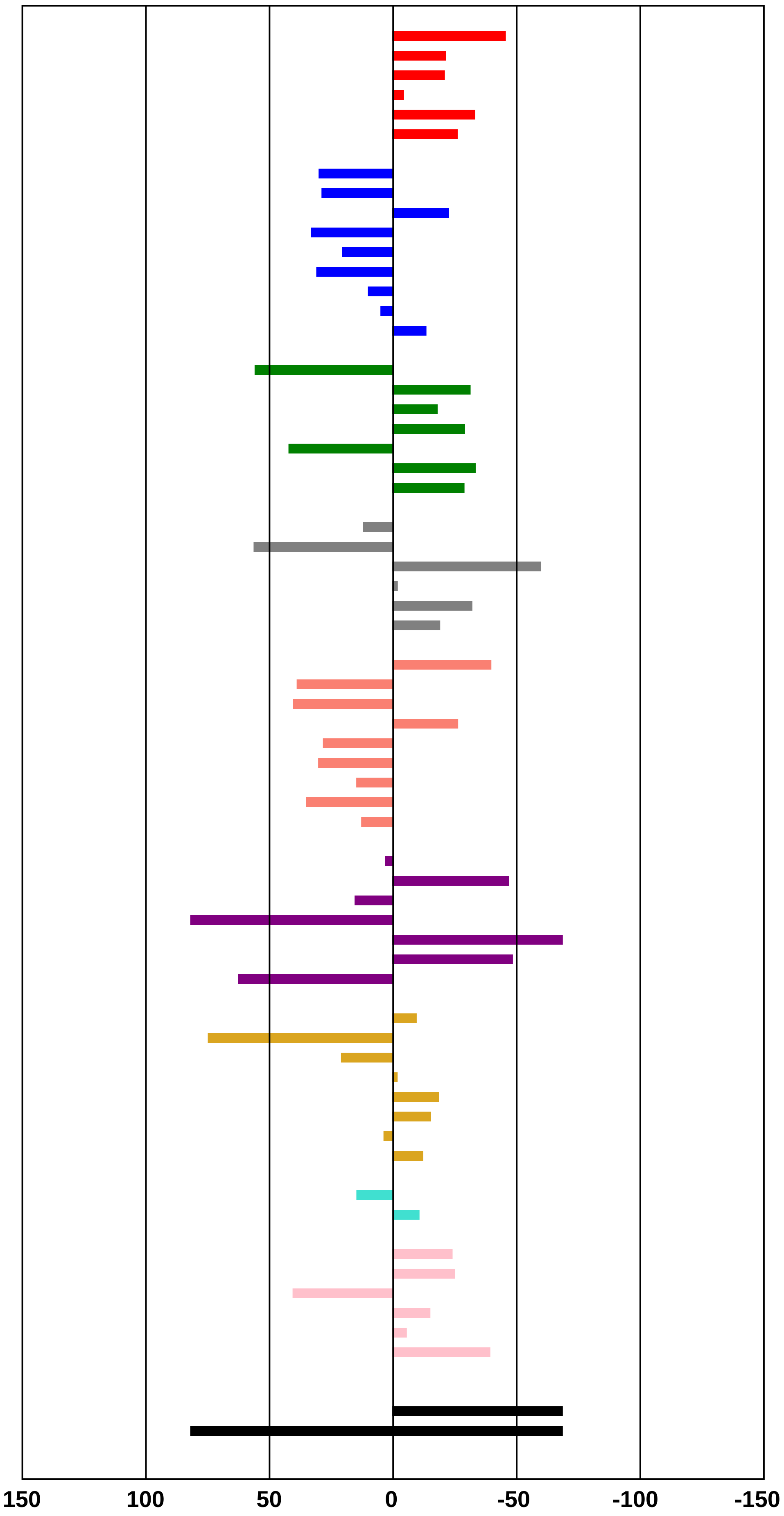

Developmental Therapeutics Program

One Dose Mean Graph

NSC: D-846306 / 1 | **Conc:** 1.00E-5 Molar | **Test Date:** Jul 10, 2023

Experiment ID: 2307OS44 | **Report Date:** Aug 11, 2023

Panel/Cell Line	Growth Percent
**Leukemia**	
CCRF-CEM	13.78
HL-60(TB)	5.25
K-562	13.77
MOLT-4	13.31
RPMI-8226	9.39
SR	-10.99
**Non-Small Cell Lung Cancer**	
A549/ATCC	19.51
EKVX	0.65
HOP-62	0.68
NCI-H226	-41.90
NCI-H23	-72.77
NCI-H322M	28.92
NCI-H460	-30.68
NCI-H522	-58.81
**Colon Cancer**	
COLO 205	-24.88
HCC-2998	-86.72
HCT-116	-6.25
HCT-15	-36.42
HT29	20.35
KM12	-78.40
SW-620	-37.80
**CNS Cancer**	
SF-268	27.46
SF-295	4.14
SF-539	-85.88
SNB-75	10.38
U251	-75.99
**Melanoma**	
LOX IMVI	-98.99
MALME-3M	-42.94
M14	1.86
MDA-MB-435	-36.95
SK-MEL-2	16.88
SK-MEL-28	-47.02
SK-MEL-5	-68.80
UACC-257	9.40
UACC-62	-56.71
**Ovarian Cancer**	
IGROV1	10.16
OVCAR-3	-79.48
OVCAR-4	13.05
OVCAR-5	-37.40
OVCAR-8	13.44
NCI/ADR-RES	8.19
SK-OV-3	44.61
**Renal Cancer**	
786-0	-10.85
A498	98.87
ACHN	-58.35
CAKI-1	-56.46
RXF 393	-91.92
SN12C	-25.64
UO-31	-89.63
**Prostate Cancer**	
PC-3	8.43
DU-145	8.55
**Breast Cancer**	
MCF7	-45.42
MDA-MB-231/ATCC	-17.58
HS 578T	24.47
BT-549	-14.03
T-47D	-56.63
MDA-MB-468	-82.86
Mean	-21.75
Delta	77.24
Range	197.86

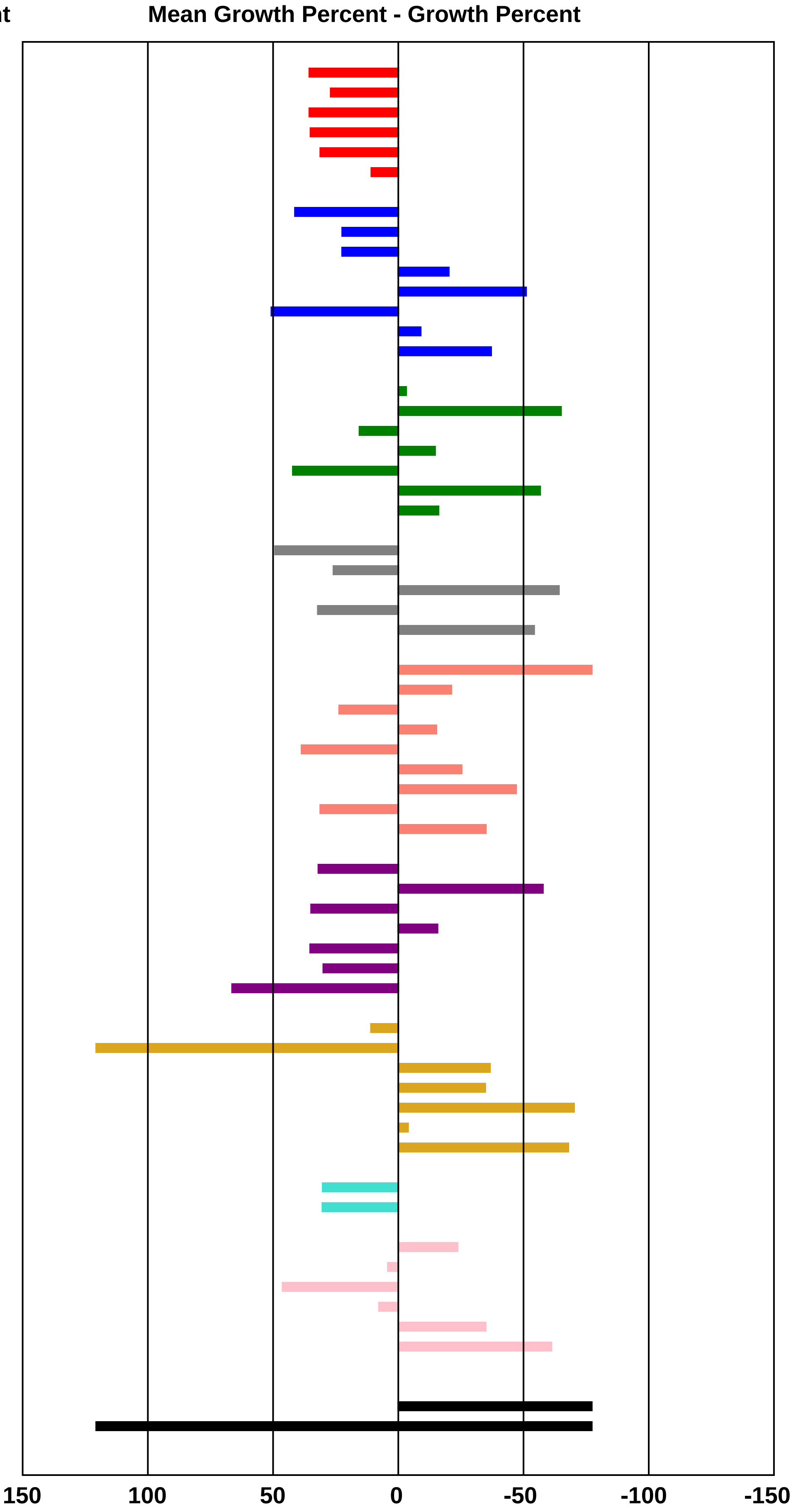

Developmental Therapeutics Program

One Dose Mean Graph

**NSC:** D-841675 / 1	**Conc:** 1.00E-5 Molar	**Test Date:** Jan 23, 2023
**Experiment ID:** 2301OS90		**Report Date:** Mar 01, 2023

Panel/Cell Line	Growth Percent
Leukemia	
CCRF-CEM	-7.25
HL-60(TB)	-5.39
K-562	12.28
MOLT-4	14.20
RPMI-8226	-7.90
SR	-4.81
Non-Small Cell Lung Cancer	
A549/ATCC	75.71
EKVX	74.66
HOP-62	21.63
HOP-92	57.63
NCI-H226	91.38
NCI-H23	49.20
NCI-H322M	50.72
NCI-H460	11.33
NCI-H522	33.00
Colon Cancer	
COLO 205	91.53
HCC-2998	-11.87
HCT-116	-47.76
HCT-15	-62.10
HT29	62.83
KM12	-72.47
SW-620	-44.79
CNS Cancer	
SF-268	47.36
SF-295	86.08
SF-539	-25.22
SNB-19	41.18
SNB-75	35.98
U251	13.65
Melanoma	
LOX IMVI	-86.21
MALME-3M	75.16
M14	69.52
MDA-MB-435	3.76
SK-MEL-2	49.55
SK-MEL-28	23.87
SK-MEL-5	53.78
UACC-257	63.26
UACC-62	47.53
Ovarian Cancer	
IGROV1	16.06
OVCAR-3	-6.00
OVCAR-4	49.16
OVCAR-5	109.56
OVCAR-8	7.06
NCI/ADR-RES	-4.45
SK-OV-3	107.77
Renal Cancer	
786-0	25.64
A498	104.64
ACHN	3.88
CAKI-1	31.07
RXF 393	-4.35
SN12C	18.63
TK-10	12.90
UO-31	-41.32
Prostate Cancer	
PC-3	51.28
DU-145	21.39
Breast Cancer	
MCF7	5.51
MDA-MB-231/ATCC	10.75
HS 578T	68.11
BT-549	20.09
T-47D	44.32
MDA-MB-468	-18.95
Mean	25.23
Delta	111.44
Range	195.77

Mean Growth Percent - Growth Percent

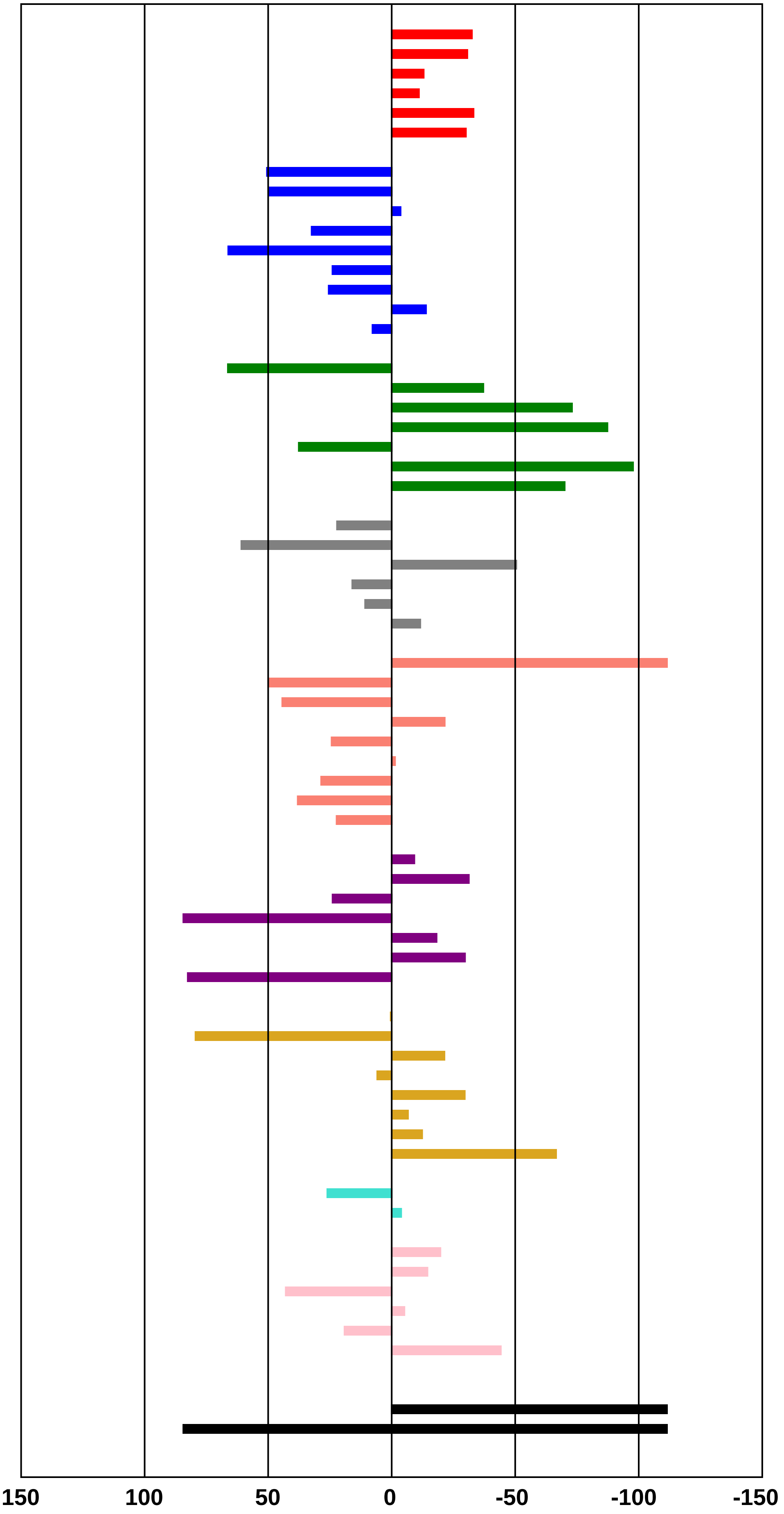

Developmental Therapeutics Program	NSC: D-841677 / 1	Conc: 1.00E-5 Molar	Test Date: Jan 23, 2023
One Dose Mean Graph	Experiment ID: 2301OS90		Report Date: Mar 01, 2023

Panel/Cell Line	Growth Percent
**Leukemia**	
CCRF-CEM	24.60
HL-60(TB)	61.00
K-562	34.16
MOLT-4	68.76
RPMI-8226	18.86
SR	45.55
**Non-Small Cell Lung Cancer**	
A549/ATCC	74.27
EKVX	55.52
HOP-62	54.10
HOP-92	98.01
NCI-H226	93.76
NCI-H23	68.54
NCI-H322M	62.15
NCI-H460	53.09
NCI-H522	60.24
**Colon Cancer**	
COLO 205	121.66
HCC-2998	77.15
HCT-116	46.74
HCT-15	38.58
HT29	98.71
KM12	29.81
SW-620	35.87
**CNS Cancer**	
SF-268	86.89
SF-295	83.38
SF-539	42.96
SNB-19	65.54
SNB-75	78.06
U251	60.37
**Melanoma**	
LOX IMVI	31.22
MALME-3M	84.65
M14	83.02
MDA-MB-435	39.01
SK-MEL-2	89.83
SK-MEL-28	92.85
SK-MEL-5	56.13
UACC-257	80.08
UACC-62	59.43
**Ovarian Cancer**	
IGROV1	83.42
OVCAR-3	14.99
OVCAR-4	59.22
OVCAR-5	138.42
OVCAR-8	17.26
NCI/ADR-RES	31.00
SK-OV-3	102.36
**Renal Cancer**	
786-0	70.71
A498	130.77
ACHN	71.22
CAKI-1	43.41
RXF 393	55.42
SN12C	62.33
TK-10	79.78
UO-31	46.79
**Prostate Cancer**	
PC-3	74.84
DU-145	54.64
**Breast Cancer**	
MCF7	28.33
MDA-MB-231/ATCC	71.69
HS 578T	101.32
BT-549	56.13
T-47D	38.31
MDA-MB-468	11.77
Mean	63.31
Delta	51.54
Range	126.65

Mean Growth Percent - Growth Percent

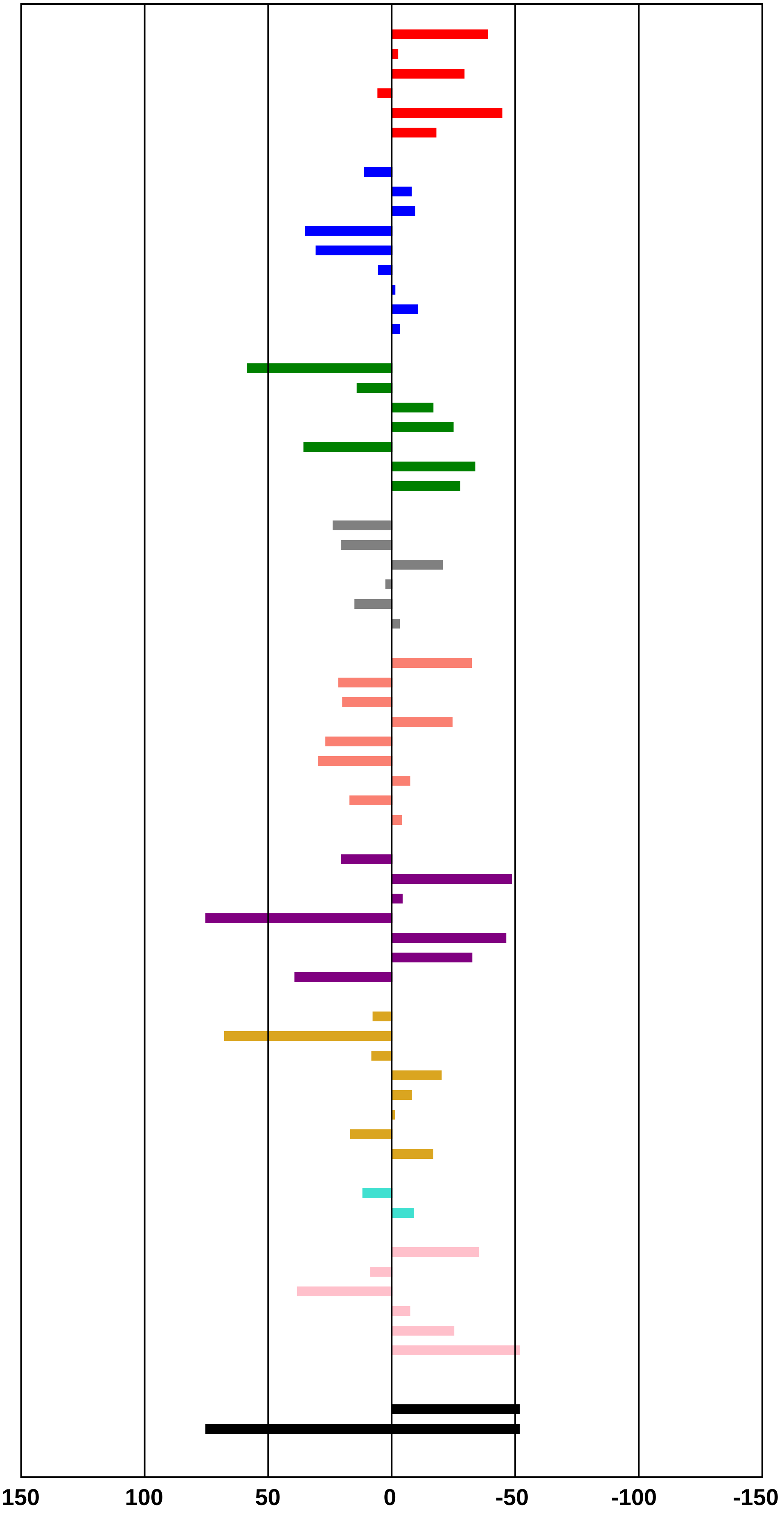

Developmental Therapeutics Program One Dose Mean Graph	**NSC:** D-841676 / 1	**Conc:** 1.00E-5 Molar	**Test Date:** Jan 23, 2023
	**Experiment ID:** 2301OS90		**Report Date:** Mar 01, 2023

Panel/Cell Line	Growth Percent
Leukemia	
CCRF-CEM	68.52
HL-60(TB)	87.05
K-562	47.03
MOLT-4	62.10
RPMI-8226	41.48
SR	57.41
Non-Small Cell Lung Cancer	
A549/ATCC	68.06
EKVX	67.01
HOP-62	86.25
HOP-92	118.71
NCI-H226	88.28
NCI-H23	71.13
NCI-H322M	81.14
NCI-H460	85.67
NCI-H522	58.91
Colon Cancer	
COLO 205	115.46
HCC-2998	95.30
HCT-116	72.22
HCT-15	64.44
HT29	94.03
KM12	62.65
SW-620	89.86
CNS Cancer	
SF-268	99.28
SF-295	79.76
SF-539	88.70
SNB-19	71.20
SNB-75	96.00
U251	67.11
Melanoma	
LOX IMVI	60.24
MALME-3M	79.42
M14	83.18
MDA-MB-435	58.35
SK-MEL-2	88.89
SK-MEL-28	92.66
SK-MEL-5	51.26
UACC-257	64.13
UACC-62	58.71
Ovarian Cancer	
IGROV1	82.27
OVCAR-3	82.20
OVCAR-4	51.23
OVCAR-5	142.09
OVCAR-8	83.37
NCI/ADR-RES	56.92
SK-OV-3	92.59
Renal Cancer	
786-0	91.67
A498	129.94
ACHN	75.67
CAKI-1	68.64
RXF 393	90.79
SN12C	75.73
TK-10	86.81
UO-31	48.26
Prostate Cancer	
PC-3	71.73
DU-145	92.79
Breast Cancer	
MCF7	59.99
MDA-MB-231/ATCC	90.28
HS 578T	118.00
BT-549	82.22
T-47D	32.35
MDA-MB-468	35.38
Mean	77.71
Delta	45.36
Range	109.74

Mean Growth Percent - Growth Percent

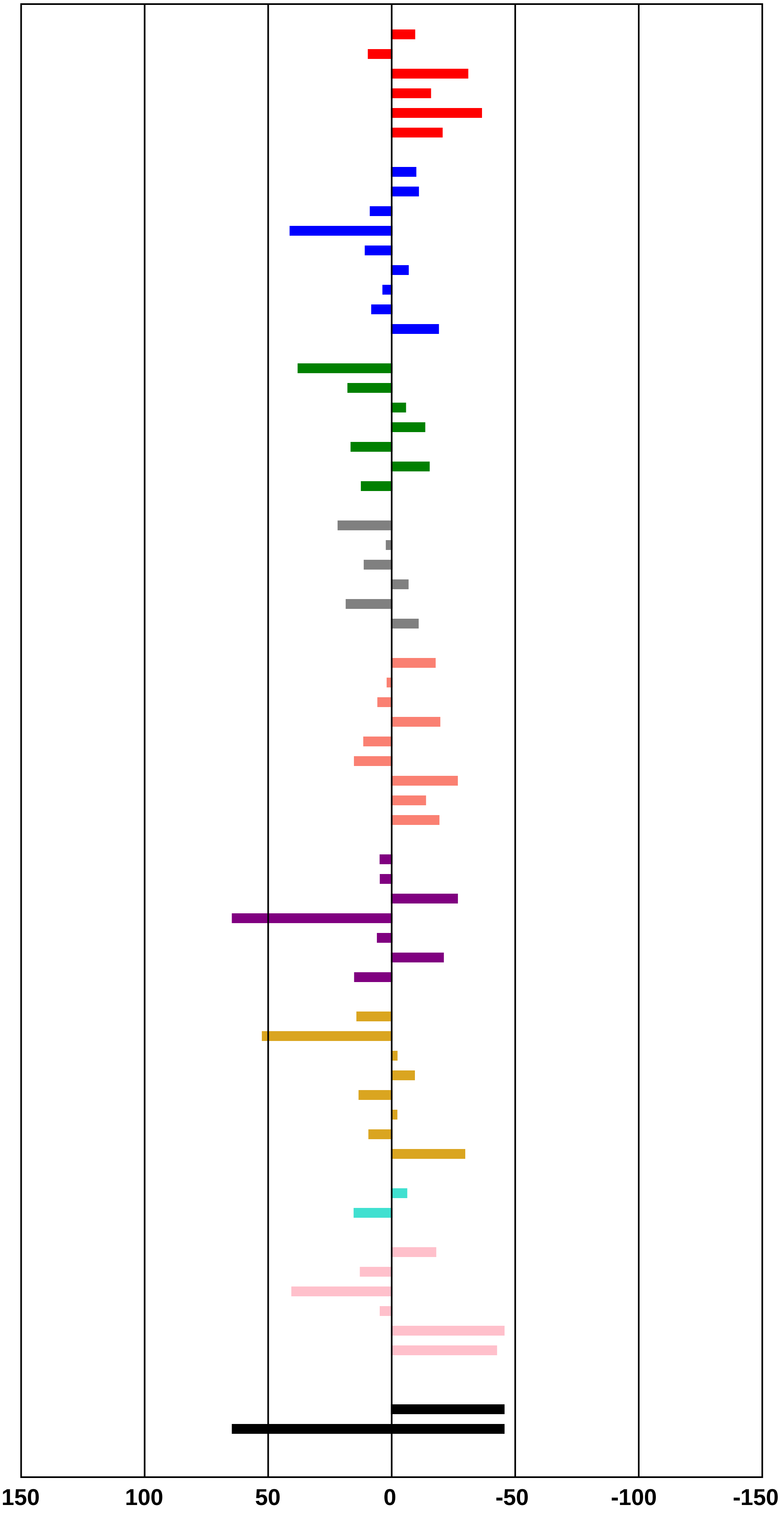

Developmental Therapeutics Program

One Dose Mean Graph

NSC: D-841671 / 1	Conc: 1.00E-5 Molar	Test Date: Jan 23, 2023
Experiment ID: 2301OS90		Report Date: Mar 01, 2023

Panel/Cell Line	Growth Percent
**Leukemia**	
CCRF-CEM	58.98
HL-60(TB)	58.48
K-562	56.62
MOLT-4	81.55
RPMI-8226	19.63
SR	43.69
**Non-Small Cell Lung Cancer**	
A549/ATCC	82.86
EKVX	84.75
HOP-92	100.67
NCI-H226	93.62
NCI-H23	81.76
NCI-H322M	92.40
NCI-H460	85.62
NCI-H522	61.35
**Colon Cancer**	
HCC-2998	100.59
HCT-116	75.95
HCT-15	50.75
HT29	96.79
KM12	42.17
SW-620	75.28
**CNS Cancer**	
SF-268	81.05
SF-295	83.49
SF-539	69.82
SNB-19	75.59
SNB-75	98.01
U251	62.29
**Melanoma**	
LOX IMVI	44.25
MALME-3M	90.04
M14	87.51
MDA-MB-435	63.89
SK-MEL-2	80.29
SK-MEL-28	101.91
SK-MEL-5	55.87
UACC-257	67.97
UACC-62	71.85
**Ovarian Cancer**	
IGROV1	89.07
OVCAR-3	66.86
OVCAR-4	72.33
OVCAR-5	144.69
OVCAR-8	76.93
NCI/ADR-RES	76.38
**Renal Cancer**	
786-0	78.11
A498	120.21
ACHN	85.64
CAKI-1	59.04
RXF 393	77.53
SN12C	74.87
TK-10	80.89
UO-31	49.00
**Prostate Cancer**	
PC-3	82.15
DU-145	58.53
**Breast Cancer**	
MCF7	30.84
MDA-MB-231/ATCC	89.32
HS 578T	96.50
BT-549	75.04
MDA-MB-468	15.11
Mean	74.58
Delta	59.47
Range	129.58

Mean Growth Percent - Growth Percent

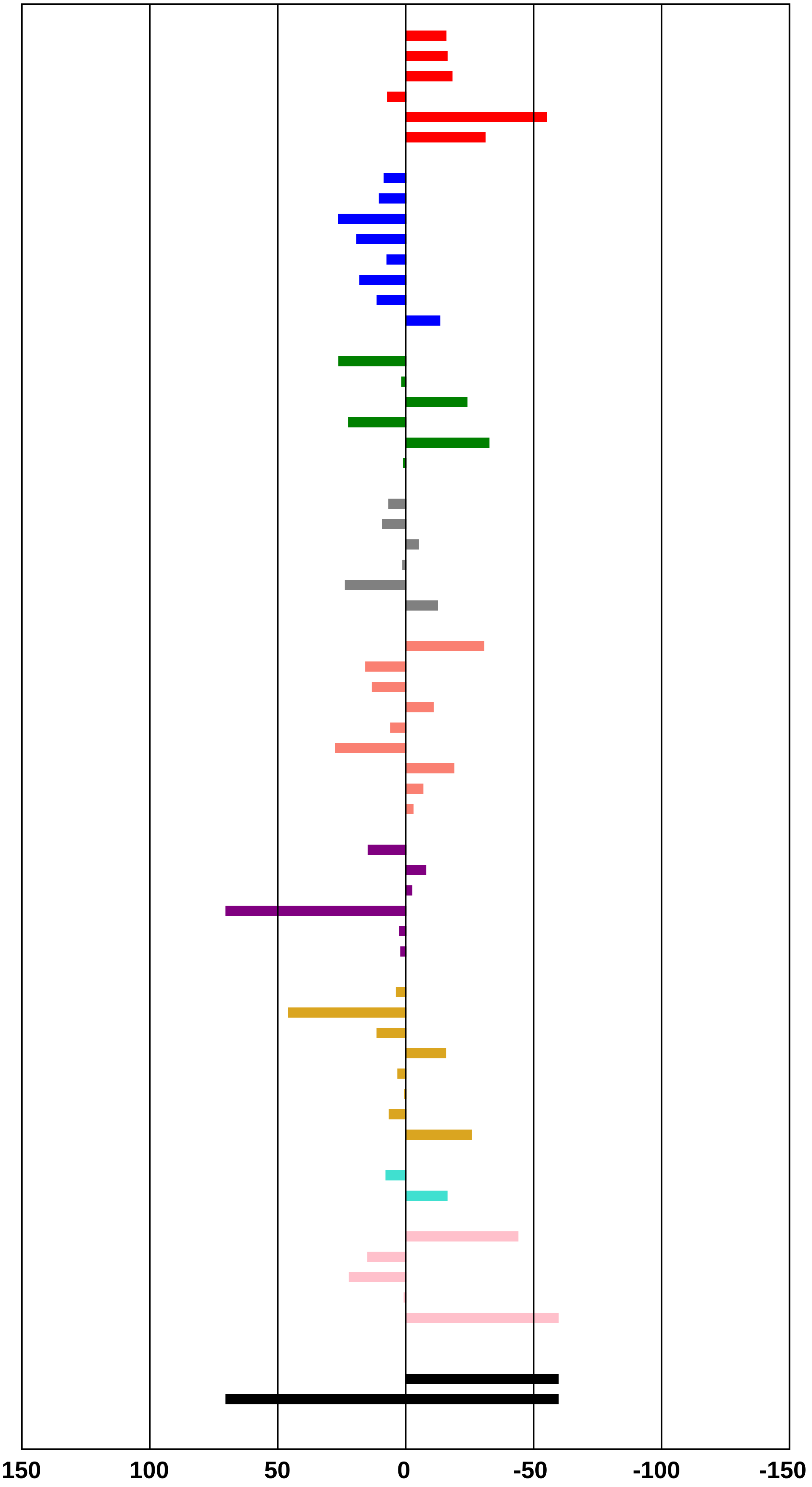

Developmental Therapeutics Program
One Dose Mean Graph

NSC: D-846771 / 1	Conc: 1.00E-5 Molar	Test Date: Jul 17, 2023
Experiment ID: 2307OS48		Report Date: Sep 05, 2023

Panel/Cell Line	Growth Percent
**Leukemia**	
CCRF-CEM	12.41
HL-60(TB)	12.74
K-562	27.51
MOLT-4	36.78
RPMI-8226	9.09
SR	5.15
**Non-Small Cell Lung Cancer**	
A549/ATCC	43.09
EKVX	54.52
HOP-62	91.75
HOP-92	61.54
NCI-H226	90.78
NCI-H23	86.47
NCI-H322M	62.76
NCI-H460	40.11
NCI-H522	32.87
**Colon Cancer**	
COLO 205	102.52
HCC-2998	79.76
HCT-116	46.54
HCT-15	32.68
KM12	25.26
SW-620	47.89
**CNS Cancer**	
SF-268	54.04
SF-295	26.31
SF-539	22.73
SNB-19	37.21
SNB-75	50.41
U251	20.33
**Melanoma**	
LOX IMVI	21.68
MALME-3M	97.13
M14	53.99
MDA-MB-435	51.86
SK-MEL-2	94.22
SK-MEL-28	70.00
SK-MEL-5	23.46
UACC-257	35.39
UACC-62	45.46
**Ovarian Cancer**	
IGROV1	73.86
OVCAR-3	24.27
OVCAR-4	62.20
OVCAR-5	81.68
OVCAR-8	44.61
NCI/ADR-RES	30.95
SK-OV-3	85.62
**Renal Cancer**	
A498	108.77
ACHN	52.53
CAKI-1	41.93
RXF 393	52.45
SN12C	44.99
TK-10	64.47
UO-31	53.74
**Prostate Cancer**	
PC-3	41.63
DU-145	41.98
**Breast Cancer**	
MCF7	16.65
MDA-MB-231/ATCC	68.26
HS 578T	49.25
BT-549	70.95
T-47D	34.48
MDA-MB-468	41.07
Mean	49.88
Delta	44.73
Range	103.62

Mean Growth Percent - Growth Percent

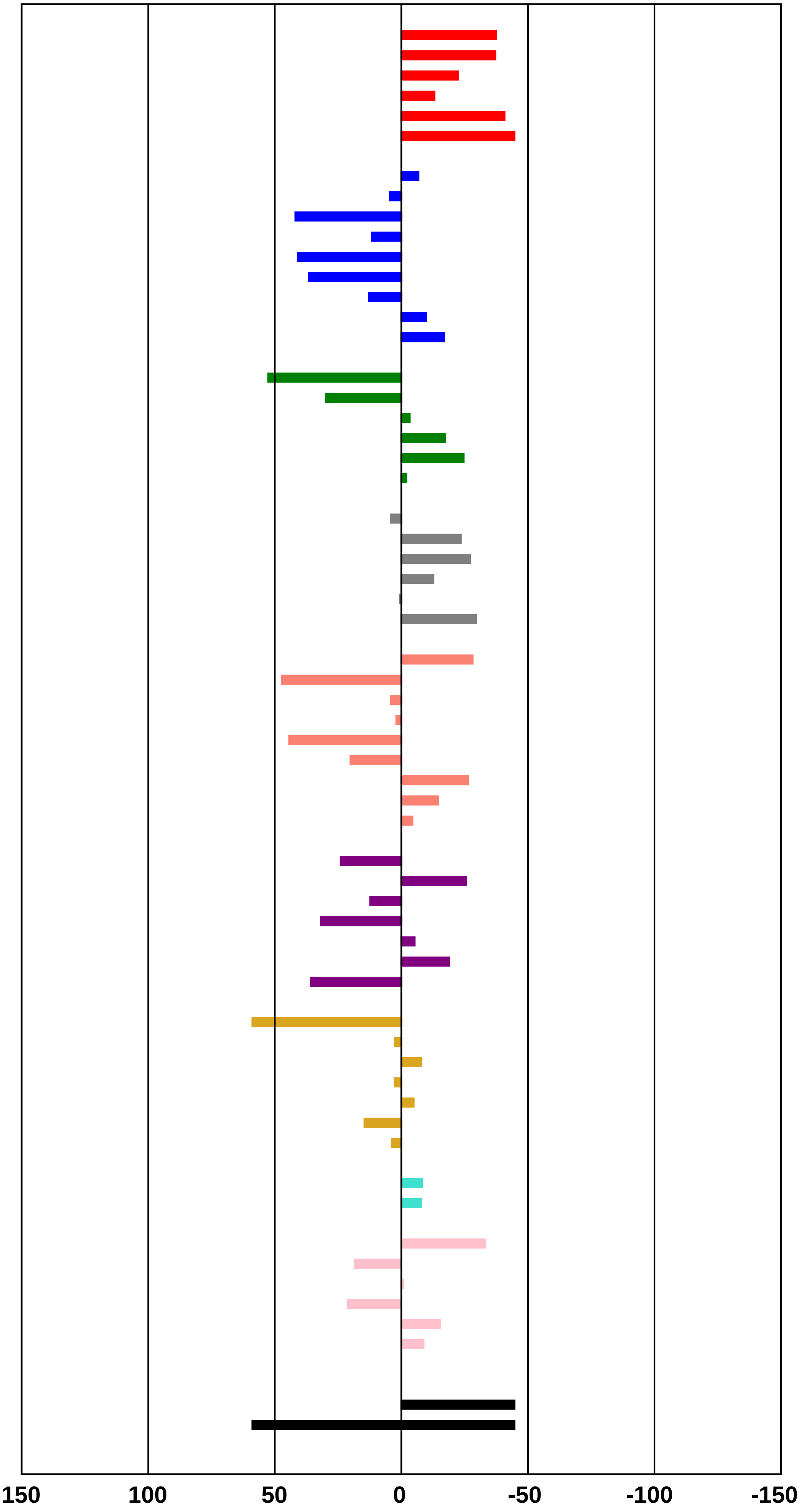

Developmental Therapeutics Program	NSC: D-845704 / 1	Conc: 1.00E-5 Molar	Test Date: Jun 12, 2023
One Dose Mean Graph	Experiment ID: 2306OS36		Report Date: Jul 23, 2023

Panel/Cell Line	Growth Percent
Leukemia	
CCRF-CEM	53.72
HL-60(TB)	55.84
K-562	48.87
MOLT-4	72.27
RPMI-8226	35.64
Non-Small Cell Lung Cancer	
A549/ATCC	90.00
EKVX	56.24
HOP-62	88.82
HOP-92	103.21
NCI-H226	79.73
NCI-H23	72.87
NCI-H460	89.77
NCI-H522	73.46
Colon Cancer	
COLO 205	122.75
HCC-2998	80.73
HCT-116	62.30
HCT-15	45.78
HT29	114.99
KM12	73.40
SW-620	84.93
CNS Cancer	
SF-268	89.73
SF-295	72.50
SF-539	87.29
SNB-19	77.37
U251	68.60
Melanoma	
LOX IMVI	57.81
M14	75.84
MDA-MB-435	84.73
SK-MEL-2	64.72
SK-MEL-28	92.80
SK-MEL-5	37.84
UACC-257	84.44
UACC-62	65.16
Ovarian Cancer	
OVCAR-3	91.16
OVCAR-4	52.05
OVCAR-5	120.39
OVCAR-8	81.51
NCI/ADR-RES	48.05
SK-OV-3	100.52
Renal Cancer	
786-0	85.94
A498	100.71
ACHN	76.96
CAKI-1	82.85
RXF 393	68.86
SN12C	80.95
TK-10	94.10
Prostate Cancer	
PC-3	75.69
DU-145	86.87
Breast Cancer	
MCF7	45.98
MDA-MB-231/ATCC	98.69
HS 578T	89.49
BT-549	59.75
T-47D	30.94
MDA-MB-468	19.19
Mean	75.09
Delta	55.90
Range	103.56

Mean Growth Percent - Growth Percent

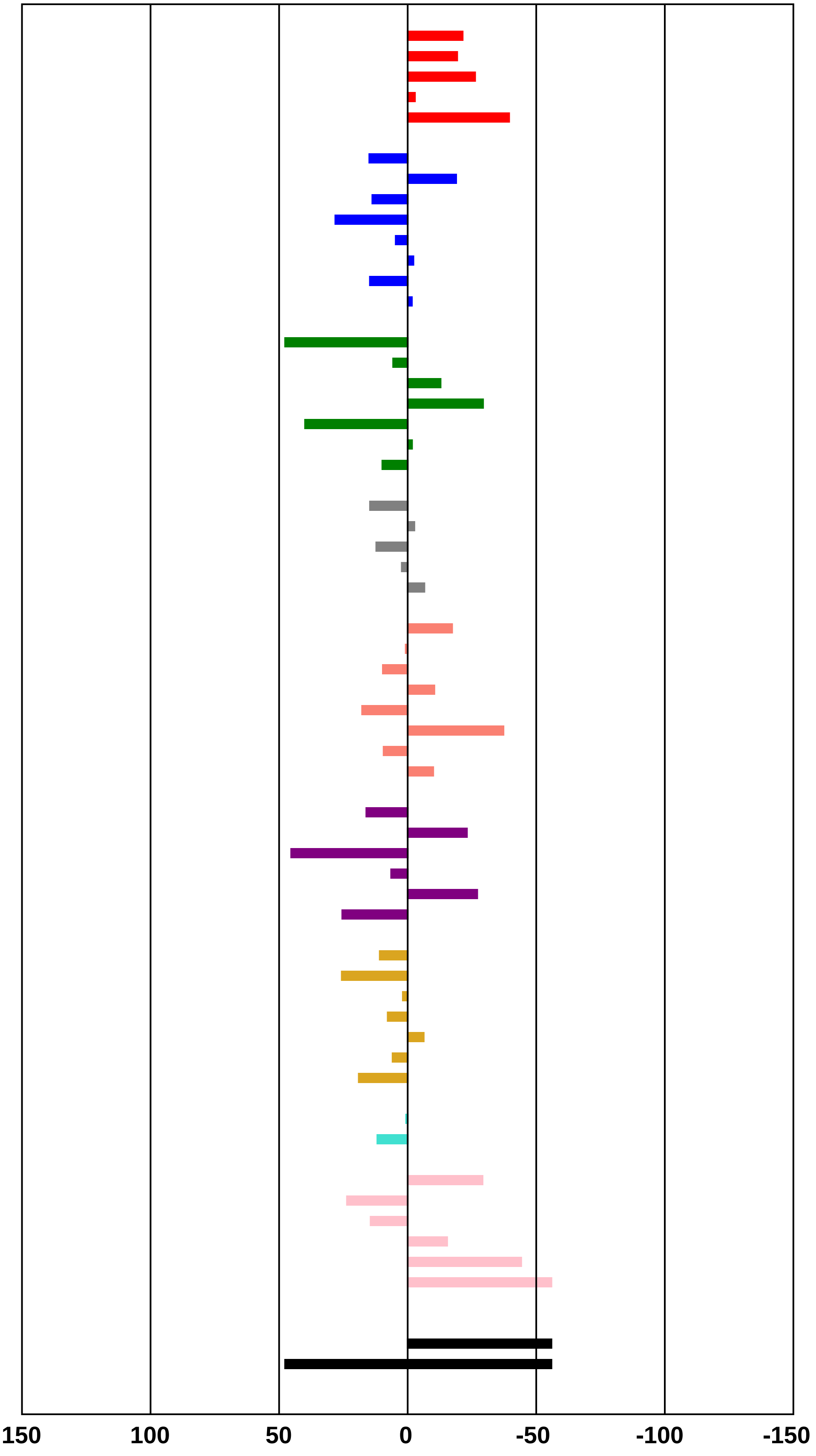

Developmental Therapeutics Program

One Dose Mean Graph

**NSC:** D-841679 / 1	**Conc:** 1.00E-5 Molar	**Test Date:** Jan 23, 2023	
**Experiment ID:** 2301OS90		**Report Date:** Mar 01, 2023	

Panel/Cell Line	Growth Percent
**Leukemia**	
CCRF-CEM	15.53
HL-60(TB)	35.60
K-562	32.43
MOLT-4	73.82
RPMI-8226	18.58
SR	32.51
**Non-Small Cell Lung Cancer**	
A549/ATCC	64.57
EKVX	54.16
HOP-62	34.07
HOP-92	83.73
NCI-H226	80.50
NCI-H23	65.13
NCI-H322M	63.43
NCI-H460	40.01
NCI-H522	56.80
**Colon Cancer**	
COLO 205	115.96
HCC-2998	41.71
HCT-116	34.55
HCT-15	31.33
HT29	97.72
KM12	42.25
SW-620	34.92
**CNS Cancer**	
SF-268	67.22
SF-295	66.50
SF-539	29.82
SNB-19	61.91
SNB-75	58.12
U251	33.72
**Melanoma**	
LOX IMVI	26.92
MALME-3M	89.57
M14	82.23
MDA-MB-435	38.89
SK-MEL-2	68.60
SK-MEL-28	83.19
SK-MEL-5	63.12
UACC-257	71.40
UACC-62	63.85
**Ovarian Cancer**	
IGROV1	69.47
OVCAR-3	3.22
OVCAR-4	47.63
OVCAR-5	131.45
OVCAR-8	34.30
NCI/ADR-RES	26.85
SK-OV-3	76.93
**Renal Cancer**	
786-0	60.00
A498	125.58
ACHN	64.88
CAKI-1	42.04
RXF 393	48.08
SN12C	45.37
TK-10	75.43
UO-31	49.17
**Prostate Cancer**	
PC-3	68.88
DU-145	48.10
**Breast Cancer**	
MCF7	28.86
MDA-MB-231/ATCC	58.36
HS 578T	84.71
BT-549	18.94
T-47D	44.15
MDA-MB-468	-0.42
Mean	55.11
Delta	55.53
Range	131.87

Mean Growth Percent - Growth Percent

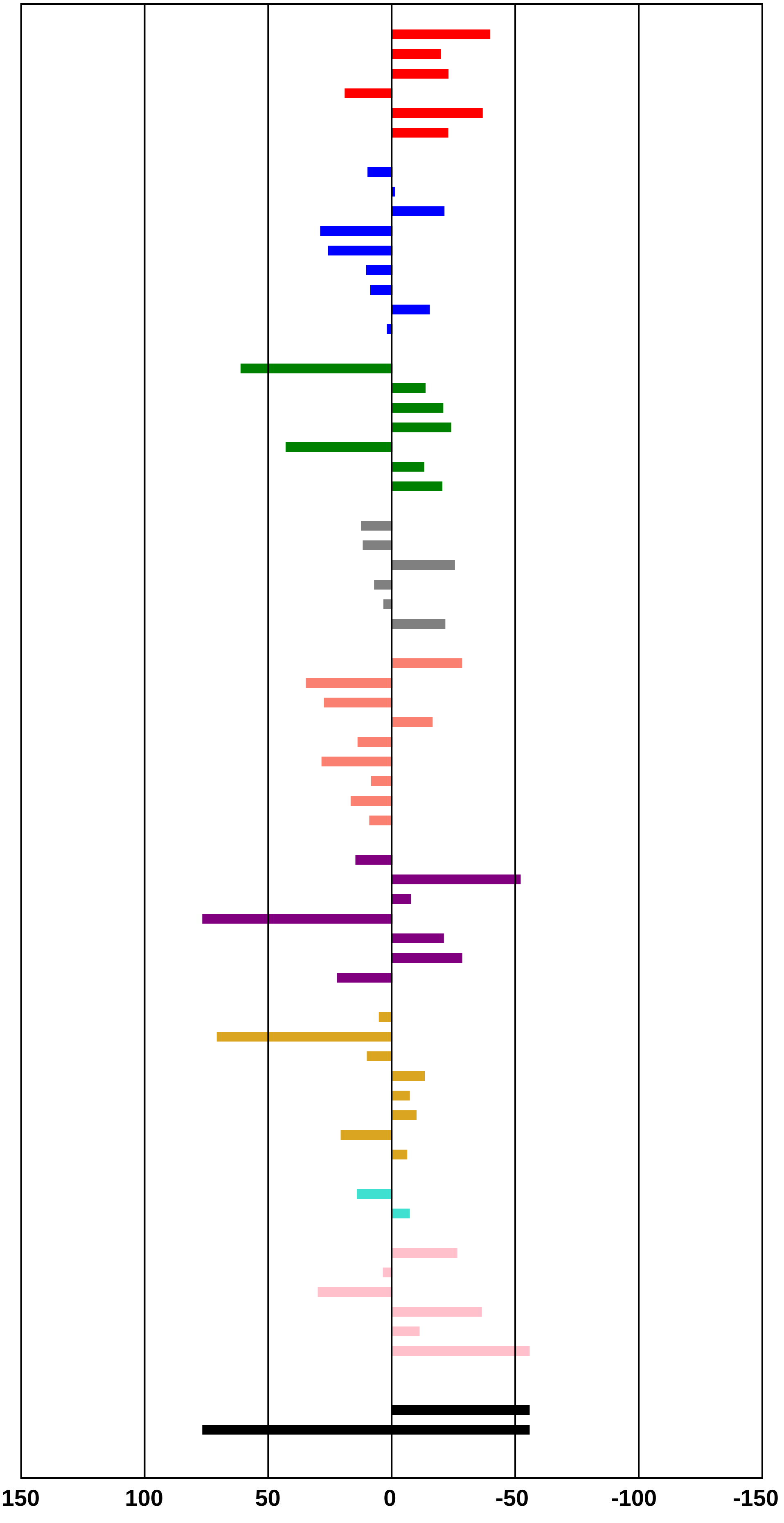

Developmental Therapeutics Program

One Dose Mean Graph

**NSC:** D-845697 / 1	**Conc:** 1.00E-5 Molar	**Test Date:** Jun 12, 2023
**Experiment ID:** 2306OS36		**Report Date:** Jul 23, 2023

Panel/Cell Line	Growth Percent
Leukemia	
CCRF-CEM	4.92
HL-60(TB)	3.95
K-562	8.21
MOLT-4	9.11
RPMI-8226	5.31
Non-Small Cell Lung Cancer	
A549/ATCC	26.57
EKVX	23.40
HOP-62	4.61
HOP-92	16.45
NCI-H226	16.43
NCI-H23	7.27
NCI-H460	-1.34
NCI-H522	16.30
Colon Cancer	
COLO 205	81.66
HCC-2998	19.01
HCT-116	6.81
HCT-15	-3.26
HT29	40.09
KM12	9.52
SW-620	6.92
CNS Cancer	
SF-268	18.50
SF-295	42.28
SF-539	-13.90
SNB-19	19.56
U251	14.98
Melanoma	
LOX IMVI	17.13
M14	15.43
MDA-MB-435	10.98
SK-MEL-2	34.94
SK-MEL-28	24.24
SK-MEL-5	-64.57
UACC-257	42.08
UACC-62	-3.11
Ovarian Cancer	
OVCAR-3	-25.23
OVCAR-4	15.60
OVCAR-5	6.56
OVCAR-8	4.32
NCI/ADR-RES	0.58
SK-OV-3	31.19
Renal Cancer	
786-0	21.09
A498	96.50
ACHN	19.58
CAKI-1	28.19
RXF 393	30.28
SN12C	7.30
TK-10	52.99
Prostate Cancer	
PC-3	35.71
DU-145	12.86
Breast Cancer	
MCF7	9.35
MDA-MB-231/ATCC	2.51
HS 578T	3.94
BT-549	-11.78
T-47D	-1.59
MDA-MB-468	3.79
Mean	14.89
Delta	79.46
Range	161.07

Mean Growth Percent - Growth Percent

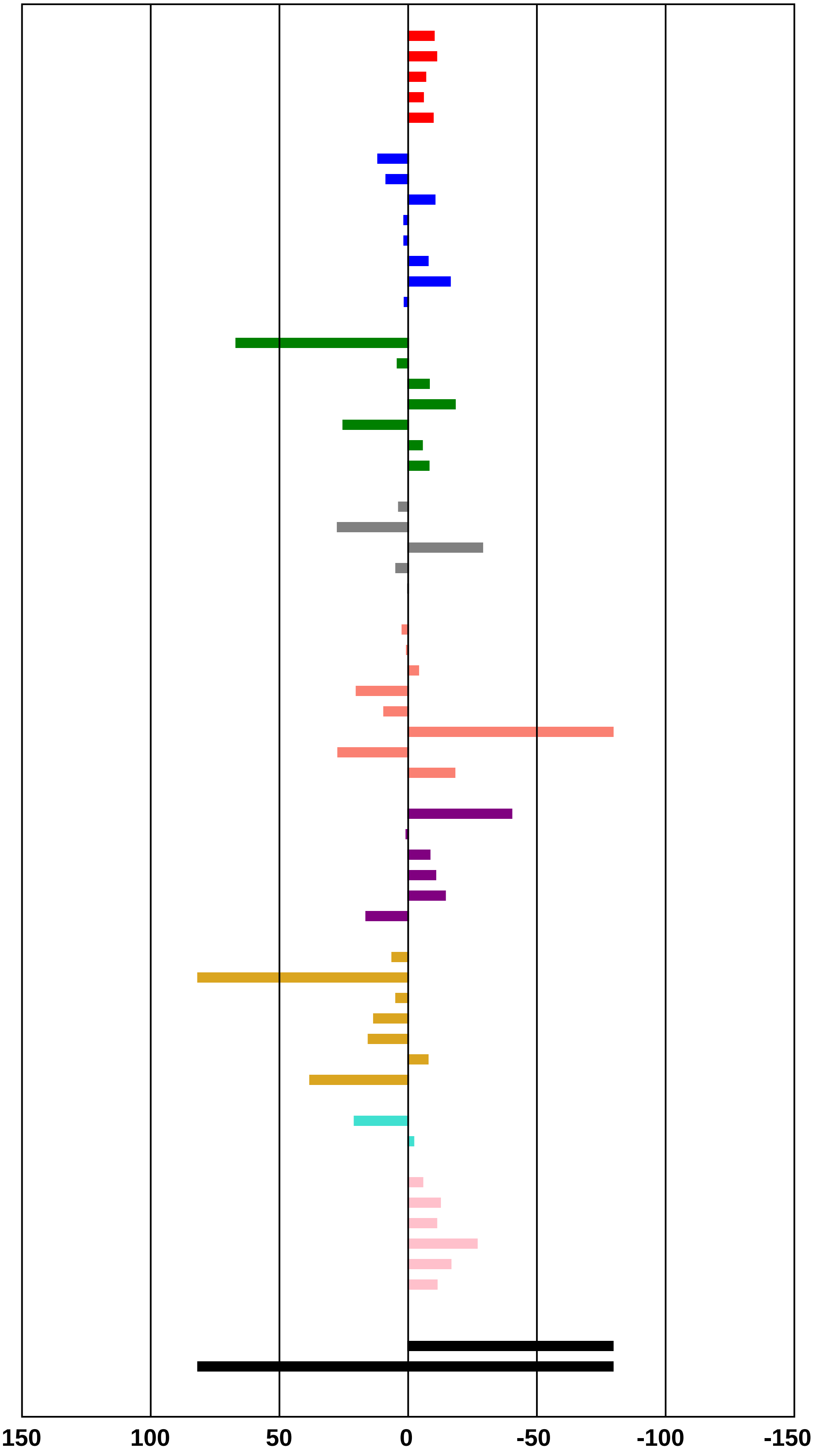

Screening of anticancer activity at five doses

Compound 2c = NSC: D-841672

Compound 2e = NSC: D-846303

Compound 2f = NSC: D- 845698

Compound 2g = NSC: D- 841678

Compound 2h = NSC: D- 846306

Compound 2i = NSC: D- 841675

Compound 2p = NSC: D- 845697

National Cancer Institute Developmental Therapeutics Program Mean Graphs	NSC : D - 841672/1	Units :Molar	SSPL :1CIU	EXP. ID :2306NS34
	Report Date :July 23, 2023		Test Date :June 12, 2023	

Panel/Cell Line	Log_{10}GI50	Log_{10}TGI	Log_{10}LC50
**Leukemia**			
CCRF-CEM	-5.43	> -4.00	> -4.00
HL-60(TB)	-5.55	-4.49	> -4.00
K-562	-5.49	-4.31	> -4.00
MOLT-4	-4.78	-4.19	> -4.00
RPMI-8226	-5.54	> -4.00	> -4.00
SR	-5.50	> -4.00	> -4.00
**Non-Small Cell Lung Cancer**			
A549/ATCC	-4.91	-4.57	-4.24
EKVX	-4.89	-4.54	-4.19
HOP-62	-5.42	-4.79	-4.31
HOP-92	-5.47	-4.80	-4.25
NCI-H226	-5.28	-4.56	> -4.00
NCI-H23	-4.98	-4.64	-4.30
NCI-H322M	-5.39	-4.75	-4.35
NCI-H460	-4.82	-4.45	-4.08
NCI-H522	-5.06	-4.67	-4.33
**Colon Cancer**			
COLO 205	-4.81	-4.45	-4.09
HCC-2998	-5.46	-4.88	-4.44
HCT-116	-5.40	-4.82	-4.37
HCT-15	-5.49	-4.85	-4.36
HT29	-4.98	-4.63	-4.28
KM12	-5.37	-4.81	-4.39
SW-620	-5.47	-4.83	-4.31
**CNS Cancer**			
SF-268	-4.89	-4.54	-4.20
SF-295	-4.90	-4.59	-4.28
SF-539	-5.53	-4.97	-4.45
SNB-19	-5.31	-4.75	-4.37
U251	-5.44	-4.82	-4.38
**Melanoma**			
LOX IMVI	-5.49	-4.90	-4.44
MALME-3M	-4.91	-4.56	-4.22
M14	-5.16	-4.65	-4.25
MDA-MB-435	-5.37	-4.76	-4.31
SK-MEL-2	-4.94	-4.62	-4.30
SK-MEL-28	-4.93	-4.61	-4.29
SK-MEL-5	-5.17	-4.71	-4.35
UACC-257	-4.90	-4.57	-4.25
UACC-62	-5.28	-4.71	-4.34
**Ovarian Cancer**			
IGROV1	-5.31	-4.71	-4.21
OVCAR-3	-5.62	-5.17	-4.61
OVCAR-4	-5.22	-4.71	-4.35
OVCAR-5	-4.81	-4.52	-4.24
OVCAR-8	-5.40	-4.79	-4.29
NCI/ADR-RES	-5.23	-4.60	-4.07
SK-OV-3	-4.96	-4.58	-4.21
**Renal Cancer**			
786-0	-5.34	-4.75	-4.33
A498	-4.87	-4.57	-4.28
ACHN	-5.15	-4.71	-4.35
CAKI-1	-5.16	-4.69	-4.34
RXF 393	-5.52	-4.89	-4.43
SN12C	-5.40	-4.77	-4.33
TK-10	-4.90	-4.60	-4.29
UO-31	-5.54	-4.81	-4.38
**Prostate Cancer**			
PC-3	-5.23	-4.53	> -4.00
DU-145	-5.36	-4.80	-4.40
**Breast Cancer**			
MCF7	-5.58	-4.94	-4.43
MDA-MB-231/ATCC	-4.80	-4.49	-4.18
HS 578T	-4.88	-4.40	> -4.00
BT-549	-5.63	-5.08	-4.48
T-47D	-5.40	-4.54	> -4.00
MDA-MB-468	-5.71	-5.19	-4.60
_MID	-5.23	-4.65	-4.26
Delta	0.48	0.54	0.35
Range	0.93	1.19	0.61

Graph columns: GI50, TGI, LC50; axis scale: +3 +2 +1 0 -1 -2 -3

National Cancer Institute Developmental Therapeutics Program
In-Vitro Screening Data Review Checklist

**NSC:** D - 846303 / 1	**Experiment ID:** 2310NS93
**Test Date:** October 16, 2023	**Review Date:** November 29, 2023

Pending Action by the NCI for this experiment

1. ___X___ None
2. _______ Repeat testing in the Primary Screen
3. _______ Refer to Biological Evaluation Committee
4. _______ Currently under Review by Biological Evaluation Committee

National Cancer Institute Developmental Therapeutics Program
In-Vitro Testing Results

NSC : D - 846303 / 1	Experiment ID : 2310NS93	Test Type : 08	Units : Molar
Report Date : May 19, 2024	Test Date : October 16, 2023	QNS :	MC :
COMI : T10	Stain Reagent : SRB Dual-Pass Related	SSPL : 1CXQ	

			Log10 Concentration												
	Time		Mean Optical Densities					Percent Growth							
Panel/Cell Line	Zero	Ctrl	-8.0	-7.0	-6.0	-5.0	-4.0	-8.0	-7.0	-6.0	-5.0	-4.0	GI50	TGI	LC50
**Leukemia**															
CCRF-CEM	0.490	2.299	2.323	2.229	2.295	0.585	0.396	101	96	100	5	-19	3.36E-6	1.64E-5	> 1.00E-4
HL-60(TB)	0.608	2.555	2.451	2.487	2.500	1.044	0.531	95	96	97	22	-13	4.27E-6	4.34E-5	> 1.00E-4
K-562	0.271	2.193	2.219	2.078	2.307	0.792	0.362	101	94	106	27	5	5.12E-6	> 1.00E-4	> 1.00E-4
MOLT-4	0.497	2.357	2.314	2.294	2.334	1.338	0.508	98	97	99	45	1	8.14E-6	> 1.00E-4	> 1.00E-4
RPMI-8226	0.670	2.386	2.356	2.277	2.201	0.746	0.560	98	94	89	4	-16	2.90E-6	1.63E-5	> 1.00E-4
SR	0.288	1.558	1.437	1.452	1.409	0.461	0.369	90	92	88	14	6	3.25E-6	> 1.00E-4	> 1.00E-4
**Non-Small Cell Lung Cancer**															
A549/ATCC	0.434	2.158	2.055	1.993	2.038	1.399	0.155	94	90	93	56	-64	1.12E-5	2.92E-5	7.61E-5
EKVX	0.695	1.888	1.866	1.800	1.845	1.619	0.050	98	93	96	77	-93	1.45E-5	2.85E-5	5.60E-5
HOP-62	0.592	1.839	1.749	1.724	1.770	0.920	0.123	93	91	94	26	-79	4.49E-6	1.77E-5	5.28E-5
HOP-92	0.989	1.537	1.500	1.445	1.511	1.288	0.220	93	83	95	55	-78	1.08E-5	2.58E-5	6.17E-5
NCI-H226	1.181	2.650	2.631	2.604	2.525	1.772	0.623	99	97	91	40	-47	6.45E-6	2.88E-5	> 1.00E-4
NCI-H23	0.593	2.276	2.257	2.221	2.196	1.682	0.038	99	97	95	65	-94	1.24E-5	2.56E-5	5.30E-5
NCI-H322M	0.756	2.307	2.244	2.233	2.222	1.817	0.064	96	95	95	68	-92	1.30E-5	2.68E-5	5.50E-5
NCI-H460	0.281	2.710	2.768	2.848	2.801	0.983	0.084	102	106	104	29	-70	5.23E-6	1.96E-5	6.24E-5
NCI-H522	0.878	2.528	2.336	2.433	2.367	1.424	0.069	88	94	90	33	-92	5.06E-6	1.84E-5	4.60E-5
**Colon Cancer**															
COLO 205	0.397	1.953	2.004	2.006	2.110	1.552	0.068	103	103	110	74	-83	1.43E-5	2.97E-5	6.17E-5
HCC-2998	0.734	2.671	2.560	2.531	2.581	1.771	0.020	94	93	95	54	-97	1.06E-5	2.26E-5	4.85E-5
HCT-116	0.197	2.057	2.107	1.954	1.884	0.665	0.093	103	94	91	25	-53	4.18E-6	2.10E-5	9.14E-5
HCT-15	0.621	2.697	2.526	2.586	2.436	1.133	0.056	92	95	87	25	-91	3.94E-6	1.63E-5	4.42E-5
HT29	0.294	1.984	1.922	1.992	1.943	0.702	0.065	96	100	98	24	-78	4.44E-6	1.72E-5	5.33E-5
KM12	0.618	2.806	2.796	2.854	2.731	0.768	0.058	100	102	97	7	-91	3.30E-6	1.18E-5	3.83E-5
SW-620	0.274	2.037	2.064	2.026	2.135	0.376	0.073	102	99	106	6	-73	3.60E-6	1.18E-5	5.07E-5
**CNS Cancer**															
SF-268	0.814	2.595	2.435	2.474	2.344	1.756	0.143	91	93	86	53	-82	1.05E-5	2.46E-5	5.76E-5
SF-295	0.925	2.698	2.526	2.489	2.575	2.080	0.029	90	88	93	65	-97	1.24E-5	2.52E-5	5.13E-5
SF-539	0.749	2.320	2.377	2.229	2.244	1.170	0.130	104	94	95	27	-83	4.58E-6	1.76E-5	5.03E-5
SNB-19	0.653	1.881	1.813	1.768	1.818	1.360	0.007	94	91	95	58	-99	1.12E-5	2.33E-5	4.86E-5
SNB-75	1.119	2.104	2.076	2.104	1.992	1.035	0.117	97	100	89	-8	-90	2.52E-6	8.35E-6	3.29E-5
U251	0.418	1.742	1.701	1.595	1.715	0.531	0.022	97	89	98	8	-95	3.43E-6	1.21E-5	3.69E-5
**Melanoma**															
LOX IMVI	0.317	2.034	1.983	1.951	1.892	0.796	0.018	97	95	92	28	-94	4.50E-6	1.69E-5	4.33E-5
MALME-3M	0.636	1.493	1.534	1.487	1.554	1.249	0.071	105	99	107	72	-89	1.36E-5	2.79E-5	5.73E-5
M14	0.360	1.636	1.642	1.596	1.584	1.202	0.091	100	97	96	66	-75	1.30E-5	2.94E-5	6.67E-5
MDA-MB-435	0.558	2.532	2.510	2.489	2.435	0.886	0.154	99	98	95	17	-72	3.75E-6	1.54E-5	5.59E-5
SK-MEL-2	1.711	3.170	3.133	3.078	3.077	2.543	0.201	97	94	94	57	-88	1.12E-5	2.47E-5	5.45E-5
SK-MEL-28	0.725	2.121	2.082	1.973	2.072	1.703	0.022	97	89	97	70	-97	1.32E-5	2.63E-5	5.23E-5
SK-MEL-5	0.822	3.055	3.004	2.923	2.949	1.791	0.007	98	94	95	43	-99	7.45E-6	2.01E-5	4.52E-5
UACC-257	1.052	2.525	2.405	2.350	2.446	1.879	0.068	92	88	95	56	-94	1.10E-5	2.37E-5	5.12E-5
UACC-62	0.769	2.629	2.382	2.292	2.276	1.410	0.031	87	82	81	34	-96	4.64E-6	1.84E-5	4.44E-5
**Ovarian Cancer**															
IGROV1	0.522	2.087	2.052	2.097	2.267	0.906	0.111	98	101	111	25	-79	5.10E-6	1.73E-5	5.27E-5
OVCAR-3	0.582	1.880	1.903	1.790	1.813	0.529	0.009	102	93	95	-9	-98	2.70E-6	8.17E-6	2.87E-5
OVCAR-4	0.788	2.339	2.355	2.336	2.276	1.728	0.102	101	100	96	61	-87	1.18E-5	2.57E-5	5.61E-5
OVCAR-5	0.509	1.315	1.273	1.243	1.313	1.170	0.016	95	91	100	82	-97	1.51E-5	2.87E-5	5.47E-5
OVCAR-8	0.372	1.942	1.989	1.923	1.901	0.347	0.057	103	99	97	-7	-85	2.85E-6	8.60E-6	3.58E-5
NCI/ADR-RES	0.508	1.832	1.900	1.747	1.743	1.140	0.270	105	94	93	48	-47	8.93E-6	3.19E-5	> 1.00E-4
SK-OV-3	0.648	1.644	1.674	1.660	1.658	1.432	0.078	103	102	101	79	-88	1.49E-5	2.96E-5	5.91E-5
**Renal Cancer**															
786-0	0.607	2.603	2.580	2.492	2.558	1.824	0.100	99	94	98	61	-84	1.19E-5	2.64E-5	5.86E-5
A498	1.468	2.417	2.430	2.365	2.356	2.381	0.057	101	95	94	96	-96	1.74E-5	3.16E-5	5.76E-5
ACHN	0.435	1.990	1.959	2.022	1.990	1.269	-0.001	98	102	100	54	-100	1.06E-5	2.23E-5	4.73E-5
CAKI-1	1.050	2.706	2.641	2.542	2.630	1.993	0.172	96	90	95	57	-84	1.12E-5	2.54E-5	5.76E-5
RXF 393	1.108	1.820	1.836	1.788	1.740	1.414	0.061	102	95	89	43	-94	7.00E-6	2.05E-5	4.74E-5
SN12C	0.516	1.907	1.798	1.788	1.840	0.683	0.011	92	91	95	12	-98	3.49E-6	1.28E-5	3.66E-5
TK-10	0.961	2.063	1.859	1.805	1.883	1.828	0.040	81	77	84	79	-96	1.46E-5	2.82E-5	5.46E-5
UO-31	0.716	2.162	2.007	1.964	1.928	1.296	0.066	89	86	84	40	-91	5.94E-6	2.02E-5	4.88E-5
**Prostate Cancer**															
PC-3	0.618	2.218	2.157	2.113	2.159	1.166	0.142	96	93	96	34	-77	5.57E-6	2.03E-5	5.72E-5
DU-145	0.276	1.233	1.316	1.204	1.230	0.227	0.013	109	97	100	-18	-95	2.64E-6	7.04E-6	2.59E-5
**Breast Cancer**															
MCF7	1.238	3.172	3.119	3.061	3.026	1.391	0.029	97	94	92	8	-98	3.18E-6	1.19E-5	3.54E-5
MDA-MB-231/ATCC	0.549	1.183	1.178	1.141	1.193	1.030	0.043	99	93	102	76	-92	1.43E-5	2.83E-5	5.61E-5
HS 578T	1.446	2.448	2.385	2.300	2.399	2.166	1.057	94	85	95	72	-27	1.66E-5	5.34E-5	> 1.00E-4
BT-549	1.082	2.299	2.196	2.181	2.209	1.576	0.141	92	90	93	41	-87	6.59E-6	2.08E-5	5.13E-5
T-47D	0.545	1.402	1.359	1.263	1.283	0.789	0.215	95	84	86	28	-61	4.22E-6	2.08E-5	7.59E-5
MDA-MB-468	1.122	2.636	2.699	2.663	2.619	0.949	0.111	104	102	99	-15	-90	2.68E-6	7.32E-6	2.90E-5

National Cancer Institute Developmental Therapeutics Program Dose Response Curves	NSC: D - 846303 / 1	SSPL: 1CXQ	EXP. ID: 2310NS93
	Report Date: May 19, 2024	Test Date: October 16, 2023	

Leukemia

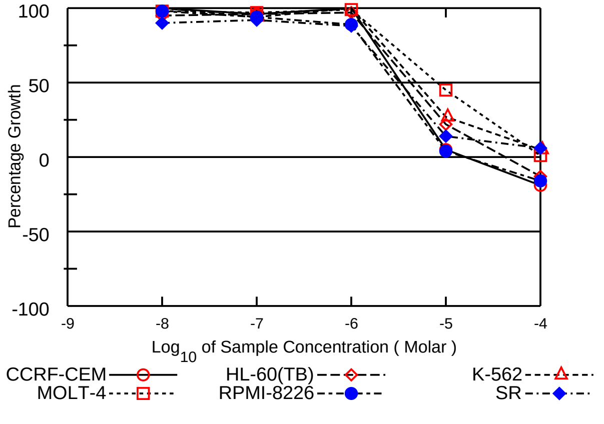

Non-Small Cell Lung Cancer

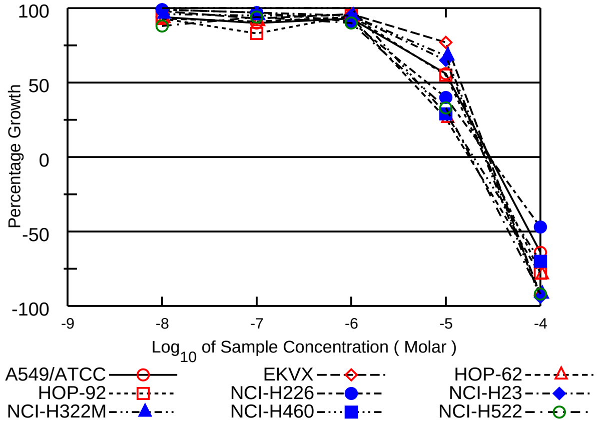

Colon Cancer

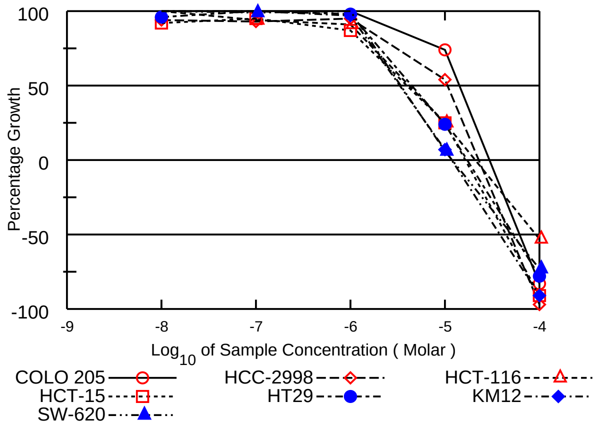

CNS Cancer

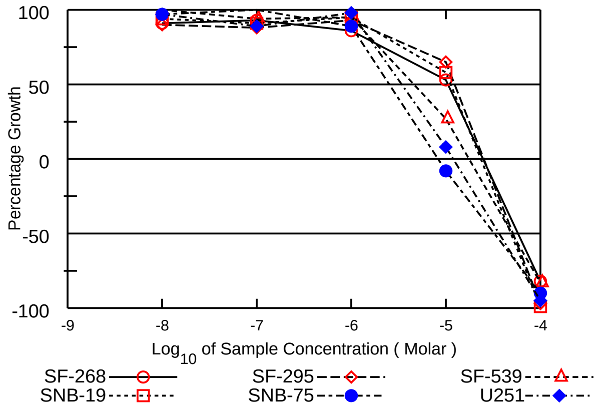

Melanoma

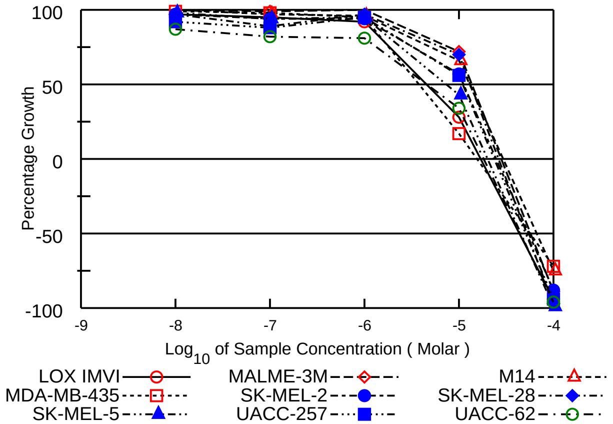

Ovarian Cancer

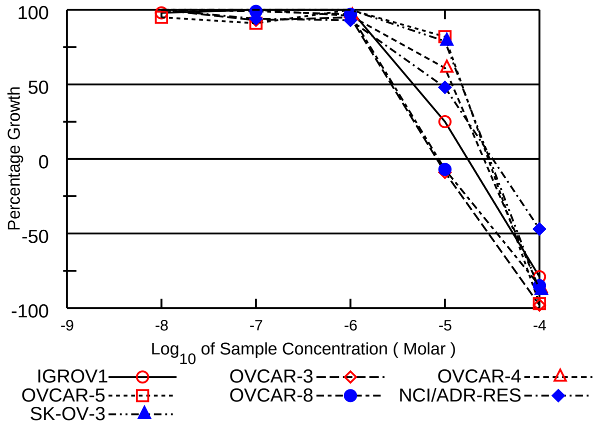

Renal Cancer

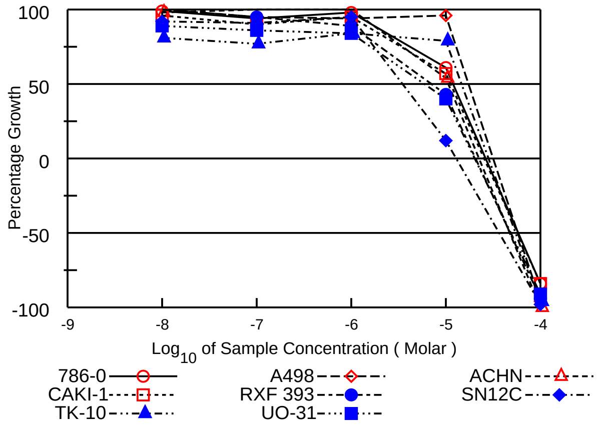

Prostate Cancer

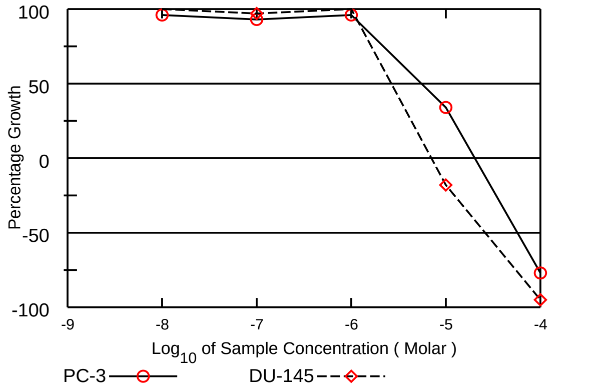

Breast Cancer

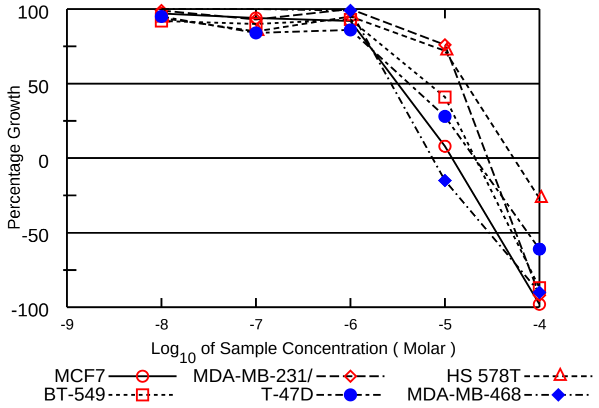

National Cancer Institute Developmental Therapeutics Program
Mean Graphs

NSC : D - 846303/1	Units :Molar	SSPL :1CXQ	EXP. ID :2310NS93
Report Date :May 19, 2024		Test Date :October 16, 2023	

Panel/Cell Line	Log_{10}GI50	Log_{10}TGI	Log_{10}LC50
**Leukemia**			
CCRF-CEM	-5.47	-4.79	> -4.00
HL-60(TB)	-5.37	-4.36	> -4.00
K-562	-5.29	> -4.00	> -4.00
MOLT-4	-5.09	> -4.00	> -4.00
RPMI-8226	-5.54	-4.79	> -4.00
SR	-5.49	> -4.00	> -4.00
**Non-Small Cell Lung Cancer**			
A549/ATCC	-4.95	-4.53	-4.12
EKVX	-4.84	-4.55	-4.25
HOP-62	-5.35	-4.75	-4.28
HOP-92	-4.97	-4.59	-4.21
NCI-H226	-5.19	-4.54	> -4.00
NCI-H23	-4.91	-4.59	-4.28
NCI-H322M	-4.89	-4.57	-4.26
NCI-H460	-5.28	-4.71	-4.20
NCI-H522	-5.30	-4.74	-4.34
**Colon Cancer**			
COLO 205	-4.85	-4.53	-4.21
HCC-2998	-4.98	-4.65	-4.31
HCT-116	-5.38	-4.68	-4.04
HCT-15	-5.40	-4.79	-4.35
HT29	-5.35	-4.76	-4.27
KM12	-5.48	-4.93	-4.42
SW-620	-5.44	-4.93	-4.30
**CNS Cancer**			
SF-268	-4.98	-4.61	-4.24
SF-295	-4.91	-4.60	-4.29
SF-539	-5.34	-4.76	-4.30
SNB-19	-4.95	-4.63	-4.31
SNB-75	-5.60	-5.08	-4.48
U251	-5.46	-4.92	-4.43
**Melanoma**			
LOX IMVI	-5.35	-4.77	-4.36
MALME-3M	-4.87	-4.55	-4.24
M14	-4.89	-4.53	-4.18
MDA-MB-435	-5.43	-4.81	-4.25
SK-MEL-2	-4.95	-4.61	-4.26
SK-MEL-28	-4.88	-4.58	-4.28
SK-MEL-5	-5.13	-4.70	-4.35
UACC-257	-4.96	-4.63	-4.29
UACC-62	-5.33	-4.74	-4.35
**Ovarian Cancer**			
IGROV1	-5.29	-4.76	-4.28
OVCAR-3	-5.57	-5.09	-4.54
OVCAR-4	-4.93	-4.59	-4.25
OVCAR-5	-4.82	-4.54	-4.26
OVCAR-8	-5.55	-5.07	-4.45
NCI/ADR-RES	-5.05	-4.50	> -4.00
SK-OV-3	-4.83	-4.53	-4.23
**Renal Cancer**			
786-0	-4.92	-4.58	-4.23
A498	-4.76	-4.50	-4.24
ACHN	-4.98	-4.65	-4.33
CAKI-1	-4.95	-4.60	-4.24
RXF 393	-5.15	-4.69	-4.32
SN12C	-5.46	-4.89	-4.44
TK-10	-4.84	-4.55	-4.26
UO-31	-5.23	-4.69	-4.31
**Prostate Cancer**			
PC-3	-5.25	-4.69	-4.24
DU-145	-5.58	-5.15	-4.59
**Breast Cancer**			
MCF7	-5.50	-4.93	-4.45
MDA-MB-231/ATCC	-4.85	-4.55	-4.25
HS 578T	-4.78	-4.27	> -4.00
BT-549	-5.18	-4.68	-4.29
T-47D	-5.37	-4.68	-4.12
MDA-MB-468	-5.57	-5.14	-4.54
_MID	-5.17	-4.66	-4.26
Delta	0.43	0.49	0.33
Range	0.84	1.15	0.59

GI50 / TGI / LC50 axes: +3 +2 +1 0 -1 -2 -3

National Cancer Institute Developmental Therapeutics Program Dose Response Curves	NSC : D - 846303/1	SSPL:1CXQ	EXP. ID:2310NS93
	Report Date:May 19, 2024	Test Date:October 16, 2023	

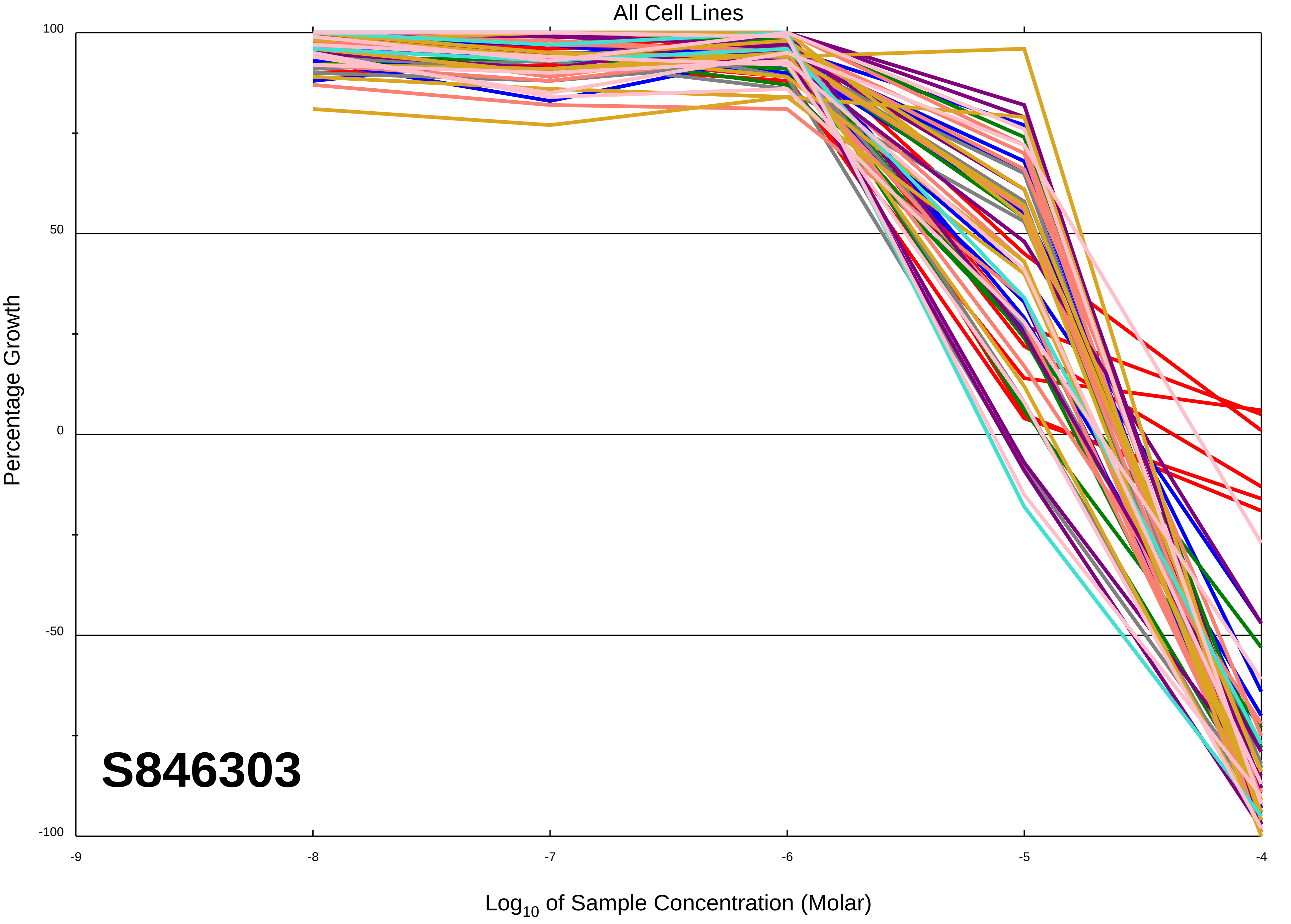

National Cancer Institute Developmental Therapeutics Program
Waterfall Graph GI50

NSC : D - 846303/1	Units :Molar	SSPL :1CXQ	EXP. ID :2310NS93
Report Date :May 19, 2024		Test Date :October 16, 2023	

Panel	Cell Name	Hollow Fiber	GI50
CNS Cancer	SNB-75		-5.60
Prostate Cancer	DU-145		-5.58
Breast Cancer	MDA-MB-468		-5.57
Ovarian Cancer	OVCAR-3	*	-5.57
Ovarian Cancer	OVCAR-8		-5.55
Leukemia	RPMI-8226		-5.54
Breast Cancer	MCF7		-5.50
Leukemia	SR		-5.49
Colon Cancer	KM12		-5.48
Leukemia	CCRF-CEM		-5.47
CNS Cancer	U251	*	-5.46
Renal Cancer	SN12C		-5.46
Colon Cancer	SW-620	*	-5.44
Melanoma	MDA-MB-435	*	-5.43
Colon Cancer	HCT-15		-5.40
Colon Cancer	HCT-116		-5.38
Breast Cancer	T-47D		-5.37
Leukemia	HL-60(TB)		-5.37
Colon Cancer	HT29		-5.35
Non-Small Cell Lung Cancer	HOP-62		-5.35
Melanoma	LOX IMVI	*	-5.35
CNS Cancer	SF-539		-5.34
Melanoma	UACC-62	*	-5.33
Non-Small Cell Lung Cancer	NCI-H522	*	-5.30
Ovarian Cancer	IGROV1		-5.29
Leukemia	K-562		-5.29
Non-Small Cell Lung Cancer	NCI-H460		-5.28
Prostate Cancer	PC-3		-5.25
Renal Cancer	UO-31		-5.23
Non-Small Cell Lung Cancer	NCI-H226		-5.19
Breast Cancer	BT-549		-5.18
Renal Cancer	RXF 393		-5.15
Melanoma	SK-MEL-5		-5.13
Leukemia	MOLT-4		-5.09
Ovarian Cancer	NCI/ADR-RES		-5.05
CNS Cancer	SF-268		-4.98
Colon Cancer	HCC-2998		-4.98
Renal Cancer	ACHN		-4.98
Non-Small Cell Lung Cancer	HOP-92		-4.97
Melanoma	UACC-257		-4.96
CNS Cancer	SNB-19		-4.95
Melanoma	SK-MEL-2		-4.95
Renal Cancer	CAKI-1		-4.95
Non-Small Cell Lung Cancer	A549/ATCC		-4.95
Ovarian Cancer	OVCAR-4		-4.93
Renal Cancer	786-0		-4.92
Non-Small Cell Lung Cancer	NCI-H23	*	-4.91
CNS Cancer	SF-295	*	-4.91
Melanoma	M14		-4.89
Non-Small Cell Lung Cancer	NCI-H322M		-4.89
Melanoma	SK-MEL-28		-4.88
Melanoma	MALME-3M		-4.87
Breast Cancer	MDA-MB-231/ATCC	*	-4.85
Colon Cancer	COLO 205	*	-4.85
Non-Small Cell Lung Cancer	EKVX		-4.84
Renal Cancer	TK-10		-4.84
Ovarian Cancer	SK-OV-3		-4.83
Ovarian Cancer	OVCAR-5	*	-4.82
Breast Cancer	HS 578T		-4.78
Renal Cancer	A498		-4.76

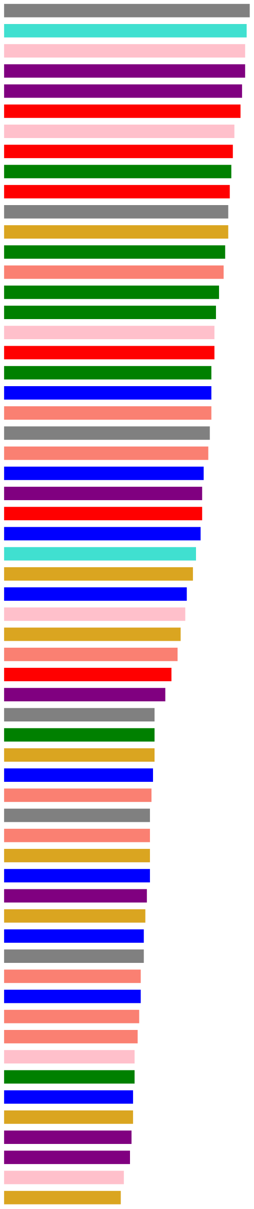

Log10 High Conc :-4.0

National Cancer Institute Developmental Therapeutics Program<br>Waterfall Graph TGI	NSC : D - 846303/1	Units :Molar	SSPL :1CXQ	EXP. ID :2310NS93
	Report Date :May 19, 2024		Test Date :October 16, 2023	

Panel	Cell Name	Hollow Fiber	TGI
Prostate Cancer	DU-145		-5.15
Breast Cancer	MDA-MB-468		-5.14
Ovarian Cancer	OVCAR-3	*	-5.09
CNS Cancer	SNB-75		-5.08
Ovarian Cancer	OVCAR-8		-5.07
Colon Cancer	KM12		-4.93
Colon Cancer	SW-620	*	-4.93
Breast Cancer	MCF7		-4.93
CNS Cancer	U251	*	-4.92
Renal Cancer	SN12C		-4.89
Melanoma	MDA-MB-435	*	-4.81
Leukemia	RPMI-8226		-4.79
Colon Cancer	HCT-15		-4.79
Leukemia	CCRF-CEM		-4.79
Melanoma	LOX IMVI	*	-4.77
Colon Cancer	HT29		-4.76
Ovarian Cancer	IGROV1		-4.76
CNS Cancer	SF-539		-4.76
Non-Small Cell Lung Cancer	HOP-62		-4.75
Non-Small Cell Lung Cancer	NCI-H522	*	-4.74
Melanoma	UACC-62	*	-4.74
Non-Small Cell Lung Cancer	NCI-H460		-4.71
Melanoma	SK-MEL-5		-4.70
Renal Cancer	UO-31		-4.69
Prostate Cancer	PC-3		-4.69
Renal Cancer	RXF 393		-4.69
Breast Cancer	BT-549		-4.68
Breast Cancer	T-47D		-4.68
Colon Cancer	HCT-116		-4.68
Renal Cancer	ACHN		-4.65
Colon Cancer	HCC-2998		-4.65
CNS Cancer	SNB-19		-4.63
Melanoma	UACC-257		-4.63
CNS Cancer	SF-268		-4.61
Melanoma	SK-MEL-2		-4.61
CNS Cancer	SF-295	*	-4.60
Renal Cancer	CAKI-1		-4.60
Non-Small Cell Lung Cancer	NCI-H23	*	-4.59
Ovarian Cancer	OVCAR-4		-4.59
Non-Small Cell Lung Cancer	HOP-92		-4.59
Melanoma	SK-MEL-28		-4.58
Renal Cancer	786-0		-4.58
Non-Small Cell Lung Cancer	NCI-H322M		-4.57
Melanoma	MALME-3M		-4.55
Renal Cancer	TK-10		-4.55
Breast Cancer	MDA-MB-231/ATCC	*	-4.55
Non-Small Cell Lung Cancer	EKVX		-4.55
Ovarian Cancer	OVCAR-5	*	-4.54
Non-Small Cell Lung Cancer	NCI-H226		-4.54
Non-Small Cell Lung Cancer	A549/ATCC		-4.53
Melanoma	M14		-4.53
Ovarian Cancer	SK-OV-3		-4.53
Colon Cancer	COLO 205	*	-4.53
Renal Cancer	A498		-4.50
Ovarian Cancer	NCI/ADR-RES		-4.50
Leukemia	HL-60(TB)		-4.36
Breast Cancer	HS 578T		-4.27
Leukemia	K-562		> -4.00
Leukemia	MOLT-4		> -4.00
Leukemia	SR		> -4.00

Log10 High Conc : -4.0

National Cancer Institute Developmental Therapeutics Program<br>Waterfall Graph LC50	NSC : D - 846303/1	Units :Molar	SSPL :1CXQ	EXP. ID :2310NS93
	Report Date :May 19, 2024		Test Date :October 16, 2023	

Panel	Cell Name	Hollow Fiber	LC50
Prostate Cancer	DU-145		-4.59
Ovarian Cancer	OVCAR-3	*	-4.54
Breast Cancer	MDA-MB-468		-4.54
CNS Cancer	SNB-75		-4.48
Breast Cancer	MCF7		-4.45
Ovarian Cancer	OVCAR-8		-4.45
Renal Cancer	SN12C		-4.44
CNS Cancer	U251	*	-4.43
Colon Cancer	KM12		-4.42
Melanoma	LOX IMVI	*	-4.36
Colon Cancer	HCT-15		-4.35
Melanoma	UACC-62	*	-4.35
Melanoma	SK-MEL-5		-4.35
Non-Small Cell Lung Cancer	NCI-H522	*	-4.34
Renal Cancer	ACHN		-4.33
Renal Cancer	RXF 393		-4.32
Colon Cancer	HCC-2998		-4.31
CNS Cancer	SNB-19		-4.31
Renal Cancer	UO-31		-4.31
CNS Cancer	SF-539		-4.30
Colon Cancer	SW-620	*	-4.30
Melanoma	UACC-257		-4.29
Breast Cancer	BT-549		-4.29
CNS Cancer	SF-295	*	-4.29
Melanoma	SK-MEL-28		-4.28
Ovarian Cancer	IGROV1		-4.28
Non-Small Cell Lung Cancer	HOP-62		-4.28
Non-Small Cell Lung Cancer	NCI-H23	*	-4.28
Colon Cancer	HT29		-4.27
Melanoma	SK-MEL-2		-4.26
Renal Cancer	TK-10		-4.26
Ovarian Cancer	OVCAR-5	*	-4.26
Non-Small Cell Lung Cancer	NCI-H322M		-4.26
Melanoma	MDA-MB-435	*	-4.25
Non-Small Cell Lung Cancer	EKVX		-4.25
Breast Cancer	MDA-MB-231/ATCC	*	-4.25
Ovarian Cancer	OVCAR-4		-4.25
Prostate Cancer	PC-3		-4.24
Melanoma	MALME-3M		-4.24
Renal Cancer	A498		-4.24
CNS Cancer	SF-268		-4.24
Renal Cancer	CAKI-1		-4.24
Renal Cancer	786-0		-4.23
Ovarian Cancer	SK-OV-3		-4.23
Non-Small Cell Lung Cancer	HOP-92		-4.21
Colon Cancer	COLO 205	*	-4.21
Non-Small Cell Lung Cancer	NCI-H460		-4.20
Melanoma	M14		-4.18
Breast Cancer	T-47D		-4.12
Non-Small Cell Lung Cancer	A549/ATCC		-4.12
Colon Cancer	HCT-116		-4.04
Leukemia	CCRF-CEM		> -4.00
Leukemia	HL-60(TB)		> -4.00
Leukemia	K-562		> -4.00
Leukemia	MOLT-4		> -4.00
Leukemia	RPMI-8226		> -4.00
Leukemia	SR		> -4.00
Non-Small Cell Lung Cancer	NCI-H226		> -4.00
Ovarian Cancer	NCI/ADR-RES		> -4.00
Breast Cancer	HS 578T		> -4.00

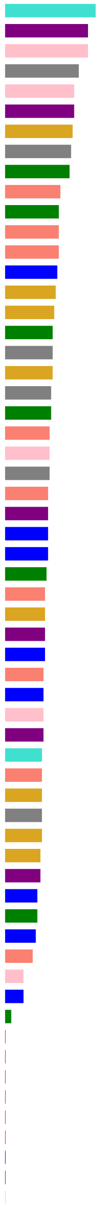

Log10 High Conc : -4.0

National Cancer Institute Developmental Therapeutics Program
In-Vitro Screening Data Review Checklist

**NSC:** D - 845698 / 1	**Experiment ID:** 2310NS90
**Test Date:** October 10, 2023	**Review Date:** November 22, 2023

Pending Action by the NCI for this experiment

1. __X__ None
2. ______ Repeat testing in the Primary Screen
3. ______ Refer to Biological Evaluation Committee
4. ______ Currently under Review by Biological Evaluation Committee

National Cancer Institute Developmental Therapeutics Program
In-Vitro Testing Results

NSC : D - 845698 / 1	Experiment ID : 2310NS90	Test Type : 08	Units : Molar
Report Date : November 23, 2023	Test Date : October 10, 2023	QNS :	MC :
COMI : T12	Stain Reagent : SRB Dual-Pass Related	SSPL : 1CIU	

Panel/Cell Line	Time Zero	Ctrl	Mean Optical Densities -8.0	-7.0	-6.0	-5.0	-4.0	Percent Growth -8.0	-7.0	-6.0	-5.0	-4.0	GI50	TGI	LC50
**Leukemia**															
CCRF-CEM	0.316	1.595	1.575	1.530	1.491	0.333	0.234	98	95	92	1	-26	2.90E-6	1.12E-5	> 1.00E-4
HL-60(TB)	0.474	2.165	2.014	1.977	1.910	0.832	0.402	91	89	85	21	-15	3.53E-6	3.82E-5	> 1.00E-4
K-562	0.135	1.180	1.201	1.146	1.150	0.320	0.164	102	97	97	18	3	3.92E-6	> 1.00E-4	> 1.00E-4
MOLT-4	0.532	2.550	2.463	2.422	2.375	1.764	0.498	96	94	91	61	-6	1.46E-5	8.02E-5	> 1.00E-4
RPMI-8226	0.801	2.460	2.504	2.405	2.223	0.742	0.747	103	97	86	-7	-7	2.42E-6	8.32E-6	> 1.00E-4
SR	0.510	2.132	2.037	2.019	2.107	0.678	0.493	94	93	98	10	-3	3.55E-6	5.64E-5	> 1.00E-4
**Non-Small Cell Lung Cancer**															
A549/ATCC	0.316	1.712	1.603	1.598	1.598	1.078	0.032	92	92	92	55	-90	1.07E-5	2.38E-5	5.29E-5
EKVX	0.746	2.117	2.075	1.950	2.044	1.687	0.034	97	88	95	69	-96	1.30E-5	2.62E-5	5.28E-5
HOP-62	0.472	1.696	1.768	1.571	1.551	0.910	0.171	106	90	88	36	-64	5.35E-6	2.29E-5	7.27E-5
HOP-92	1.030	1.732	1.635	1.629	1.635	1.442	0.232	86	85	86	59	-77	1.16E-5	2.70E-5	6.28E-5
NCI-H226	1.191	2.534	2.418	2.368	2.319	2.075	0.634	91	88	84	66	-47	1.38E-5	3.84E-5	> 1.00E-4
NCI-H23	0.671	2.266	2.236	2.161	2.156	1.913	0.080	98	93	93	78	-88	1.47E-5	2.95E-5	5.90E-5
NCI-H322M	0.838	2.269	2.218	2.187	2.264	1.582	0.071	96	94	100	52	-92	1.03E-5	2.30E-5	5.14E-5
NCI-H460	0.323	1.898	1.884	1.873	1.857	1.277	0.094	99	98	97	61	-71	1.20E-5	2.89E-5	6.93E-5
NCI-H522	0.964	2.656	2.546	2.543	2.487	2.001	0.065	94	93	90	61	-93	1.18E-5	2.49E-5	5.25E-5
**Colon Cancer**															
COLO 205	0.530	2.127	2.165	2.143	2.188	2.100	0.145	102	101	104	98	-73	1.92E-5	3.76E-5	7.37E-5
HCT-116	0.194	2.637	2.573	2.432	2.478	1.188	0.071	97	92	93	41	-64	6.66E-6	2.45E-5	7.40E-5
HCT-15	0.361	2.594	2.456	2.343	2.392	0.888	0.059	94	89	91	24	-84	4.06E-6	1.66E-5	4.86E-5
HT29	0.203	1.152	1.184	1.223	1.222	0.565	0.036	103	107	107	38	-82	6.73E-6	2.07E-5	5.39E-5
KM12	0.662	2.357	2.209	2.479	2.218	1.072	0.080	91	107	92	24	-88	4.15E-6	1.64E-5	4.59E-5
SW-620	0.309	1.469	1.385	1.435	1.384	0.368	0.037	93	97	93	5	-88	3.07E-6	1.13E-5	3.90E-5
**CNS Cancer**															
SF-268	0.932	2.427	2.200	2.281	2.213	1.851	0.221	85	90	86	61	-76	1.21E-5	2.79E-5	6.44E-5
SF-295	1.028	3.004	2.776	2.687	2.642	2.742	0.026	88	84	82	87	-98	1.58E-5	2.96E-5	5.52E-5
SF-539	0.944	2.567	2.645	2.464	2.531	1.611	0.071	105	94	98	41	-93	6.96E-6	2.03E-5	4.81E-5
SNB-19	0.787	2.240	2.192	2.105	2.078	1.711	0.005	97	91	89	64	-99	1.21E-5	2.46E-5	4.98E-5
SNB-75	1.063	1.887	1.830	1.804	1.755	0.884	0.123	93	90	84	-17	-88	2.17E-6	6.81E-6	2.90E-5
U251	0.232	1.188	1.188	1.137	1.121	0.348	0.026	100	95	93	12	-89	3.40E-6	1.32E-5	4.11E-5
**Melanoma**															
LOX IMVI	0.425	2.334	2.247	2.174	2.274	0.578	0.035	95	92	97	8	-92	3.37E-6	1.20E-5	3.81E-5
MALME-3M	0.650	1.535	1.497	1.405	1.504	1.292	0.219	96	85	96	73	-66	1.45E-5	3.33E-5	7.63E-5
M14	0.408	1.715	1.756	1.686	1.682	1.328	0.127	103	98	97	70	-69	1.40E-5	3.20E-5	7.32E-5
MDA-MB-435	0.636	2.377	2.321	2.326	2.299	1.234	0.072	97	97	96	34	-89	5.55E-6	1.90E-5	4.84E-5
SK-MEL-2	1.172	2.459	2.439	2.446	2.362	2.115	0.111	98	99	92	73	-91	1.39E-5	2.80E-5	5.65E-5
SK-MEL-28	0.693	2.146	2.078	2.044	2.031	1.835	0.020	95	93	92	79	-97	1.45E-5	2.80E-5	5.39E-5
SK-MEL-5	1.062	3.041	3.014	2.792	2.958	2.205	0.011	99	87	96	58	-99	1.12E-5	2.34E-5	4.87E-5
UACC-257	1.151	2.607	2.511	2.469	2.505	2.220	0.091	93	90	93	73	-92	1.38E-5	2.78E-5	5.57E-5
UACC-62	0.704	2.463	2.280	2.138	2.136	1.579	0.040	90	82	81	50	-94	9.80E-6	2.21E-5	4.92E-5
**Ovarian Cancer**															
IGROV1	0.591	1.991	1.993	2.115	2.257	1.489	0.274	100	109	119	64	-54	1.32E-5	3.50E-5	9.31E-5
OVCAR-3	0.858	2.252	2.162	2.238	2.089	0.898	0.016	94	99	88	3	-98	2.81E-6	1.07E-5	3.34E-5
OVCAR-4	0.794	2.294	2.236	2.237	2.171	1.879	0.064	96	96	92	72	-92	1.37E-5	2.75E-5	5.55E-5
OVCAR-5	0.643	1.487	1.406	1.368	1.383	1.472	0.054	90	86	88	98	-92	1.80E-5	3.29E-5	6.04E-5
OVCAR-8	0.495	2.160	2.106	2.012	2.041	1.274	0.079	97	91	93	47	-84	8.50E-6	2.28E-5	5.48E-5
NCI/ADR-RES	0.372	1.300	1.297	1.209	1.226	0.545	0.155	100	90	92	19	-58	3.74E-6	1.75E-5	7.77E-5
SK-OV-3	0.624	1.618	1.697	1.643	1.760	1.436	0.132	108	103	114	82	-79	1.58E-5	3.23E-5	6.61E-5
**Renal Cancer**															
786-0	0.440	2.444	2.322	2.195	2.120	1.108	0.113	94	88	84	33	-74	4.68E-6	2.04E-5	5.93E-5
A498	1.307	2.097	2.063	2.022	1.989	1.992	0.048	96	91	86	87	-96	1.59E-5	2.98E-5	5.58E-5
ACHN	0.426	1.812	1.843	1.815	1.835	1.453	.	102	100	102	74	-100	1.38E-5	2.66E-5	5.16E-5
CAKI-1	0.462	1.819	1.692	1.683	1.648	0.863	0.021	91	90	87	30	-96	4.43E-6	1.72E-5	4.32E-5
RXF 393	1.053	1.680	1.664	1.637	1.626	1.290	0.103	97	93	91	38	-90	5.93E-6	1.97E-5	4.85E-5
SN12C	0.552	2.121	1.998	1.916	1.871	1.097	0.015	92	87	84	35	-97	4.90E-6	1.83E-5	4.38E-5
TK-10	0.990	2.169	2.056	2.050	1.984	1.960	0.024	90	90	84	82	-98	1.51E-5	2.87E-5	5.44E-5
UO-31	0.704	2.198	2.045	1.956	1.909	1.156	0.087	90	84	81	30	-88	4.05E-6	1.80E-5	4.79E-5
**Prostate Cancer**															
PC-3	0.414	1.761	1.769	1.706	1.678	0.873	0.171	101	96	94	34	-59	5.41E-6	2.33E-5	8.06E-5
DU-145	0.339	1.319	1.325	1.309	1.265	0.631	0.013	101	99	94	30	-96	4.87E-6	1.72E-5	4.29E-5
**Breast Cancer**															
MCF7	0.420	2.035	1.888	1.845	1.846	0.608	0.068	91	88	88	12	-84	3.16E-6	1.32E-5	4.41E-5
MDA-MB-231/ATCC	0.549	1.194	1.177	1.122	1.172	1.111	0.072	97	89	97	87	-87	1.63E-5	3.16E-5	6.13E-5
HS 578T	1.661	2.661	2.514	2.436	2.564	2.458	1.496	85	78	90	80	-10	2.14E-5	7.74E-5	> 1.00E-4
BT-549	1.156	2.279	2.297	2.158	2.223	1.556	0.258	102	89	95	36	-78	5.73E-6	2.06E-5	5.70E-5
T-47D	0.631	1.485	1.399	1.409	1.331	0.825	0.271	90	91	82	23	-57	3.46E-6	1.93E-5	8.16E-5
MDA-MB-468	1.202	2.070	2.029	1.931	1.963	1.115	0.055	95	84	88	-7	-95	2.50E-6	8.38E-6	3.05E-5

Log10 Concentration

National Cancer Institute Developmental Therapeutics Program<br>Dose Response Curves	NSC: D - 845698 / 1	SSPL: 1CIU	EXP. ID: 2310NS90
	Report Date: November 23, 2023	Test Date: October 10, 2023	

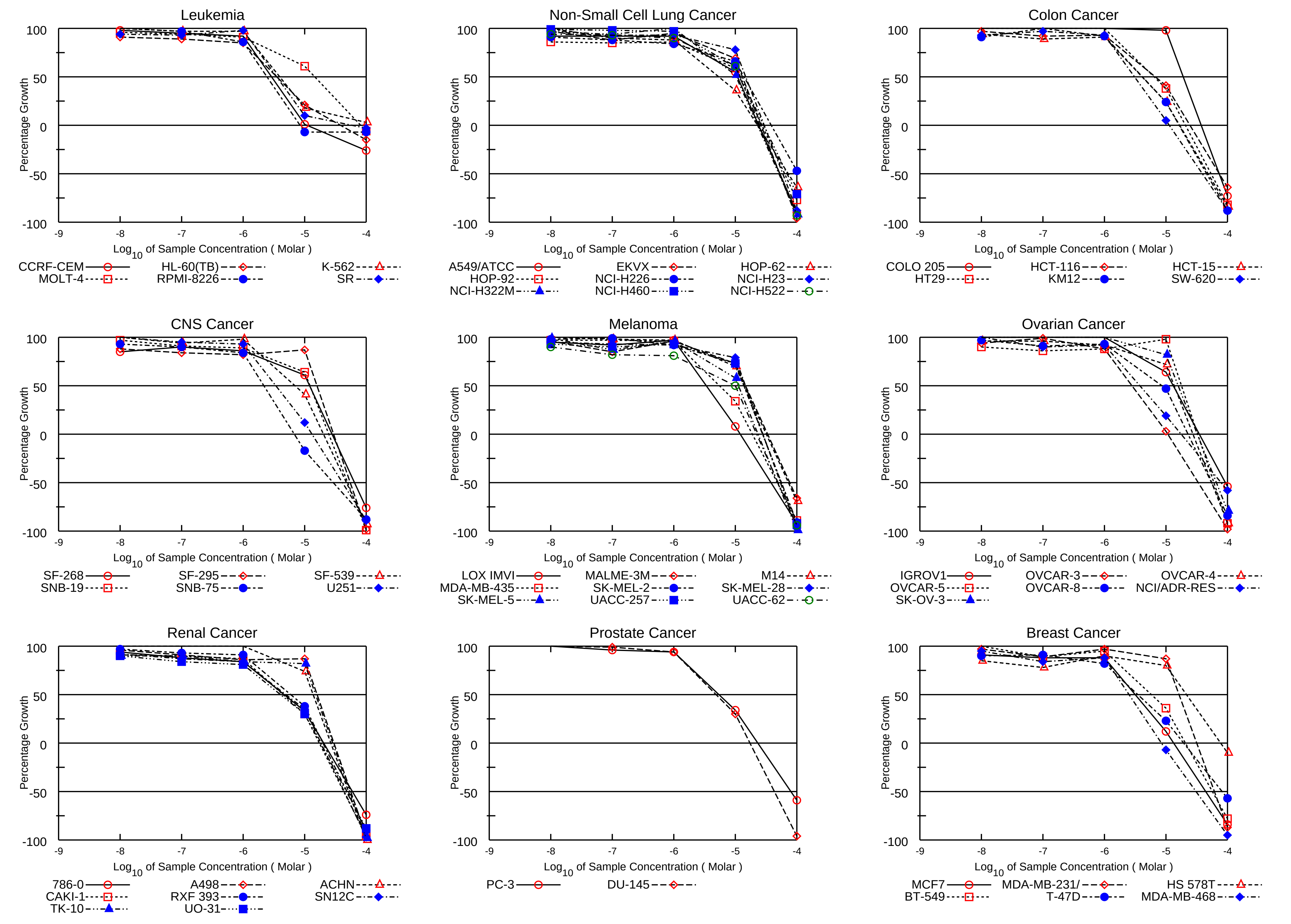

National Cancer Institute Developmental Therapeutics Program Mean Graphs	NSC : D - 845698/1	Units :Molar	SSPL :1CIU	EXP. ID :2310NS90
	Report Date :November 23, 2023		Test Date :October 10, 2023	

Panel/Cell Line	Log_{10}GI50	Log_{10}TGI	Log_{10}LC50
**Leukemia**			
CCRF-CEM	-5.54	-4.95	> -4.00
HL-60(TB)	-5.45	-4.42	> -4.00
K-562	-5.41	> -4.00	> -4.00
MOLT-4	-4.84	-4.10	> -4.00
RPMI-8226	-5.62	-5.08	> -4.00
SR	-5.45	-4.25	> -4.00
**Non-Small Cell Lung Cancer**			
A549/ATCC	-4.97	-4.62	-4.28
EKVX	-4.89	-4.58	-4.28
HOP-62	-5.27	-4.64	-4.14
HOP-92	-4.94	-4.57	-4.20
NCI-H226	-4.86	-4.42	> -4.00
NCI-H23	-4.83	-4.53	-4.23
NCI-H322M	-4.99	-4.64	-4.29
NCI-H460	-4.92	-4.54	-4.16
NCI-H522	-4.93	-4.60	-4.28
**Colon Cancer**			
COLO 205	-4.72	-4.42	-4.13
HCT-116	-5.18	-4.61	-4.13
HCT-15	-5.39	-4.78	-4.31
HT29	-5.17	-4.68	-4.27
KM12	-5.38	-4.78	-4.34
SW-620	-5.51	-4.95	-4.41
**CNS Cancer**			
SF-268	-4.92	-4.55	-4.19
SF-295	-4.80	-4.53	-4.26
SF-539	-5.16	-4.69	-4.32
SNB-19	-4.92	-4.61	-4.30
SNB-75	-5.66	-5.17	-4.54
U251	-5.47	-4.88	-4.39
**Melanoma**			
LOX IMVI	-5.47	-4.92	-4.42
MALME-3M	-4.84	-4.48	-4.12
M14	-4.85	-4.49	-4.14
MDA-MB-435	-5.26	-4.72	-4.31
SK-MEL-2	-4.86	-4.55	-4.25
SK-MEL-28	-4.84	-4.55	-4.27
SK-MEL-5	-4.95	-4.63	-4.31
UACC-257	-4.86	-4.56	-4.25
UACC-62	-5.01	-4.65	-4.31
**Ovarian Cancer**			
IGROV1	-4.88	-4.46	-4.03
OVCAR-3	-5.55	-4.97	-4.48
OVCAR-4	-4.86	-4.56	-4.26
OVCAR-5	-4.75	-4.48	-4.22
OVCAR-8	-5.07	-4.64	-4.26
NCI/ADR-RES	-5.43	-4.76	-4.11
SK-OV-3	-4.80	-4.49	-4.18
**Renal Cancer**			
786-0	-5.33	-4.69	-4.23
A498	-4.80	-4.53	-4.25
ACHN	-4.86	-4.57	-4.29
CAKI-1	-5.35	-4.76	-4.36
RXF 393	-5.23	-4.70	-4.31
SN12C	-5.31	-4.74	-4.36
TK-10	-4.82	-4.54	-4.26
UO-31	-5.39	-4.74	-4.32
**Prostate Cancer**			
PC-3	-5.27	-4.63	-4.09
DU-145	-5.31	-4.76	-4.37
**Breast Cancer**			
MCF7	-5.50	-4.88	-4.36
MDA-MB-231/ATCC	-4.79	-4.50	-4.21
HS 578T	-4.67	-4.11	> -4.00
BT-549	-5.24	-4.69	-4.24
T-47D	-5.46	-4.72	-4.09
MDA-MB-468	-5.60	-5.08	-4.52

	GI50	TGI	LC50
_MID	-5.13	-4.63	-4.23
Delta	0.53	0.54	0.31
Range	0.99	1.17	0.54

GI50

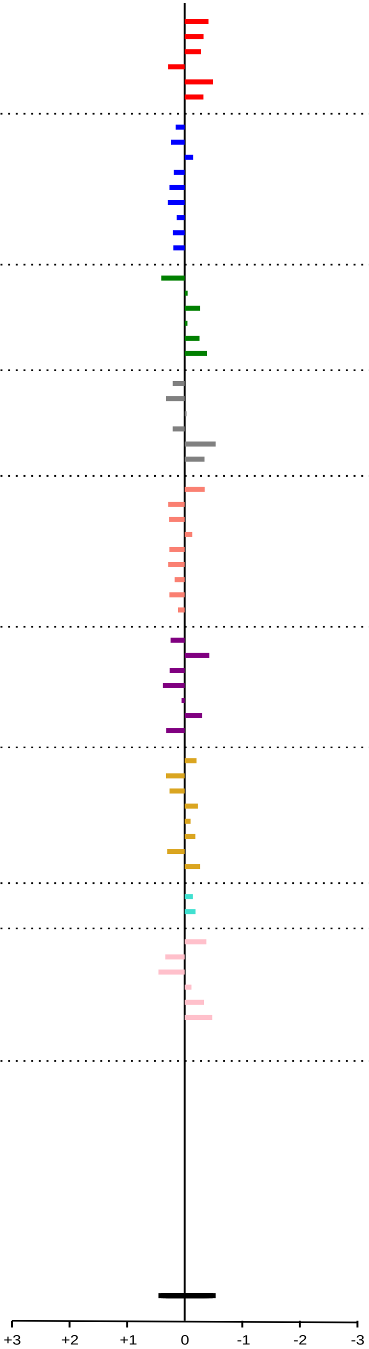

TGI

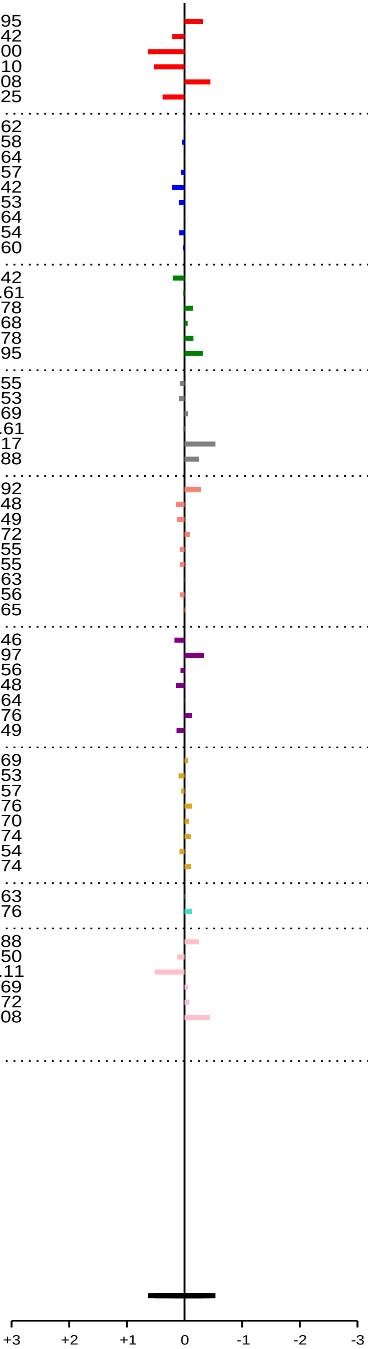

LC50

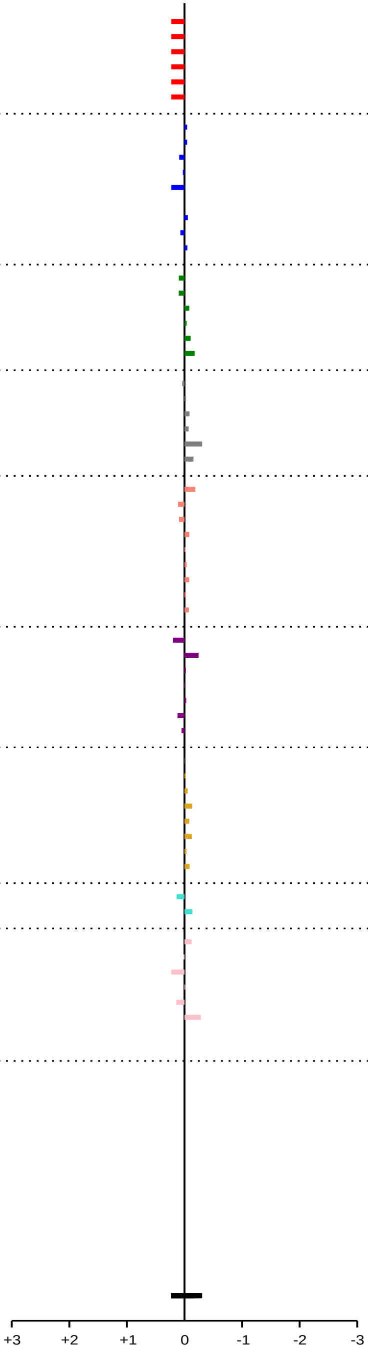

National Cancer Institute Developmental Therapeutics Program<br>Dose Response Curves	NSC : D - 845698/1	SSPL:1CIU	EXP. ID:2310NS90
	Report Date:November 23, 2023	Test Date:October 10, 2023	

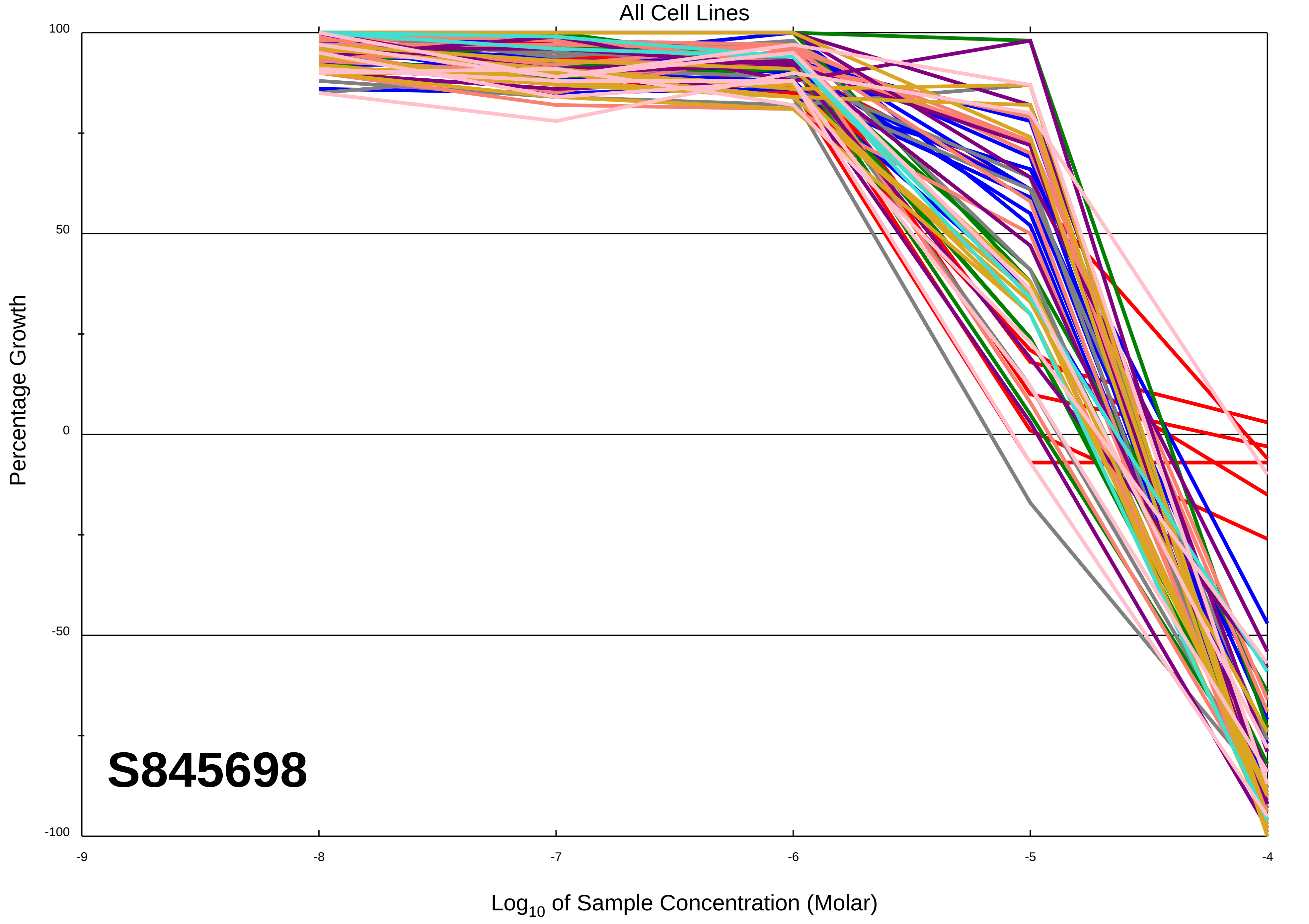

National Cancer Institute Developmental Therapeutics Program<br>Waterfall Graph GI50	NSC : D - 845698/1	Units :Molar	SSPL :1CIU	EXP. ID :2310NS90
	Report Date :November 23, 2023		Test Date :October 10, 2023	

Panel	Cell Name	Hollow Fiber	GI50
CNS Cancer	SNB-75		-5.66
Leukemia	RPMI-8226		-5.62
Breast Cancer	MDA-MB-468		-5.60
Ovarian Cancer	OVCAR-3	*	-5.55
Leukemia	CCRF-CEM		-5.54
Colon Cancer	SW-620	*	-5.51
Breast Cancer	MCF7		-5.50
Melanoma	LOX IMVI	*	-5.47
CNS Cancer	U251	*	-5.47
Breast Cancer	T-47D		-5.46
Leukemia	HL-60(TB)		-5.45
Leukemia	SR		-5.45
Ovarian Cancer	NCI/ADR-RES		-5.43
Leukemia	K-562		-5.41
Renal Cancer	UO-31		-5.39
Colon Cancer	HCT-15		-5.39
Colon Cancer	KM12		-5.38
Renal Cancer	CAKI-1		-5.35
Renal Cancer	786-0		-5.33
Prostate Cancer	DU-145		-5.31
Renal Cancer	SN12C		-5.31
Non-Small Cell Lung Cancer	HOP-62		-5.27
Prostate Cancer	PC-3		-5.27
Melanoma	MDA-MB-435	*	-5.26
Breast Cancer	BT-549		-5.24
Renal Cancer	RXF 393		-5.23
Colon Cancer	HCT-116		-5.18
Colon Cancer	HT29		-5.17
CNS Cancer	SF-539		-5.16
Ovarian Cancer	OVCAR-8		-5.07
Melanoma	UACC-62	*	-5.01
Non-Small Cell Lung Cancer	NCI-H322M		-4.99
Non-Small Cell Lung Cancer	A549/ATCC		-4.97
Melanoma	SK-MEL-5		-4.95
Non-Small Cell Lung Cancer	HOP-92		-4.94
Non-Small Cell Lung Cancer	NCI-H522	*	-4.93
Non-Small Cell Lung Cancer	NCI-H460		-4.92
CNS Cancer	SF-268		-4.92
CNS Cancer	SNB-19		-4.92
Non-Small Cell Lung Cancer	EKVX		-4.89
Ovarian Cancer	IGROV1		-4.88
Ovarian Cancer	OVCAR-4		-4.86
Renal Cancer	ACHN		-4.86
Non-Small Cell Lung Cancer	NCI-H226		-4.86
Melanoma	UACC-257		-4.86
Melanoma	SK-MEL-2		-4.86
Melanoma	M14		-4.85
Melanoma	MALME-3M		-4.84
Melanoma	SK-MEL-28		-4.84
Leukemia	MOLT-4		-4.84
Non-Small Cell Lung Cancer	NCI-H23	*	-4.83
Renal Cancer	TK-10		-4.82
Ovarian Cancer	SK-OV-3		-4.80
CNS Cancer	SF-295	*	-4.80
Renal Cancer	A498		-4.80
Breast Cancer	MDA-MB-231/ATCC	*	-4.79
Ovarian Cancer	OVCAR-5	*	-4.75
Colon Cancer	COLO 205	*	-4.72
Breast Cancer	HS 578T		-4.67

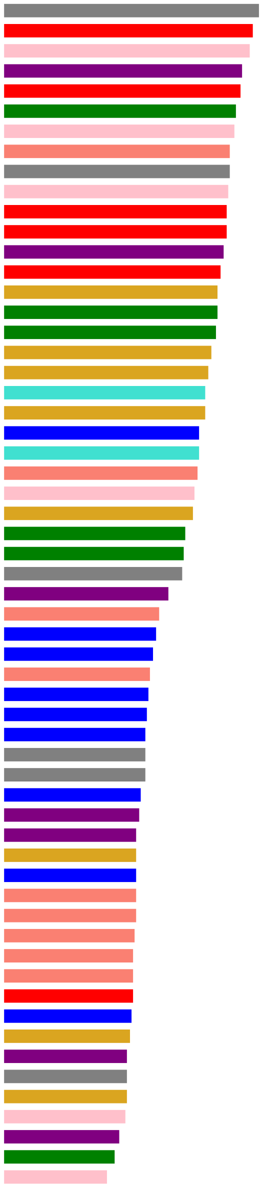

Log10 High Conc :-4.0

National Cancer Institute Developmental Therapeutics Program
Waterfall Graph TGI

NSC : D - 845698/1	Units :Molar	SSPL :1CIU	EXP. ID :2310NS90
Report Date :November 23, 2023		Test Date :October 10, 2023	

Panel	Cell Name	Hollow Fiber	TGI
CNS Cancer	SNB-75		-5.17
Leukemia	RPMI-8226		-5.08
Breast Cancer	MDA-MB-468		-5.08
Ovarian Cancer	OVCAR-3	*	-4.97
Leukemia	CCRF-CEM		-4.95
Colon Cancer	SW-620	*	-4.95
Melanoma	LOX IMVI	*	-4.92
CNS Cancer	U251	*	-4.88
Breast Cancer	MCF7		-4.88
Colon Cancer	KM12		-4.78
Colon Cancer	HCT-15		-4.78
Renal Cancer	CAKI-1		-4.76
Prostate Cancer	DU-145		-4.76
Ovarian Cancer	NCI/ADR-RES		-4.76
Renal Cancer	UO-31		-4.74
Renal Cancer	SN12C		-4.74
Melanoma	MDA-MB-435	*	-4.72
Breast Cancer	T-47D		-4.72
Renal Cancer	RXF 393		-4.70
CNS Cancer	SF-539		-4.69
Renal Cancer	786-0		-4.69
Breast Cancer	BT-549		-4.69
Colon Cancer	HT29		-4.68
Melanoma	UACC-62	*	-4.65
Ovarian Cancer	OVCAR-8		-4.64
Non-Small Cell Lung Cancer	HOP-62		-4.64
Non-Small Cell Lung Cancer	NCI-H322M		-4.64
Prostate Cancer	PC-3		-4.63
Melanoma	SK-MEL-5		-4.63
Non-Small Cell Lung Cancer	A549/ATCC		-4.62
Colon Cancer	HCT-116		-4.61
CNS Cancer	SNB-19		-4.61
Non-Small Cell Lung Cancer	NCI-H522	*	-4.60
Non-Small Cell Lung Cancer	EKVX		-4.58
Renal Cancer	ACHN		-4.57
Non-Small Cell Lung Cancer	HOP-92		-4.57
Ovarian Cancer	OVCAR-4		-4.56
Melanoma	UACC-257		-4.56
CNS Cancer	SF-268		-4.55
Melanoma	SK-MEL-28		-4.55
Melanoma	SK-MEL-2		-4.55
Renal Cancer	TK-10		-4.54
Non-Small Cell Lung Cancer	NCI-H460		-4.54
Non-Small Cell Lung Cancer	NCI-H23	*	-4.53
CNS Cancer	SF-295	*	-4.53
Renal Cancer	A498		-4.53
Breast Cancer	MDA-MB-231/ATCC	*	-4.50
Melanoma	M14		-4.49
Ovarian Cancer	SK-OV-3		-4.49
Ovarian Cancer	OVCAR-5	*	-4.48
Melanoma	MALME-3M		-4.48
Ovarian Cancer	IGROV1		-4.46
Colon Cancer	COLO 205	*	-4.42
Leukemia	HL-60(TB)		-4.42
Non-Small Cell Lung Cancer	NCI-H226		-4.42
Leukemia	SR		-4.25
Breast Cancer	HS 578T		-4.11
Leukemia	MOLT-4		-4.10
Leukemia	K-562		> -4.00

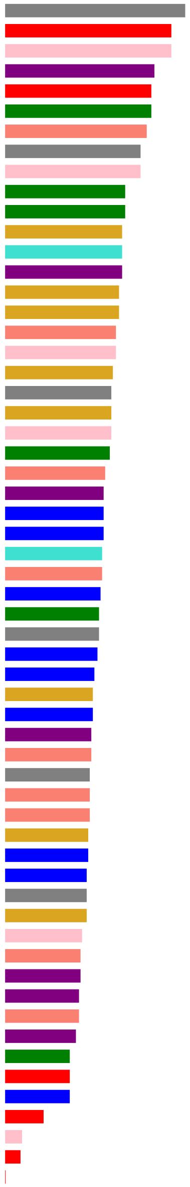

Log10 High Conc : -4.0

National Cancer Institute Developmental Therapeutics Program<br>Waterfall Graph LC50	NSC : D - 845698/1	Units :Molar	SSPL :1CIU	EXP. ID :2310NS90
	Report Date :November 23, 2023		Test Date :October 10, 2023	

Panel	Cell Name	Hollow Fiber	LC50
CNS Cancer	SNB-75		-4.54
Breast Cancer	MDA-MB-468		-4.52
Ovarian Cancer	OVCAR-3	*	-4.48
Melanoma	LOX IMVI	*	-4.42
Colon Cancer	SW-620	*	-4.41
CNS Cancer	U251	*	-4.39
Prostate Cancer	DU-145		-4.37
Renal Cancer	CAKI-1		-4.36
Renal Cancer	SN12C		-4.36
Breast Cancer	MCF7		-4.36
Colon Cancer	KM12		-4.34
Renal Cancer	UO-31		-4.32
CNS Cancer	SF-539		-4.32
Melanoma	MDA-MB-435	*	-4.31
Renal Cancer	RXF 393		-4.31
Colon Cancer	HCT-15		-4.31
Melanoma	SK-MEL-5		-4.31
Melanoma	UACC-62	*	-4.31
CNS Cancer	SNB-19		-4.30
Non-Small Cell Lung Cancer	NCI-H322M		-4.29
Renal Cancer	ACHN		-4.29
Non-Small Cell Lung Cancer	NCI-H522	*	-4.28
Non-Small Cell Lung Cancer	EKVX		-4.28
Non-Small Cell Lung Cancer	A549/ATCC		-4.28
Melanoma	SK-MEL-28		-4.27
Colon Cancer	HT29		-4.27
Renal Cancer	TK-10		-4.26
Ovarian Cancer	OVCAR-8		-4.26
CNS Cancer	SF-295	*	-4.26
Ovarian Cancer	OVCAR-4		-4.26
Melanoma	UACC-257		-4.25
Renal Cancer	A498		-4.25
Melanoma	SK-MEL-2		-4.25
Breast Cancer	BT-549		-4.24
Non-Small Cell Lung Cancer	NCI-H23	*	-4.23
Renal Cancer	786-0		-4.23
Ovarian Cancer	OVCAR-5	*	-4.22
Breast Cancer	MDA-MB-231/ATCC	*	-4.21
Non-Small Cell Lung Cancer	HOP-92		-4.20
CNS Cancer	SF-268		-4.19
Ovarian Cancer	SK-OV-3		-4.18
Non-Small Cell Lung Cancer	NCI-H460		-4.16
Non-Small Cell Lung Cancer	HOP-62		-4.14
Melanoma	M14		-4.14
Colon Cancer	COLO 205	*	-4.13
Colon Cancer	HCT-116		-4.13
Melanoma	MALME-3M		-4.12
Ovarian Cancer	NCI/ADR-RES		-4.11
Prostate Cancer	PC-3		-4.09
Breast Cancer	T-47D		-4.09
Ovarian Cancer	IGROV1		-4.03
Leukemia	CCRF-CEM		> -4.00
Leukemia	HL-60(TB)		> -4.00
Leukemia	K-562		> -4.00
Leukemia	MOLT-4		> -4.00
Leukemia	RPMI-8226		> -4.00
Leukemia	SR		> -4.00
Non-Small Cell Lung Cancer	NCI-H226		> -4.00
Breast Cancer	HS 578T		> -4.00

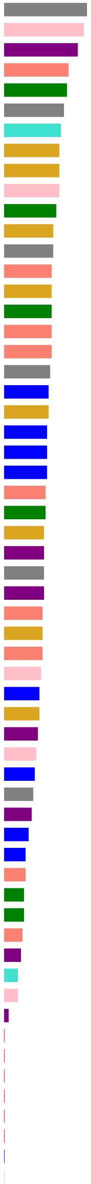

Log10 High Conc : -4.0

National Cancer Institute Developmental Therapeutics Program Mean Graphs	NSC : D - 841678/1	Units :Molar	SSPL :1CIU	EXP. ID :2306NS34
	Report Date :July 23, 2023		Test Date :June 12, 2023	

Panel/Cell Line	Log_{10}GI50	Log_{10}TGI	Log_{10}LC50
**Leukemia**			
CCRF-CEM	-5.51	-4.21	> -4.00
HL-60(TB)	-5.71	-5.00	> -4.00
K-562	-5.56	> -4.00	> -4.00
MOLT-4	-5.45	-4.44	> -4.00
RPMI-8226	-5.64	> -4.00	> -4.00
SR	-5.55	-4.63	> -4.00
**Non-Small Cell Lung Cancer**			
A549/ATCC	-4.93	-4.59	-4.26
EKVX	-4.97	-4.63	-4.29
HOP-62	-5.40	-4.85	-4.21
HOP-92	-5.19	-4.65	-4.23
NCI-H226	-5.38	-4.59	> -4.00
NCI-H23	-5.08	-4.67	-4.32
NCI-H322M	-5.42	-4.82	-4.38
NCI-H460	-4.84	-4.47	-4.09
NCI-H522	-5.47	-4.84	-4.39
**Colon Cancer**			
COLO 205	-4.86	-4.48	-4.10
HCC-2998	-5.57	-5.02	-4.50
HCT-116	-5.42	-4.88	-4.39
HCT-15	-5.58	-5.00	-4.44
HT29	-5.29	-4.71	-4.24
KM12	-5.49	-4.96	-4.44
SW-620	-5.55	-4.93	-4.38
**CNS Cancer**			
SF-268	-4.87	-4.53	-4.19
SF-295	-4.90	-4.59	-4.28
SF-539	-5.70	-5.33	-4.89
SNB-19	-5.46	-4.87	-4.42
U251	-5.54	-4.93	-4.40
**Melanoma**			
LOX IMVI	-5.55	-4.99	-4.47
MALME-3M	-4.90	-4.54	-4.17
M14	-5.04	-4.64	-4.26
MDA-MB-435	-5.39	-4.78	-4.35
SK-MEL-2	-5.23	-4.71	-4.32
SK-MEL-28	-4.92	-4.61	-4.30
SK-MEL-5	-5.24	-4.73	-4.37
UACC-257	-4.95	-4.62	-4.28
UACC-62	-5.29	-4.73	-4.35
**Ovarian Cancer**			
IGROV1	-5.48	-4.93	-4.27
OVCAR-3	-5.73	-5.37	-5.01
OVCAR-4	-4.97	-4.64	-4.31
OVCAR-5	-4.81	-4.53	-4.24
OVCAR-8	-5.54	-5.06	-4.43
NCI/ADR-RES	-5.22	-4.63	-4.13
SK-OV-3	-4.86	-4.49	-4.13
**Renal Cancer**			
786-0	-5.48	-4.88	-4.38
A498	-4.87	-4.58	-4.29
ACHN	-5.12	-4.69	-4.34
CAKI-1	-5.00	-4.66	-4.33
RXF 393	-5.59	-5.03	-4.48
SN12C	-5.50	-4.88	-4.43
TK-10	-5.52	-4.93	-4.45
UO-31	-5.61	-4.87	-4.40
**Prostate Cancer**			
PC-3	-5.33	-4.58	> -4.00
DU-145	-5.45	-4.92	-4.45
**Breast Cancer**			
MCF7	-5.59	-4.98	-4.41
MDA-MB-231/ATCC	-4.96	-4.63	-4.29
HS 578T	-4.98	-4.48	> -4.00
BT-549	-5.58	-5.14	-4.51
T-47D	-5.56	-4.93	-4.21
MDA-MB-468	-5.75	-5.17	-4.56
_MID	-5.31	-4.75	-4.3
Delta	0.44	0.62	0.71
Range	0.94	1.37	1.01

GI50

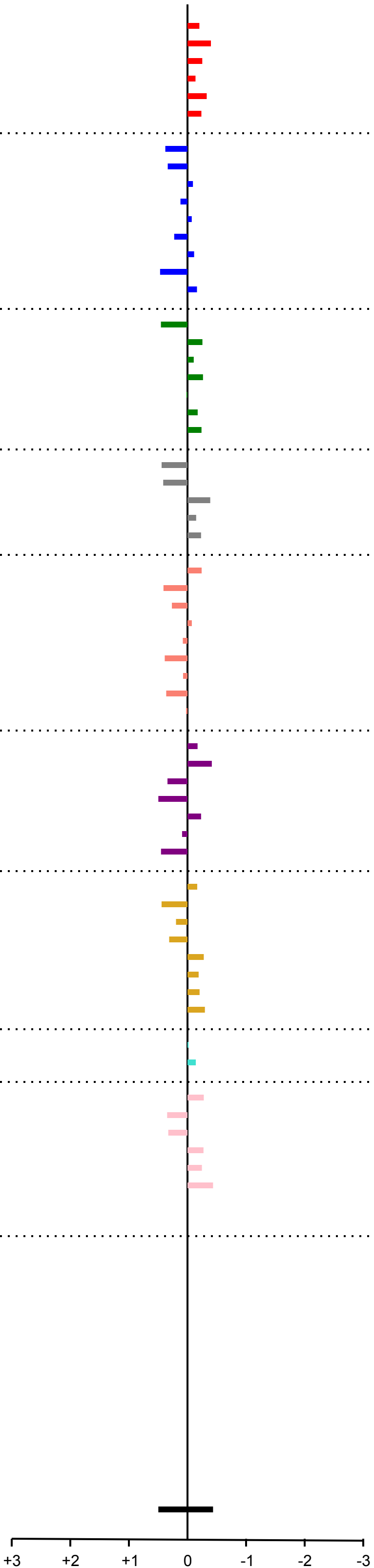

TGI

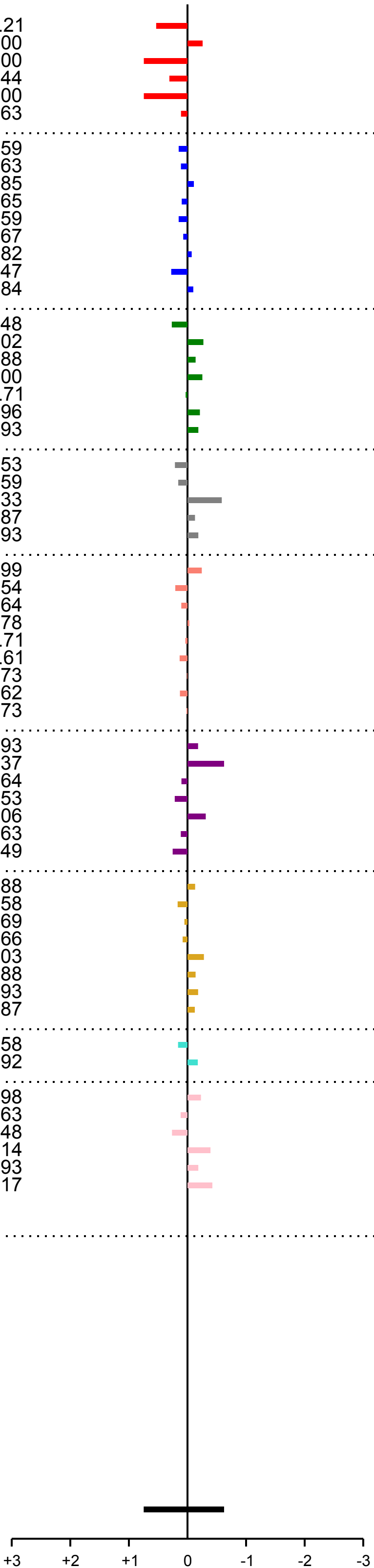

LC50

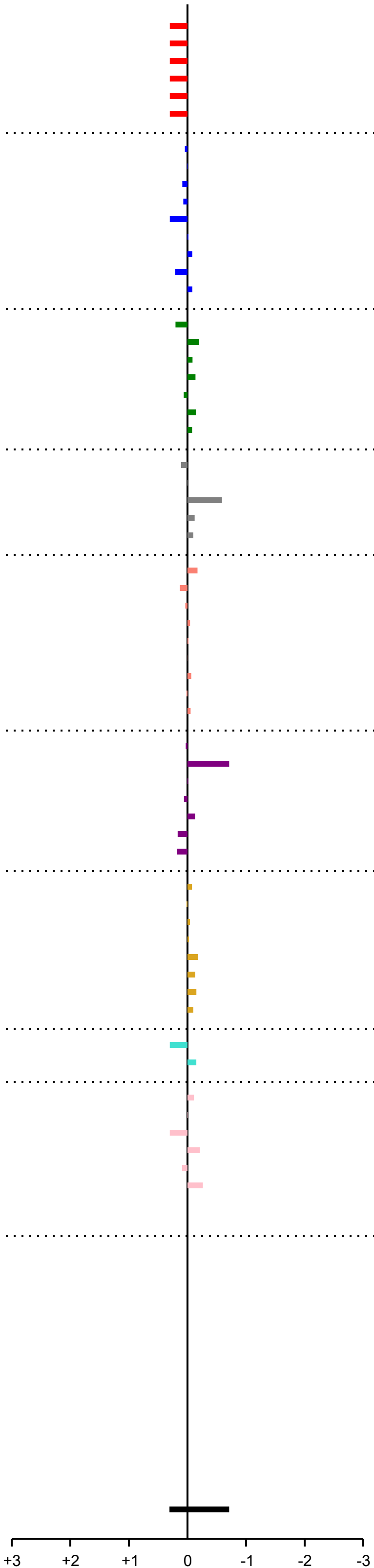

National Cancer Institute Developmental Therapeutics Program
In-Vitro Screening Data Review Checklist

**NSC:**	D - 846306 / 1	**Experiment ID:**	2310NS93
**Test Date:**	October 16, 2023	**Review Date:**	November 29, 2023

Pending Action by the NCI for this experiment

1. ___X___ None
2. _______ Repeat testing in the Primary Screen
3. _______ Refer to Biological Evaluation Committee
4. _______ Currently under Review by Biological Evaluation Committee

National Cancer Institute Developmental Therapeutics Program
In-Vitro Testing Results

NSC : D - 846306 / 1	Experiment ID : 2310NS93	Test Type : 08	Units : Molar
Report Date : November 29, 2023	Test Date : October 16, 2023	QNS :	MC :
COMI : T24	Stain Reagent : SRB Dual-Pass Related	SSPL : 1CXQ	

			Log10 Concentration												
	Time		Mean Optical Densities					Percent Growth							
Panel/Cell Line	Zero	Ctrl	-8.0	-7.0	-6.0	-5.0	-4.0	-8.0	-7.0	-6.0	-5.0	-4.0	GI50	TGI	LC50
**Leukemia**															
CCRF-CEM	0.490	2.299	2.226	2.234	1.998	0.403	0.274	96	96	83	-18	-44	2.14E-6	6.67E-6	> 1.00E-4
HL-60(TB)	0.608	2.555	2.430	2.380	2.180	0.452	0.433	94	91	81	-26	-29	1.95E-6	5.74E-6	> 1.00E-4
K-562	0.271	2.193	2.249	2.211	1.983	0.439	0.325	103	101	89	9	3	3.06E-6	> 1.00E-4	> 1.00E-4
MOLT-4	0.497	2.357	2.297	2.329	2.252	0.718	0.434	97	98	94	12	-13	3.45E-6	3.05E-5	> 1.00E-4
RPMI-8226	0.670	2.386	2.423	2.346	2.058	0.537	0.430	102	98	81	-20	-36	2.03E-6	6.35E-6	> 1.00E-4
SR	0.288	1.558	1.492	1.515	1.375	0.359	0.321	95	97	86	6	3	2.78E-6	> 1.00E-4	> 1.00E-4
**Non-Small Cell Lung Cancer**															
A549/ATCC	0.434	2.158	2.032	2.106	2.075	0.533	0.133	93	97	95	6	-69	3.20E-6	1.19E-5	5.52E-5
EKVX	0.695	1.888	1.843	1.850	1.770	1.143	0.050	96	97	90	38	-93	5.78E-6	1.94E-5	4.69E-5
HOP-62	0.592	1.839	1.786	1.834	1.967	0.706	0.068	96	100	110	9	-89	3.94E-6	1.24E-5	4.03E-5
HOP-92	0.989	1.537	1.477	1.501	1.451	1.133	0.092	89	93	84	26	-91	3.90E-6	1.68E-5	4.49E-5
NCI-H226	1.181	2.650	2.556	2.647	2.480	1.984	0.707	94	100	88	55	-40	1.12E-5	3.77E-5	> 1.00E-4
NCI-H23	0.593	2.276	2.274	2.299	2.211	1.141	0.043	100	101	96	33	-93	5.32E-6	1.82E-5	4.56E-5
NCI-H322M	0.756	2.307	2.198	2.232	2.187	1.492	0.055	93	95	92	47	-93	8.77E-6	2.18E-5	4.96E-5
NCI-H460	0.281	2.710	2.681	2.813	2.678	0.563	0.080	99	104	99	12	-72	3.62E-6	1.38E-5	5.49E-5
NCI-H522	0.878	2.528	2.337	2.493	2.499	1.359	0.090	88	98	98	29	-90	4.99E-6	1.76E-5	4.63E-5
**Colon Cancer**															
COLO 205	0.397	1.953	1.959	2.037	2.008	0.916	0.063	100	105	104	33	-84	5.79E-6	1.92E-5	5.11E-5
HCC-2998	0.734	2.671	2.595	2.669	2.518	0.393	0.020	96	100	92	-46	-97	2.01E-6	4.62E-6	1.17E-5
HCT-116	0.197	2.057	1.985	2.161	1.845	0.281	0.212	96	106	89	4	1	2.88E-6	> 1.00E-4	> 1.00E-4
HCT-15	0.621	2.697	2.607	2.610	2.281	0.561	0.057	96	96	80	-10	-91	2.16E-6	7.79E-6	3.13E-5
HT29	0.294	1.984	1.922	2.067	2.061	0.821	0.079	96	105	105	31	-73	5.54E-6	1.99E-5	5.98E-5
KM12	0.618	2.806	2.863	2.780	2.775	0.886	0.081	103	99	99	12	-87	3.65E-6	1.33E-5	4.24E-5
SW-620	0.274	2.037	1.969	1.993	2.068	0.334	0.063	96	97	102	3	-77	3.36E-6	1.10E-5	4.60E-5
**CNS Cancer**															
SF-268	0.814	2.595	2.369	2.469	2.357	1.745	0.146	87	93	87	52	-82	1.04E-5	2.45E-5	5.77E-5
SF-295	0.925	2.698	2.478	2.512	2.488	1.279	0.030	88	89	88	20	-97	3.62E-6	1.48E-5	3.97E-5
SF-539	0.749	2.320	2.299	2.371	2.298	0.416	0.022	99	103	99	-44	-97	2.19E-6	4.89E-6	1.27E-5
SNB-19	0.653	1.881	1.795	1.849	1.818	1.016	0.011	93	97	95	30	-98	4.86E-6	1.70E-5	4.19E-5
SNB-75	1.119	2.104	1.983	1.977	1.955	0.982	0.027	88	87	85	-12	-98	2.29E-6	7.48E-6	2.77E-5
U251	0.418	1.742	1.642	1.733	1.669	0.032	0.031	92	99	94	-92	-93	1.73E-6	3.20E-6	5.93E-6
**Melanoma**															
LOX IMVI	0.317	2.034	1.920	1.977	1.786	0.035	0.019	93	97	86	-89	-94	1.60E-6	3.09E-6	5.97E-6
MALME-3M	0.636	1.493	1.456	1.452	1.391	1.189	0.068	96	95	88	64	-89	1.24E-5	2.62E-5	5.55E-5
M14	0.360	1.636	1.600	1.656	1.724	0.867	0.152	97	102	107	40	-58	7.04E-6	2.56E-5	8.32E-5
MDA-MB-435	0.558	2.532	2.487	2.486	2.431	1.089	0.053	98	98	95	27	-91	4.57E-6	1.69E-5	4.51E-5
SK-MEL-2	1.711	3.170	3.080	3.140	3.132	2.678	0.132	94	98	97	66	-92	1.27E-5	2.62E-5	5.41E-5
SK-MEL-28	0.725	2.121	2.067	2.119	2.047	1.204	0.028	96	100	95	34	-96	5.49E-6	1.83E-5	4.43E-5
SK-MEL-5	0.822	3.055	2.976	3.058	2.961	2.395	0.012	96	100	96	70	-99	1.32E-5	2.61E-5	5.16E-5
UACC-257	1.052	2.525	2.415	2.465	2.389	1.266	0.044	93	96	91	15	-96	3.42E-6	1.35E-5	3.84E-5
UACC-62	0.769	2.629	2.375	2.388	2.296	1.208	0.037	86	87	82	24	-95	3.54E-6	1.58E-5	4.16E-5
**Ovarian Cancer**															
IGROV1	0.522	2.087	2.026	2.099	1.920	0.646	0.119	96	101	89	8	-77	3.04E-6	1.24E-5	4.79E-5
OVCAR-3	0.582	1.880	1.840	1.870	1.802	0.453	0.032	97	99	94	-22	-95	2.39E-6	6.44E-6	2.42E-5
OVCAR-4	0.788	2.339	2.270	2.365	2.261	1.662	0.055	96	102	95	56	-93	1.10E-5	2.38E-5	5.15E-5
OVCAR-5	0.509	1.315	1.284	1.282	1.266	0.633	0.021	96	96	94	15	-96	3.62E-6	1.37E-5	3.86E-5
OVCAR-8	0.372	1.942	1.903	1.952	1.895	0.488	0.029	97	101	97	7	-92	3.35E-6	1.19E-5	3.77E-5
NCI/ADR-RES	0.508	1.832	1.900	1.986	1.885	0.342	0.323	105	112	104	-33	-36	2.48E-6	5.77E-6	> 1.00E-4
SK-OV-3	0.648	1.644	1.660	1.663	1.672	0.991	0.039	102	102	103	34	-94	5.92E-6	1.85E-5	4.54E-5
**Renal Cancer**															
786-0	0.607	2.603	2.508	2.580	2.529	0.995	0.123	95	99	96	19	-80	4.00E-6	1.57E-5	5.01E-5
A498	1.468	2.417	2.284	2.320	2.302	2.235	0.045	86	90	88	81	-97	1.49E-5	2.85E-5	5.44E-5
ACHN	0.435	1.990	1.894	2.042	1.928	0.746	0.005	94	103	96	20	-99	4.03E-6	1.47E-5	3.88E-5
CAKI-1	1.050	2.706	2.611	2.591	2.632	1.914	0.082	94	93	96	52	-92	1.04E-5	2.30E-5	5.10E-5
RXF 393	1.108	1.820	1.745	1.815	1.702	1.474	0.032	89	99	83	51	-97	1.02E-5	2.22E-5	4.82E-5
SN12C	0.516	1.907	1.950	1.796	1.721	0.527	0.016	103	92	87	1	-97	2.67E-6	1.02E-5	3.31E-5
TK-10	0.961	2.063	1.891	1.940	2.064	1.599	0.028	84	89	100	58	-97	1.12E-5	2.36E-5	4.97E-5
UO-31	0.716	2.162	1.961	2.011	1.907	1.115	0.052	86	90	82	28	-93	3.89E-6	1.69E-5	4.41E-5
**Prostate Cancer**															
PC-3	0.618	2.218	2.174	2.214	2.067	1.012	0.159	97	100	91	25	-74	4.12E-6	1.77E-5	5.68E-5
DU-145	0.276	1.233	1.195	1.296	1.202	0.207	0.021	96	107	97	-25	-92	2.42E-6	6.23E-6	2.35E-5
**Breast Cancer**															
MCF7	1.238	3.172	3.024	3.098	2.979	1.232	0.176	92	96	90	0	-86	2.77E-6	9.88E-6	3.80E-5
MDA-MB-231/ATCC	0.549	1.183	1.181	1.221	1.178	0.745	0.032	100	106	99	31	-94	5.25E-6	1.76E-5	4.43E-5
HS 578T	1.446	2.448	2.349	2.370	2.357	2.194	1.025	90	92	91	75	-29	1.73E-5	5.24E-5	> 1.00E-4
BT-549	1.082	2.299	2.169	2.240	2.231	1.298	0.129	89	95	94	18	-88	3.80E-6	1.47E-5	4.37E-5
T-47D	0.545	1.402	1.335	1.295	1.214	0.467	0.230	92	87	78	-14	-58	2.01E-6	7.00E-6	6.59E-5
MDA-MB-468	1.122	2.636	2.658	2.710	2.519	1.165	0.181	101	105	92	3	-84	2.97E-6	1.08E-5	4.06E-5

National Cancer Institute Developmental Therapeutics Program<br>Dose Response Curves	NSC: D - 846306 / 1	SSPL: 1CXQ	EXP. ID: 2310NS93
	Report Date: November 29, 2023	Test Date: October 16, 2023	

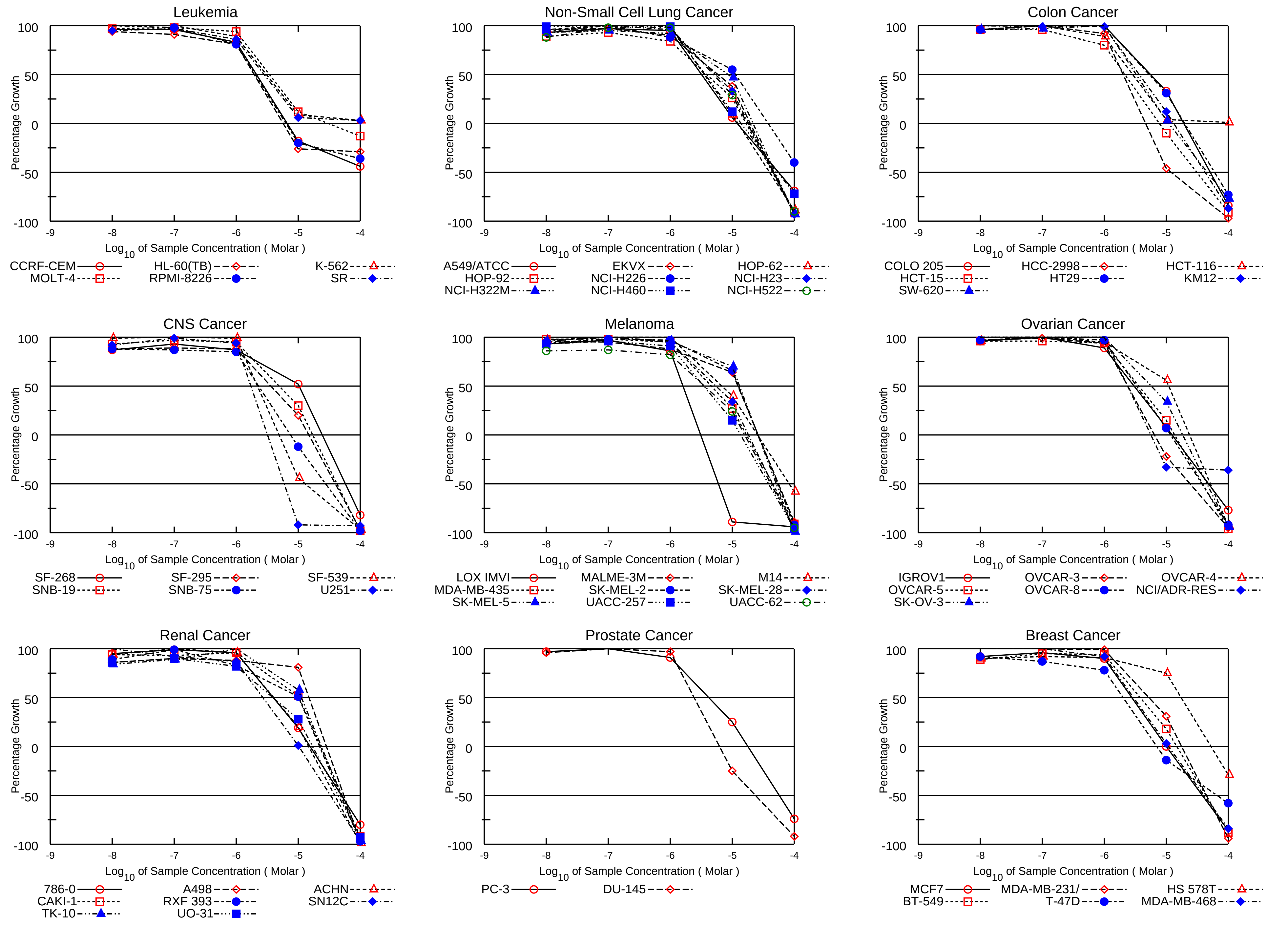

National Cancer Institute Developmental Therapeutics Program
Mean Graphs

NSC : D - 846306/1 | Units :Molar | SSPL :1CXQ | EXP. ID :2310NS93

Report Date :November 29, 2023 | Test Date :October 16, 2023

Panel/Cell Line	Log_{10}GI50	Log_{10}TGI	Log_{10}LC50
**Leukemia**			
CCRF-CEM	-5.67	-5.18	> -4.00
HL-60(TB)	-5.71	-5.24	> -4.00
K-562	-5.51	> -4.00	> -4.00
MOLT-4	-5.46	-4.52	> -4.00
RPMI-8226	-5.69	-5.20	> -4.00
SR	-5.56	> -4.00	> -4.00
**Non-Small Cell Lung Cancer**			
A549/ATCC	-5.49	-4.92	-4.26
EKVX	-5.24	-4.71	-4.33
HOP-62	-5.40	-4.91	-4.39
HOP-92	-5.41	-4.78	-4.35
NCI-H226	-4.95	-4.42	> -4.00
NCI-H23	-5.27	-4.74	-4.34
NCI-H322M	-5.06	-4.66	-4.30
NCI-H460	-5.44	-4.86	-4.26
NCI-H522	-5.30	-4.75	-4.33
**Colon Cancer**			
COLO 205	-5.24	-4.72	-4.29
HCC-2998	-5.70	-5.34	-4.93
HCT-116	-5.54	> -4.00	> -4.00
HCT-15	-5.67	-5.11	-4.50
HT29	-5.26	-4.70	-4.22
KM12	-5.44	-4.88	-4.37
SW-620	-5.47	-4.96	-4.34
**CNS Cancer**			
SF-268	-4.98	-4.61	-4.24
SF-295	-5.44	-4.83	-4.40
SF-539	-5.66	-5.31	-4.89
SNB-19	-5.31	-4.77	-4.38
SNB-75	-5.64	-5.13	-4.56
U251	-5.76	-5.49	-5.23
**Melanoma**			
LOX IMVI	-5.80	-5.51	-5.22
MALME-3M	-4.91	-4.58	-4.26
M14	-5.15	-4.59	-4.08
MDA-MB-435	-5.34	-4.77	-4.35
SK-MEL-2	-4.90	-4.58	-4.27
SK-MEL-28	-5.26	-4.74	-4.35
SK-MEL-5	-4.88	-4.58	-4.29
UACC-257	-5.47	-4.87	-4.42
UACC-62	-5.45	-4.80	-4.38
**Ovarian Cancer**			
IGROV1	-5.52	-4.91	-4.32
OVCAR-3	-5.62	-5.19	-4.62
OVCAR-4	-4.96	-4.62	-4.29
OVCAR-5	-5.44	-4.86	-4.41
OVCAR-8	-5.48	-4.93	-4.42
NCI/ADR-RES	-5.60	-5.24	> -4.00
SK-OV-3	-5.23	-4.73	-4.34
**Renal Cancer**			
786-0	-5.40	-4.80	-4.30
A498	-4.83	-4.55	-4.26
ACHN	-5.39	-4.83	-4.41
CAKI-1	-4.98	-4.64	-4.29
RXF 393	-4.99	-4.65	-4.32
SN12C	-5.57	-4.99	-4.48
TK-10	-4.95	-4.63	-4.30
UO-31	-5.41	-4.77	-4.36
**Prostate Cancer**			
PC-3	-5.39	-4.75	-4.25
DU-145	-5.62	-5.21	-4.63
**Breast Cancer**			
MCF7	-5.56	-5.01	-4.42
MDA-MB-231/ATCC	-5.28	-4.75	-4.35
HS 578T	-4.76	-4.28	> -4.00
BT-549	-5.42	-4.83	-4.36
T-47D	-5.70	-5.15	-4.18
MDA-MB-468	-5.53	-4.97	-4.39
_MID	-5.37	-4.82	-4.34
Delta	0.43	0.69	0.89
Range	1.04	1.51	1.23

GI50
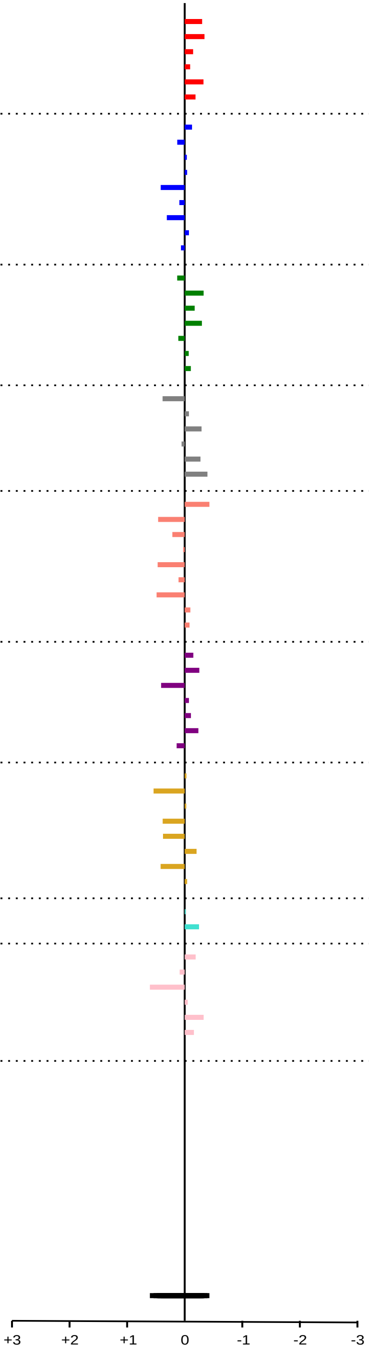

TGI
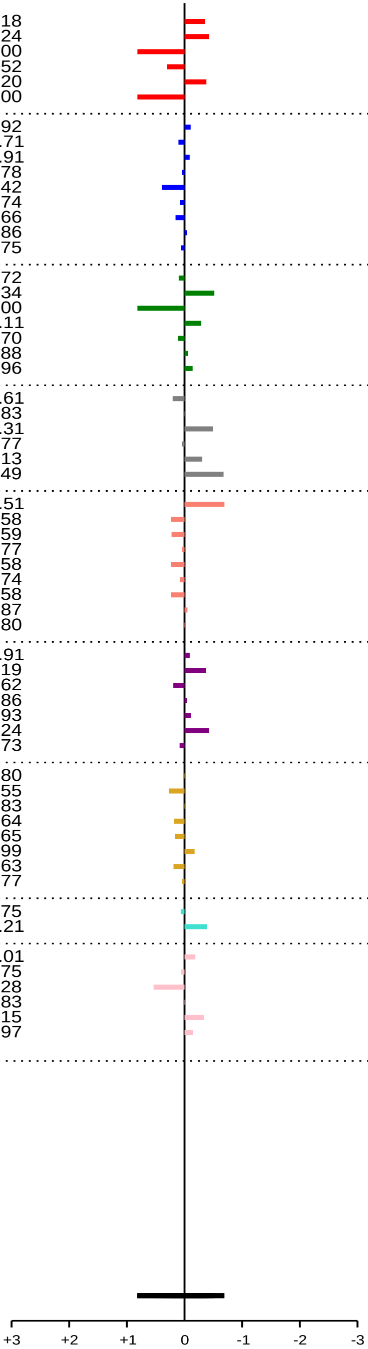

LC50
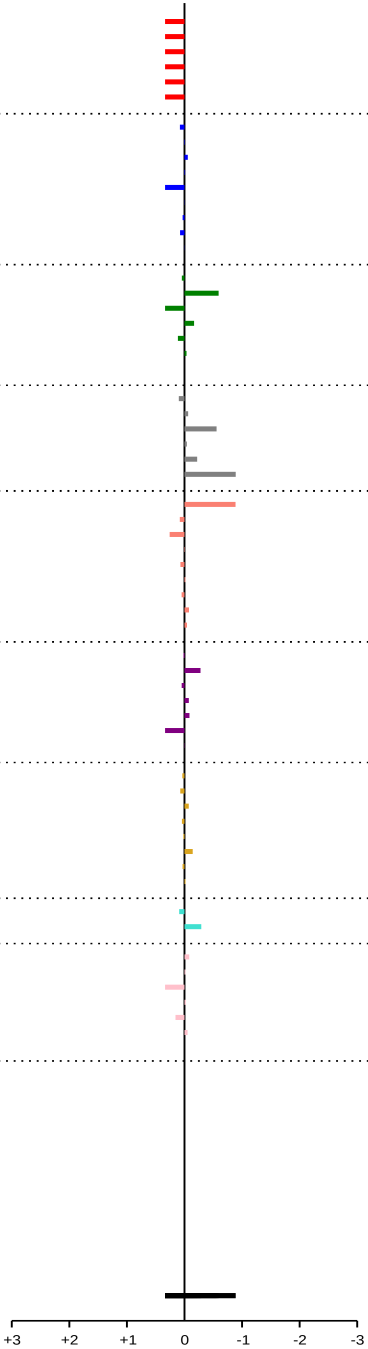

National Cancer Institute Developmental Therapeutics Program<br>Dose Response Curves	NSC : D - 846306/1	SSPL:1CXQ	EXP. ID:2310NS93
	Report Date:November 29, 2023	Test Date:October 16, 2023	

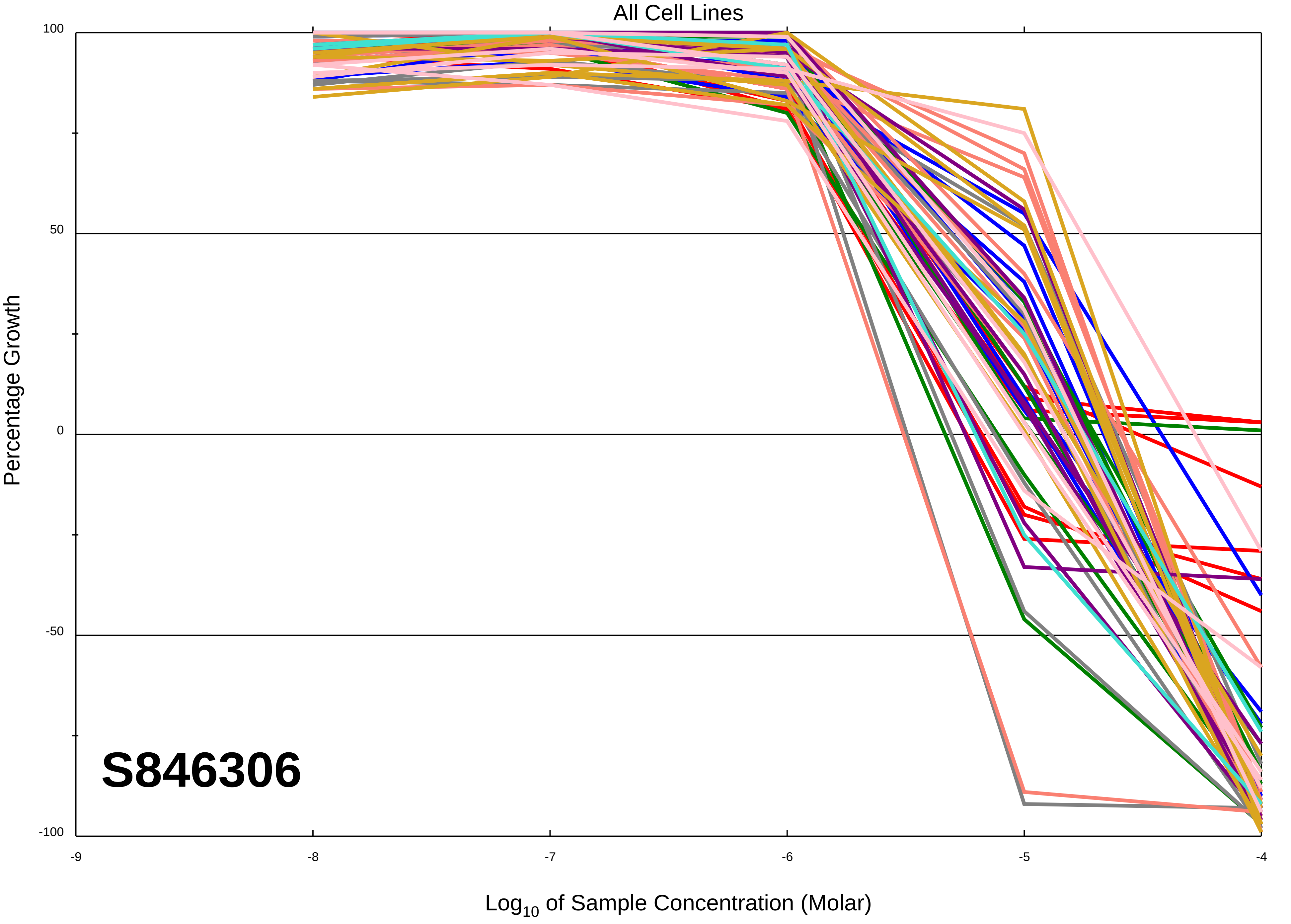

National Cancer Institute Developmental Therapeutics Program<br>Waterfall Graph GI50	NSC : D - 846306/1	Units :Molar	SSPL :1CXQ	EXP. ID :2310NS93
	Report Date :November 29, 2023		Test Date :October 16, 2023	

Panel	Cell Name	Hollow Fiber	GI50
Melanoma	LOX IMVI	*	-5.80
CNS Cancer	U251	*	-5.76
Leukemia	HL-60(TB)		-5.71
Breast Cancer	T-47D		-5.70
Colon Cancer	HCC-2998		-5.70
Leukemia	RPMI-8226		-5.69
Leukemia	CCRF-CEM		-5.67
Colon Cancer	HCT-15		-5.67
CNS Cancer	SF-539		-5.66
CNS Cancer	SNB-75		-5.64
Ovarian Cancer	OVCAR-3	*	-5.62
Prostate Cancer	DU-145		-5.62
Ovarian Cancer	NCI/ADR-RES		-5.60
Renal Cancer	SN12C		-5.57
Breast Cancer	MCF7		-5.56
Leukemia	SR		-5.56
Colon Cancer	HCT-116		-5.54
Breast Cancer	MDA-MB-468		-5.53
Ovarian Cancer	IGROV1		-5.52
Leukemia	K-562		-5.51
Non-Small Cell Lung Cancer	A549/ATCC		-5.49
Ovarian Cancer	OVCAR-8		-5.48
Colon Cancer	SW-620	*	-5.47
Melanoma	UACC-257		-5.47
Leukemia	MOLT-4		-5.46
Melanoma	UACC-62	*	-5.45
Non-Small Cell Lung Cancer	NCI-H460		-5.44
Ovarian Cancer	OVCAR-5	*	-5.44
CNS Cancer	SF-295	*	-5.44
Colon Cancer	KM12		-5.44
Breast Cancer	BT-549		-5.42
Renal Cancer	UO-31		-5.41
Non-Small Cell Lung Cancer	HOP-92		-5.41
Non-Small Cell Lung Cancer	HOP-62		-5.40
Renal Cancer	786-0		-5.40
Renal Cancer	ACHN		-5.39
Prostate Cancer	PC-3		-5.39
Melanoma	MDA-MB-435	*	-5.34
CNS Cancer	SNB-19		-5.31
Non-Small Cell Lung Cancer	NCI-H522	*	-5.30
Breast Cancer	MDA-MB-231/ATCC	*	-5.28
Non-Small Cell Lung Cancer	NCI-H23	*	-5.27
Melanoma	SK-MEL-28		-5.26
Colon Cancer	HT29		-5.26
Non-Small Cell Lung Cancer	EKVX		-5.24
Colon Cancer	COLO 205	*	-5.24
Ovarian Cancer	SK-OV-3		-5.23
Melanoma	M14		-5.15
Non-Small Cell Lung Cancer	NCI-H322M		-5.06
Renal Cancer	RXF 393		-4.99
Renal Cancer	CAKI-1		-4.98
CNS Cancer	SF-268		-4.98
Ovarian Cancer	OVCAR-4		-4.96
Non-Small Cell Lung Cancer	NCI-H226		-4.95
Renal Cancer	TK-10		-4.95
Melanoma	MALME-3M		-4.91
Melanoma	SK-MEL-2		-4.90
Melanoma	SK-MEL-5		-4.88
Renal Cancer	A498		-4.83
Breast Cancer	HS 578T		-4.76

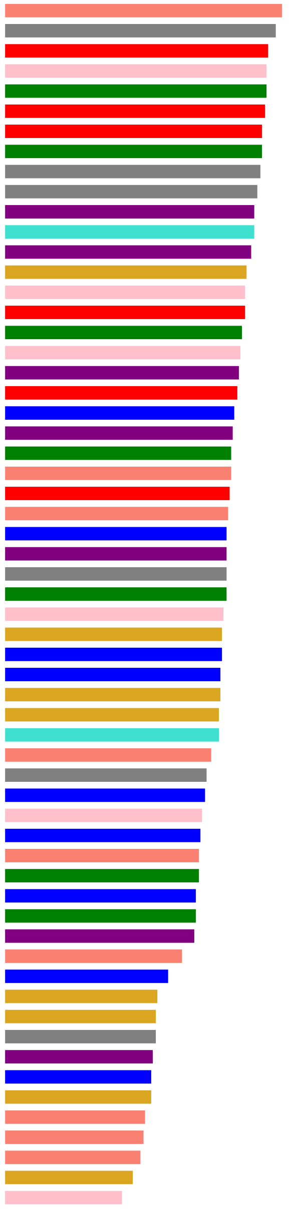

Log10 High Conc :-4.0

National Cancer Institute Developmental Therapeutics Program Waterfall Graph TGI	NSC : D - 846306/1	Units :Molar	SSPL :1CXQ	EXP. ID :2310NS93
	Report Date :November 29, 2023		Test Date :October 16, 2023	

Panel	Cell Name	Hollow Fiber	TGI
Melanoma	LOX IMVI	*	-5.51
CNS Cancer	U251	*	-5.49
Colon Cancer	HCC-2998		-5.34
CNS Cancer	SF-539		-5.31
Leukemia	HL-60(TB)		-5.24
Ovarian Cancer	NCI/ADR-RES		-5.24
Prostate Cancer	DU-145		-5.21
Leukemia	RPMI-8226		-5.20
Ovarian Cancer	OVCAR-3	*	-5.19
Leukemia	CCRF-CEM		-5.18
Breast Cancer	T-47D		-5.15
CNS Cancer	SNB-75		-5.13
Colon Cancer	HCT-15		-5.11
Breast Cancer	MCF7		-5.01
Renal Cancer	SN12C		-4.99
Breast Cancer	MDA-MB-468		-4.97
Colon Cancer	SW-620	*	-4.96
Ovarian Cancer	OVCAR-8		-4.93
Non-Small Cell Lung Cancer	A549/ATCC		-4.92
Ovarian Cancer	IGROV1		-4.91
Non-Small Cell Lung Cancer	HOP-62		-4.91
Colon Cancer	KM12		-4.88
Melanoma	UACC-257		-4.87
Ovarian Cancer	OVCAR-5	*	-4.86
Non-Small Cell Lung Cancer	NCI-H460		-4.86
Breast Cancer	BT-549		-4.83
Renal Cancer	ACHN		-4.83
CNS Cancer	SF-295	*	-4.83
Renal Cancer	786-0		-4.80
Melanoma	UACC-62	*	-4.80
Non-Small Cell Lung Cancer	HOP-92		-4.78
Melanoma	MDA-MB-435	*	-4.77
Renal Cancer	UO-31		-4.77
CNS Cancer	SNB-19		-4.77
Non-Small Cell Lung Cancer	NCI-H522	*	-4.75
Breast Cancer	MDA-MB-231/ATCC	*	-4.75
Prostate Cancer	PC-3		-4.75
Non-Small Cell Lung Cancer	NCI-H23	*	-4.74
Melanoma	SK-MEL-28		-4.74
Ovarian Cancer	SK-OV-3		-4.73
Colon Cancer	COLO 205	*	-4.72
Non-Small Cell Lung Cancer	EKVX		-4.71
Colon Cancer	HT29		-4.70
Non-Small Cell Lung Cancer	NCI-H322M		-4.66
Renal Cancer	RXF 393		-4.65
Renal Cancer	CAKI-1		-4.64
Renal Cancer	TK-10		-4.63
Ovarian Cancer	OVCAR-4		-4.62
CNS Cancer	SF-268		-4.61
Melanoma	M14		-4.59
Melanoma	SK-MEL-5		-4.58
Melanoma	SK-MEL-2		-4.58
Melanoma	MALME-3M		-4.58
Renal Cancer	A498		-4.55
Leukemia	MOLT-4		-4.52
Non-Small Cell Lung Cancer	NCI-H226		-4.42
Breast Cancer	HS 578T		-4.28
Leukemia	K-562		> -4.00
Leukemia	SR		> -4.00
Colon Cancer	HCT-116		> -4.00

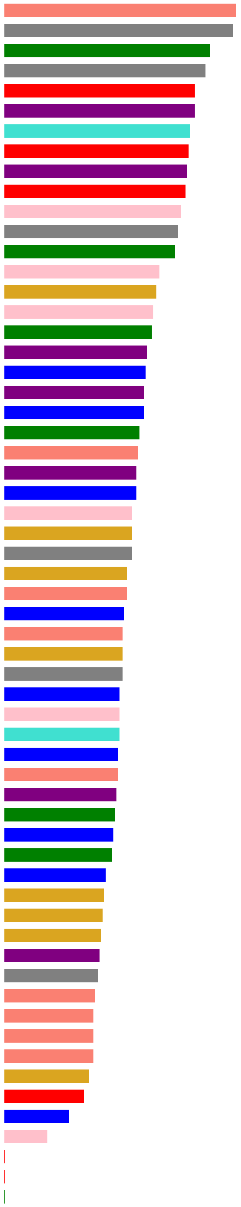

Log10 High Conc : -4.0

National Cancer Institute Developmental Therapeutics Program<br>Waterfall Graph LC50	NSC : D - 846306/1	Units :Molar	SSPL :1CXQ	EXP. ID :2310NS93
	Report Date :November 29, 2023		Test Date :October 16, 2023	

Panel	Cell Name	Hollow Fiber	LC50
CNS Cancer	U251	*	-5.23
Melanoma	LOX IMVI	*	-5.22
Colon Cancer	HCC-2998		-4.93
CNS Cancer	SF-539		-4.89
Prostate Cancer	DU-145		-4.63
Ovarian Cancer	OVCAR-3	*	-4.62
CNS Cancer	SNB-75		-4.56
Colon Cancer	HCT-15		-4.50
Renal Cancer	SN12C		-4.48
Ovarian Cancer	OVCAR-8		-4.42
Breast Cancer	MCF7		-4.42
Melanoma	UACC-257		-4.42
Ovarian Cancer	OVCAR-5	*	-4.41
Renal Cancer	ACHN		-4.41
CNS Cancer	SF-295	*	-4.40
Non-Small Cell Lung Cancer	HOP-62		-4.39
Breast Cancer	MDA-MB-468		-4.39
Melanoma	UACC-62	*	-4.38
CNS Cancer	SNB-19		-4.38
Colon Cancer	KM12		-4.37
Breast Cancer	BT-549		-4.36
Renal Cancer	UO-31		-4.36
Breast Cancer	MDA-MB-231/ATCC	*	-4.35
Melanoma	SK-MEL-28		-4.35
Non-Small Cell Lung Cancer	HOP-92		-4.35
Melanoma	MDA-MB-435	*	-4.35
Ovarian Cancer	SK-OV-3		-4.34
Non-Small Cell Lung Cancer	NCI-H23	*	-4.34
Colon Cancer	SW-620	*	-4.34
Non-Small Cell Lung Cancer	NCI-H522	*	-4.33
Non-Small Cell Lung Cancer	EKVX		-4.33
Ovarian Cancer	IGROV1		-4.32
Renal Cancer	RXF 393		-4.32
Non-Small Cell Lung Cancer	NCI-H322M		-4.30
Renal Cancer	TK-10		-4.30
Renal Cancer	786-0		-4.30
Renal Cancer	CAKI-1		-4.29
Colon Cancer	COLO 205	*	-4.29
Ovarian Cancer	OVCAR-4		-4.29
Melanoma	SK-MEL-5		-4.29
Melanoma	SK-MEL-2		-4.27
Renal Cancer	A498		-4.26
Non-Small Cell Lung Cancer	NCI-H460		-4.26
Non-Small Cell Lung Cancer	A549/ATCC		-4.26
Melanoma	MALME-3M		-4.26
Prostate Cancer	PC-3		-4.25
CNS Cancer	SF-268		-4.24
Colon Cancer	HT29		-4.22
Breast Cancer	T-47D		-4.18
Melanoma	M14		-4.08
Leukemia	CCRF-CEM		> -4.00
Leukemia	HL-60(TB)		> -4.00
Leukemia	K-562		> -4.00
Leukemia	MOLT-4		> -4.00
Leukemia	RPMI-8226		> -4.00
Leukemia	SR		> -4.00
Non-Small Cell Lung Cancer	NCI-H226		> -4.00
Colon Cancer	HCT-116		> -4.00
Ovarian Cancer	NCI/ADR-RES		> -4.00
Breast Cancer	HS 578T		> -4.00

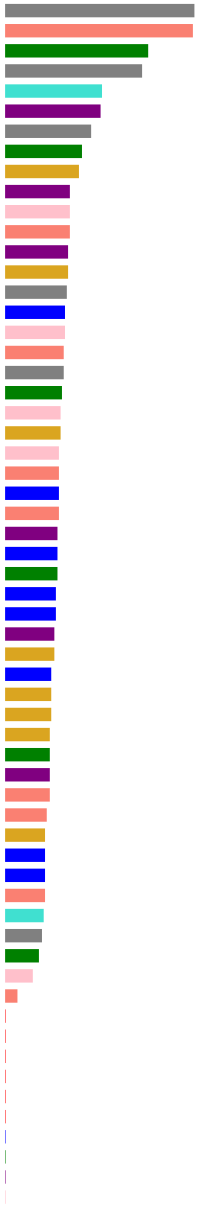

Log10 High Conc : -4.0

National Cancer Institute Developmental Therapeutics Program

Mean Graphs

NSC : D - 841675/1	Units :Molar	SSPL :1CIU	EXP. ID :2306NS34
Report Date :July 23, 2023		Test Date :June 12, 2023	

Panel/Cell Line	Log_{10}GI50	Log_{10}TGI	Log_{10}LC50
**Leukemia**			
CCRF-CEM	-5.34	> -4.00	> -4.00
HL-60(TB)	-5.52	-4.77	> -4.00
K-562	-5.46	> -4.00	> -4.00
MOLT-4	-5.42	> -4.00	> -4.00
RPMI-8226	-5.45	> -4.00	> -4.00
SR	-5.54	> -4.00	> -4.00
**Non-Small Cell Lung Cancer**			
A549/ATCC	-4.77	-4.47	-4.16
EKVX	-4.79	-4.51	-4.24
HOP-62	-5.19	-4.63	-4.17
HOP-92	-4.91	-4.55	-4.19
NCI-H226	-4.81	-4.38	> -4.00
NCI-H23	-4.91	-4.60	-4.28
NCI-H322M	-4.96	-4.61	-4.26
NCI-H460	-4.80	-4.47	-4.14
NCI-H522	-5.03	-4.65	-4.29
**Colon Cancer**			
COLO 205	-4.80	-4.39	> -4.00
HCC-2998	-5.03	-4.67	-4.33
HCT-116	-5.56	-5.10	-4.35
HCT-15	-5.61	-5.10	-4.47
HT29	-4.97	-4.57	-4.16
KM12	-5.42	-4.87	-4.40
SW-620	-5.44	-4.86	-4.35
**CNS Cancer**			
SF-268	-4.81	-4.49	-4.17
SF-295	-4.79	-4.52	-4.25
SF-539	-5.64	-5.15	-4.56
SNB-19	-5.27	-4.74	-4.36
U251	-5.39	-4.77	-4.28
**Melanoma**			
LOX IMVI	-5.73	-5.39	-5.05
MALME-3M	-4.86	-4.51	-4.15
M14	-4.91	-4.55	-4.18
MDA-MB-435	-5.13	-4.68	-4.29
SK-MEL-2	-4.87	-4.54	-4.21
SK-MEL-28	-4.86	-4.57	-4.28
SK-MEL-5	-4.92	-4.61	-4.30
UACC-257	-4.82	-4.52	-4.21
UACC-62	-4.98	-4.65	-4.32
**Ovarian Cancer**			
IGROV1	-5.35	-4.83	-4.29
OVCAR-3	-5.65	-5.28	-4.78
OVCAR-4	-4.83	-4.55	-4.27
OVCAR-5	-4.75	-4.49	-4.24
OVCAR-8	-5.11	-4.62	-4.19
NCI/ADR-RES	-4.83	-4.45	-4.08
SK-OV-3	-4.73	-4.42	-4.11
**Renal Cancer**			
786-0	-5.40	-4.75	-4.27
A498	-4.81	-4.53	-4.26
ACHN	-4.95	-4.63	-4.31
CAKI-1	-4.86	-4.57	-4.28
RXF 393	-5.31	-4.73	-4.35
SN12C	-5.22	-4.71	-4.35
TK-10	-4.91	-4.60	-4.29
UO-31	-5.31	-4.73	-4.33
**Prostate Cancer**			
PC-3	-4.99	-4.43	> -4.00
DU-145	-5.47	-4.96	-4.46
**Breast Cancer**			
MCF7	-5.57	-4.93	-4.29
MDA-MB-231/ATCC	-4.92	-4.61	-4.29
HS 578T	-4.89	-4.49	-4.09
BT-549	-5.30	-4.71	-4.31
T-47D	-5.30	-4.59	> -4.00
MDA-MB-468	-5.66	-5.10	-4.50
_MID	-5.13	-4.62	-4.25
Delta	0.6	0.77	0.8
Range	1.0	1.39	1.05

Axis (GI50, TGI, LC50): +3 +2 +1 0 -1 -2 -3

National Cancer Institute Developmental Therapeutics Program
In-Vitro Screening Data Review Checklist

**NSC:** D - 845697 / 1	**Experiment ID:** 2310NS90
**Test Date:** October 10, 2023	**Review Date:** November 22, 2023

Pending Action by the NCI for this experiment

1. ___X___ None
2. _______ Repeat testing in the Primary Screen
3. _______ Refer to Biological Evaluation Committee
4. _______ Currently under Review by Biological Evaluation Committee

National Cancer Institute Developmental Therapeutics Program
In-Vitro Testing Results

NSC : D - 845697 / 1	Experiment ID : 2310NS90	Test Type : 08	Units : Molar
Report Date : November 23, 2023	Test Date : October 10, 2023	QNS :	MC :
COMI : T2	Stain Reagent : SRB Dual-Pass Related	SSPL : 1CIU	

			Log10 Concentration												
	Time		Mean Optical Densities					Percent Growth							
Panel/Cell Line	Zero	Ctrl	-8.0	-7.0	-6.0	-5.0	-4.0	-8.0	-7.0	-6.0	-5.0	-4.0	GI50	TGI	LC50
**Leukemia**															
CCRF-CEM	0.316	1.559	1.530	1.528	1.374	0.300	0.219	98	98	85	-5	-31	2.45E-6	8.75E-6	> 1.00E-4
HL-60(TB)	0.474	2.108	1.953	1.965	1.844	0.500	0.339	90	91	84	2	-28	2.58E-6	1.13E-5	> 1.00E-4
K-562	0.135	1.095	1.066	1.065	0.992	0.205	0.152	97	97	89	7	2	3.02E-6	> 1.00E-4	> 1.00E-4
MOLT-4	0.532	2.572	2.553	2.527	2.302	0.657	0.474	99	98	87	6	-11	2.86E-6	2.27E-5	> 1.00E-4
RPMI-8226	0.801	2.470	2.487	2.415	2.093	0.777	0.615	101	97	77	-3	-23	2.19E-6	9.16E-6	> 1.00E-4
SR	0.510	2.210	2.069	2.027	1.832	0.571	0.440	92	89	78	4	-14	2.37E-6	1.61E-5	> 1.00E-4
**Non-Small Cell Lung Cancer**															
A549/ATCC	0.316	1.624	1.468	1.578	1.546	0.502	0.050	88	96	94	14	-84	3.56E-6	1.39E-5	4.49E-5
EKVX	0.746	2.138	1.972	2.041	1.992	1.096	0.413	88	93	89	25	-45	4.11E-6	2.29E-5	> 1.00E-4
HOP-62	0.472	1.638	1.490	1.554	1.558	0.547	0.360	87	93	93	6	-24	3.15E-6	1.63E-5	> 1.00E-4
HOP-92	1.030	1.761	1.687	1.732	1.645	1.115	0.757	90	96	84	12	-27	2.95E-6	2.01E-5	> 1.00E-4
NCI-H226	1.191	2.430	2.297	2.355	2.293	1.424	1.015	89	94	89	19	-15	3.59E-6	3.63E-5	> 1.00E-4
NCI-H23	0.671	2.162	2.079	2.155	2.065	0.691	0.148	94	99	93	1	-78	2.96E-6	1.04E-5	4.44E-5
NCI-H322M	0.838	2.196	2.069	2.078	2.015	1.265	0.140	91	91	87	31	-83	4.60E-6	1.88E-5	5.12E-5
NCI-H460	0.323	1.874	1.915	2.021	1.698	0.226	0.127	103	109	89	-30	-61	2.12E-6	5.58E-6	4.45E-5
NCI-H522	0.964	2.512	2.353	2.448	2.368	1.097	0.124	90	96	91	9	-87	3.13E-6	1.23E-5	4.09E-5
**Colon Cancer**															
COLO 205	0.530	2.023	1.997	2.085	2.057	1.755	0.139	98	104	102	82	-74	1.61E-5	3.36E-5	7.04E-5
HCC-2998	1.164	3.429	3.421	3.435	3.428	1.832	0.078	100	100	100	29	-93	5.11E-6	1.74E-5	4.44E-5
HCT-116	0.194	2.343	2.183	2.192	2.074	0.455	0.075	93	93	87	12	-62	3.14E-6	1.46E-5	6.96E-5
HCT-15	0.361	2.455	2.245	2.316	2.189	0.646	0.147	90	93	87	14	-59	3.21E-6	1.54E-5	7.43E-5
HT29	0.203	1.102	1.002	1.106	1.101	0.268	0.105	89	100	100	7	-49	3.45E-6	1.35E-5	> 1.00E-4
KM12	0.662	2.174	2.059	2.061	1.961	0.887	0.080	92	93	86	15	-88	3.20E-6	1.39E-5	4.27E-5
SW-620	0.309	1.478	1.351	1.368	1.304	0.306	0.080	89	91	85	0	-74	2.56E-6	9.74E-6	4.68E-5
**CNS Cancer**															
SF-268	0.932	2.455	2.304	2.321	2.212	0.991	0.293	90	91	84	4	-69	2.66E-6	1.13E-5	5.54E-5
SF-295	1.028	2.906	2.640	2.795	2.822	1.673	0.322	86	94	96	34	-69	5.55E-6	2.15E-5	6.58E-5
SF-539	0.944	2.534	2.446	2.522	2.414	0.637	0.126	94	99	92	-33	-87	2.19E-6	5.49E-6	2.10E-5
SNB-19	0.787	2.163	2.011	2.141	2.058	1.229	0.024	89	98	92	32	-97	5.04E-6	1.77E-5	4.33E-5
SNB-75	1.063	1.867	1.821	1.828	1.765	0.823	0.189	94	95	87	-23	-82	2.18E-6	6.23E-6	2.88E-5
U251	0.232	1.194	1.150	1.188	1.089	0.301	0.043	95	99	89	7	-82	3.00E-6	1.20E-5	4.40E-5
**Melanoma**															
LOX IMVI	0.425	2.135	2.123	2.127	2.033	0.556	0.045	99	100	94	8	-90	3.23E-6	1.20E-5	3.92E-5
MALME-3M	0.650	1.496	1.410	1.484	1.430	0.958	0.125	90	99	92	36	-81	5.71E-6	2.05E-5	5.46E-5
M14	0.408	1.613	1.534	1.675	1.537	0.506	0.166	93	105	94	8	-59	3.24E-6	1.32E-5	7.25E-5
MDA-MB-435	0.636	2.232	2.199	2.194	1.966	0.656	0.322	98	98	83	1	-49	2.55E-6	1.06E-5	> 1.00E-4
SK-MEL-2	1.172	2.386	2.282	2.383	2.304	1.240	0.199	91	100	93	6	-83	3.11E-6	1.16E-5	4.24E-5
SK-MEL-28	0.693	2.111	2.026	2.070	2.004	1.013	0.186	94	97	92	23	-73	4.05E-6	1.72E-5	5.72E-5
SK-MEL-5	1.062	2.951	2.631	2.689	2.478	0.533	0.123	83	86	75	-50	-88	1.58E-6	3.99E-6	1.01E-5
UACC-257	1.151	2.573	2.463	2.511	2.505	1.602	0.446	92	96	95	32	-61	5.15E-6	2.19E-5	7.56E-5
UACC-62	0.704	2.414	2.160	2.225	2.144	0.854	0.112	85	89	84	9	-84	2.84E-6	1.24E-5	4.29E-5
**Ovarian Cancer**															
IGROV1	0.591	1.994	1.969	1.951	1.933	0.706	0.306	98	97	96	8	-48	3.32E-6	1.40E-5	> 1.00E-4
OVCAR-3	0.858	2.205	2.096	2.200	2.100	0.711	0.017	92	100	92	-17	-98	2.43E-6	6.97E-6	2.55E-5
OVCAR-4	0.794	2.198	2.149	2.155	1.982	0.906	0.210	97	97	85	8	-74	2.83E-6	1.25E-5	5.14E-5
OVCAR-5	0.643	1.492	1.466	1.435	1.422	0.813	0.063	97	93	92	20	-90	3.82E-6	1.52E-5	4.32E-5
OVCAR-8	0.495	2.158	1.992	2.151	2.005	0.336	0.203	90	100	91	-32	-59	2.15E-6	5.47E-6	4.61E-5
NCI/ADR-RES	0.372	1.246	1.226	1.228	1.148	0.322	0.221	98	98	89	-13	-41	2.40E-6	7.39E-6	> 1.00E-4
SK-OV-3	0.624	1.529	1.440	1.495	1.573	0.999	0.647	90	96	105	41	2	7.34E-6	> 1.00E-4	> 1.00E-4
**Renal Cancer**															
786-0	0.440	2.297	2.198	2.320	2.318	0.884	0.216	95	101	101	24	-51	4.59E-6	2.09E-5	9.72E-5
A498	1.307	2.124	1.994	1.989	2.034	1.764	0.673	84	83	89	56	-49	1.14E-5	3.43E-5	> 1.00E-4
ACHN	0.426	1.815	1.713	1.812	1.715	0.713	0.107	93	100	93	21	-75	3.92E-6	1.64E-5	5.48E-5
CAKI-1	0.462	1.783	1.594	1.674	1.477	0.606	0.085	86	92	77	11	-82	2.55E-6	1.31E-5	4.54E-5
RXF 393	1.053	1.687	1.617	1.637	1.654	0.881	0.162	89	92	95	-16	-85	2.53E-6	7.12E-6	3.11E-5
SN12C	0.552	2.066	1.844	1.936	1.747	0.544	0.142	85	91	79	-1	-74	2.29E-6	9.59E-6	4.64E-5
TK-10	0.990	2.146	2.023	2.046	2.153	1.323	0.120	89	91	101	29	-88	5.06E-6	1.76E-5	4.73E-5
UO-31	0.704	2.184	1.860	1.980	1.854	0.997	0.113	78	86	78	20	-84	3.01E-6	1.55E-5	4.70E-5
**Prostate Cancer**															
PC-3	0.414	1.665	1.636	1.638	1.629	0.789	0.339	98	98	97	30	-18	5.03E-6	4.20E-5	> 1.00E-4
DU-145	0.339	1.266	1.185	1.197	1.148	0.474	0.129	91	93	87	15	-62	3.25E-6	1.55E-5	6.98E-5
**Breast Cancer**															
MCF7	0.420	1.952	1.751	1.836	1.862	0.457	0.098	87	92	94	2	-77	3.03E-6	1.07E-5	4.60E-5
MDA-MB-231/ATCC	0.549	1.183	1.180	1.206	1.148	0.590	0.295	100	104	94	6	-46	3.20E-6	1.33E-5	> 1.00E-4
HS 578T	1.661	2.700	2.527	2.524	2.488	1.825	1.488	83	83	80	16	-10	2.91E-6	4.00E-5	> 1.00E-4
BT-549	1.156	2.242	2.160	2.226	2.161	1.179	0.318	92	99	93	2	-73	2.95E-6	1.07E-5	4.99E-5
T-47D	0.631	1.458	1.335	1.323	1.253	0.645	0.556	85	84	75	2	-12	2.20E-6	1.33E-5	> 1.00E-4
MDA-MB-468	1.202	1.981	1.879	1.905	1.751	0.756	0.138	87	90	70	-37	-89	1.55E-6	4.52E-6	1.78E-5

National Cancer Institute Developmental Therapeutics Program
Dose Response Curves

NSC: D - 845697 / 1	SSPL: 1CIU	EXP. ID: 2310NS90
Report Date: November 23, 2023	Test Date: October 10, 2023	

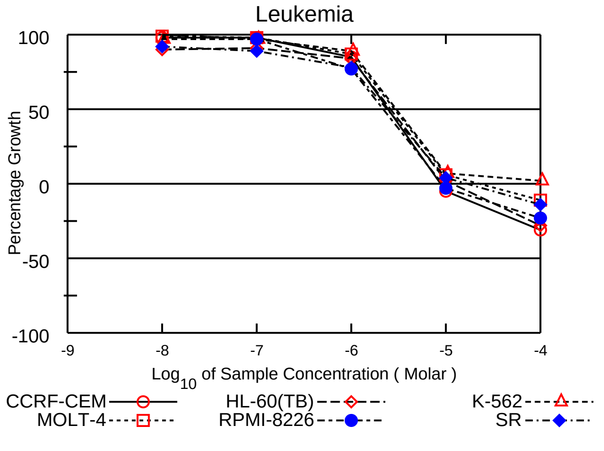

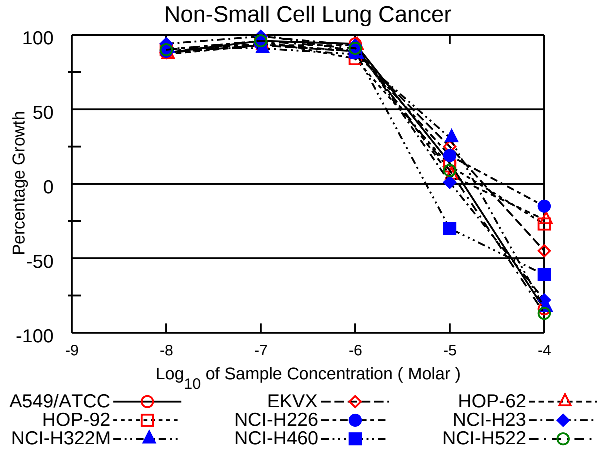

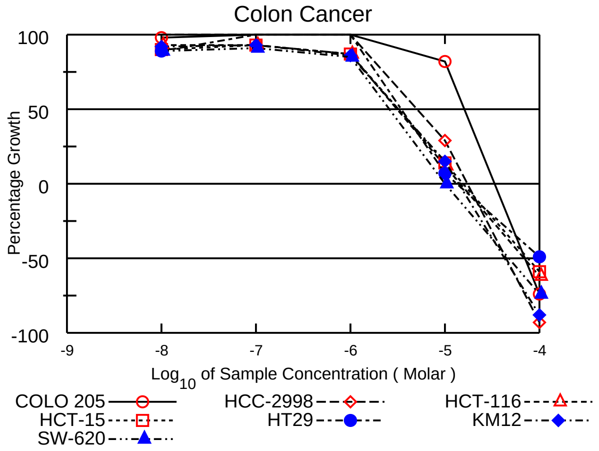

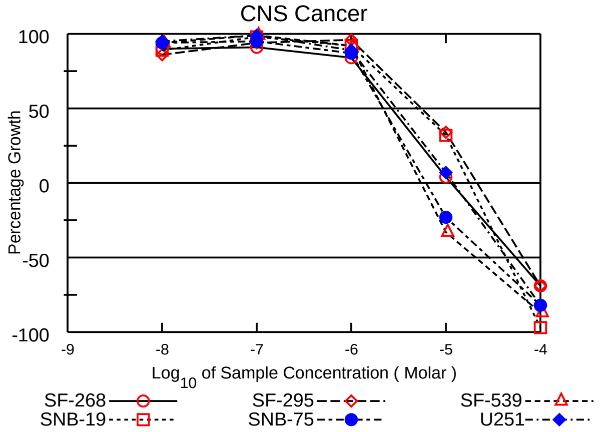

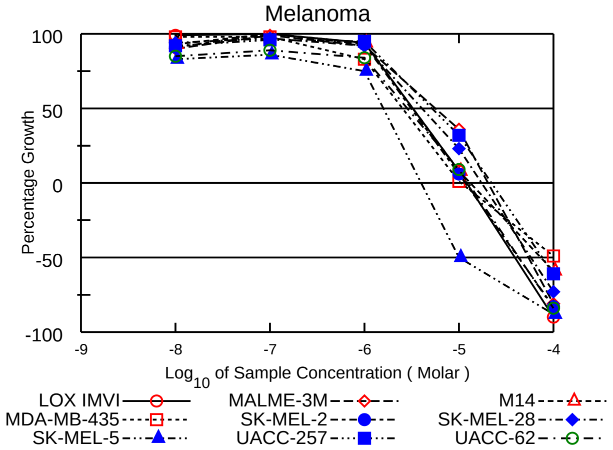

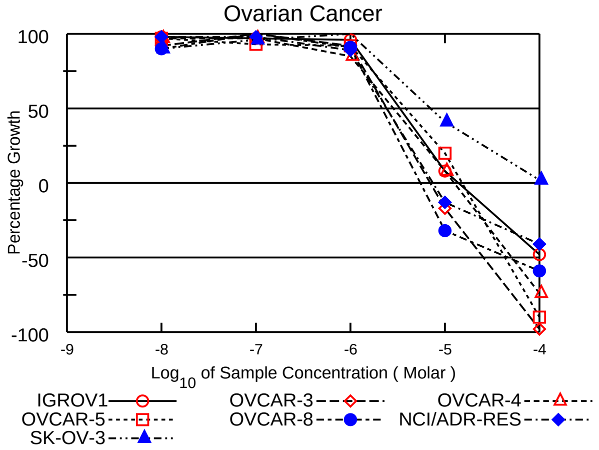

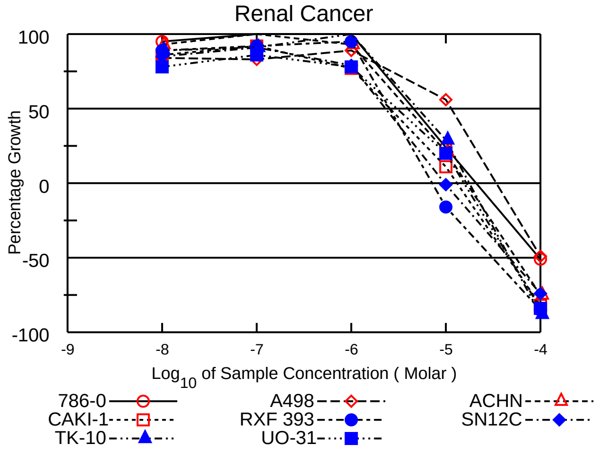

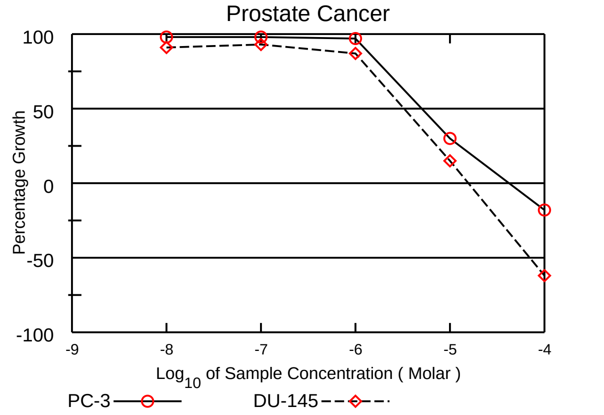

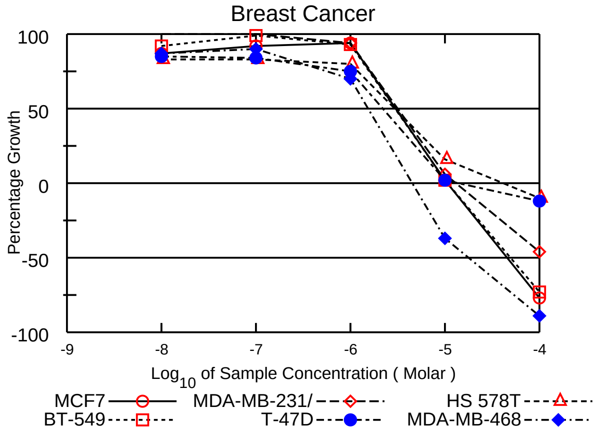

National Cancer Institute Developmental Therapeutics Program
Mean Graphs

NSC : D - 845697/1	Units :Molar	SSPL :1CIU	EXP. ID :2310NS90
Report Date :November 23, 2023		Test Date :October 10, 2023	

Panel/Cell Line	Log_{10}GI50	Log_{10}TGI	Log_{10}LC50
**Leukemia**			
CCRF-CEM	-5.61	-5.06	> -4.00
HL-60(TB)	-5.59	-4.95	> -4.00
K-562	-5.52	> -4.00	> -4.00
MOLT-4	-5.54	-4.64	> -4.00
RPMI-8226	-5.66	-5.04	> -4.00
SR	-5.63	-4.79	> -4.00
**Non-Small Cell Lung Cancer**			
A549/ATCC	-5.45	-4.86	-4.35
EKVX	-5.39	-4.64	> -4.00
HOP-62	-5.50	-4.79	> -4.00
HOP-92	-5.53	-4.70	> -4.00
NCI-H226	-5.44	-4.44	> -4.00
NCI-H23	-5.53	-4.98	-4.35
NCI-H322M	-5.34	-4.73	-4.29
NCI-H460	-5.67	-5.25	-4.35
NCI-H522	-5.50	-4.91	-4.39
**Colon Cancer**			
COLO 205	-4.79	-4.47	-4.15
HCC-2998	-5.29	-4.76	-4.35
HCT-116	-5.50	-4.84	-4.16
HCT-15	-5.49	-4.81	-4.13
HT29	-5.46	-4.87	> -4.00
KM12	-5.49	-4.86	-4.37
SW-620	-5.59	-5.01	-4.33
**CNS Cancer**			
SF-268	-5.58	-4.95	-4.26
SF-295	-5.26	-4.67	-4.18
SF-539	-5.66	-5.26	-4.68
SNB-19	-5.30	-4.75	-4.36
SNB-75	-5.66	-5.21	-4.54
U251	-5.52	-4.92	-4.36
**Melanoma**			
LOX IMVI	-5.49	-4.92	-4.41
MALME-3M	-5.24	-4.69	-4.26
M14	-5.49	-4.88	-4.14
MDA-MB-435	-5.59	-4.98	> -4.00
SK-MEL-2	-5.51	-4.94	-4.37
SK-MEL-28	-5.39	-4.76	-4.24
SK-MEL-5	-5.80	-5.40	-5.00
UACC-257	-5.29	-4.66	-4.12
UACC-62	-5.55	-4.91	-4.37
**Ovarian Cancer**			
IGROV1	-5.48	-4.86	> -4.00
OVCAR-3	-5.61	-5.16	-4.59
OVCAR-4	-5.55	-4.90	-4.29
OVCAR-5	-5.42	-4.82	-4.36
OVCAR-8	-5.67	-5.26	-4.34
NCI/ADR-RES	-5.62	-5.13	> -4.00
SK-OV-3	-5.13	> -4.00	> -4.00
**Renal Cancer**			
786-0	-5.34	-4.68	-4.01
A498	-4.94	-4.46	> -4.00
ACHN	-5.41	-4.78	-4.26
CAKI-1	-5.59	-4.88	-4.34
RXF 393	-5.60	-5.15	-4.51
SN12C	-5.64	-5.02	-4.33
TK-10	-5.30	-4.75	-4.32
UO-31	-5.52	-4.81	-4.33
**Prostate Cancer**			
PC-3	-5.30	-4.38	> -4.00
DU-145	-5.49	-4.81	-4.16
**Breast Cancer**			
MCF7	-5.52	-4.97	-4.34
MDA-MB-231/ATCC	-5.49	-4.88	> -4.00
HS 578T	-5.54	-4.40	> -4.00
BT-549	-5.53	-4.97	-4.30
T-47D	-5.66	-4.88	> -4.00
MDA-MB-468	-5.81	-5.35	-4.75
_MID	-5.48	-4.84	-4.23
Delta	0.33	0.56	0.77
Range	1.02	1.4	1.0

GI50
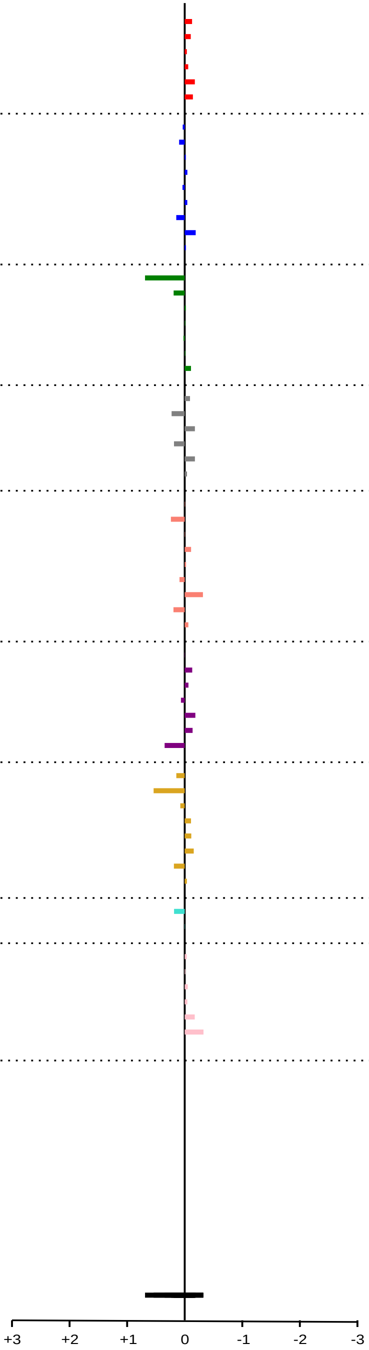

TGI
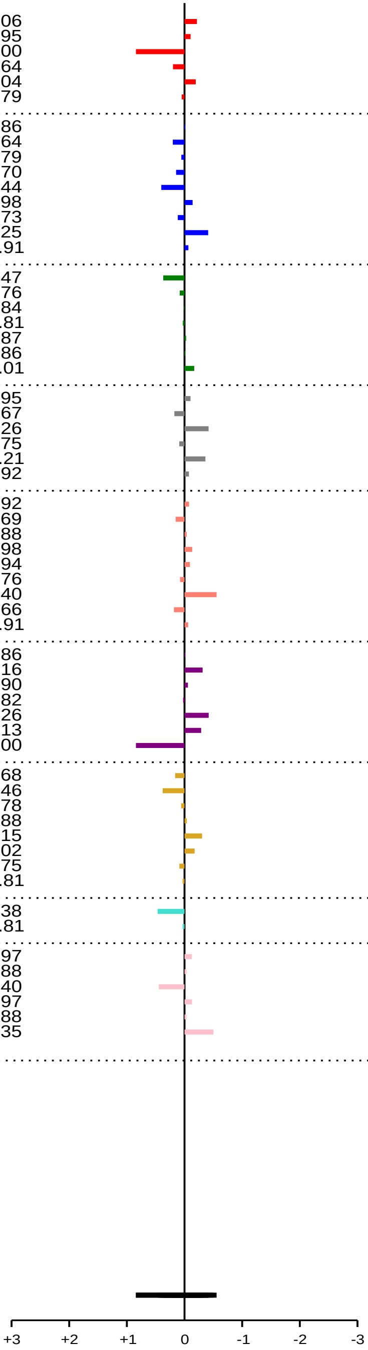

LC50
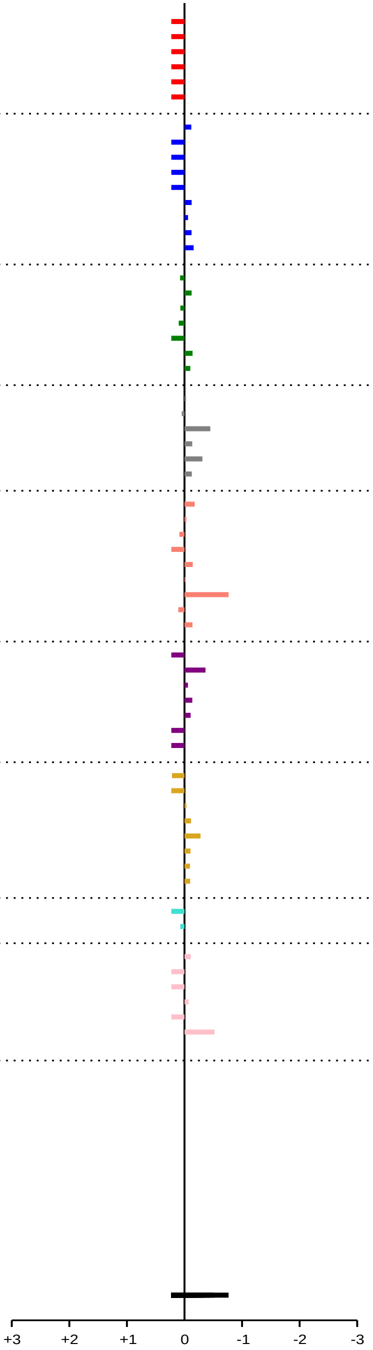

National Cancer Institute Developmental Therapeutics Program Dose Response Curves	NSC : D - 845697/1	SSPL:1CIU	EXP. ID:2310NS90
	Report Date:November 23, 2023	Test Date:October 10, 2023	

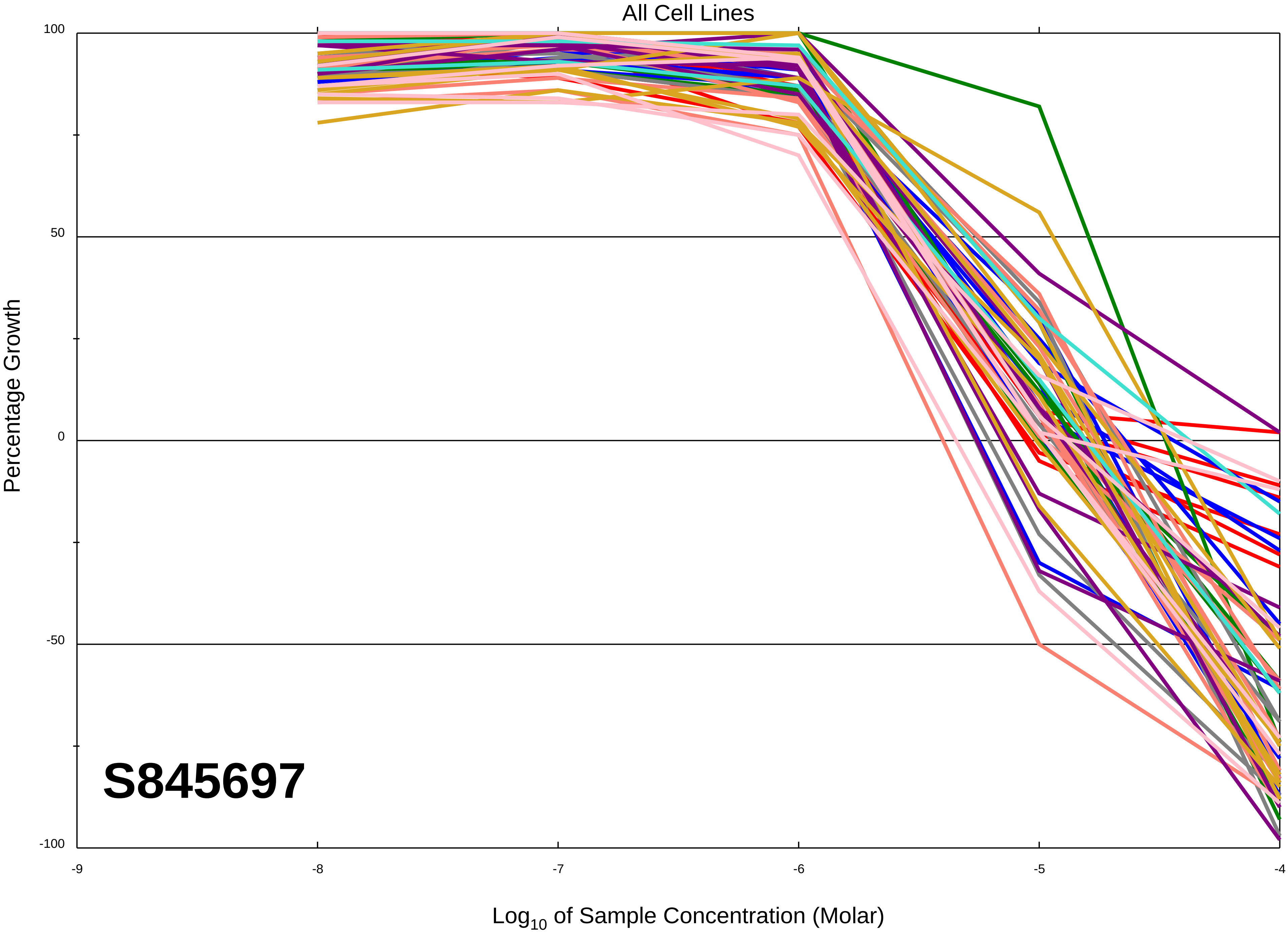

National Cancer Institute Developmental Therapeutics Program<br>Waterfall Graph GI50	NSC : D - 845697/1	Units :Molar	SSPL :1CIU	EXP. ID :2310NS90
	Report Date :November 23, 2023		Test Date :October 10, 2023	

Panel	Cell Name	Hollow Fiber	GI50
Breast Cancer	MDA-MB-468		-5.81
Melanoma	SK-MEL-5		-5.80
Non-Small Cell Lung Cancer	NCI-H460		-5.67
Ovarian Cancer	OVCAR-8		-5.67
CNS Cancer	SNB-75		-5.66
CNS Cancer	SF-539		-5.66
Leukemia	RPMI-8226		-5.66
Breast Cancer	T-47D		-5.66
Renal Cancer	SN12C		-5.64
Leukemia	SR		-5.63
Ovarian Cancer	NCI/ADR-RES		-5.62
Ovarian Cancer	OVCAR-3	*	-5.61
Leukemia	CCRF-CEM		-5.61
Renal Cancer	RXF 393		-5.60
Melanoma	MDA-MB-435	*	-5.59
Renal Cancer	CAKI-1		-5.59
Colon Cancer	SW-620	*	-5.59
Leukemia	HL-60(TB)		-5.59
CNS Cancer	SF-268		-5.58
Ovarian Cancer	OVCAR-4		-5.55
Melanoma	UACC-62	*	-5.55
Leukemia	MOLT-4		-5.54
Breast Cancer	HS 578T		-5.54
Non-Small Cell Lung Cancer	HOP-92		-5.53
Breast Cancer	BT-549		-5.53
Non-Small Cell Lung Cancer	NCI-H23	*	-5.53
CNS Cancer	U251	*	-5.52
Renal Cancer	UO-31		-5.52
Leukemia	K-562		-5.52
Breast Cancer	MCF7		-5.52
Melanoma	SK-MEL-2		-5.51
Non-Small Cell Lung Cancer	NCI-H522	*	-5.50
Colon Cancer	HCT-116		-5.50
Non-Small Cell Lung Cancer	HOP-62		-5.50
Colon Cancer	KM12		-5.49
Breast Cancer	MDA-MB-231/ATCC	*	-5.49
Colon Cancer	HCT-15		-5.49
Melanoma	LOX IMVI	*	-5.49
Melanoma	M14		-5.49
Prostate Cancer	DU-145		-5.49
Ovarian Cancer	IGROV1		-5.48
Colon Cancer	HT29		-5.46
Non-Small Cell Lung Cancer	A549/ATCC		-5.45
Non-Small Cell Lung Cancer	NCI-H226		-5.44
Ovarian Cancer	OVCAR-5	*	-5.42
Renal Cancer	ACHN		-5.41
Melanoma	SK-MEL-28		-5.39
Non-Small Cell Lung Cancer	EKVX		-5.39
Renal Cancer	786-0		-5.34
Non-Small Cell Lung Cancer	NCI-H322M		-5.34
Prostate Cancer	PC-3		-5.30
CNS Cancer	SNB-19		-5.30
Renal Cancer	TK-10		-5.30
Colon Cancer	HCC-2998		-5.29
Melanoma	UACC-257		-5.29
CNS Cancer	SF-295	*	-5.26
Melanoma	MALME-3M		-5.24
Ovarian Cancer	SK-OV-3		-5.13
Renal Cancer	A498		-4.94
Colon Cancer	COLO 205	*	-4.79

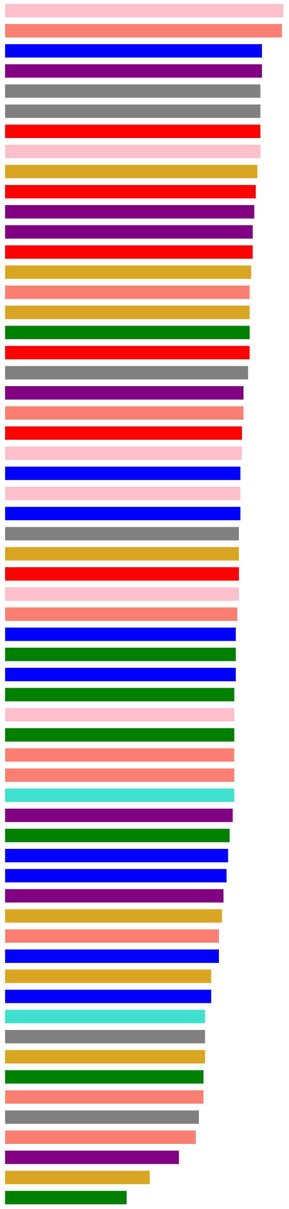

Log10 High Conc :-4.0

National Cancer Institute Developmental Therapeutics Program<br>Waterfall Graph TGI	NSC : D - 845697/1	Units :Molar	SSPL :1CIU	EXP. ID :2310NS90
	Report Date :November 23, 2023		Test Date :October 10, 2023	

Panel	Cell Name	Hollow Fiber	TGI
Melanoma	SK-MEL-5		-5.40
Breast Cancer	MDA-MB-468		-5.35
Ovarian Cancer	OVCAR-8		-5.26
CNS Cancer	SF-539		-5.26
Non-Small Cell Lung Cancer	NCI-H460		-5.25
CNS Cancer	SNB-75		-5.21
Ovarian Cancer	OVCAR-3	*	-5.16
Renal Cancer	RXF 393		-5.15
Ovarian Cancer	NCI/ADR-RES		-5.13
Leukemia	CCRF-CEM		-5.06
Leukemia	RPMI-8226		-5.04
Renal Cancer	SN12C		-5.02
Colon Cancer	SW-620	*	-5.01
Non-Small Cell Lung Cancer	NCI-H23	*	-4.98
Melanoma	MDA-MB-435	*	-4.98
Breast Cancer	BT-549		-4.97
Breast Cancer	MCF7		-4.97
Leukemia	HL-60(TB)		-4.95
CNS Cancer	SF-268		-4.95
Melanoma	SK-MEL-2		-4.94
Melanoma	LOX IMVI	*	-4.92
CNS Cancer	U251	*	-4.92
Non-Small Cell Lung Cancer	NCI-H522	*	-4.91
Melanoma	UACC-62	*	-4.91
Ovarian Cancer	OVCAR-4		-4.90
Renal Cancer	CAKI-1		-4.88
Melanoma	M14		-4.88
Breast Cancer	MDA-MB-231/ATCC	*	-4.88
Breast Cancer	T-47D		-4.88
Colon Cancer	HT29		-4.87
Colon Cancer	KM12		-4.86
Non-Small Cell Lung Cancer	A549/ATCC		-4.86
Ovarian Cancer	IGROV1		-4.86
Colon Cancer	HCT-116		-4.84
Ovarian Cancer	OVCAR-5	*	-4.82
Colon Cancer	HCT-15		-4.81
Prostate Cancer	DU-145		-4.81
Renal Cancer	UO-31		-4.81
Leukemia	SR		-4.79
Non-Small Cell Lung Cancer	HOP-62		-4.79
Renal Cancer	ACHN		-4.78
Melanoma	SK-MEL-28		-4.76
Colon Cancer	HCC-2998		-4.76
Renal Cancer	TK-10		-4.75
CNS Cancer	SNB-19		-4.75
Non-Small Cell Lung Cancer	NCI-H322M		-4.73
Non-Small Cell Lung Cancer	HOP-92		-4.70
Melanoma	MALME-3M		-4.69
Renal Cancer	786-0		-4.68
CNS Cancer	SF-295	*	-4.67
Melanoma	UACC-257		-4.66
Leukemia	MOLT-4		-4.64
Non-Small Cell Lung Cancer	EKVX		-4.64
Colon Cancer	COLO 205	*	-4.47
Renal Cancer	A498		-4.46
Non-Small Cell Lung Cancer	NCI-H226		-4.44
Breast Cancer	HS 578T		-4.40
Prostate Cancer	PC-3		-4.38
Leukemia	K-562		> -4.00
Ovarian Cancer	SK-OV-3		> -4.00

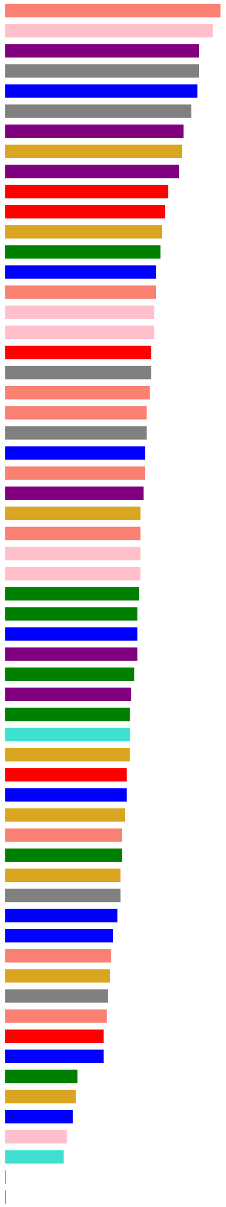

Log10 High Conc : -4.0

National Cancer Institute Developmental Therapeutics Program
Waterfall Graph LC50

NSC : D - 845697/1	Units :Molar	SSPL :1CIU	EXP. ID :2310NS90
Report Date :November 23, 2023		Test Date :October 10, 2023	

Panel	Cell Name	Hollow Fiber	LC50
Melanoma	SK-MEL-5		-5.00
Breast Cancer	MDA-MB-468		-4.75
CNS Cancer	SF-539		-4.68
Ovarian Cancer	OVCAR-3	*	-4.59
CNS Cancer	SNB-75		-4.54
Renal Cancer	RXF 393		-4.51
Melanoma	LOX IMVI	*	-4.41
Non-Small Cell Lung Cancer	NCI-H522	*	-4.39
Melanoma	SK-MEL-2		-4.37
Colon Cancer	KM12		-4.37
Melanoma	UACC-62	*	-4.37
Ovarian Cancer	OVCAR-5	*	-4.36
CNS Cancer	SNB-19		-4.36
CNS Cancer	U251	*	-4.36
Non-Small Cell Lung Cancer	NCI-H23	*	-4.35
Colon Cancer	HCC-2998		-4.35
Non-Small Cell Lung Cancer	NCI-H460		-4.35
Non-Small Cell Lung Cancer	A549/ATCC		-4.35
Renal Cancer	CAKI-1		-4.34
Breast Cancer	MCF7		-4.34
Ovarian Cancer	OVCAR-8		-4.34
Renal Cancer	SN12C		-4.33
Colon Cancer	SW-620	*	-4.33
Renal Cancer	UO-31		-4.33
Renal Cancer	TK-10		-4.32
Breast Cancer	BT-549		-4.30
Non-Small Cell Lung Cancer	NCI-H322M		-4.29
Ovarian Cancer	OVCAR-4		-4.29
Melanoma	MALME-3M		-4.26
Renal Cancer	ACHN		-4.26
CNS Cancer	SF-268		-4.26
Melanoma	SK-MEL-28		-4.24
CNS Cancer	SF-295	*	-4.18
Colon Cancer	HCT-116		-4.16
Prostate Cancer	DU-145		-4.16
Colon Cancer	COLO 205	*	-4.15
Melanoma	M14		-4.14
Colon Cancer	HCT-15		-4.13
Melanoma	UACC-257		-4.12
Renal Cancer	786-0		-4.01
Leukemia	CCRF-CEM		> -4.00
Leukemia	HL-60(TB)		> -4.00
Leukemia	K-562		> -4.00
Leukemia	MOLT-4		> -4.00
Leukemia	RPMI-8226		> -4.00
Leukemia	SR		> -4.00
Non-Small Cell Lung Cancer	EKVX		> -4.00
Non-Small Cell Lung Cancer	HOP-62		> -4.00
Non-Small Cell Lung Cancer	HOP-92		> -4.00
Non-Small Cell Lung Cancer	NCI-H226		> -4.00
Colon Cancer	HT29		> -4.00
Melanoma	MDA-MB-435	*	> -4.00
Ovarian Cancer	IGROV1		> -4.00
Ovarian Cancer	NCI/ADR-RES		> -4.00
Ovarian Cancer	SK-OV-3		> -4.00
Renal Cancer	A498		> -4.00
Prostate Cancer	PC-3		> -4.00
Breast Cancer	MDA-MB-231/ATCC	*	> -4.00
Breast Cancer	HS 578T		> -4.00
Breast Cancer	T-47D		> -4.00

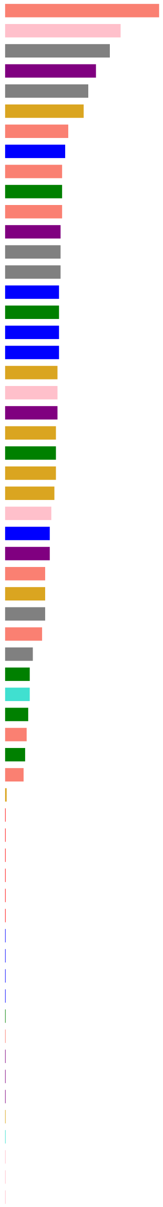

Log10 High Conc : -4.0
